# Ultrasonic-Assisted Extraction of Polysaccharides from *Brassica rapa* L. and Its Effects on Gut Microbiota in Humanized Mice

**DOI:** 10.3390/foods14111994

**Published:** 2025-06-05

**Authors:** Mengying Zhang, Wei Wang, Wei Li, Zhipeng Wang, Kaiyue Bi, Yanbo Li, Yuhan Wu, Yu Zhao, Rui Yang, Qingping Du

**Affiliations:** College of Food Science and Pharmacy, Xinjiang Agricultural University, Urumqi 830052, China; zmy991013@163.com (M.Z.);

**Keywords:** *Brassica rapa* L. polysaccharide, ultrasound-assisted extraction, structural characterization, gut microbiota

## Abstract

This study optimized ultrasound-assisted extraction (UAE) for polysaccharide isolation from *Brassica rapa* L. using Box–Behnken design, achieving a maximum yield of 41.12% under conditions of 60 °C, 60 min, 175 W ultrasonic power, and 30 mL/g liquid–solid ratios. The crude polysaccharide (BRAP) was purified via DEAE-52 cellulose and Sephadex G-100 chromatography, yielding BRAP1-1 with the highest recovery rate. Structural analyses (FT-IR, HPGPC, SEM, SEC-MALLS-RI) identified BRAP1-1 as a β-glycosidic pyranose polysaccharide (32.55 kDa) composed of fucose, rhamnose, arabinose, galactose, and galacturonic acid (molar ratio 0.81:4.30:3.61:1.69:89.59). In a humanized mouse model via fecal microbiota transplantation (FMT), BRAP1-1 significantly increased α-diversity indices (ACE, Chao1; *p* < 0.05) and altered β-diversity, with PCA explaining 73% variance (PC1: 60.70%, PC2: 13.53%). BRAP1-1 elevated beneficial genera (*Lysinibacillus*, *Solibacillus*, *Bacteroides*, etc.) while suppressing pathogens (*Treponema*, *Flavobacterium*, etc.). Six genera, including *[Eubacterium]_coprostanoligenes_group* and *Bacteroidales* (*p* < 0.05), correlated with acetic/propionic acid production. These findings demonstrate BRAP1-1’s potential to modulate gut microbiota composition and enhance intestinal homeostasis.

## 1. Introduction

*Brassica rapa* L. is a radish-tuber plant cultivated in high-altitude Chinese regions, including the Tianshan Mountains, Tarim Basin, and Tibet [1]. Valued as both vegetable and traditional medicine, its protein-rich roots exhibit medicinal properties such as detoxification, anti-cancer, and blood pressure regulation [2]. Recent research focuses on bioactive components like polysaccharides (BRAP), showing anti-fatigue [3], antioxidant [4], and immunomodulatory activities [5].

Traditional hot-water polysaccharide extraction suffers from inefficiency, long duration, and low yields [6]. Emerging methods like microwave, enzymatic, and ultrasonic-assisted extraction (UAE) address these limitations [7]. UAE stands out due to its rapid processing, enhanced efficiency, and preserved bioactivity through synergistic mechanical vibration, cavitation, and thermal effects [8]. These mechanisms disrupt cell walls, improve solvent penetration, and boost compound release by optimizing diffusivity and mass transfer [9]. However, its thermal effect during the extraction process is relatively weak, requiring prolonged durations to reach the desired temperature levels [10,11]. Within a certain range, the polysaccharide yield increases with rising ultrasonic intensity, processing time, and extraction temperature [12]. However, under high-intensity ultrasonication, polysaccharide structures may undergo partial modification. Existing studies demonstrate that ultrasound-assisted extraction reduces solution viscosity and particle size of polysaccharides [13,14] while altering their monosaccharide composition [15]. Furthermore, ultrasonication generates degraded polysaccharide fragments [16], yet interestingly enhances their bioactivity in most cases [17].

Therefore, process optimization of polysaccharide extraction using ultrasound-assisted methods remains a scientifically valuable research objective. However, current research predominantly examines isolated ultrasonic parameters rather than systematically evaluating operational modes and frequencies. Comprehensive optimization of these variables is critical to maximize extraction performance.

Studies indicate that most macromolecular polysaccharides evade complete digestion in the upper gastrointestinal tract but are metabolized by gut microbiota via glycosidases and functional proteins [18]. Polysaccharides also modulate microbial diversity and abundance by nourishing specific bacterial taxa [19]. As gut microbiota is a complex ecosystem critical to host health, animal models are commonly used to study human-microbiota interactions [20]. However, anatomical, physiological, and genetic differences between mice and humans limit translatability. Humanized microbiota models, established by colonizing germ-free mice with human fecal microbiota through repeated oral gavage, provide a physiologically relevant platform to investigate BRAP-microbiota interactions in vivo [21].

In the present research, we established an efficient method for obtaining BRAP and investigating its effects on the gut microbiota of humanized mice. BRAP was extracted from *Brassica rapa* L. using UAE, with extraction conditions optimized through response surface methodology (RSM). The structural properties of BRAP were characterized using chemical and spectroscopic techniques. Subsequently, the influence of BRAP on the gut microbiota of humanized mouse models was systematically evaluated. High-throughput 16S rDNA sequencing analysis was employed to identify taxonomic shifts in bacterial communities. This research not only offers a novel perspective for understanding the interplay between BRAP and the gut microbiota but also furnishes scientific rationale for the investigation and development of BRAP-containing functional foods.

## 2. Materials and Methods

### 2.1. Materials and Reagents

The fresh *Brassica rapa* L. was obtained from a farmer’s market (Urumqi, Xinjiang, China). The roots were botanically identified as *Brassica rapa* L. by Associate Professor Wei Wang from the College of Food Science and Pharmacy, Xinjiang Agricultural University. DEAE-52 cellulose and Sephadex G-100 dextran gel were purchased from Shanghai Yuanye Bio-Technology Co., Ltd. (Shanghai, China). Monosaccharide standards were obtained from Sigma-Aldrich (St. Louis, MO, USA). Methanol, acetonitrile, formic acid, isopropanol, and ammonium acetate were purchased from Thermo Fisher Scientific (Waltham, MA, USA). Other reagents used were analytical grade.

### 2.2. Extraction Process

Based on the method of Wang et al. [22] with some modifications. Fresh *Brassica rapa* L. roots were thoroughly washed, peeled, sliced, and dried to constant weight at 50 °C in a thermostatic air dryer. The dried material was pulverized and sieved. Extraction was performed with 85% ethanol at a liquid-to-solid ratios of 30 mL/g at 25 °C for 24 h per cycle, repeated three times. The extracted material was subsequently dried and stored for further use.

The extracts obtained under different ultrasound-assisted extraction conditions were centrifuged (4000 rpm, 10 min) to collect the supernatant. After concentration to 1/8 of the original volume using a rotary evaporator, four volumes of absolute ethanol (relative to the concentrated sample volume) were added, and the mixture was kept at 4 °C overnight, followed by centrifugation (4000 rpm, 10 min) to collect the precipitate. The precipitate was then sequentially washed with diethyl ether, absolute ethanol, and acetone, and dried at 50 °C to obtain crude BRAP. To eliminate interference from impurities and obtain highly purified polysaccharide fractions with well-defined structures, thereby ensuring the reliability of biological function studies and practical applications, the crude BRAP was subjected to isolation and purification. The crude BRAP (500 mg) was dissolved in 10 mL of deionized water and loaded onto a DEAE-52 cellulose column (2.6 × 30 cm). Fractionation was carried out using a stepwise NaCl gradient (0, 0.1, 0.3, and 0.5 mol/L) at a flow rate of 1.0 mL/min, with 10 mL fractions collected. The major fractions were pooled, concentrated, and lyophilized to yield three purified fractions (BRAP-1, BRAP-2, and BRAP-3). The major fraction BRAP-1 was filtered through a 0.45 μm aqueous membrane and subsequently purified on a Sephadex G-100 column (1.6 × 100 cm) at a flow rate of 0.5 mL/min, with 5 mL fractions collected. The purified fraction BRAP1-1 was obtained through this step.

### 2.3. Determination of Chemical Composition

The biochemical composition of BRAPs was quantified using established spectrophotometric methods: total sugars (phenol-sulfuric acid method with glucose standard) [23], proteins (Bradford assay with BSA standard) [24], as well as uronic acids (m-hydroxybiphenyl method) and sulfate groups (barium chloride-gelatin method) [25].

### 2.4. Determination of Monosaccharide and Molecular Weight

Approximately 5 mg of the sample was hydrolyzed with trifluoroacetic acid (2 M) at 121 °C for 2 h in a sealed tube. The sample was dried by a stream of nitrogen gas. Methanol adding and blow drying was repeated twice. The residue was re-dissolved in deionized water and filtered through 0.22 μm microporous filtering film for measurement [26]. The sample extracts were analyzed by high-performance anion-exchange chromatography (HPAEC) on a CarboPac PA-20 anion-exchange column (3 by 150 mm; Dionex) using a pulsed amperometric detector (PAD; Dionex ICS 5000 + system). Flow rate, 0.5 mL/min; injection volume, 5 μL; solvent system A: (dd H_2_O), solvent system B: (0.1 M NaOH), solvent system C: (0.1 M NaOH, 0.2 M NaAc).

The polysaccharide molecular weight (Mw) analysis followed previously reported methodologies [27]. The molecular weight of various fractions was measured using SEC-MALLS-RI. The molecular weight (Mw) of various fractions in 0.1 M NaNO_3_ aqueous solution containing 0.02% NaN_3_ were measured on a DAWN HELEOS-II laser photometer (Wyatt Technology Co., Santa Barbara, CA, USA) equipped with two tandem columns (300 × 8 mm, Shodex OH-pak SB-805 and 803) which was held at 45 °C using a model column heater by Sanshu Biotech. Co., Ltd. (Shanghai, China). The flow rate is 0.6 mL/min (or 0.3 mL/min).

### 2.5. FT-IR and SEM Analysis

The polysaccharide FT-IR analysis followed previously established protocols as described in prior literature [28]. The dried BRAP1-1 samples (2 mg) were weighed and mixed individually with a small amount of KBr solid. The mixtures were then analyzed using a Nicolet iZ-10 Fourier transform infrared spectrometer with a scanning range of 4000–400 cm^−1^.

In addition, the high-resolution field emission scanning electron microscope (Carl Zeiss AG, Zeiss Merlin Compact, Oberkochen, BW, Germany) equipped with a scanning electron detector was utilized to capture SEM images across multiple magnification levels [29].

### 2.6. Animal Experiments

Ethical approval for this animal study was granted by Xinjiang Agricultural University’s Institutional Animal Care and Use Committee (Protocol ID: 2024011). The Laboratory Animal Center of Xinjiang Medical University supplied male germ-free KM mice aged 5–8 weeks and weighing 20 ± 2 g (experimental animal use license: SCXK (XIN) 2023-0001). During the adaptive feeding in the clean-grade animal room, the mice had free access to standard feed and water.

#### Construction of a Humanized Microbiota Mice Model

Fresh fecal samples were provided by three healthy volunteers who had not used antibiotics within the previous three months. Fecal microbiota transplantation (FMT) material was prepared as described with modifications [30]: fresh fecal samples (1.0 g) were homogenized in 9 mL sterile PBS (pH 7.2), followed by centrifugation (800× *g*, 10 min) of pellet particulate matter. The samples were cryopreserved at −80 °C and subsequently used for further experiments.

By gavaging 60 germ-free KM mice with human fecal suspension (0.3 mL per mouse), the human gut microbiota was allowed to colonize the mouse intestines for a period of 3 weeks, resulting in the establishment of the HFA (human flora-associated) mouse model [31]. During the experimental period, the mice had free access to food and sterile water. Subsequently, sixty HFA mice were randomly allocated into four treatment groups (n = 15 per cohort). Throughout the experiment, the mice were gavaged at a fixed time daily with varying treatments: the low-dose BRAP1-1 group (LB group, 50 mg/kg), the high-dose BRAP1-1 group (HB group, 200 mg/kg), the fructooligosaccharide positive control group (FOS group, 100 mg/kg), and the blank control group (CK group, gavaged daily with the same volume of sterile saline). This gavaging protocol was maintained for 3 consecutive weeks. Sample collection occurred post-experiment, with murine feces immediately cryopreserved (−80 °C) for later examination.

### 2.7. Measurement of Short-Chain Fatty Acids (SCFAs)

The method was adapted from Martínez-Reyes et al. with slight modifications [32], as follows: the fecal samples from different experimental groups were lyophilized using liquid nitrogen and ground into fine powder. Subsequently, 100 mg of each sample was weighed and covered with liquid nitrogen, followed by the addition of an appropriate volume of 80% aqueous methanol. The mixture was vortexed vigorously for 30 s to ensure homogenization before complete evaporation of the liquid nitrogen and centrifuged at 12,000 rpm for 10 min to remove the protein. A 50 μL aliquot of the supernatant was mixed with 150 μL of derivatization reagent (containing three components: (1) 3-nitrophenylhydrazine-d4 (3-NPH-d4) at 160 mM in 80% aqueous methanol (*v*/*v*), (2) N-(3-dimethylaminopropyl)-N’-ethylcarbodiimide (EDC) at 120 mM in anhydrous methanol, and (3) pyridine at 8% (*v*/*v*) in methanol, with 50 μL of each reagent being added) and derivatized at 40 °C for 40 min. The sample was diluted with 80% (*v*/*v*) aqueous methanol and subsequently centrifuged (12,000 rpm, 4 °C, 10 min). Then, the supernatant (95 μL) was homogenized with 5 μL mixed internal standard solution. Finally, it was injected into the LC-MS system for analysis. An ultra-high performance liquid chromatography coupled to tandem mass spectrometry (UHPLC-MS) system (Vanquish™ Flex UHPLC-TSQ Altis™, Thermo Scientific Corp., Bremen Germany) was used to quantitate SCFAs in Novogene Co., Ltd. (Beijing, China). Separation was performed on a Waters ACQUITY UPLC BEH C18 column (2.1 × 100 mm, 1.7 μm) which was maintained at 40 °C. The mobile phase, consisting of 10 mM ammonium acetate in water (solvent A) and acetonitrile:isopropanol (1:1) (solvent B), was delivered at a flow rate of 0.30 mL/min.

### 2.8. 16S rDNA Gene and Bioinformatics Analysis

Five fecal samples from each group were collected and transported on dry ice to Shanghai Tianhao Biotechnology Co., Ltd. (Shanghai, China) for sequencing. DNA extraction and quality assessment of the fecal genome were performed using a standard microbial genomic DNA extraction kit. PCR amplification was conducted on the bacterial 16S rDNA gene within the V3-V4 region using forward primers (Illumina adapter sequence 1 + CCTACGGGNGGCWGCAG) and reverse primers (Illumina adapter sequence 2 + GACTACHVGGGTATCTAATCC) [33]. Finally, sequencing and data analysis were carried out using the Illumina 2 × 250 bp paired-end sequencing strategy.

### 2.9. Statistical Analysis

Data are expressed as mean ± SD and subjected to parametric analysis. All statistical evaluations and graphical visualizations were executed using IBM SPSS Statistics 27 (SPSS Inc., Chicago, IL, USA) and Origin Software Version 2021 (Origin Lab Corp., Northampton, MA, USA), respectively. A *p*-value < 0.05 was established as the threshold for statistical significance.

## 3. Results

### 3.1. Preparation Process of BRAP

Based on preliminary experiments, single-factor experiments were conducted to investigate the key parameters affecting the extraction yield of BRAP, including ultrasonic temperature (A), liquid-to-solid ratios (B), and ultrasonic time (C). The results showed that the polysaccharide yield gradually increased with liquid-to-solid ratios ranging from 20 mL/g to 30 mL/g, achieving a maximum at 30 mL/g. The yield initially rose with increasing ultrasonic temperature, peaking at 60 °C, followed by a decline at higher temperatures. Similarly, the yield reached its highest at an ultrasonic time of 60 min. Extended ultrasonic durations or elevated temperatures induced the breakdown of glycosidic bonds [34], causing polysaccharide degradation and a subsequent reduction in yield. Based on the single-factor experimental results, a response surface model was established with the BRAP yield as the response value. The quadratic polynomial regression equation for A, B, C, and BRAP content (Y) was obtained as follows:

Y = + 41.18 − 2.02A − 0.230B + 1.30C + 0.91AB − 1.22AC + 1.99BC − 2.43A^2^ − 2.32B^2^ − 3.72C^2.^

Preliminary analysis of variance (ANOVA) results revealed the model’s *p*-value was < 0.0001, indicating a highly significant overall fit. The R^2^ value of 0.9739 demonstrated that the model accounts for approximately 97.39% of the variability in the data, reflecting an excellent fit between the model and the experimental observation. Furthermore, the adjusted R^2^ (R^2^_Adj_) value of 0.9404 confirmed the model’s high fitting accuracy and predictive capability [35].

**Figure 1:**
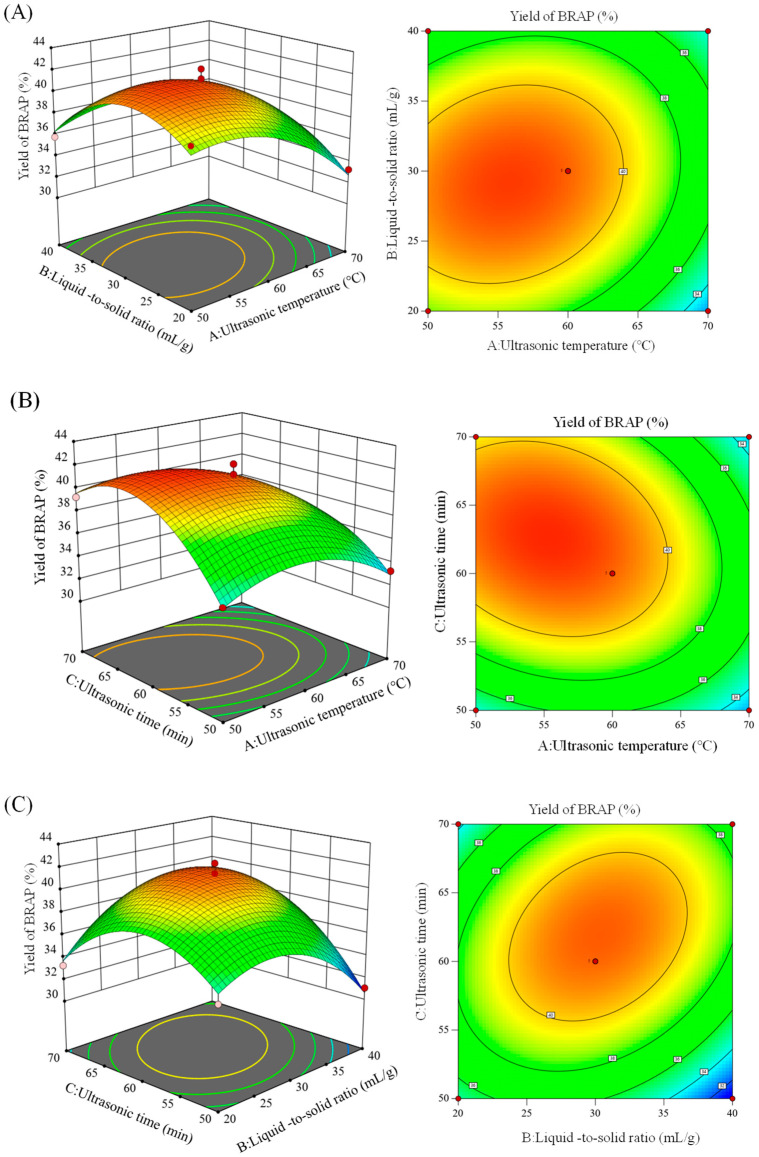
Response surface plots and contour plots (**A**–**C**) showing the effects of ultrasonic temperature, liquid-to-solid ratio, ultrasonic time, and their mutual effects on the extraction yield of BRAP.

Three-dimensional (3D) response surface plots and contour maps were generated using Design-Expert 10, as illustrated in Figure 1. The steepness of the surfaces highlights the strength of interactions between the variables. Figure 1A,C indicates that the 3D response surfaces for BC and AC exhibit pronounced curvature and steepness, with their contour maps displaying distinct elliptical shapes, suggesting significant interaction effects. In contrast, the AB interaction shows less steepness, indicating a non-significant interaction effect. By substituting the optimal conditions into the model equation, the ideal extraction parameters were determined as follows: ultrasonic temperature of 55.18 °C, liquid-to-solid ratio of 29.73 mL/g, ultrasonic time of 62.47 min, and ultrasonic power of 175 W. Under these conditions, the maximum yield of BRAP was predicted to be 41.83%. Considering practical constraints, the actual experimental conditions were adjusted to an ultrasonic power of 175 W, an ultrasonic temperature of 60 °C, a liquid-to-solid ratio of 30 mL/g, and an ultrasonic time of 60 min. The actual maximum yield achieved under these conditions was 41.12%, closely aligning with the predicted value.

### 3.2. Structural Analysis and Chemical Composition of BRAP1-1

#### 3.2.1. Molecular Weight Determination and Monosaccharide Composition

**Figure 2:**
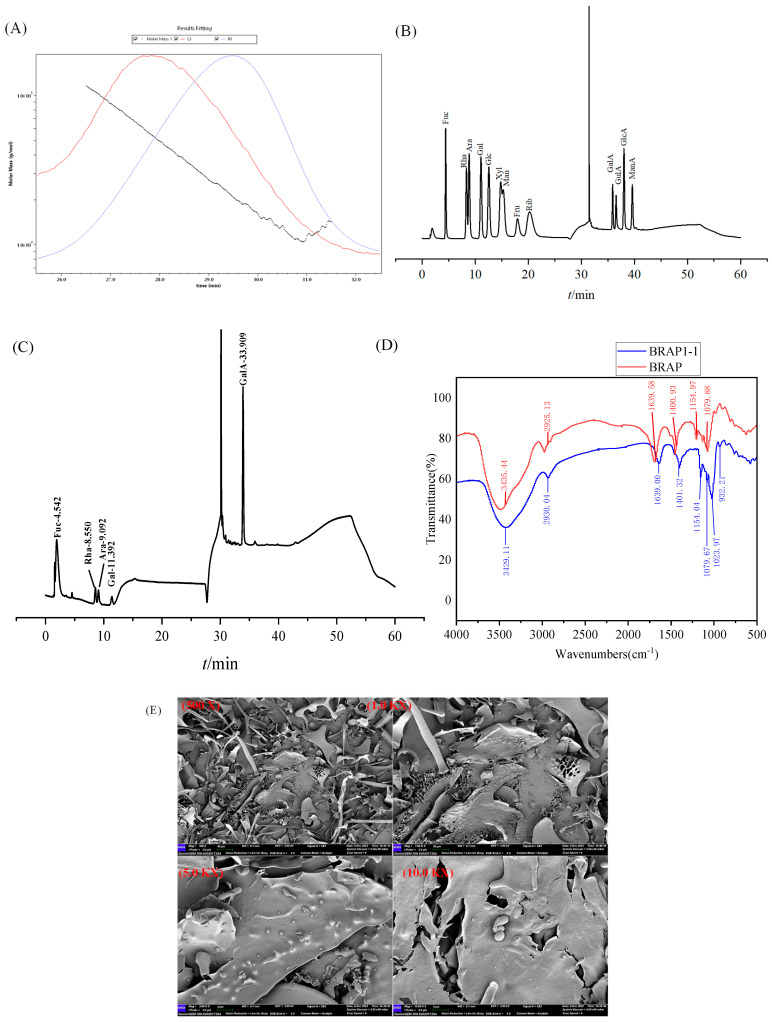
Structural characteristics of BRAP1-1. (**A**) Molecular weight determination of BRAP1-1; (**B**) monosaccharide reference substance mixed solution; (**C**) monosaccharide composition of BRAP1-1; (**D**) FT-IR spectra of BRAP and BRAP1-1; (**E**) SEM images of BRAP1-1.

As shown in Figure 2A, the LS curve represents the fitted data, exhibiting a relatively smooth decreasing trend, indicating a gradual reduction in molar mass over time. The RI profile follows a consistent trend with the LS curve. The molar mass 1 curve displays the variation of molar mass with time, where the data points are more scattered, showing a fluctuating pattern [36]. Over time, the molar mass generally decreases, with a particularly pronounced decline observed between 27.0 and 29.0 min. Overall, the weight-average molecular mass (Mw) of BRAP1-1 was determined to be 32.55 kDa. Prior research has established that extraction methodologies significantly impact polysaccharide molecular weights [37]. Notably, microwave-extracted polysaccharides consistently exhibit lower molecular masses than those obtained via hot water extraction [38]. This difference may be attributed to the intense and rapidly changing mechanical motion of plant polysaccharides induced by the high acceleration of ultrasonic waves, which could lead to the breakage of chemical bonds in their main chains and result in self-degradation [39].

The monosaccharide composition of BRAP1-1 was determined using HPAEC. As shown in Figure 2B, the chromatogram of standard monosaccharides demonstrated complete separation of 13 monosaccharide standards. The retention times (tR) of the standard monosaccharides were as follows: fucose (tR = 4.42 min), rhamnose (tR = 8.37 min), arabinose (tR = 8.86 min), galactose (tR = 11.09 min), glucose (tR = 12.59 min), xylose (tR = 14.86 min), mannose (tR = 15.39 min), fructose (tR = 18.14 min), ribose (tR = 20.46 min), galacturonic acid (tR = 35.18 min), guluronic acid (tR = 35.83 min), glucuronic acid (tR = 37.35 min), and mannuronic acid (tR = 38.96 min). The monosaccharide composition analysis of BRAP1-1, depicted in Figure 2C, was conducted by comparing its retention times with those of the standards (see details in Table S1). BRAP1-1 was identified to consist of fucose, rhamnose, arabinose, galactose, and galacturonic acid, with molar ratios of 0.81: 4.30: 3.61: 1.69: 89.59. The polysaccharide isolated from *Ziziphus jujuba* by Ji et al. [40] was composed of rhamnose, arabinose, galactose, and galacturonic acid, which is consistent with the findings of the present study. Wang et al. [41] characterized polysaccharide MP21 as comprising rhamnose, arabinose, and galactose. These discrepancies in monosaccharide profiles are primarily attributable to variations in raw material sources and analytical detection methodologies.

#### 3.2.2. FT-IR Analysis

FT-IR spectroscopy revealed the main functional groups of the polysaccharides. Figure 2D shows a typical polysaccharide FT-IR spectrum with the typical characteristic features. A broad absorption peak in the range of 3600–3200 cm^−1^ was observed, corresponding to the O–H stretching vibration, characteristic of hydroxyl groups. The absorption peaks at 2930.04 cm^−1^ and 2925.13 cm^−1^ were the result of the C–H stretching vibrations of saturated alkanes. The peaks at 1639.00 cm^−1^ and 1639.58 cm^−1^ were assigned to the C=O double bond stretching of carboxyl groups and the asymmetric stretching vibration of COO^−^. Similarly, the absorption peaks at 1401.32 cm^−1^ and 1400.93 cm^−1^ were associated with the symmetric stretching vibration of COO^−^ and the C–O stretching vibration of COOH [42]. The presence of absorption peaks between 1000 cm^−1^ and 1100 cm^−1^ indicated the existence of pyranose rings [43], with contributions from the C–O–C ether bond and O–H groups. Additionally, the absorption peak at 932 cm^−1^ likely originates from transverse vibrations of methylene groups (—CH_2_—). The presence of β-configuration in BRAP1-1 requires further verification through advanced structural analyses such as X-ray diffractometer (XRD) and NMR spectroscopy.

#### 3.2.3. SEM Analysis

Figure 2E displays the surface morphological characteristics of BRAP1-1 as observed by SEM. BRAP1-1 exhibits a combination of smooth spherical particles, a honeycomb-like porous structure, and rough, flaky sheets. At higher magnification, pores and cracks are visible on the polysaccharide surface, likely resulting from intermolecular repulsion and weak attractive forces between molecules [44]. In contrast, studies have indicated that freeze-dried polysaccharides derived from Sargassum spp. exhibit a uniform structure and a smooth surface morphology [45]. In comparison to the hot water-extracted and freeze-dried polysaccharides, those isolated via ultrasound-assisted extraction display a more porous and irregular structure, suggesting an increased specific surface area. The extraction method significantly influences the morphology of polysaccharides. Ultrasonic treatment disrupts the cross-linking within polysaccharide molecules, leading to noticeably smaller particles and a more porous, looser structure [46]. Similarly, the samples obtained through microwave extraction exhibit greater structural degradation, likely due to the elevated thermal energy inherent to the microwave extraction process [47].

#### 3.2.4. Chemical Composition

The contents of total carbohydrates, protein, uronic acid, and sulfate in the BRAPss (including crude BRAP and purified BRAP1-1) of *Brassica rapa* L. are presented in Table 1. As shown in the table, significant variations were observed in the contents of total carbohydrates and proteins among different fractions. The purified BRAP1-1 fraction showed significantly increased total carbohydrates (73.68 ± 1.81%, *p* < 0.05) and uronic acids (7.93 ± 0.20%, *p* < 0.05) relative to the crude fraction, with a corresponding reduction in protein content (0.48 ± 0.16%). Additionally, the sulfate content of the crude BRAP was reduced to some extent following purification.

### 3.3. Gut Microbiota Analysis

#### 3.3.1. Dilution Curves and Shannon Index Curves

**Figure 3:**
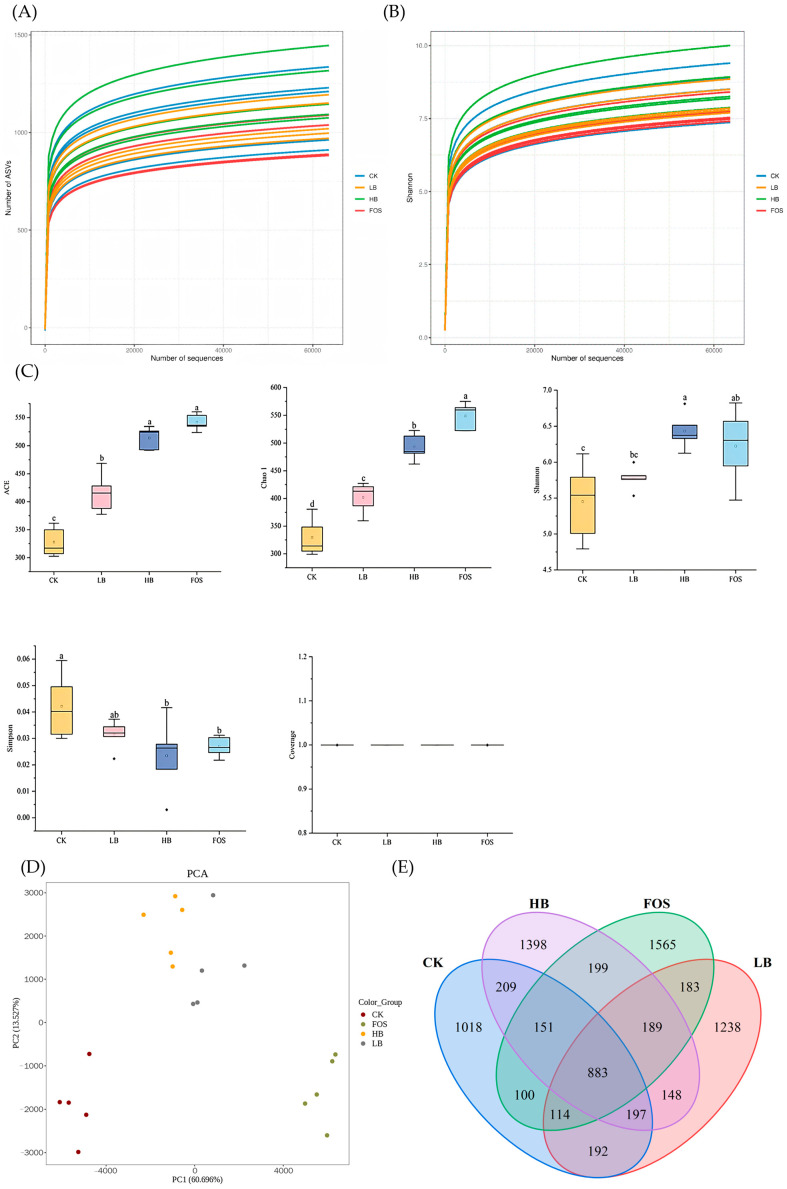
Microbial alpha diversity and beta diversity. (**A**) Dilution curves; (**B**) Shannon index curves; (**C**) box plot of the α-diversity index of the gut microbiota; (**D**) principal component analysis of different groups; (**E**) Venn diagram for different groups. Values were presented as means ± SD (n = 5), different lowercases indicate significant difference (*p* < 0.05) for the different blood samples.

The dilution curves and Shannon index curves are illustrated in Figure 3A,B. The dilution curves reflect community richness, while the Shannon index represents community diversity. Rarefaction curves reflect sampling adequacy and sequencing coverage [48]. Both curves approached a plateau, demonstrating that the sequencing data were saturated and sufficiently comprehensive to capture the majority of species within the intestinal microbiota.

#### 3.3.2. Microbial Alpha Diversity

Alpha diversity reflects species diversity and abundance within samples, with primary metrics including Chao1, Ace, Shannon, and Simpson indices. Specifically, Chao1 and Ace indices measure species richness, while Shannon and Simpson indices collectively evaluate species diversity [49]. As shown in Figure 3C, compared to the CK group, the ACE and Chao1 indices significantly increased in the other three groups (*p* < 0.05), indicating that BRAP1-1 in the experimental groups promotes the growth of specific bacteria, thereby enhancing microbial richness and species abundance [50]. In the LB and HB groups, the Shannon index, ACE index, and Chao1 index increased with the concentration of BRAP1-1 (*p* < 0.05), suggesting that the diversity of gut microbiota exhibits a concentration-dependent effect based on BRAP1-1 dosage. Regarding the Simpson index across experimental groups, the overall trend was CK group > LB group > FOS group > HB group. The comparison between CK and HB/FOS groups yielded significant differences (*p* < 0.05), while no significant differences existed among the LB, HB, and FOS groups (*p* > 0.05). These results demonstrate that varying concentrations of BRAP1-1 exert regulatory effects on the total abundance and structural diversity of gut microbiota.

#### 3.3.3. Microbial Beta Diversity

Beta diversity analysis is employed in microbial community studies to assess the similarity or dissimilarity of microbial compositions between different samples. Principal Component Analysis (PCA) [51], based on Euclidean distance, is commonly employed for data dimensionality reduction while preserving the features that contribute most to variance in the dataset, thereby effectively identifying the most “significant” elements and structures. Venn diagrams [52] enable the screening of unique OTUs within each sample group and shared OTUs among groups, with the visual representation of these relationships. An operational taxonomic unit (OTU) is defined when sequence similarity within a sample exceeds 97%. Each OTU represents a distinct sequence, and clustering sequences into OTUs facilitates experimental analysis. The number of OTUs in a sample directly correlates with microbial species richness. As shown in the PCA analysis in Figure 3D, significant differences in microbial communities were observed between the CK group and the other groups, indicating that the intake of BRAP can induce alterations in the gut microbiota of mice. The clustering of PCA data for the LB and HB groups suggests a similarity in microbial composition [49]. The principal components PC1 and PC2 accounted for 69.70% and 13.53% of the variance, respectively. Overall, the microbial community structures among the groups exhibited considerable differences, indicating that the composition underwent certain changes, resulting in a more complex community structure. As illustrated in Figure 3E, Venn analysis at the OTU level revealed that 883 OTUs were shared among the four groups. The groups retained 1018, 1238, 1398, and 1565 OTUs, respectively, with the order being CK group < LB group < HB group < FOS group, all showing an increase compared to the CK group. The trend of these results is consistent with the alpha diversity analysis, suggesting that the addition of BRAP1-1 has a certain regulatory effect on the diversity of the gut microbiota.

#### 3.3.4. Modulation of the Gut Microbiota Structure by BRAP1-1

**Figure 4:**
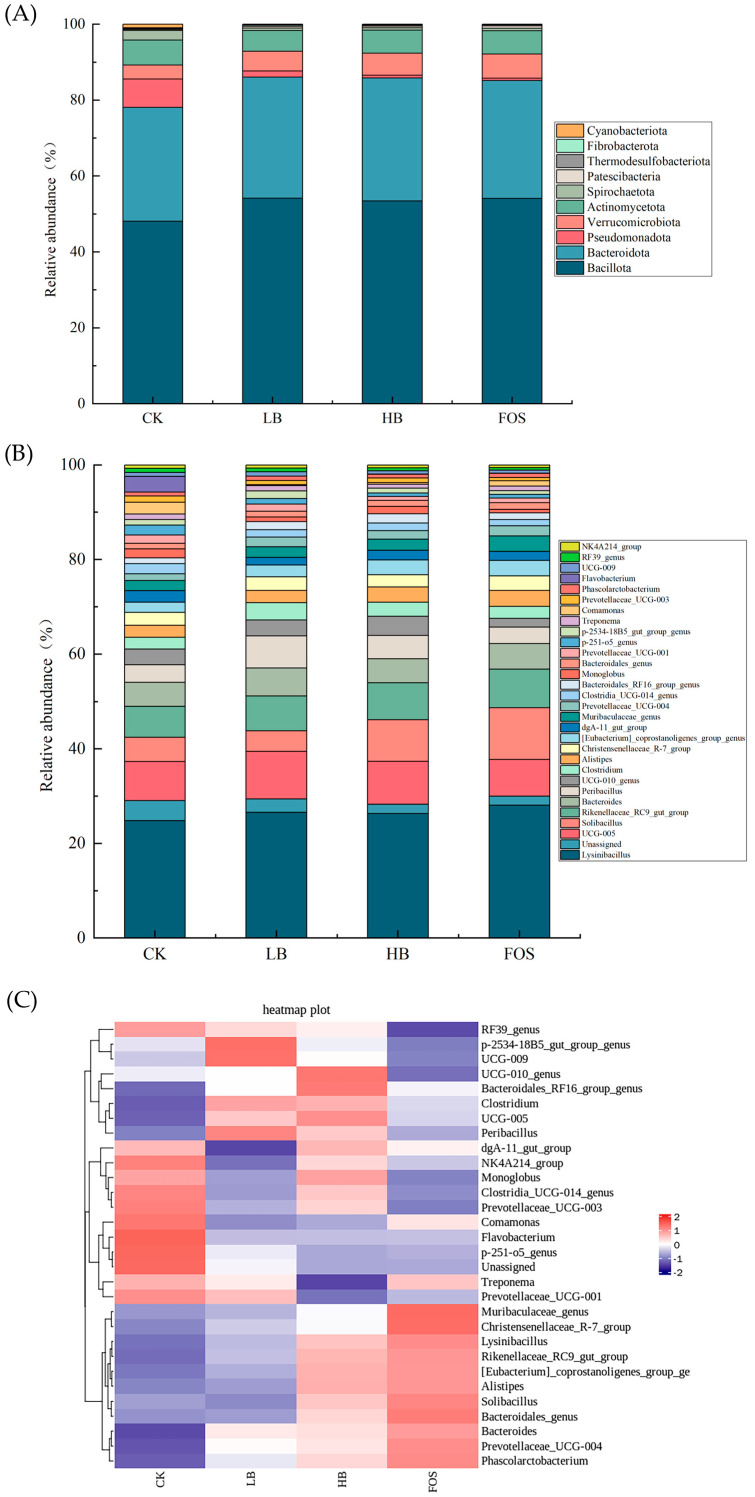

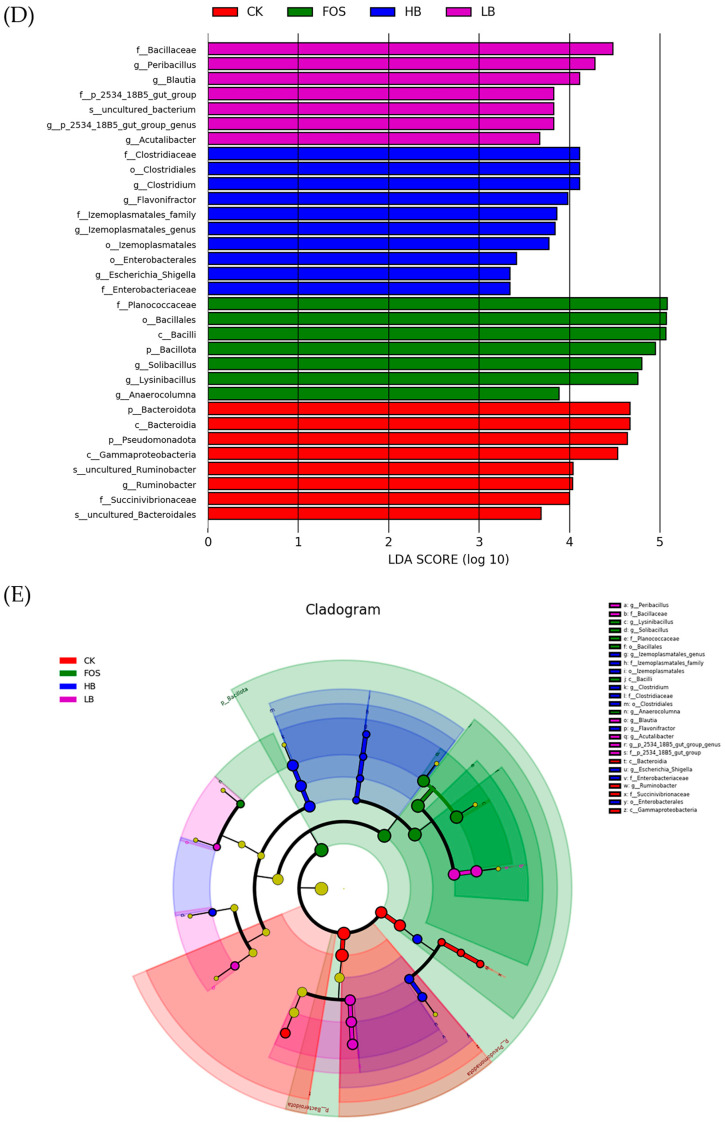
Microbial composition at phylum (**A**), genus level (**B**), changes in the most abundant microbiota at the genus level (**C**), and LEfSe analysis of the gut microbiota (**D**,**E**).

To characterize changes in gut microbial community structure, the composition of the gut microbiota at different taxonomic levels was analyzed. As shown in Figure 4A, at the phylum level, the gut microbiota in the control group (CK) was dominated by five major phyla: *Bacillota*, *Bacteroidota*, *Pseudomonadota*, *Verrucomicrobiota*, and *Actinomycetota*. However, *Pseudomonadota* was almost absent in the other three groups. Relevant studies have indicated that certain species within *Pseudomonadota* (formerly *Proteobacteria*), particularly *Pseudomonas aeruginosa*, may have pathogenic effects on human health, especially under immunosuppressed conditions or in the presence of wounds, where they are more likely to cause infections [53]. Relative to the control (CK) group, Bacillota abundance was elevated across all treatment cohorts (LB, HB, and FOS).

For genus-level analysis, the 30 most dominant species were prioritized for investigation, as shown in Figure 4B,C. The identified microorganisms at the genus level mainly included *Lysinibacillus*, *UCG-005*, *Solibacillus*, *Rikenellaceae_RC9_gut_group*, *Bacteroides*, *Alistipes*, *Christensenellaceae_R-7_group*, and *[Eubacterium]_coprostanoligenes_group_genus*, among others. Among these, *Lysinibacillus* and *UCG-005* had the highest proportions, with average relative abundances of 26.45% and 8.80%, respectively. Compared to the CK group, the abundances of *Lysinibacillus*, *Solibacillus*, *Rikenellaceae_RC9_gut_group*, *Bacteroides*, and *Alistipes* increased in the groups treated with different concentrations of BRAP1-1. In contrast, the abundances of *dgA-11_gut_group*, *Monoglobus*, *p-251-o5_genus*, *Treponema*, *Comamonas*, and *Flavobacterium* decreased. *The Rikenellaceae_RC9_gut_group* is an important gut bacterial group that may play a significant role in maintaining gut microbiota diversity, disease associations, and microbial interactions. For instance, Zhang et al. [54] found an increase in the relative abundance of *Rikenellaceae_RC9_gut_group* in the gut microbiota of mice following intervention with Sophora flavescens alcohol extract in a study on gastric cancer inhibition and gut microbiota modulation. Another important gut genus, *Alistipes*, has both beneficial and detrimental effects on the host. For example, *A. finegoldii* administration demonstrated marked efficacy in symptom amelioration within the DSS-induced colitis murine model [55], but it may also promote right-sided colon cancer through the IL-6/STAT3 pathway [56]. However, the study identified a subtle increase in Alistipes prevalence within the intestinal microbiome of mice receiving differential BRAP1-1 treatments, but overall remained balanced. As shown in Figure 4C, the genus *Flavobacterium* accounted for 3.34% of the CK group but was nearly absent in the other three groups. *Flavobacterium*, as a potential gut pathogen, may disrupt the structure of the gut microbiota when overrepresented, resulting in a depletion of beneficial bacteria and a proliferation of harmful bacteria [57].

As shown in Figure 4D,E, a total of 32 highly abundant differential taxa were identified across the four groups. In the CK group, the key differential taxa included *Bacteroidia*, *Ruminobacter*, *Succinivibrionaceae*, and *Gammaproteobacteria*. In the LB group, the primary differential taxa were *Blautia*, *Acutalibacter*, *Peribacillus*, and *Bacillaceae*. For the HB group, the significant differential taxa were *Izemoplasmatales genus*, *Clostridium*, *Flavonifractor*, *Escherichia_Shigella*, and *Enterobacteriaceae*. In the FOS group, the dominant differential taxa were *Lysinibacillus*, *Solibacillus*, *Planococcaceae*, *Bacillales*, and *Anaerocolumna*. In summary, each group exhibited distinct differential microbial communities, indicating that different concentrations of BRAP1-1 exert selective effects on the gut microbiota. By comparing the structural differences in microbial communities among the groups, it can be inferred that specific microbial taxa have a strong capacity to degrade and utilize BRAP1-1 at particular concentrations [58].

### 3.4. Effect of BRAP1-1 on SCFAs in Mice Feces

**Figure 5:**
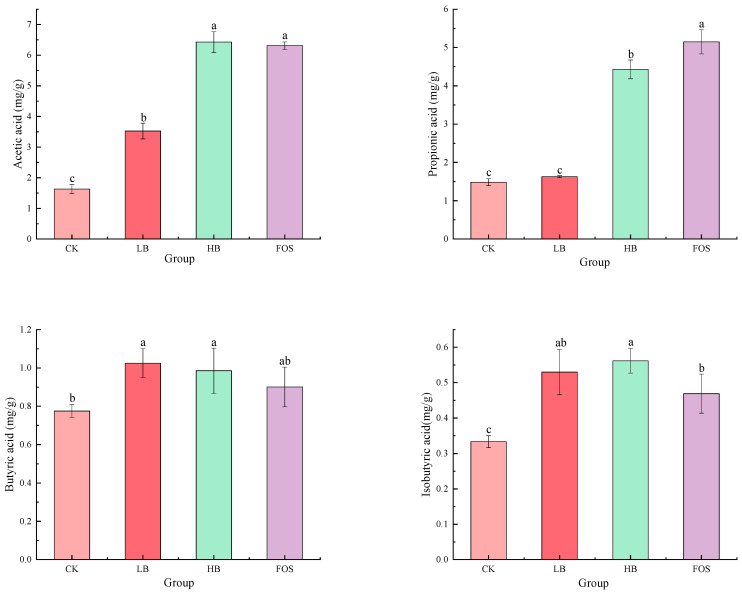

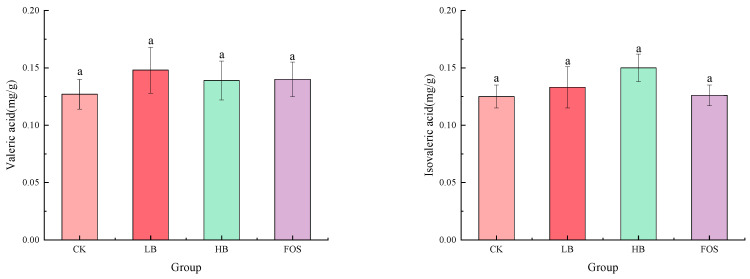
Changes in the content of SCFAs in the feces of different groups of mice (mg/g). Values were presented as means ± SD (n = 5), and different lowercase letters indicate significant differences (*p* < 0.05) for the different blood samples.

Specialized intestinal microbial communities enzymatically convert dietary polysaccharides into short-chain fatty acids (SCFAs)—primarily acetate, propionate, and butyrate—via anaerobic fermentation pathways [25]. It is difficult to quantitatively analyze them using mass spectrometry in experiments to confirm the production of SCFAs because they have poor ionization efficiency and susceptibility to water loss during ionization of low molecular weight organic acids [59]. Therefore, SCFAs were identified and quantified by UHPLC-MS according to the method described by Song et al. [60], with chromatographic retention time matching against authentic standards (acetate, propionate, butyrate, etc.) and multiple reaction monitoring (MRM) of characteristic ions for precise quantification. As shown in Figure 5, compared to the CK group, the levels of acetate, butyrate, and isobutyrate were significantly increased in the LB and HB groups (*p* < 0.05). In the FOS group, the levels of acetate, propionate, and isobutyrate were significantly elevated (*p* < 0.05). Compared to the FOS group, the acetate and propionate levels were significantly lower in the LB group (*p* < 0.05), while the propionate level was significantly lower in the HB group (*p* < 0.05); concurrently, isobutyrate concentrations demonstrated a marked elevation (*p* < 0.05) compared to baseline measurements. Valerate and isovalerate concentrations across experimental cohorts demonstrated elevated levels relative to controls, though without reaching statistical significance (*p* > 0.05). These results indicate that BRAP1-1, at specific concentrations, can effectively promote the production of SCFAs, thereby improving the intestinal environment [61].

### 3.5. Relationship Between SCFAs and Microbiota

**Figure 6:**
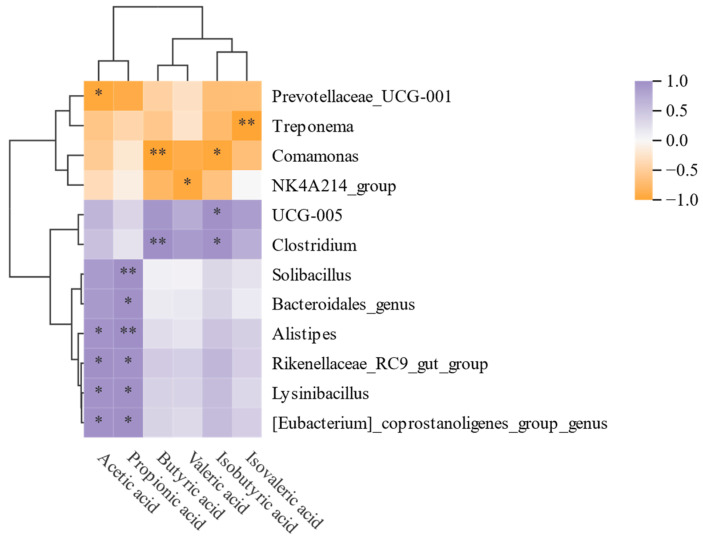
Analysis of the correlation between SCFAs and gut microbiota (*: significant correlation (*p* < 0.05), **: highly significant correlation (*p* < 0.01)).

To explore the connections between the gut microbiota and its metabolic SCFAs, we represented the findings using a clustering heatmap (Figure 6). Notably, the content of isovaleric acid exhibited an inverse trend in comparison to the remaining SCFAs. Additionally, six genera demonstrated a significant correlation with both acetic acid and propionic acid, including *[Eubacterium]_coprostanoligenes_group_genus* (*p* < 0.05) and *Bacteroidales_genus* (*p* < 0.05). These genera have been previously reported to regulate SCFA synthesis [62], which is consistent with the results of this study. Both butyric acid and isobutyric acid were negatively regulated by *Comamonas*, while acetic acid and isovaleric acid were negatively regulated by *Prevotellaceae_UCG-001* and *Treponema*, respectively. The findings indicated that BRAP1-1 supplementation strengthened the relationship between microbiota and beneficial metabolites.

## 4. Discussion

This study employed ultrasound-assisted extraction (UAE) to optimize the polysaccharide isolation process from *Brassica rapa* L., demonstrating it as an efficient and eco-friendly technology that overcomes the limitations of traditional methods (e.g., hot water extraction), including prolonged processing time, high temperatures, and low yields [63]. Through systematic optimization, the peak extraction parameters for BRAP emerged as: ultrasonic power 175 W, temperature 60 °C, liquid-to-solid ratios 30 mL/g, and extraction time 60 min. Wang et al. [22] extracted *Brassica rapa* L. polysaccharides using conventional hot water extraction under optimized conditions (extraction temperature 93 °C, duration 4.3 h, liquid-to-solid ratios 75 mL/g, achieving a yield of 21.48 ± 0.41%. In comparison with our experimental results, the ultrasound-assisted extraction method significantly reduced both processing time and temperature while simultaneously improving polysaccharide yield. In addition to UAE, emerging “green” extraction technologies, such as freeze-thaw cold pressing, pressurized water extraction, repeated freeze-thaw cycles, and ultrasound-ultralow temperature synergistic methods, have been developed. For instance, He et al. [64] extracted Dendrobium polysaccharides via cold pressing and freeze–thaw methods, while He et al. [65] applied pressurized water extraction to isolate highland barley polysaccharides. These techniques could be further explored for BRAP extraction.

The precise chemical structures of plant polysaccharides remain a focal yet challenging research area in scientific investigations. Elucidating their structural features is critical for advancing their applications [66]. In this study, Monosaccharide composition and molecular weight analyses demonstrated that the BRAP1-1 consists of fucose, rhamnose, arabinose, galactose, and galacturonic acid in a molar ratio of 0.81:4.30:3.61:1.69:89.59, with a molecular weight of 32.55 kDa. Structural characterization indicated smooth spherical particles, honeycomb-like porous structures, and rough lamellar morphologies. However, the methods employed here only provided preliminary insights into BRAP1-1’s primary structure. Recent studies have achieved deeper structural elucidation. Structural characterization of jujube polysaccharides was performed by Ji et al. [67] using HPGPC, GC, and NMR techniques. The results demonstrated that the jujube polysaccharides had a molecular weight of 27.90 kDa and exhibited a netted structure with molecular aggregates. The backbone was primarily composed of (1→4)-linked Gal*p*A residues, with three branched chains attached at the O-3 position, consisting of (1→3)-linked Ara*f*, (1→2)-linked Rha*p*, and terminal Gal*p*A units.

Polysaccharides serve as energy substrates for gut microbiota, promoting beneficial bacterial growth and modulating microbial composition through metabolic byproducts, thereby altering intestinal ecological diversity [68]. Shao et al. [69] found that *Hericium erinaceus mycelium* polysaccharides significantly increased the diversity and abundance of gut microbiota, restored the populations of *Verrucomicrobia*, *Firmicutes*, *Bacteroidetes*, and *Proteobacteria* while elevating the concentrations of acetic acid and butyric acid in intestinal contents, thereby enhancing SCFAs production. In healthy human gut microbiota, *Firmicutes* and *Bacteroidetes* dominate (>90%), with minor proportions of *Proteobacteria* and *Actinobacteria* [70]. Increased *Proteobacteria* abundance is recognized as a marker of dysbiosis, while *Bacteroidetes* levels correlate positively with pro-inflammatory factors [71]. In this study, 16S rDNA sequencing of fecal samples from humanized mice treated with BRAP1-1 showed elevated relative abundances of beneficial genera (*Lysinibacillus*, *Rikenellaceae_RC9_gut_group*, *Bacteroides*, *Alistipes*) and reduced pathogenic genera (*dgA-11_gut_group*, *Monoglobus*, *p-251-o5_genus*, *Flavobacterium*) compared to the CK group. BRAP1-1 supplementation significantly increased fecal SCFAs (acetic, propionic, butyric, and isobutyric acids; *p* < 0.05). Loo et al. [72] found that sugarcane polyphenols promoted the growth and proliferation of beneficial bacteria, increased total SCFA production, particularly propionate and butyrate, and improved host intestinal health. These findings are consistent with our results. Correlation analysis identified *Alistipes*, *Rikenellaceae_RC9_gut_group*, *Lysinibacillus*, and *[Eubacterium]_coprostanoligenes_group* as key contributors to acetic/propionic acid synthesis. Butyric and isobutyric acids were negatively regulated by *Comamonas*, while acetic and isovaleric acids were suppressed by *Prevotellaceae_UCG-001* and *Treponema*, respectively. These findings indicate that BRAP1-1 modulates gut microbiota to enhance SCFA metabolism, thereby maintaining intestinal homeostasis.

## 5. Conclusions

Ultrasonic-assisted extraction has developed into an efficient technique for enhancing the extraction efficiency of polysaccharides. In this study, we investigated the yield of polysaccharides from *Brassica rapa* L. (BRAP) under ultrasonic-assisted extraction and optimized its extraction process using response surface methodology software. The extraction results demonstrated that ultrasound-assisted extraction significantly improved the yield of BRAP compared to conventional hot water extraction, achieving a yield of 41.12% under optimal conditions (ultrasonic temperature 60 °C, 30 mL/g liquid–solid ratios, ultrasonic time 60 min, and power 175 W). Based on this, we further performed structural identification of the purified polysaccharides (BRAP1-1). Structural analysis revealed that BRAP1-1 had a molecular weight of 32.55 kDa, with a monosaccharide composition primarily consisting of fucose, rhamnose, arabinose, galactose, and galacturonic acid in a molar ratio of 0.81:4.30:3.61:1.69:89.59. Additionally, BRAP1-1 exhibited characteristic polysaccharide absorption peaks and displayed a smooth spherical morphology with a porous honeycomb-like and rough lamellar structure.

Gavage experiments in BRAP1-1’s humanized mouse model have demonstrated that Brassica rapa L. polysaccharides can effectively regulate human gut microbiota through increasing short-chain fatty acid production and promoting the growth of beneficial bacteria such as *Lysinibacillus*, *Rikenellaceae_RC9_gut_group*, and *Bacteroides*, while inhibiting harmful bacteria, like *Flavobacterium*. These findings indicate that *Brassica rapa* L. polysaccharides enhance gut microbial metabolic activity, increase bacterial diversity, and stimulate the proliferation of beneficial gut bacteria. Collectively, this research establishes a theoretical framework for BRAP1-1’s application in functional foods and offers novel insights into the development of microbiota-regulating natural products.

## Figures and Tables

**Table 1:** Chemical composition analysis of BRAP and BRAP1-1.

Item	Carbohydrate (%)	Protein (%)	Uronic Acid (%)	Sulfuric Radical (%)
BRAP	38.17 ± 1.67 ^b^	1.86 ± 0.23 ^a^	11.32 ± 1.38 ^b^	1.25 ± 0.11 ^a^
BRAP1-1	73.68 ± 1.81 ^a^	0.48 ± 0.16 ^b^	26.11 ± 1.25 ^a^	0.56 ± 0.12 ^b^

Different lowercase letters in the table denote statistically significant differences (*p* < 0.05) in chemical composition among the various fractions.

## Data Availability

The original contributions presented in this study are included in the article/supplementary materials. Further inquiries can be directed to the corresponding author.

## Supplementary Materials

The following supporting information can be downloaded at: https://www.mdpi.com/article/10.3390/foods14111994/s1, Table S1: Chromatographic retention time and signal intensity of BRAP1-1.
