# Supplementary material for: Ultrasonic-Assisted Extraction of Polysaccharides from Brassica rapa L. and Its Effects on Gut Microbiota in Humanized Mice
Source: Foods. 2025 Jun 5;14(11):1994. doi: 10.3390/foods14111994 (PMC12154061; doi:10.3390/foods14111994)
Supplement: Supplementary file 1 [file foods-14-01994-s001.zip › foods-3603470-supplementary.pdf]

Table S1. Chromatographic retention time and signal intensity of BRAP1-1.

time	Signal intensity (nC)		
	①	②	③
0.000333	0.03565	0.03568	0.03564
0.00867	0.0362	0.03622	0.03619
0.017	0.0405	0.04052	0.04049
0.02533	0.04845	0.04846	0.04844
0.03367	0.06155	0.06156	0.06154
0.042	0.06995	0.06996	0.06994
0.05033	0.06855	0.06856	0.06854
0.05867	0.065	0.06502	0.06499
0.067	0.0564	0.05641	0.05639
0.07533	0.04675	0.04676	0.04674
0.08367	0.03515	0.03516	0.03514
0.092	0.0164	0.01641	0.01639
0.10033	-0.0117	-0.01169	-0.01171
0.10867	-0.0379	-0.03789	-0.03791
0.117	-0.0642	-0.06417	-0.06421
0.12533	-0.0788	-0.07878	-0.07881
0.13367	-0.07845	-0.07844	-0.07846
0.142	-0.07225	-0.07224	-0.07226
0.15033	-0.0706	-0.07059	-0.07061
0.15867	-0.0668	-0.06679	-0.06681
0.167	-0.06225	-0.06224	-0.06226
0.17533	-0.05845	-0.05844	-0.05846
0.18367	-0.05665	-0.05664	-0.05666
0.192	-0.05315	-0.05313	-0.05316
0.20033	-0.04855	-0.04852	-0.04856
0.20867	-0.04145	-0.04144	-0.04146
0.217	-0.0348	-0.03477	-0.03481
0.22533	-0.0301	-0.03009	-0.03011
0.23367	-0.0271	-0.02709	-0.02711
0.242	-0.02585	-0.02584	-0.02586
0.25033	-0.02585	-0.02584	-0.02586
0.25867	-0.0325	-0.03249	-0.03251
0.267	-0.0431	-0.04308	-0.04311
0.27533	-0.05625	-0.05624	-0.05626
0.28367	-0.08005	-0.08003	-0.08006
0.292	-0.10445	-0.10444	-0.10446
0.30033	-0.12965	-0.12963	-0.12966
0.30867	-0.1507	-0.15069	-0.15071
0.317	-0.15545	-0.15544	-0.15546
0.32533	-0.1494	-0.14939	-0.14941

0.33367	-0.144	-0.14398	-0.14401
0.342	-0.1405	-0.14049	-0.14051
0.35033	-0.13595	-0.13594	-0.13596
0.35867	-0.13205	-0.13204	-0.13206
0.367	-0.1293	-0.12929	-0.12931
0.37533	-0.1254	-0.12539	-0.12541
0.38367	-0.1183	-0.11828	-0.11831
0.392	-0.11185	-0.11184	-0.11186
0.40033	-0.1059	-0.10589	-0.10591
0.40867	-0.097	-0.09699	-0.09701
0.417	-0.08805	-0.08804	-0.08806
0.42533	-0.0904	-0.09039	-0.09041
0.43367	-0.0906	-0.09058	-0.09061
0.442	-0.09495	-0.09494	-0.09496
0.45033	-0.1039	-0.10389	-0.10391
0.45867	-0.11405	-0.11404	-0.11406
0.467	-0.13275	-0.13274	-0.13276
0.47533	-0.15925	-0.15924	-0.15926
0.48367	-0.18425	-0.18423	-0.18426
0.492	-0.209	-0.20899	-0.20901
0.50033	-0.2204	-0.22039	-0.22041
0.50867	-0.2161	-0.21609	-0.21611
0.517	-0.20825	-0.20824	-0.20826
0.52533	-0.20495	-0.20493	-0.20496
0.53367	-0.2013	-0.20129	-0.20131
0.542	-0.19605	-0.19604	-0.19606
0.55033	-0.1909	-0.19089	-0.19091
0.55867	-0.1819	-0.18189	-0.18191
0.567	-0.1756	-0.17559	-0.17561
0.57533	-0.17255	-0.17254	-0.17256
0.58367	-0.16325	-0.16324	-0.16326
0.592	-0.1536	-0.15359	-0.15361
0.60033	-0.14775	-0.14774	-0.14776
0.60867	-0.14725	-0.14724	-0.14726
0.617	-0.1463	-0.14629	-0.14631
0.62533	-0.14645	-0.14644	-0.14646
0.63367	-0.15425	-0.15424	-0.15426
0.642	-0.1649	-0.16489	-0.16491
0.65033	-0.1797	-0.17969	-0.17971
0.65867	-0.206	-0.20599	-0.20601
0.667	-0.23135	-0.23134	-0.23136
0.67533	-0.2552	-0.25519	-0.25521
0.68367	-0.2751	-0.27509	-0.27511
0.692	-0.27515	-0.27514	-0.27516

0.70033	-0.26935	-0.26934	-0.26936
0.70867	-0.2641	-0.26409	-0.26411
0.717	-0.25985	-0.25984	-0.25986
0.72533	-0.2549	-0.25489	-0.25491
0.73367	-0.24875	-0.24874	-0.24876
0.742	-0.24525	-0.24524	-0.24526
0.75033	-0.2416	-0.24159	-0.24161
0.75867	-0.2343	-0.23429	-0.23431
0.767	-0.2259	-0.22589	-0.22591
0.77533	-0.22075	-0.22074	-0.22076
0.78367	-0.21335	-0.21334	-0.21336
0.792	-0.20605	-0.20604	-0.20606
0.80033	-0.2083	-0.20829	-0.20831
0.80867	-0.2065	-0.20649	-0.20651
0.817	-0.21095	-0.21094	-0.21096
0.82533	-0.2202	-0.22019	-0.22021
0.83367	-0.232	-0.23199	-0.23201
0.842	-0.2533	-0.25329	-0.25331
0.85033	-0.27945	-0.27944	-0.27946
0.85867	-0.3036	-0.30359	-0.30361
0.867	-0.32565	-0.32564	-0.32566
0.87533	-0.33415	-0.33414	-0.33416
0.88367	-0.3268	-0.32679	-0.32681
0.892	-0.31945	-0.31944	-0.31946
0.90033	-0.31475	-0.31474	-0.31476
0.90867	-0.3113	-0.31129	-0.31131
0.917	-0.30565	-0.30564	-0.30566
0.92533	-0.30285	-0.30284	-0.30286
0.93367	-0.29985	-0.29984	-0.29986
0.942	-0.29295	-0.29294	-0.29296
0.95033	-0.28695	-0.28694	-0.28696
0.95867	-0.2788	-0.27879	-0.27881
0.967	-0.26905	-0.26904	-0.26906
0.97533	-0.2626	-0.26259	-0.26261
0.98367	-0.2602	-0.26019	-0.26021
0.992	-0.25675	-0.25674	-0.25676
1.00033	-0.2594	-0.25939	-0.25941
1.00867	-0.2674	-0.26739	-0.26741
1.017	-0.27735	-0.27734	-0.27736
1.02533	-0.295	-0.29499	-0.29501
1.03367	-0.32185	-0.32184	-0.32186
1.042	-0.3464	-0.34639	-0.34641
1.05033	-0.37	-0.36999	-0.37001
1.05867	-0.3849	-0.38489	-0.38491

1.067	-0.38185	-0.38184	-0.38186
1.07533	-0.3742	-0.37419	-0.37421
1.08367	-0.3685	-0.36849	-0.36851
1.092	-0.3637	-0.36369	-0.36371
1.10033	-0.35965	-0.35964	-0.35966
1.10867	-0.35275	-0.35274	-0.35276
1.117	-0.3538	-0.35379	-0.35381
1.12533	-0.3514	-0.35139	-0.35141
1.13367	-0.34295	-0.34294	-0.34296
1.142	-0.3343	-0.33429	-0.33431
1.15033	-0.32545	-0.32544	-0.32546
1.15867	-0.31725	-0.31724	-0.31726
1.167	-0.31185	-0.31184	-0.31186
1.17533	-0.31025	-0.31024	-0.31026
1.18367	-0.30945	-0.30944	-0.30946
1.192	-0.31445	-0.31444	-0.31446
1.20033	-0.32255	-0.32254	-0.32256
1.20867	-0.3339	-0.33389	-0.33391
1.217	-0.35905	-0.35904	-0.35906
1.22533	-0.3829	-0.38289	-0.38291
1.23367	-0.4076	-0.40759	-0.40761
1.242	-0.4268	-0.42679	-0.42681
1.25033	-0.4316	-0.43159	-0.43161
1.25867	-0.4233	-0.42329	-0.42331
1.267	-0.41845	-0.41844	-0.41846
1.27533	-0.41815	-0.41814	-0.41816
1.28367	-0.4236	-0.42359	-0.42361
1.292	-0.43	-0.42999	-0.43001
1.30033	-0.4195	-0.41949	-0.41951
1.30867	-0.3652	-0.36519	-0.36521
1.317	-0.26135	-0.26134	-0.26136
1.32533	-0.13625	-0.13624	-0.13626
1.33367	-0.03125	-0.03124	-0.03126
1.342	0.03115	0.03116	0.03114
1.35033	0.05435	0.05436	0.05434
1.35867	0.0463	0.04631	0.04629
1.367	0.02905	0.02906	0.02904
1.37533	0.0044	0.00441	0.00439
1.38367	-0.02225	-0.02224	-0.02226
1.392	-0.0466	-0.04659	-0.04661
1.40033	-0.07665	-0.07664	-0.07666
1.40867	-0.10845	-0.10844	-0.10846
1.417	-0.1335	-0.13349	-0.13351
1.42533	-0.1535	-0.15349	-0.15351

1.43367	-0.15835	-0.15834	-0.15836
1.442	-0.149	-0.14899	-0.14901
1.45033	-0.14305	-0.14304	-0.14306
1.45867	-0.1451	-0.14509	-0.14511
1.467	-0.15025	-0.15024	-0.15026
1.47533	-0.15475	-0.15474	-0.15476
1.48367	-0.15575	-0.15574	-0.15576
1.492	-0.1434	-0.14339	-0.14341
1.50033	-0.0847	-0.08469	-0.08471
1.50867	0.09805	0.09806	0.09804
1.517	0.6045	0.60451	0.60449
1.52533	1.6877	1.68771	1.68769
1.53367	3.39305	3.39306	3.39304
1.542	5.3737	5.37371	5.37369
1.55033	7.05015	7.05016	7.05014
1.55867	7.9781	7.97811	7.97809
1.567	8.0806	8.08061	8.08059
1.57533	7.57405	7.57406	7.57404
1.58367	6.7572	6.75721	6.75719
1.592	5.8712	5.87121	5.87119
1.60033	5.06835	5.06836	5.06834
1.60867	4.41695	4.41696	4.41694
1.617	3.9377	3.93771	3.93769
1.62533	3.63585	3.63586	3.63584
1.63367	3.48135	3.48136	3.48134
1.642	3.44165	3.44166	3.44164
1.65033	3.5006	3.50061	3.50059
1.65867	3.6459	3.64591	3.64589
1.667	3.8657	3.86571	3.86569
1.67533	4.1489	4.14891	4.14889
1.68367	4.4839	4.48391	4.48389
1.692	4.86285	4.86286	4.86284
1.70033	5.28195	5.28197	5.28194
1.70867	5.7298	5.72981	5.72979
1.717	6.1998	6.19981	6.19979
1.72533	6.68585	6.68588	6.68584
1.73367	7.17515	7.17516	7.17514
1.742	7.67075	7.67076	7.67074
1.75033	8.15965	8.15966	8.15964
1.75867	8.63845	8.63846	8.63844
1.767	9.10545	9.10546	9.10544
1.77533	9.55075	9.55076	9.55074
1.78367	9.97475	9.97476	9.97474
1.792	10.38365	10.38366	10.38364

1.80033	10.77235	10.77236	10.77234
1.80867	11.1523	11.15231	11.15229
1.817	11.51745	11.51746	11.51744
1.82533	11.8549	11.85491	11.85489
1.83367	12.1606	12.16061	12.16059
1.842	12.43495	12.43497	12.43494
1.85033	12.68125	12.68126	12.68124
1.85867	12.89565	12.89566	12.89564
1.867	13.08235	13.08236	13.08234
1.87533	13.2436	13.24361	13.24359
1.88367	13.37385	13.37387	13.37384
1.892	13.47965	13.47967	13.47964
1.90033	13.56435	13.56437	13.56434
1.90867	13.6202	13.62022	13.62019
1.917	13.64675	13.64677	13.64674
1.92533	13.65095	13.65097	13.65094
1.93367	13.626	13.62602	13.62599
1.942	13.57735	13.57737	13.57734
1.95033	13.50595	13.50597	13.50594
1.95867	13.41185	13.41187	13.41184
1.967	13.2884	13.28842	13.28839
1.97533	13.1537	13.15372	13.15369
1.98367	13.01055	13.01057	13.01054
1.992	12.8686	12.86862	12.86859
2.00033	12.73905	12.73906	12.73904
2.00867	12.60225	12.60226	12.60224
2.017	12.45325	12.45326	12.45324
2.02533	12.289	12.28901	12.28899
2.03367	12.1126	12.11261	12.11259
2.042	11.92285	11.92286	11.92284
2.05033	11.72	11.72001	11.71999
2.05867	11.50875	11.50876	11.50874
2.067	11.2956	11.29563	11.29559
2.07533	11.08285	11.08288	11.08284
2.08367	10.8648	10.86483	10.86479
2.092	10.64355	10.64358	10.64354
2.10033	10.4174	10.41743	10.41739
2.10867	10.1892	10.18923	10.18919
2.117	9.95915	9.95918	9.95914
2.12533	9.72115	9.72118	9.72114
2.13367	9.4807	9.48073	9.48069
2.142	9.23515	9.23518	9.23514
2.15033	8.9778	8.97783	8.97779
2.15867	8.71595	8.71598	8.71594

2.167	8.45495	8.45498	8.45494
2.17533	8.2027	8.20273	8.20269
2.18367	7.9684	7.96843	7.96839
2.192	7.7518	7.75183	7.75179
2.20033	7.541	7.54102	7.54099
2.20867	7.33235	7.33237	7.33234
2.217	7.126	7.12602	7.12599
2.22533	6.9271	6.92712	6.92709
2.23367	6.7257	6.72572	6.72569
2.242	6.5297	6.52972	6.52969
2.25033	6.3456	6.34562	6.34559
2.25867	6.1691	6.16912	6.16909
2.267	6.006	6.00602	6.00599
2.27533	5.8548	5.85482	5.85479
2.28367	5.7146	5.71462	5.71459
2.292	5.5761	5.57612	5.57609
2.30033	5.44585	5.44587	5.44584
2.30867	5.30825	5.30827	5.30824
2.317	5.15905	5.15907	5.15904
2.32533	5.00035	5.00037	5.00034
2.33367	4.82015	4.82017	4.82014
2.342	4.62705	4.62707	4.62704
2.35033	4.4322	4.43222	4.43219
2.35867	4.2394	4.23942	4.23939
2.367	4.0624	4.06242	4.06239
2.37533	3.90915	3.90917	3.90914
2.38367	3.7658	3.76582	3.76579
2.392	3.6235	3.62352	3.62349
2.40033	3.4881	3.48812	3.48809
2.40867	3.36	3.36002	3.35999
2.417	3.23675	3.23677	3.23674
2.42533	3.1138	3.11382	3.11379
2.43367	3.00225	3.00227	3.00224
2.442	2.8966	2.89662	2.89659
2.45033	2.7939	2.79392	2.79389
2.45867	2.69915	2.69917	2.69914
2.467	2.6108	2.61082	2.61079
2.47533	2.51435	2.51437	2.51434
2.48367	2.42045	2.42047	2.42044
2.492	2.32885	2.32886	2.32884
2.50033	2.23485	2.23486	2.23484
2.50867	2.1393	2.13931	2.13929
2.517	2.0412	2.04121	2.04119
2.52533	1.9355	1.93551	1.93549

2.53367	1.83185	1.83186	1.83184
2.542	1.73055	1.73056	1.73054
2.55033	1.6398	1.63981	1.63979
2.55867	1.57075	1.57076	1.57074
2.567	1.51145	1.51146	1.51144
2.57533	1.4543	1.45431	1.45429
2.58367	1.3973	1.39731	1.39729
2.592	1.34485	1.34486	1.34484
2.60033	1.29505	1.29506	1.29504
2.60867	1.24795	1.24796	1.24794
2.617	1.20245	1.20246	1.20244
2.62533	1.1577	1.15771	1.15769
2.63367	1.1186	1.11861	1.11859
2.642	1.0838	1.08381	1.08379
2.65033	1.0465	1.04651	1.04649
2.65867	1.01075	1.01076	1.01074
2.667	0.97305	0.97306	0.97304
2.67533	0.93745	0.93746	0.93744
2.68367	0.8955	0.89551	0.89549
2.692	0.85095	0.85096	0.85094
2.70033	0.80495	0.80496	0.80494
2.70867	0.7479	0.74791	0.74789
2.717	0.6877	0.68771	0.68769
2.72533	0.63065	0.63066	0.63064
2.73367	0.57635	0.57636	0.57634
2.742	0.54025	0.54026	0.54024
2.75033	0.5185	0.51851	0.51849
2.75867	0.4991	0.49911	0.49909
2.767	0.47665	0.47666	0.47664
2.77533	0.4562	0.45621	0.45619
2.78367	0.43705	0.43706	0.43704
2.792	0.4177	0.41771	0.41769
2.80033	0.39635	0.39636	0.39634
2.80867	0.38475	0.38476	0.38474
2.817	0.37335	0.37336	0.37334
2.82533	0.3603	0.36031	0.36029
2.83367	0.35275	0.35276	0.35274
2.842	0.3461	0.34611	0.34609
2.85033	0.32825	0.32826	0.32824
2.85867	0.31485	0.31486	0.31484
2.867	0.2959	0.29591	0.29589
2.87533	0.27355	0.27356	0.27354
2.88367	0.246	0.24601	0.24599
2.892	0.2149	0.21491	0.21489

2.90033	0.1731	0.17311	0.17309
2.90867	0.1334	0.13341	0.13339
2.917	0.09505	0.09506	0.09504
2.92533	0.06915	0.06916	0.06914
2.93367	0.0613	0.06131	0.06129
2.942	0.0586	0.05861	0.05859
2.95033	0.0552	0.05521	0.05519
2.95867	0.04855	0.04856	0.04854
2.967	0.0474	0.04741	0.04739
2.97533	0.0439	0.04391	0.04389
2.98367	0.04205	0.04206	0.04204
2.992	0.0406	0.04061	0.04059
3.00033	0.0389	0.03891	0.03889
3.00867	0.04205	0.04206	0.04204
3.017	0.04705	0.04706	0.04704
3.02533	0.049	0.04901	0.04899
3.03367	0.04685	0.04686	0.04684
3.042	0.0437	0.04371	0.04369
3.05033	0.03865	0.03866	0.03864
3.05867	0.0281	0.02811	0.02809
3.067	0.0161	0.01611	0.01609
3.07533	7E-4	0.00071	0.00069
3.08367	-0.0277	-0.02769	-0.02771
3.092	-0.05675	-0.05674	-0.05676
3.10033	-0.0853	-0.08529	-0.08531
3.10867	-0.10845	-0.10844	-0.10846
3.117	-0.1126	-0.11259	-0.11261
3.12533	-0.10475	-0.10474	-0.10476
3.13367	-0.10115	-0.10114	-0.10116
3.142	-0.09915	-0.09914	-0.09916
3.15033	-0.09555	-0.09554	-0.09556
3.15867	-0.09015	-0.09014	-0.09016
3.167	-0.08905	-0.08904	-0.08906
3.17533	-0.09045	-0.09044	-0.09046
3.18367	-0.0831	-0.08309	-0.08311
3.192	-0.07655	-0.07654	-0.07656
3.20033	-0.0715	-0.07149	-0.07151
3.20867	-0.06465	-0.06464	-0.06466
3.217	-0.05945	-0.05944	-0.05946
3.22533	-0.06375	-0.06374	-0.06376
3.23367	-0.06365	-0.06364	-0.06366
3.242	-0.0705	-0.07049	-0.07051
3.25033	-0.08175	-0.08174	-0.08176
3.25867	-0.09605	-0.09604	-0.09606

3.267	-0.11995	-0.11994	-0.11996
3.27533	-0.1511	-0.15109	-0.15111
3.28367	-0.1793	-0.17929	-0.17931
3.292	-0.20625	-0.20624	-0.20626
3.30033	-0.22085	-0.22084	-0.22086
3.30867	-0.2184	-0.21839	-0.21841
3.317	-0.21475	-0.21474	-0.21476
3.32533	-0.21155	-0.21154	-0.21156
3.33367	-0.2085	-0.20849	-0.20851
3.342	-0.20235	-0.20234	-0.20236
3.35033	-0.1964	-0.19639	-0.19641
3.35867	-0.1854	-0.18539	-0.18541
3.367	-0.1723	-0.17229	-0.17231
3.37533	-0.1568	-0.15679	-0.15681
3.38367	-0.1339	-0.13389	-0.13391
3.392	-0.10955	-0.10954	-0.10956
3.40033	-0.0854	-0.08539	-0.08541
3.40867	-0.06485	-0.06484	-0.06486
3.417	-0.04405	-0.04404	-0.04406
3.42533	-0.0248	-0.02479	-0.02481
3.43367	-0.00965	-0.00964	-0.00966
3.442	0.00235	0.00236	0.00234
3.45033	0.00845	0.00846	0.00844
3.45867	0.0059	0.00591	0.00589
3.467	0.0015	0.00151	0.00149
3.47533	-0.0044	-0.00439	-0.00441
3.48367	-0.0063	-0.00629	-0.00631
3.492	0.00595	0.00596	0.00594
3.50033	0.0191	0.01911	0.01909
3.50867	0.02475	0.02476	0.02474
3.517	0.0248	0.02481	0.02479
3.52533	0.0179	0.01791	0.01789
3.53367	0.01055	0.01056	0.01054
3.542	-0.00715	-0.00714	-0.00716
3.55033	-0.0256	-0.02559	-0.02561
3.55867	-0.04415	-0.04414	-0.04416
3.567	-0.0615	-0.06149	-0.06151
3.57533	-0.0771	-0.07709	-0.07711
3.58367	-0.0951	-0.09509	-0.09511
3.592	-0.1156	-0.11559	-0.11561
3.60033	-0.13545	-0.13544	-0.13546
3.60867	-0.15405	-0.15404	-0.15406
3.617	-0.1779	-0.17789	-0.17791
3.62533	-0.2035	-0.20349	-0.20351

3.63367	-0.2297	-0.22969	-0.22971
3.642	-0.2658	-0.26579	-0.26581
3.65033	-0.3044	-0.30439	-0.30441
3.65867	-0.3406	-0.34059	-0.34061
3.667	-0.3721	-0.37209	-0.37211
3.67533	-0.38905	-0.38904	-0.38906
3.68367	-0.38935	-0.38934	-0.38936
3.692	-0.3909	-0.39089	-0.39091
3.70033	-0.3928	-0.39279	-0.39281
3.70867	-0.39395	-0.39394	-0.39396
3.717	-0.39465	-0.39464	-0.39466
3.72533	-0.39625	-0.39624	-0.39626
3.73367	-0.3978	-0.39779	-0.39781
3.742	-0.39405	-0.39404	-0.39406
3.75033	-0.3878	-0.38779	-0.38781
3.75867	-0.3832	-0.38319	-0.38321
3.767	-0.37795	-0.37794	-0.37796
3.77533	-0.3707	-0.37069	-0.37071
3.78367	-0.37	-0.36999	-0.37001
3.792	-0.3683	-0.36829	-0.36831
3.80033	-0.37045	-0.37044	-0.37046
3.80867	-0.3758	-0.37579	-0.37581
3.817	-0.3851	-0.38509	-0.38511
3.82533	-0.39845	-0.39844	-0.39846
3.83367	-0.41965	-0.41964	-0.41966
3.842	-0.438	-0.43799	-0.43801
3.85033	-0.45565	-0.45564	-0.45566
3.85867	-0.46115	-0.46114	-0.46116
3.867	-0.45055	-0.45054	-0.45056
3.87533	-0.4357	-0.43569	-0.43571
3.88367	-0.42775	-0.42774	-0.42776
3.892	-0.41935	-0.41934	-0.41936
3.90033	-0.4148	-0.41479	-0.41481
3.90867	-0.41205	-0.41204	-0.41206
3.917	-0.41375	-0.41374	-0.41376
3.92533	-0.4134	-0.41339	-0.41341
3.93367	-0.4163	-0.41629	-0.41631
3.942	-0.417	-0.41699	-0.41701
3.95033	-0.4154	-0.41539	-0.41541
3.95867	-0.41325	-0.41324	-0.41326
3.967	-0.4177	-0.41769	-0.41771
3.97533	-0.42035	-0.42034	-0.42036
3.98367	-0.42505	-0.42504	-0.42506
3.992	-0.43455	-0.43454	-0.43456

4.00033	-0.4476	-0.44759	-0.44761
4.00867	-0.465	-0.46499	-0.46501
4.017	-0.4922	-0.49219	-0.49221
4.02533	-0.5194	-0.51939	-0.51941
4.03367	-0.54815	-0.54814	-0.54816
4.042	-0.57075	-0.57074	-0.57076
4.05033	-0.57645	-0.57644	-0.57646
4.05867	-0.5716	-0.57159	-0.57161
4.067	-0.5674	-0.56739	-0.56741
4.07533	-0.5629	-0.56289	-0.56291
4.08367	-0.55915	-0.55914	-0.55916
4.092	-0.55585	-0.55584	-0.55586
4.10033	-0.55145	-0.55144	-0.55146
4.10867	-0.5488	-0.54879	-0.54881
4.117	-0.54415	-0.54414	-0.54416
4.12533	-0.53405	-0.53404	-0.53406
4.13367	-0.52505	-0.52504	-0.52506
4.142	-0.5188	-0.51879	-0.51881
4.15033	-0.51305	-0.51304	-0.51306
4.15867	-0.51175	-0.51174	-0.51176
4.167	-0.50965	-0.50964	-0.50966
4.17533	-0.51465	-0.51464	-0.51466
4.18367	-0.5231	-0.52309	-0.52311
4.192	-0.5349	-0.53489	-0.53491
4.20033	-0.55595	-0.55594	-0.55596
4.20867	-0.58415	-0.58414	-0.58416
4.217	-0.60815	-0.60814	-0.60816
4.22533	-0.63245	-0.63244	-0.63246
4.23367	-0.64355	-0.64354	-0.64356
4.242	-0.6399	-0.63989	-0.63991
4.25033	-0.63315	-0.63314	-0.63316
4.25867	-0.6288	-0.62879	-0.62881
4.267	-0.62515	-0.62514	-0.62516
4.27533	-0.61995	-0.61994	-0.61996
4.28367	-0.61655	-0.61654	-0.61656
4.292	-0.6168	-0.61679	-0.61681
4.30033	-0.6083	-0.60829	-0.60831
4.30867	-0.59995	-0.59994	-0.59996
4.317	-0.5923	-0.59229	-0.59231
4.32533	-0.58055	-0.58054	-0.58056
4.33367	-0.56765	-0.56764	-0.56766
4.342	-0.5653	-0.56529	-0.56531
4.35033	-0.5593	-0.55929	-0.55931
4.35867	-0.5586	-0.55859	-0.55861

4.367	-0.55915	-0.55914	-0.55916
4.37533	-0.5627	-0.56269	-0.56271
4.38367	-0.5682	-0.56819	-0.56821
4.392	-0.57985	-0.57984	-0.57986
4.40033	-0.589	-0.58899	-0.58901
4.40867	-0.5915	-0.59149	-0.59151
4.417	-0.57835	-0.57834	-0.57836
4.42533	-0.53875	-0.53874	-0.53876
4.43367	-0.47965	-0.47964	-0.47966
4.442	-0.40805	-0.40804	-0.40806
4.45033	-0.3253	-0.32529	-0.32531
4.45867	-0.22635	-0.22634	-0.22636
4.467	-0.1124	-0.11239	-0.11241
4.47533	0.01135	0.01136	0.01134
4.48367	0.14145	0.14146	0.14144
4.492	0.2752	0.27522	0.27519
4.50033	0.4034	0.40341	0.40339
4.50867	0.51695	0.51696	0.51694
4.517	0.6075	0.60751	0.60749
4.52533	0.67125	0.67126	0.67124
4.53367	0.7035	0.70351	0.70349
4.542	0.70945	0.70946	0.70944
4.55033	0.67855	0.67856	0.67854
4.55867	0.62105	0.62106	0.62104
4.567	0.5413	0.54131	0.54129
4.57533	0.43675	0.43676	0.43674
4.58367	0.3165	0.31651	0.31649
4.592	0.19335	0.19336	0.19334
4.60033	0.0709	0.07091	0.07089
4.60867	-0.0343	-0.03429	-0.03431
4.617	-0.11895	-0.11894	-0.11896
4.62533	-0.19425	-0.19424	-0.19426
4.63367	-0.26135	-0.26134	-0.26136
4.642	-0.32005	-0.32004	-0.32006
4.65033	-0.36835	-0.36834	-0.36836
4.65867	-0.41225	-0.41224	-0.41226
4.667	-0.45005	-0.45004	-0.45006
4.67533	-0.4736	-0.47359	-0.47361
4.68367	-0.4941	-0.49409	-0.49411
4.692	-0.50885	-0.50884	-0.50886
4.70033	-0.51895	-0.51894	-0.51896
4.70867	-0.5268	-0.52679	-0.52681
4.717	-0.53835	-0.53834	-0.53836
4.72533	-0.54485	-0.54484	-0.54486

4.73367	-0.55615	-0.55614	-0.55616
4.742	-0.5703	-0.57029	-0.57031
4.75033	-0.58755	-0.58754	-0.58756
4.75867	-0.6098	-0.60979	-0.60981
4.767	-0.64035	-0.64034	-0.64036
4.77533	-0.6696	-0.66959	-0.66961
4.78367	-0.6968	-0.69679	-0.69681
4.792	-0.71415	-0.71414	-0.71416
4.80033	-0.7123	-0.71229	-0.71231
4.80867	-0.70795	-0.70794	-0.70796
4.817	-0.70445	-0.70444	-0.70446
4.82533	-0.70265	-0.70264	-0.70266
4.83367	-0.69935	-0.69934	-0.69936
4.842	-0.69365	-0.69364	-0.69366
4.85033	-0.68965	-0.68964	-0.68966
4.85867	-0.68445	-0.68444	-0.68446
4.867	-0.6781	-0.67809	-0.67811
4.87533	-0.67265	-0.67264	-0.67266
4.88367	-0.6641	-0.66409	-0.66411
4.892	-0.6555	-0.65549	-0.65551
4.90033	-0.6517	-0.65169	-0.65171
4.90867	-0.64975	-0.64974	-0.64976
4.917	-0.64975	-0.64974	-0.64976
4.92533	-0.6559	-0.65589	-0.65591
4.93367	-0.66595	-0.66594	-0.66596
4.942	-0.6787	-0.67869	-0.67871
4.95033	-0.70305	-0.70304	-0.70306
4.95867	-0.7297	-0.72969	-0.72971
4.967	-0.7567	-0.75669	-0.75671
4.97533	-0.777	-0.77699	-0.77701
4.98367	-0.7825	-0.78249	-0.78251
4.992	-0.77365	-0.77364	-0.77366
5.00033	-0.76835	-0.76834	-0.76836
5.00867	-0.7637	-0.76369	-0.76371
5.017	-0.7588	-0.75879	-0.75881
5.02533	-0.7505	-0.75049	-0.75051
5.03367	-0.7496	-0.74959	-0.74961
5.042	-0.744	-0.74399	-0.74401
5.05033	-0.73925	-0.73924	-0.73926
5.05867	-0.7305	-0.73049	-0.73051
5.067	-0.71935	-0.71934	-0.71936
5.07533	-0.70865	-0.70864	-0.70866
5.08367	-0.70195	-0.70194	-0.70196
5.092	-0.70015	-0.70014	-0.70016

5.10033	-0.69715	-0.69714	-0.69716
5.10867	-0.7001	-0.70009	-0.70011
5.117	-0.70665	-0.70664	-0.70666
5.12533	-0.71605	-0.71604	-0.71606
5.13367	-0.7369	-0.73689	-0.73691
5.142	-0.7627	-0.76269	-0.76271
5.15033	-0.7877	-0.78769	-0.78771
5.15867	-0.8103	-0.81029	-0.81031
5.167	-0.82065	-0.82064	-0.82066
5.17533	-0.8164	-0.81639	-0.81641
5.18367	-0.80895	-0.80894	-0.80896
5.192	-0.80435	-0.80434	-0.80436
5.20033	-0.79885	-0.79884	-0.79886
5.20867	-0.7943	-0.79429	-0.79431
5.217	-0.7892	-0.78919	-0.78921
5.22533	-0.78615	-0.78614	-0.78616
5.23367	-0.77855	-0.77854	-0.77856
5.242	-0.76805	-0.76804	-0.76806
5.25033	-0.7608	-0.76079	-0.76081
5.25867	-0.75305	-0.75304	-0.75306
5.267	-0.7441	-0.74409	-0.74411
5.27533	-0.74025	-0.74024	-0.74026
5.28367	-0.7364	-0.73639	-0.73641
5.292	-0.73675	-0.73674	-0.73676
5.30033	-0.74225	-0.74224	-0.74226
5.30867	-0.7521	-0.75209	-0.75211
5.317	-0.7658	-0.76579	-0.76581
5.32533	-0.7908	-0.79079	-0.79081
5.33367	-0.81535	-0.81534	-0.81536
5.342	-0.84125	-0.84124	-0.84126
5.35033	-0.85925	-0.85924	-0.85926
5.35867	-0.8589	-0.85889	-0.85891
5.367	-0.85195	-0.85194	-0.85196
5.37533	-0.84765	-0.84764	-0.84766
5.38367	-0.8427	-0.84269	-0.84271
5.392	-0.83665	-0.83664	-0.83666
5.40033	-0.8303	-0.83029	-0.83031
5.40867	-0.8243	-0.82429	-0.82431
5.417	-0.81455	-0.81454	-0.81456
5.42533	-0.80905	-0.80904	-0.80906
5.43367	-0.8024	-0.80239	-0.80241
5.442	-0.791	-0.79099	-0.79101
5.45033	-0.7824	-0.78239	-0.78241
5.45867	-0.7792	-0.77919	-0.77921

5.467	-0.7778	-0.77779	-0.77781
5.47533	-0.7761	-0.77609	-0.77611
5.48367	-0.7796	-0.77959	-0.77961
5.492	-0.78835	-0.78834	-0.78836
5.50033	-0.79945	-0.79944	-0.79946
5.50867	-0.82165	-0.82164	-0.82166
5.517	-0.8486	-0.84859	-0.84861
5.52533	-0.87515	-0.87514	-0.87516
5.53367	-0.89795	-0.89794	-0.89796
5.542	-0.9059	-0.90589	-0.90591
5.55033	-0.89915	-0.89914	-0.89916
5.55867	-0.89195	-0.89194	-0.89196
5.567	-0.8867	-0.88669	-0.88671
5.57533	-0.88255	-0.88254	-0.88256
5.58367	-0.8767	-0.87669	-0.87671
5.592	-0.87155	-0.87154	-0.87156
5.60033	-0.86875	-0.86874	-0.86876
5.60867	-0.8611	-0.86109	-0.86111
5.617	-0.8513	-0.85129	-0.85131
5.62533	-0.8427	-0.84269	-0.84271
5.63367	-0.83515	-0.83514	-0.83516
5.642	-0.8254	-0.82539	-0.82541
5.65033	-0.8232	-0.82319	-0.82321
5.65867	-0.82235	-0.82234	-0.82236
5.667	-0.8243	-0.82429	-0.82431
5.67533	-0.83165	-0.83164	-0.83166
5.68367	-0.84355	-0.84354	-0.84356
5.692	-0.8596	-0.85959	-0.85961
5.70033	-0.8865	-0.88649	-0.88651
5.70867	-0.9116	-0.91159	-0.91161
5.717	-0.93775	-0.93774	-0.93776
5.72533	-0.95355	-0.95354	-0.95356
5.73367	-0.95235	-0.95234	-0.95236
5.742	-0.9463	-0.94629	-0.94631
5.75033	-0.94165	-0.94164	-0.94166
5.75867	-0.9392	-0.93919	-0.93921
5.767	-0.93385	-0.93384	-0.93386
5.77533	-0.92985	-0.92984	-0.92986
5.78367	-0.9231	-0.92309	-0.92311
5.792	-0.91595	-0.91594	-0.91596
5.80033	-0.91	-0.90999	-0.91001
5.80867	-0.90255	-0.90254	-0.90256
5.817	-0.8937	-0.89369	-0.89371
5.82533	-0.88655	-0.88654	-0.88656

5.83367	-0.8836	-0.88359	-0.88361
5.842	-0.8853	-0.88529	-0.88531
5.85033	-0.88605	-0.88604	-0.88606
5.85867	-0.8925	-0.89249	-0.89251
5.867	-0.9024	-0.90239	-0.90241
5.87533	-0.917	-0.91699	-0.91701
5.88367	-0.941	-0.94099	-0.94101
5.892	-0.9681	-0.96809	-0.96811
5.90033	-0.99465	-0.99464	-0.99466
5.90867	-1.01755	-1.01754	-1.01756
5.917	-1.02335	-1.02334	-1.02336
5.92533	-1.0179	-1.01789	-1.01791
5.93367	-1.01245	-1.01244	-1.01246
5.942	-1.0078	-1.00779	-1.00781
5.95033	-1.0019	-1.00189	-1.00191
5.95867	-0.9937	-0.99369	-0.99371
5.967	-0.989	-0.98899	-0.98901
5.97533	-0.98335	-0.98334	-0.98336
5.98367	-0.9751	-0.97509	-0.97511
5.992	-0.96625	-0.96624	-0.96626
6.00033	-0.9553	-0.95529	-0.95531
6.00867	-0.9425	-0.94249	-0.94251
6.017	-0.9352	-0.93519	-0.93521
6.02533	-0.9332	-0.93319	-0.93321
6.03367	-0.9291	-0.92909	-0.92911
6.042	-0.9317	-0.93169	-0.93171
6.05033	-0.94015	-0.94014	-0.94016
6.05867	-0.95375	-0.95374	-0.95376
6.067	-0.97355	-0.97354	-0.97356
6.07533	-1.00345	-1.00344	-1.00346
6.08367	-1.0334	-1.03339	-1.03341
6.092	-1.0616	-1.06159	-1.06161
6.10033	-1.07745	-1.07744	-1.07746
6.10867	-1.0759	-1.07589	-1.07591
6.117	-1.0747	-1.07469	-1.07471
6.12533	-1.0729	-1.07289	-1.07291
6.13367	-1.0729	-1.07289	-1.07291
6.142	-1.0714	-1.07139	-1.07141
6.15033	-1.07015	-1.07014	-1.07016
6.15867	-1.06555	-1.06554	-1.06556
6.167	-1.0644	-1.06439	-1.06441
6.17533	-1.06005	-1.06004	-1.06006
6.18367	-1.0531	-1.05309	-1.05311
6.192	-1.04725	-1.04724	-1.04726

6.20033	-1.0439	-1.04389	-1.04391
6.20867	-1.0413	-1.04129	-1.04131
6.217	-1.04135	-1.04134	-1.04136
6.22533	-1.04415	-1.04414	-1.04416
6.23367	-1.05395	-1.05394	-1.05396
6.242	-1.06545	-1.06544	-1.06546
6.25033	-1.0817	-1.08169	-1.08171
6.25867	-1.1093	-1.10929	-1.10931
6.267	-1.13665	-1.13664	-1.13666
6.27533	-1.1633	-1.16329	-1.16331
6.28367	-1.185	-1.18499	-1.18501
6.292	-1.18855	-1.18854	-1.18856
6.30033	-1.18535	-1.18534	-1.18536
6.30867	-1.18185	-1.18184	-1.18186
6.317	-1.1795	-1.17949	-1.17951
6.32533	-1.1779	-1.17789	-1.17791
6.33367	-1.1731	-1.17309	-1.17311
6.342	-1.17345	-1.17344	-1.17346
6.35033	-1.1694	-1.16939	-1.16941
6.35867	-1.163	-1.16299	-1.16301
6.367	-1.15885	-1.15884	-1.15886
6.37533	-1.15345	-1.15344	-1.15346
6.38367	-1.1429	-1.14289	-1.14291
6.392	-1.13885	-1.13884	-1.13886
6.40033	-1.14165	-1.14164	-1.14166
6.40867	-1.14165	-1.14164	-1.14166
6.417	-1.149	-1.14899	-1.14901
6.42533	-1.1605	-1.16049	-1.16051
6.43367	-1.17365	-1.17364	-1.17366
6.442	-1.19805	-1.19804	-1.19806
6.45033	-1.2266	-1.22659	-1.22661
6.45867	-1.257	-1.25699	-1.25701
6.467	-1.28325	-1.28324	-1.28326
6.47533	-1.2962	-1.29619	-1.29621
6.48367	-1.29235	-1.29234	-1.29236
6.492	-1.28845	-1.28844	-1.28846
6.50033	-1.2852	-1.28519	-1.28521
6.50867	-1.28415	-1.28414	-1.28416
6.517	-1.2802	-1.28019	-1.28021
6.52533	-1.2776	-1.27759	-1.27761
6.53367	-1.2727	-1.27269	-1.27271
6.542	-1.2704	-1.27039	-1.27041
6.55033	-1.2647	-1.26469	-1.26471
6.55867	-1.2567	-1.25669	-1.25671

6.567	-1.2513	-1.25129	-1.25131
6.57533	-1.24735	-1.24734	-1.24736
6.58367	-1.2465	-1.24649	-1.24651
6.592	-1.24555	-1.24554	-1.24556
6.60033	-1.24825	-1.24824	-1.24826
6.60867	-1.25855	-1.25854	-1.25856
6.617	-1.27155	-1.27154	-1.27156
6.62533	-1.2911	-1.29109	-1.29111
6.63367	-1.3202	-1.32019	-1.32021
6.642	-1.34765	-1.34764	-1.34766
6.65033	-1.37595	-1.37594	-1.37596
6.65867	-1.396	-1.39599	-1.39601
6.667	-1.39745	-1.39744	-1.39746
6.67533	-1.39305	-1.39304	-1.39306
6.68367	-1.39155	-1.39154	-1.39156
6.692	-1.3903	-1.39029	-1.39031
6.70033	-1.38775	-1.38774	-1.38776
6.70867	-1.38485	-1.38484	-1.38486
6.717	-1.3838	-1.38379	-1.38381
6.72533	-1.379	-1.37899	-1.37901
6.73367	-1.37145	-1.37144	-1.37146
6.742	-1.3669	-1.36689	-1.36691
6.75033	-1.36065	-1.36064	-1.36066
6.75867	-1.35245	-1.35244	-1.35246
6.767	-1.35	-1.34999	-1.35001
6.77533	-1.3502	-1.35019	-1.35021
6.78367	-1.3507	-1.35069	-1.35071
6.792	-1.3589	-1.35889	-1.35891
6.80033	-1.37145	-1.37144	-1.37146
6.80867	-1.38515	-1.38514	-1.38516
6.817	-1.4114	-1.41139	-1.41141
6.82533	-1.4403	-1.44029	-1.44031
6.83367	-1.4699	-1.46989	-1.46991
6.842	-1.49465	-1.49464	-1.49466
6.85033	-1.5045	-1.50449	-1.50451
6.85867	-1.5005	-1.50049	-1.50051
6.867	-1.4976	-1.49759	-1.49761
6.87533	-1.49585	-1.49584	-1.49586
6.88367	-1.4934	-1.49339	-1.49341
6.892	-1.49075	-1.49074	-1.49076
6.90033	-1.48565	-1.48564	-1.48566
6.90867	-1.48065	-1.48064	-1.48066
6.917	-1.4776	-1.47759	-1.47761
6.92533	-1.4725	-1.47249	-1.47251

6.93367	-1.4662	-1.46619	-1.46621
6.942	-1.4593	-1.45929	-1.45931
6.95033	-1.4537	-1.45369	-1.45371
6.95867	-1.45665	-1.45664	-1.45666
6.967	-1.4571	-1.45709	-1.45711
6.97533	-1.46135	-1.46134	-1.46136
6.98367	-1.4723	-1.47229	-1.47231
6.992	-1.48455	-1.48454	-1.48456
7.00033	-1.50515	-1.50514	-1.50516
7.00867	-1.53595	-1.53594	-1.53596
7.017	-1.56415	-1.56414	-1.56416
7.02533	-1.59305	-1.59304	-1.59306
7.03367	-1.6075	-1.60749	-1.60751
7.042	-1.60665	-1.60664	-1.60666
7.05033	-1.6004	-1.60039	-1.60041
7.05867	-1.5972	-1.59719	-1.59721
7.067	-1.59515	-1.59514	-1.59516
7.07533	-1.59245	-1.59244	-1.59246
7.08367	-1.5876	-1.58759	-1.58761
7.092	-1.5869	-1.58689	-1.58691
7.10033	-1.58295	-1.58294	-1.58296
7.10867	-1.5777	-1.57769	-1.57771
7.117	-1.5699	-1.56989	-1.56991
7.12533	-1.5616	-1.56159	-1.56161
7.13367	-1.5564	-1.55639	-1.55641
7.142	-1.55395	-1.55394	-1.55396
7.15033	-1.552	-1.55199	-1.55201
7.15867	-1.55485	-1.55484	-1.55486
7.167	-1.56285	-1.56284	-1.56286
7.17533	-1.5745	-1.57449	-1.57451
7.18367	-1.59115	-1.59114	-1.59116
7.192	-1.6185	-1.61849	-1.61851
7.20033	-1.64685	-1.64684	-1.64686
7.20867	-1.6756	-1.67559	-1.67561
7.217	-1.6979	-1.69789	-1.69791
7.22533	-1.70215	-1.70214	-1.70216
7.23367	-1.6975	-1.69749	-1.69751
7.242	-1.69505	-1.69504	-1.69506
7.25033	-1.69295	-1.69294	-1.69296
7.25867	-1.6904	-1.69039	-1.69041
7.267	-1.6865	-1.68649	-1.68651
7.27533	-1.6809	-1.68089	-1.68091
7.28367	-1.67605	-1.67604	-1.67606
7.292	-1.6686	-1.66859	-1.66861

7.30033	-1.66355	-1.66354	-1.66356
7.30867	-1.65715	-1.65714	-1.65716
7.317	-1.65055	-1.65054	-1.65056
7.32533	-1.64355	-1.64354	-1.64356
7.33367	-1.6464	-1.64639	-1.64641
7.342	-1.645	-1.64499	-1.64501
7.35033	-1.65205	-1.65204	-1.65206
7.35867	-1.66315	-1.66314	-1.66316
7.367	-1.6755	-1.67549	-1.67551
7.37533	-1.69785	-1.69784	-1.69786
7.38367	-1.72815	-1.72814	-1.72816
7.392	-1.75605	-1.75604	-1.75606
7.40033	-1.78335	-1.78334	-1.78336
7.40867	-1.79515	-1.79514	-1.79516
7.417	-1.79235	-1.79234	-1.79236
7.42533	-1.78735	-1.78734	-1.78736
7.43367	-1.78365	-1.78364	-1.78366
7.442	-1.78025	-1.78024	-1.78026
7.45033	-1.7739	-1.77389	-1.77391
7.45867	-1.7722	-1.77219	-1.77221
7.467	-1.76885	-1.76884	-1.76886
7.47533	-1.76325	-1.76324	-1.76326
7.48367	-1.7595	-1.75949	-1.75951
7.492	-1.75065	-1.75064	-1.75066
7.50033	-1.741	-1.74099	-1.74101
7.50867	-1.7357	-1.73569	-1.73571
7.517	-1.733	-1.73299	-1.73301
7.52533	-1.7324	-1.73239	-1.73241
7.53367	-1.73325	-1.73324	-1.73326
7.542	-1.74295	-1.74294	-1.74296
7.55033	-1.7526	-1.75259	-1.75261
7.55867	-1.77055	-1.77054	-1.77056
7.567	-1.7985	-1.79849	-1.79851
7.57533	-1.8272	-1.82719	-1.82721
7.58367	-1.85375	-1.85374	-1.85376
7.592	-1.87365	-1.87364	-1.87366
7.60033	-1.87305	-1.87304	-1.87306
7.60867	-1.866	-1.86599	-1.86601
7.617	-1.8614	-1.86139	-1.86141
7.62533	-1.85975	-1.85974	-1.85976
7.63367	-1.8552	-1.85519	-1.85521
7.642	-1.84915	-1.84914	-1.84916
7.65033	-1.849	-1.84899	-1.84901
7.65867	-1.8482	-1.84819	-1.84821

7.667	-1.83915	-1.83914	-1.83916
7.67533	-1.82835	-1.82834	-1.82836
7.68367	-1.82095	-1.82094	-1.82096
7.692	-1.81045	-1.81044	-1.81046
7.70033	-1.8045	-1.80449	-1.80451
7.70867	-1.80485	-1.80484	-1.80486
7.717	-1.80155	-1.80154	-1.80156
7.72533	-1.8086	-1.80859	-1.80861
7.73367	-1.81765	-1.81764	-1.81766
7.742	-1.83085	-1.83084	-1.83086
7.75033	-1.85475	-1.85474	-1.85476
7.75867	-1.88165	-1.88164	-1.88166
7.767	-1.9086	-1.90859	-1.90861
7.77533	-1.93365	-1.93364	-1.93366
7.78367	-1.94135	-1.94134	-1.94136
7.792	-1.9345	-1.93449	-1.93451
7.80033	-1.93045	-1.93044	-1.93046
7.80867	-1.92515	-1.92514	-1.92516
7.817	-1.9206	-1.92059	-1.92061
7.82533	-1.9142	-1.91419	-1.91421
7.83367	-1.91065	-1.91064	-1.91066
7.842	-1.90445	-1.90444	-1.90446
7.85033	-1.89785	-1.89784	-1.89786
7.85867	-1.8923	-1.89229	-1.89231
7.867	-1.88435	-1.88434	-1.88436
7.87533	-1.87475	-1.87474	-1.87476
7.88367	-1.8679	-1.86789	-1.86791
7.892	-1.86465	-1.86464	-1.86466
7.90033	-1.86175	-1.86174	-1.86176
7.90867	-1.86255	-1.86254	-1.86256
7.917	-1.872	-1.87199	-1.87201
7.92533	-1.88165	-1.88164	-1.88166
7.93367	-1.90115	-1.90114	-1.90116
7.942	-1.92925	-1.92924	-1.92926
7.95033	-1.95815	-1.95814	-1.95816
7.95867	-1.98345	-1.98344	-1.98346
7.967	-1.9833	-1.98329	-1.98331
7.97533	-1.99385	-1.99384	-1.99386
7.98367	-1.9869	-1.98689	-1.98691
7.992	-1.9819	-1.98189	-1.98191
8.00033	-1.9769	-1.97689	-1.97691
8.00867	-1.97175	-1.97174	-1.97176
8.017	-1.96625	-1.96624	-1.96626
8.02533	-1.9642	-1.96419	-1.96421

8.03367	-1.9585	-1.95849	-1.95851
8.042	-1.94975	-1.94974	-1.94976
8.05033	-1.93985	-1.93984	-1.93986
8.05867	-1.9309	-1.93089	-1.93091
8.067	-1.9197	-1.91969	-1.91971
8.07533	-1.9172	-1.91719	-1.91721
8.08367	-1.9126	-1.91259	-1.91261
8.092	-1.91065	-1.91064	-1.91066
8.10033	-1.91635	-1.91634	-1.91636
8.10867	-1.9244	-1.92439	-1.92441
8.117	-1.9362	-1.93619	-1.93621
8.12533	-1.96065	-1.96064	-1.96066
8.13367	-1.9864	-1.98639	-1.98641
8.142	-2.0101	-2.01009	-2.01011
8.15033	-2.029	-2.02899	-2.02901
8.15867	-2.0283	-2.02829	-2.02831
8.167	-2.01725	-2.01724	-2.01726
8.17533	-2.00865	-2.00864	-2.00866
8.18367	-2.0021	-2.00209	-2.00211
8.192	-1.9921	-1.99209	-1.99211
8.20033	-1.9812	-1.98119	-1.98121
8.20867	-1.9715	-1.97149	-1.97151
8.217	-1.95845	-1.95844	-1.95846
8.22533	-1.94365	-1.94364	-1.94366
8.23367	-1.92675	-1.92674	-1.92676
8.242	-1.9086	-1.90859	-1.90861
8.25033	-1.88975	-1.88974	-1.88976
8.25867	-1.8699	-1.86989	-1.86991
8.267	-1.85415	-1.85414	-1.85416
8.27533	-1.83595	-1.83594	-1.83596
8.28367	-1.8215	-1.82149	-1.82151
8.292	-1.8072	-1.80719	-1.80721
8.30033	-1.79185	-1.79184	-1.79186
8.30867	-1.7835	-1.78349	-1.78351
8.317	-1.77395	-1.77394	-1.77396
8.32533	-1.7568	-1.75679	-1.75681
8.33367	-1.73275	-1.73274	-1.73276
8.342	-1.6864	-1.68639	-1.68641
8.35033	-1.61455	-1.61454	-1.61456
8.35867	-1.53105	-1.53104	-1.53106
8.367	-1.43995	-1.43994	-1.43996
8.37533	-1.33565	-1.33564	-1.33566
8.38367	-1.22165	-1.22164	-1.22166
8.392	-1.09505	-1.09504	-1.09506

8.40033	-0.95715	-0.95714	-0.95716
8.40867	-0.8001	-0.80009	-0.80011
8.417	-0.63	-0.62999	-0.63001
8.42533	-0.44705	-0.44704	-0.44706
8.43367	-0.2509	-0.25089	-0.25091
8.442	-0.0448	-0.04479	-0.04481
8.45033	0.1603	0.16031	0.16029
8.45867	0.3717	0.37171	0.37169
8.467	0.582	0.58201	0.58199
8.47533	0.7883	0.78831	0.78829
8.48367	0.9865	0.98651	0.98649
8.492	1.17345	1.17346	1.17344
8.50033	1.3432	1.34321	1.34319
8.50867	1.49855	1.49856	1.49854
8.517	1.6316	1.63161	1.63159
8.52533	1.7494	1.74941	1.74939
8.53367	1.8472	1.84721	1.84719
8.542	1.91785	1.91786	1.91784
8.55033	1.95285	1.95286	1.95284
8.55867	1.95235	1.95236	1.95234
8.567	1.9216	1.92161	1.92158
8.57533	1.85545	1.85546	1.85544
8.58367	1.76355	1.76356	1.76354
8.592	1.6442	1.64421	1.64419
8.60033	1.5038	1.50381	1.50379
8.60867	1.34775	1.34776	1.34774
8.617	1.1782	1.17821	1.17819
8.62533	0.996	0.99601	0.99599
8.63367	0.8055	0.80551	0.80549
8.642	0.61065	0.61066	0.61064
8.65033	0.4148	0.41481	0.41479
8.65867	0.21695	0.21696	0.21694
8.667	0.0204	0.02041	0.02039
8.67533	-0.1728	-0.17279	-0.17281
8.68367	-0.3671	-0.36709	-0.36711
8.692	-0.5542	-0.55419	-0.55421
8.70033	-0.7311	-0.73109	-0.73111
8.70867	-0.8945	-0.89449	-0.89451
8.717	-1.0318	-1.03179	-1.03181
8.72533	-1.14495	-1.14494	-1.14496
8.73367	-1.2425	-1.24249	-1.24251
8.742	-1.32885	-1.32884	-1.32886
8.75033	-1.40465	-1.40464	-1.40466
8.75867	-1.46765	-1.46764	-1.46766

8.767	-1.52085	-1.52084	-1.52086
8.77533	-1.5664	-1.56639	-1.56641
8.78367	-1.59725	-1.59724	-1.59726
8.792	-1.62105	-1.62104	-1.62106
8.80033	-1.63815	-1.63814	-1.63816
8.80867	-1.6444	-1.64439	-1.64441
8.817	-1.646	-1.64599	-1.64601
8.82533	-1.6471	-1.64709	-1.64711
8.83367	-1.64	-1.63999	-1.64001
8.842	-1.63235	-1.63234	-1.63236
8.85033	-1.62285	-1.62284	-1.62286
8.85867	-1.6078	-1.60779	-1.60781
8.867	-1.59425	-1.59424	-1.59426
8.87533	-1.5816	-1.58159	-1.58161
8.88367	-1.55925	-1.55924	-1.55926
8.892	-1.52595	-1.52594	-1.52596
8.90033	-1.46895	-1.46894	-1.46896
8.90867	-1.3851	-1.38509	-1.38511
8.917	-1.2872	-1.28719	-1.28721
8.92533	-1.1798	-1.17979	-1.17981
8.93367	-1.0632	-1.06319	-1.06321
8.942	-0.9332	-0.93319	-0.93321
8.95033	-0.794	-0.79399	-0.79401
8.95867	-0.64455	-0.64454	-0.64456
8.967	-0.4859	-0.48589	-0.48591
8.97533	-0.32045	-0.32044	-0.32046
8.98367	-0.1454	-0.14539	-0.14541
8.992	0.03435	0.03436	0.03434
9.00033	0.2114	0.21141	0.21139
9.00867	0.38545	0.38546	0.38544
9.017	0.5517	0.55171	0.55169
9.02533	0.70935	0.70936	0.70934
9.03367	0.8528	0.85281	0.85279
9.042	0.98345	0.98346	0.98344
9.05033	1.0961	1.09611	1.09609
9.05867	1.18225	1.18226	1.18224
9.067	1.24935	1.24936	1.24934
9.07533	1.29395	1.29396	1.29394
9.08367	1.3176	1.31761	1.31759
9.092	1.3288	1.32881	1.32879
9.10033	1.31845	1.31846	1.31844
9.10867	1.2824	1.28241	1.28239
9.117	1.22095	1.22096	1.22094
9.12533	1.1386	1.13861	1.13859

9.13367	1.0392	1.03921	1.03919
9.142	0.91805	0.91806	0.91804
9.15033	0.78575	0.78576	0.78574
9.15867	0.64725	0.64726	0.64724
9.167	0.50135	0.50137	0.50132
9.17533	0.3506	0.35061	0.35059
9.18367	0.2006	0.20061	0.20059
9.192	0.04975	0.04976	0.04974
9.20033	-0.10255	-0.10254	-0.10256
9.20867	-0.24775	-0.24774	-0.24776
9.217	-0.39285	-0.39284	-0.39286
9.22533	-0.5367	-0.53669	-0.53671
9.23367	-0.6711	-0.67109	-0.67111
9.242	-0.8085	-0.80849	-0.80851
9.25033	-0.94185	-0.94184	-0.94186
9.25867	-1.06595	-1.06594	-1.06596
9.267	-1.1802	-1.18019	-1.18021
9.27533	-1.2728	-1.27279	-1.27281
9.28367	-1.34005	-1.34004	-1.34006
9.292	-1.3965	-1.39649	-1.39651
9.30033	-1.4473	-1.44729	-1.44731
9.30867	-1.4892	-1.48919	-1.48921
9.317	-1.5231	-1.52309	-1.52311
9.32533	-1.55315	-1.55314	-1.55316
9.33367	-1.5792	-1.57919	-1.57921
9.342	-1.5981	-1.59809	-1.59811
9.35033	-1.6125	-1.61249	-1.61251
9.35867	-1.62375	-1.62374	-1.62376
9.367	-1.63275	-1.63274	-1.63276
9.37533	-1.6411	-1.64109	-1.64111
9.38367	-1.654	-1.65399	-1.65401
9.392	-1.6676	-1.66759	-1.66761
9.40033	-1.6822	-1.68219	-1.68221
9.40867	-1.70335	-1.70334	-1.70336
9.417	-1.7275	-1.72749	-1.72751
9.42533	-1.75935	-1.75934	-1.75936
9.43367	-1.80115	-1.80114	-1.80116
9.442	-1.84295	-1.84294	-1.84296
9.45033	-1.8861	-1.88609	-1.88611
9.45867	-1.92055	-1.92054	-1.92056
9.467	-1.9359	-1.93589	-1.93591
9.47533	-1.9454	-1.94539	-1.94541
9.48367	-1.95645	-1.95644	-1.95646
9.492	-1.96815	-1.96814	-1.96816

9.50033	-1.9802	-1.98019	-1.98021
9.50867	-1.98975	-1.98974	-1.98976
9.517	-2.0024	-2.00239	-2.00241
9.52533	-2.01235	-2.01234	-2.01236
9.53367	-2.0186	-2.01859	-2.01861
9.542	-2.0243	-2.02429	-2.02431
9.55033	-2.0263	-2.02629	-2.02631
9.55867	-2.0266	-2.02659	-2.02661
9.567	-2.03455	-2.03454	-2.03456
9.57533	-2.04185	-2.04184	-2.04186
9.58367	-2.05015	-2.05014	-2.05016
9.592	-2.0637	-2.06369	-2.06371
9.60033	-2.08225	-2.08224	-2.08226
9.60867	-2.1009	-2.10089	-2.10091
9.617	-2.13145	-2.13144	-2.13146
9.62533	-2.16545	-2.16544	-2.16546
9.63367	-2.19745	-2.19744	-2.19746
9.642	-2.225	-2.22499	-2.22501
9.65033	-2.2358	-2.23579	-2.23581
9.65867	-2.23325	-2.23324	-2.23326
9.667	-2.22905	-2.22904	-2.22906
9.67533	-2.2284	-2.22839	-2.22841
9.68367	-2.22605	-2.22604	-2.22606
9.692	-2.22175	-2.22174	-2.22176
9.70033	-2.21775	-2.21774	-2.21776
9.70867	-2.21465	-2.21464	-2.21466
9.717	-2.2088	-2.20879	-2.20881
9.72533	-2.1982	-2.19819	-2.19821
9.73367	-2.19115	-2.19114	-2.19116
9.742	-2.18495	-2.18494	-2.18496
9.75033	-2.17435	-2.17434	-2.17436
9.75867	-2.17325	-2.17324	-2.17326
9.767	-2.1691	-2.16909	-2.16911
9.77533	-2.17175	-2.17174	-2.17176
9.78367	-2.1809	-2.18089	-2.18091
9.792	-2.1895	-2.18949	-2.18951
9.80033	-2.2084	-2.20839	-2.20841
9.80867	-2.23695	-2.23694	-2.23696
9.817	-2.26225	-2.26224	-2.26226
9.82533	-2.2886	-2.28859	-2.28861
9.83367	-2.30325	-2.30324	-2.30326
9.842	-2.30025	-2.30024	-2.30026
9.85033	-2.2921	-2.29209	-2.29211
9.85867	-2.2869	-2.28689	-2.28691

9.867	-2.28055	-2.28054	-2.28056
9.87533	-2.2732	-2.27319	-2.27321
9.88367	-2.26785	-2.26784	-2.26786
9.892	-2.26345	-2.26344	-2.26346
9.90033	-2.25595	-2.25594	-2.25596
9.90867	-2.24865	-2.24864	-2.24866
9.917	-2.24125	-2.24124	-2.24126
9.92533	-2.22665	-2.22664	-2.22666
9.93367	-2.21555	-2.21554	-2.21556
9.942	-2.2135	-2.21349	-2.21351
9.95033	-2.20835	-2.20834	-2.20836
9.95867	-2.2047	-2.20469	-2.20471
9.967	-2.2094	-2.20939	-2.20941
9.97533	-2.218	-2.21799	-2.21801
9.98367	-2.2314	-2.23139	-2.23141
9.992	-2.25425	-2.25424	-2.25426
10.00033	-2.28135	-2.28134	-2.28136
10.00867	-2.3065	-2.30649	-2.30651
10.017	-2.32495	-2.32494	-2.32496
10.02533	-2.3248	-2.32479	-2.32481
10.03367	-2.31595	-2.31594	-2.31596
10.042	-2.3067	-2.30669	-2.30671
10.05033	-2.29995	-2.29994	-2.29996
10.05867	-2.2924	-2.29239	-2.29241
10.067	-2.2839	-2.28389	-2.28391
10.07533	-2.2749	-2.27489	-2.27491
10.08367	-2.2688	-2.26879	-2.26881
10.092	-2.25815	-2.25814	-2.25816
10.10033	-2.24445	-2.24444	-2.24446
10.10867	-2.23285	-2.23284	-2.23286
10.117	-2.2213	-2.22129	-2.22131
10.12533	-2.2088	-2.20879	-2.20881
10.13367	-2.2034	-2.20339	-2.20341
10.142	-2.1978	-2.19779	-2.19781
10.15033	-2.20015	-2.20014	-2.20016
10.15867	-2.20585	-2.20584	-2.20586
10.167	-2.21565	-2.21564	-2.21566
10.17533	-2.2346	-2.23459	-2.23461
10.18367	-2.2593	-2.25929	-2.25931
10.192	-2.28215	-2.28214	-2.28216
10.20033	-2.30525	-2.30524	-2.30526
10.20867	-2.31425	-2.31424	-2.31426
10.217	-2.30765	-2.30764	-2.30766
10.22533	-2.2996	-2.29959	-2.29961

10.23367	-2.2924	-2.29239	-2.29241
10.242	-2.2862	-2.28619	-2.28621
10.25033	-2.2777	-2.27769	-2.27771
10.25867	-2.27235	-2.27234	-2.27236
10.267	-2.27145	-2.27144	-2.27146
10.27533	-2.26305	-2.26304	-2.26306
10.28367	-2.25565	-2.25564	-2.25566
10.292	-2.2458	-2.24579	-2.24581
10.30033	-2.23445	-2.23444	-2.23446
10.30867	-2.2233	-2.22329	-2.22331
10.317	-2.2217	-2.22169	-2.22171
10.32533	-2.2187	-2.21869	-2.21871
10.33367	-2.22025	-2.22024	-2.22026
10.342	-2.2266	-2.22659	-2.22661
10.35033	-2.23545	-2.23544	-2.23546
10.35867	-2.2525	-2.25249	-2.25251
10.367	-2.2803	-2.28029	-2.28031
10.37533	-2.30955	-2.30954	-2.30956
10.38367	-2.3357	-2.33569	-2.33571
10.392	-2.3534	-2.35339	-2.35341
10.40033	-2.3521	-2.35209	-2.35211
10.40867	-2.34505	-2.34504	-2.34506
10.417	-2.3387	-2.33869	-2.33871
10.42533	-2.33515	-2.33514	-2.33516
10.43367	-2.32975	-2.32974	-2.32976
10.442	-2.3218	-2.32179	-2.32181
10.45033	-2.31745	-2.31744	-2.31746
10.45867	-2.31285	-2.31284	-2.31286
10.467	-2.3054	-2.30539	-2.30541
10.47533	-2.2954	-2.29539	-2.29541
10.48367	-2.284	-2.28399	-2.28401
10.492	-2.2728	-2.27279	-2.27281
10.50033	-2.26745	-2.26744	-2.26746
10.50867	-2.2639	-2.26389	-2.26391
10.517	-2.2608	-2.26079	-2.26081
10.52533	-2.26585	-2.26584	-2.26586
10.53367	-2.2743	-2.27429	-2.27431
10.542	-2.28605	-2.28604	-2.28606
10.55033	-2.3077	-2.30769	-2.30771
10.55867	-2.33495	-2.33494	-2.33496
10.567	-2.36065	-2.36064	-2.36066
10.57533	-2.38315	-2.38314	-2.38316
10.58367	-2.38865	-2.38864	-2.38866
10.592	-2.3807	-2.38069	-2.38071

10.60033	-2.37315	-2.37314	-2.37316
10.60867	-2.3668	-2.36679	-2.36681
10.617	-2.3609	-2.36089	-2.36091
10.62533	-2.35325	-2.35324	-2.35326
10.63367	-2.34975	-2.34974	-2.34976
10.642	-2.34535	-2.34534	-2.34536
10.65033	-2.3357	-2.33569	-2.33571
10.65867	-2.324	-2.32399	-2.32401
10.667	-2.3122	-2.31219	-2.31221
10.67533	-2.302	-2.30199	-2.30201
10.68367	-2.29395	-2.29394	-2.29396
10.692	-2.2896	-2.28959	-2.28961
10.70033	-2.2852	-2.28519	-2.28521
10.70867	-2.28465	-2.28464	-2.28466
10.717	-2.29095	-2.29094	-2.29096
10.72533	-2.30055	-2.30054	-2.30056
10.73367	-2.31815	-2.31814	-2.31816
10.742	-2.34405	-2.34404	-2.34406
10.75033	-2.3712	-2.37119	-2.37121
10.75867	-2.3944	-2.39439	-2.39441
10.767	-2.4079	-2.40789	-2.40791
10.77533	-2.4033	-2.40329	-2.40331
10.78367	-2.39365	-2.39364	-2.39366
10.792	-2.3868	-2.38679	-2.38681
10.80033	-2.3785	-2.37849	-2.37851
10.80867	-2.37195	-2.37194	-2.37196
10.817	-2.36425	-2.36424	-2.36426
10.82533	-2.3627	-2.36269	-2.36271
10.83367	-2.354	-2.35399	-2.35401
10.842	-2.34285	-2.34284	-2.34286
10.85033	-2.3334	-2.33339	-2.33341
10.85867	-2.32125	-2.32124	-2.32126
10.867	-2.3059	-2.30589	-2.30591
10.87533	-2.3015	-2.30149	-2.30151
10.88367	-2.2962	-2.29619	-2.29621
10.892	-2.2933	-2.29329	-2.29331
10.90033	-2.29415	-2.29414	-2.29416
10.90867	-2.3008	-2.30079	-2.30081
10.917	-2.3105	-2.31049	-2.31051
10.92533	-2.3321	-2.33209	-2.33211
10.93367	-2.35675	-2.35674	-2.35676
10.942	-2.3801	-2.38009	-2.38011
10.95033	-2.39615	-2.39614	-2.39616
10.95867	-2.3932	-2.39319	-2.39321

10.967	-2.37875	-2.37874	-2.37876
10.97533	-2.3666	-2.36659	-2.36661
10.98367	-2.35505	-2.35504	-2.35506
10.992	-2.3424	-2.34239	-2.34241
11.00033	-2.327	-2.32699	-2.32701
11.00867	-2.315	-2.31499	-2.31501
11.017	-2.2994	-2.29939	-2.29941
11.02533	-2.28255	-2.28254	-2.28256
11.03367	-2.2618	-2.26179	-2.26181
11.042	-2.237	-2.23699	-2.23701
11.05033	-2.2149	-2.21489	-2.21491
11.05867	-2.19315	-2.19314	-2.19316
11.067	-2.1727	-2.17269	-2.17271
11.07533	-2.15125	-2.15124	-2.15126
11.08367	-2.1342	-2.13419	-2.13421
11.092	-2.1207	-2.12069	-2.12071
11.10033	-2.10705	-2.10704	-2.10706
11.10867	-2.10165	-2.10164	-2.10166
11.117	-2.09955	-2.09954	-2.09956
11.12533	-2.093	-2.09299	-2.09301
11.13367	-2.07975	-2.07974	-2.07976
11.142	-2.05085	-2.05084	-2.05086
11.15033	-2.00285	-2.00284	-2.00286
11.15867	-1.94835	-1.94834	-1.94836
11.167	-1.89345	-1.89344	-1.89346
11.17533	-1.83455	-1.83454	-1.83456
11.18367	-1.77085	-1.77084	-1.77086
11.192	-1.7054	-1.70539	-1.70541
11.20033	-1.64155	-1.64154	-1.64156
11.20867	-1.56625	-1.56624	-1.56626
11.217	-1.48715	-1.48714	-1.48716
11.22533	-1.40525	-1.40524	-1.40526
11.23367	-1.31975	-1.31974	-1.31976
11.242	-1.23115	-1.23114	-1.23116
11.25033	-1.1491	-1.14909	-1.14911
11.25867	-1.0639	-1.06389	-1.06391
11.267	-0.9852	-0.98519	-0.98521
11.27533	-0.9096	-0.90959	-0.90961
11.28367	-0.8367	-0.83669	-0.83671
11.292	-0.77255	-0.77254	-0.77256
11.30033	-0.7192	-0.71919	-0.71921
11.30867	-0.66615	-0.66614	-0.66616
11.317	-0.6176	-0.61759	-0.61761
11.32533	-0.565	-0.56499	-0.56501

11.33367	-0.5035	-0.50349	-0.50351
11.342	-0.44335	-0.44334	-0.44336
11.35033	-0.39265	-0.39264	-0.39266
11.35867	-0.35245	-0.35244	-0.35246
11.367	-0.31945	-0.31944	-0.31946
11.37533	-0.29815	-0.29814	-0.29817
11.38367	-0.28395	-0.28394	-0.28396
11.392	-0.2792	-0.27919	-0.27921
11.40033	-0.28335	-0.28334	-0.28336
11.40867	-0.29425	-0.29424	-0.29426
11.417	-0.31085	-0.31084	-0.31086
11.42533	-0.33885	-0.33884	-0.33886
11.43367	-0.3761	-0.37609	-0.37611
11.442	-0.4223	-0.42229	-0.42231
11.45033	-0.47515	-0.47514	-0.47516
11.45867	-0.54075	-0.54074	-0.54076
11.467	-0.6134	-0.61339	-0.61341
11.47533	-0.6944	-0.69439	-0.69441
11.48367	-0.78805	-0.78804	-0.78806
11.492	-0.8891	-0.88909	-0.88911
11.50033	-0.9908	-0.99079	-0.99081
11.50867	-1.08945	-1.08944	-1.08946
11.517	-1.17445	-1.17444	-1.17446
11.52533	-1.2477	-1.24769	-1.24771
11.53367	-1.31925	-1.31924	-1.31926
11.542	-1.389	-1.38899	-1.38901
11.55033	-1.4538	-1.45379	-1.45381
11.55867	-1.514	-1.51399	-1.51401
11.567	-1.57425	-1.57424	-1.57426
11.57533	-1.63035	-1.63034	-1.63036
11.58367	-1.6763	-1.67629	-1.67631
11.592	-1.71825	-1.71824	-1.71826
11.60033	-1.75475	-1.75474	-1.75476
11.60867	-1.7854	-1.78539	-1.78541
11.617	-1.81615	-1.81614	-1.81616
11.62533	-1.8492	-1.84919	-1.84921
11.63367	-1.8764	-1.87639	-1.87641
11.642	-1.90595	-1.90594	-1.90596
11.65033	-1.93635	-1.93634	-1.93636
11.65867	-1.9679	-1.96789	-1.96791
11.667	-2.00555	-2.00554	-2.00556
11.67533	-2.0472	-2.04719	-2.04721
11.68367	-2.08485	-2.08484	-2.08486
11.692	-2.1208	-2.12079	-2.12081

11.70033	-2.14205	-2.14204	-2.14206
11.70867	-2.14185	-2.14184	-2.14186
11.717	-2.1384	-2.13839	-2.13841
11.72533	-2.1331	-2.13309	-2.13311
11.73367	-2.12725	-2.12724	-2.12726
11.742	-2.11795	-2.11794	-2.11796
11.75033	-2.1085	-2.10849	-2.10851
11.75867	-2.09825	-2.09824	-2.09826
11.767	-2.08495	-2.08494	-2.08496
11.77533	-2.0681	-2.06809	-2.06811
11.78367	-2.0501	-2.05009	-2.05011
11.792	-2.0305	-2.03049	-2.03051
11.80033	-2.0103	-2.01029	-2.01031
11.80867	-1.9935	-1.99349	-1.99351
11.817	-1.97985	-1.97984	-1.97986
11.82533	-1.96615	-1.96614	-1.96616
11.83367	-1.9588	-1.95879	-1.95881
11.842	-1.9543	-1.95429	-1.95431
11.85033	-1.9538	-1.95379	-1.95381
11.85867	-1.9635	-1.96349	-1.96351
11.867	-1.973	-1.97299	-1.97301
11.87533	-1.98105	-1.98104	-1.98106
11.88367	-1.9812	-1.98119	-1.98121
11.892	-1.96355	-1.96354	-1.96356
11.90033	-1.93615	-1.93614	-1.93616
11.90867	-1.90965	-1.90964	-1.90966
11.917	-1.88495	-1.88494	-1.88496
11.92533	-1.85935	-1.85934	-1.85936
11.93367	-1.8308	-1.83079	-1.83081
11.942	-1.8072	-1.80719	-1.80721
11.95033	-1.7796	-1.77959	-1.77961
11.95867	-1.74865	-1.74864	-1.74866
11.967	-1.71805	-1.71804	-1.71806
11.97533	-1.6832	-1.68319	-1.68321
11.98367	-1.6487	-1.64869	-1.64871
11.992	-1.61955	-1.61954	-1.61956
12.00033	-1.59365	-1.59364	-1.59366
12.00867	-1.567	-1.56699	-1.56701
12.017	-1.5476	-1.54759	-1.54761
12.02533	-1.53035	-1.53034	-1.53036
12.03367	-1.51605	-1.51604	-1.51606
12.042	-1.5113	-1.51129	-1.51131
12.05033	-1.5095	-1.50949	-1.50951
12.05867	-1.50665	-1.50664	-1.50666

12.067	-1.4996	-1.49959	-1.49961
12.07533	-1.4787	-1.47869	-1.47871
12.08367	-1.4421	-1.44209	-1.44211
12.092	-1.4052	-1.40519	-1.40521
12.10033	-1.3702	-1.37019	-1.37021
12.10867	-1.3366	-1.33659	-1.33661
12.117	-1.3008	-1.30079	-1.30081
12.12533	-1.2666	-1.26659	-1.26661
12.13367	-1.2307	-1.23069	-1.23071
12.142	-1.19395	-1.19394	-1.19396
12.15033	-1.156	-1.15599	-1.15601
12.15867	-1.11845	-1.11844	-1.11846
12.167	-1.0819	-1.08189	-1.08191
12.17533	-1.0407	-1.04069	-1.04071
12.18367	-1.0089	-1.00889	-1.00891
12.192	-0.9763	-0.97629	-0.97631
12.20033	-0.9496	-0.94959	-0.94961
12.20867	-0.92495	-0.92494	-0.92496
12.217	-0.9029	-0.90289	-0.90291
12.22533	-0.88685	-0.88684	-0.88686
12.23367	-0.88145	-0.88144	-0.88146
12.242	-0.87115	-0.87114	-0.87116
12.25033	-0.8617	-0.86169	-0.86171
12.25867	-0.83995	-0.83994	-0.83996
12.267	-0.80025	-0.80024	-0.80026
12.27533	-0.7573	-0.75729	-0.75731
12.28367	-0.716	-0.71599	-0.71601
12.292	-0.6759	-0.67589	-0.67591
12.30033	-0.637	-0.63699	-0.63701
12.30867	-0.5977	-0.59769	-0.59771
12.317	-0.56245	-0.56244	-0.56246
12.32533	-0.52235	-0.52234	-0.52236
12.33367	-0.48245	-0.48244	-0.48246
12.342	-0.4419	-0.44189	-0.44191
12.35033	-0.39715	-0.39714	-0.39716
12.35867	-0.35485	-0.35484	-0.35486
12.367	-0.31945	-0.31944	-0.31946
12.37533	-0.28245	-0.28244	-0.28246
12.38367	-0.2492	-0.24919	-0.24921
12.392	-0.22055	-0.22054	-0.22056
12.40033	-0.19685	-0.19684	-0.19686
12.40867	-0.1761	-0.17609	-0.17611
12.417	-0.1645	-0.16449	-0.16451
12.42533	-0.1532	-0.15319	-0.15321

12.43367	-0.1412	-0.14119	-0.14121
12.442	-0.12115	-0.12114	-0.12116
12.45033	-0.08485	-0.08484	-0.08486
12.45867	-0.0385	-0.03849	-0.03851
12.467	0.00325	0.00326	0.00324
12.47533	0.043	0.04301	0.04299
12.48367	0.08375	0.08376	0.08374
12.492	0.12405	0.12406	0.12404
12.50033	0.16305	0.16306	0.16304
12.50867	0.2012	0.20121	0.20119
12.517	0.2408	0.24081	0.24079
12.52533	0.2839	0.28391	0.28389
12.53367	0.32505	0.32506	0.32504
12.542	0.3641	0.36411	0.36409
12.55033	0.40285	0.40286	0.40284
12.55867	0.4355	0.43551	0.43549
12.567	0.4707	0.47071	0.47069
12.57533	0.4997	0.49971	0.49969
12.58367	0.52475	0.52476	0.52474
12.592	0.5479	0.54791	0.54789
12.60033	0.563	0.56301	0.56299
12.60867	0.5735	0.57351	0.57349
12.617	0.58805	0.58806	0.58804
12.62533	0.6034	0.60341	0.60339
12.63367	0.63425	0.63426	0.63424
12.642	0.67815	0.67816	0.67814
12.65033	0.7221	0.72211	0.72209
12.65867	0.76285	0.76286	0.76284
12.667	0.8037	0.80371	0.80369
12.67533	0.8425	0.84251	0.84249
12.68367	0.88125	0.88126	0.88124
12.692	0.917	0.91701	0.91699
12.70033	0.95925	0.95926	0.95924
12.70867	0.9978	0.99781	0.99779
12.717	1.03755	1.03756	1.03754
12.72533	1.08105	1.08106	1.08104
12.73367	1.12355	1.12356	1.12354
12.742	1.15615	1.15616	1.15614
12.75033	1.19285	1.19286	1.19284
12.75867	1.22545	1.22546	1.22544
12.767	1.25235	1.25236	1.25234
12.77533	1.2763	1.27631	1.27629
12.78367	1.29535	1.29536	1.29534
12.792	1.30665	1.30666	1.30664

12.80033	1.31985	1.31986	1.31984
12.80867	1.33545	1.33546	1.33544
12.817	1.3581	1.35811	1.35809
12.82533	1.399	1.39901	1.39899
12.83367	1.44395	1.44396	1.44394
12.842	1.48435	1.48436	1.48434
12.85033	1.52185	1.52186	1.52184
12.85867	1.5589	1.55891	1.55889
12.867	1.59585	1.59586	1.59584
12.87533	1.6312	1.63121	1.63119
12.88367	1.66555	1.66556	1.66554
12.892	1.6989	1.69891	1.69889
12.90033	1.7339	1.73391	1.73389
12.90867	1.76935	1.76936	1.76934
12.917	1.80315	1.80316	1.80314
12.92533	1.83205	1.83206	1.83204
12.93367	1.85625	1.85626	1.85624
12.942	1.8798	1.87981	1.87979
12.95033	1.896	1.89601	1.89599
12.95867	1.9114	1.91141	1.91139
12.967	1.92175	1.92176	1.92174
12.97533	1.92415	1.92416	1.92414
12.98367	1.92215	1.92216	1.92214
12.992	1.9239	1.92391	1.92389
13.00033	1.92855	1.92856	1.92854
13.00867	1.9494	1.94941	1.94939
13.017	1.9794	1.97941	1.97939
13.02533	2.00575	2.00576	2.00574
13.03367	2.029	2.02901	2.02899
13.042	2.0502	2.05021	2.05019
13.05033	2.07295	2.07296	2.07294
13.05867	2.0917	2.09171	2.09169
13.067	2.11065	2.11066	2.11064
13.07533	2.131	2.13101	2.13099
13.08367	2.1512	2.15121	2.15119
13.092	2.17315	2.17316	2.17314
13.10033	2.1931	2.19311	2.19309
13.10867	2.2125	2.21251	2.21249
13.117	2.22755	2.22756	2.22754
13.12533	2.2418	2.24181	2.24179
13.13367	2.24985	2.24986	2.24984
13.142	2.2545	2.25451	2.25449
13.15033	2.25855	2.25856	2.25854
13.15867	2.25415	2.25416	2.25414

13.167	2.2461	2.24611	2.24609
13.17533	2.2397	2.23971	2.23969
13.18367	2.2367	2.23671	2.23669
13.192	2.2446	2.24461	2.24459
13.20033	2.26995	2.26996	2.26994
13.20867	2.2954	2.29541	2.29539
13.217	2.3177	2.31771	2.31769
13.22533	2.33605	2.33606	2.33604
13.23367	2.3545	2.35451	2.35449
13.242	2.3728	2.37281	2.37279
13.25033	2.3916	2.39161	2.39159
13.25867	2.4118	2.41181	2.41179
13.267	2.43215	2.43216	2.43214
13.27533	2.4505	2.45051	2.45049
13.28367	2.4705	2.47051	2.47049
13.292	2.49165	2.49166	2.49164
13.30033	2.50595	2.50596	2.50594
13.30867	2.51775	2.51776	2.51774
13.317	2.5304	2.53041	2.53039
13.32533	2.5367	2.53671	2.53669
13.33367	2.5394	2.53941	2.53939
13.342	2.54015	2.54016	2.54014
13.35033	2.53175	2.53176	2.53174
13.35867	2.5259	2.52591	2.52589
13.367	2.52095	2.52096	2.52094
13.37533	2.5255	2.52551	2.52549
13.38367	2.5443	2.54431	2.54429
13.392	2.5712	2.57121	2.57119
13.40033	2.5923	2.59231	2.59229
13.40867	2.61295	2.61296	2.61294
13.417	2.6321	2.63211	2.63209
13.42533	2.65125	2.65126	2.65124
13.43367	2.66825	2.66826	2.66824
13.442	2.68795	2.68796	2.68794
13.45033	2.70485	2.70486	2.70484
13.45867	2.72435	2.72436	2.72434
13.467	2.7467	2.74671	2.74669
13.47533	2.7661	2.76611	2.76609
13.48367	2.7787	2.77871	2.77869
13.492	2.78825	2.78826	2.78824
13.50033	2.80095	2.80096	2.80094
13.50867	2.80795	2.80796	2.80794
13.517	2.81005	2.81006	2.81004
13.52533	2.81225	2.81226	2.81224

13.53367	2.80385	2.80386	2.80384
13.542	2.79435	2.79436	2.79434
13.55033	2.7876	2.78761	2.78759
13.55867	2.7836	2.78361	2.78359
13.567	2.7955	2.79551	2.79549
13.57533	2.82005	2.82006	2.82004
13.58367	2.8425	2.84251	2.84249
13.592	2.86135	2.86136	2.86134
13.60033	2.8792	2.87921	2.87919
13.60867	2.89605	2.89606	2.89604
13.617	2.91245	2.91246	2.91244
13.62533	2.92295	2.92296	2.92294
13.63367	2.9406	2.94061	2.94059
13.642	2.9607	2.96071	2.96069
13.65033	2.9781	2.97811	2.97809
13.65867	2.99325	2.99326	2.99324
13.667	3.01185	3.01186	3.01184
13.67533	3.0207	3.02071	3.02069
13.68367	3.03245	3.03246	3.03244
13.692	3.0386	3.03861	3.03859
13.70033	3.0417	3.04171	3.04169
13.70867	3.04055	3.04056	3.04054
13.717	3.03545	3.03546	3.03544
13.72533	3.022	3.02201	3.02199
13.73367	3.01345	3.01346	3.01344
13.742	3.0058	3.00581	3.00579
13.75033	3.01005	3.01006	3.01004
13.75867	3.0292	3.02921	3.02919
13.767	3.05335	3.05336	3.05334
13.77533	3.07155	3.07156	3.07154
13.78367	3.0866	3.08661	3.08659
13.792	3.1008	3.10081	3.10079
13.80033	3.1148	3.11481	3.11479
13.80867	3.12825	3.12826	3.12824
13.817	3.14545	3.14546	3.14544
13.82533	3.15845	3.15846	3.15844
13.83367	3.1732	3.17321	3.17319
13.842	3.1918	3.19181	3.19179
13.85033	3.20645	3.20646	3.20644
13.85867	3.21465	3.21466	3.21464
13.867	3.22495	3.22496	3.22494
13.87533	3.2311	3.23111	3.23109
13.88367	3.232	3.23201	3.23199
13.892	3.22915	3.22916	3.22914

13.90033	3.2252	3.22521	3.22519
13.90867	3.21235	3.21236	3.21234
13.917	3.19935	3.19936	3.19934
13.92533	3.1889	3.18891	3.18889
13.93367	3.18425	3.18426	3.18424
13.942	3.19765	3.19766	3.19764
13.95033	3.21805	3.21806	3.21804
13.95867	3.23825	3.23826	3.23824
13.967	3.2508	3.25081	3.25079
13.97533	3.2661	3.26611	3.26609
13.98367	3.27955	3.27956	3.27954
13.992	3.29215	3.29216	3.29214
14.00033	3.30135	3.30136	3.30134
14.00867	3.31575	3.31576	3.31574
14.017	3.3322	3.33221	3.33219
14.02533	3.3456	3.34561	3.34559
14.03367	3.3583	3.35831	3.35829
14.042	3.37175	3.37176	3.37174
14.05033	3.37605	3.37606	3.37604
14.05867	3.38585	3.38586	3.38584
14.067	3.387	3.38701	3.38699
14.07533	3.3854	3.38541	3.38539
14.08367	3.3822	3.38221	3.38219
14.092	3.37015	3.37016	3.37014
14.10033	3.35455	3.35456	3.35454
14.10867	3.34285	3.34286	3.34284
14.117	3.33355	3.33356	3.33354
14.12533	3.3386	3.33861	3.33859
14.13367	3.3581	3.35811	3.35809
14.142	3.3753	3.37531	3.37529
14.15033	3.3894	3.38941	3.38939
14.15867	3.4015	3.40151	3.40149
14.167	3.4162	3.41621	3.41619
14.17533	3.4259	3.42591	3.42589
14.18367	3.43505	3.43506	3.43504
14.192	3.44795	3.44796	3.44794
14.20033	3.46025	3.46026	3.46024
14.20867	3.4746	3.47461	3.47459
14.217	3.4895	3.48951	3.48949
14.22533	3.50195	3.50196	3.50194
14.23367	3.50725	3.50726	3.50724
14.242	3.5138	3.51381	3.51379
14.25033	3.5154	3.51541	3.51539
14.25867	3.51305	3.51306	3.51304

14.267	3.5086	3.50861	3.50859
14.27533	3.49695	3.49696	3.49694
14.28367	3.47895	3.47896	3.47894
14.292	3.46435	3.46436	3.46434
14.30033	3.4521	3.45211	3.45209
14.30867	3.449	3.44901	3.44899
14.317	3.4657	3.46571	3.46569
14.32533	3.4839	3.48391	3.48389
14.33367	3.49695	3.49696	3.49694
14.342	3.50735	3.50736	3.50734
14.35033	3.51825	3.51826	3.51824
14.35867	3.53135	3.53136	3.53134
14.367	3.5367	3.53671	3.53669
14.37533	3.546	3.54601	3.54599
14.38367	3.55845	3.55846	3.55844
14.392	3.57015	3.57016	3.57014
14.40033	3.58385	3.58386	3.58384
14.40867	3.59795	3.59796	3.59794
14.417	3.6037	3.60371	3.60369
14.42533	3.6083	3.60831	3.60829
14.43367	3.61205	3.61206	3.61204
14.442	3.6106	3.61061	3.61059
14.45033	3.60575	3.60576	3.60574
14.45867	3.5993	3.59931	3.59929
14.467	3.57995	3.57996	3.57994
14.47533	3.56275	3.56276	3.56274
14.48367	3.5465	3.54651	3.54649
14.492	3.5374	3.53741	3.53739
14.50033	3.5449	3.54491	3.54489
14.50867	3.5641	3.56411	3.56409
14.517	3.57805	3.57806	3.57804
14.52533	3.59	3.59001	3.58999
14.53367	3.59925	3.59926	3.59924
14.542	3.61005	3.61006	3.61004
14.55033	3.6192	3.61921	3.61919
14.55867	3.6286	3.62861	3.62859
14.567	3.6385	3.63851	3.63849
14.57533	3.65185	3.65186	3.65184
14.58367	3.66435	3.66436	3.66434
14.592	3.6725	3.67251	3.67249
14.60033	3.6819	3.68191	3.68189
14.60867	3.68475	3.68476	3.68474
14.617	3.6881	3.68811	3.68809
14.62533	3.6866	3.68661	3.68659

14.63367	3.6805	3.68051	3.68049
14.642	3.67335	3.67336	3.67334
14.65033	3.6587	3.65871	3.65869
14.65867	3.63735	3.63736	3.63734
14.667	3.6223	3.62231	3.62229
14.67533	3.6075	3.60751	3.60749
14.68367	3.6078	3.60781	3.60779
14.692	3.62435	3.62436	3.62434
14.70033	3.64195	3.64196	3.64194
14.70867	3.65175	3.65176	3.65174
14.717	3.66065	3.66066	3.66064
14.72533	3.6693	3.66931	3.66929
14.73367	3.6794	3.67941	3.67939
14.742	3.68265	3.68266	3.68264
14.75033	3.69495	3.69496	3.69494
14.75867	3.70595	3.70596	3.70594
14.767	3.714	3.71401	3.71399
14.77533	3.7266	3.72661	3.72659
14.78367	3.74065	3.74066	3.74064
14.792	3.742	3.74201	3.74199
14.80033	3.7467	3.74671	3.74669
14.80867	3.7452	3.74521	3.74519
14.817	3.74215	3.74216	3.74214
14.82533	3.73555	3.73556	3.73554
14.83367	3.7258	3.72581	3.72579
14.842	3.70405	3.70406	3.70404
14.85033	3.6866	3.68661	3.68659
14.85867	3.6711	3.67111	3.67109
14.867	3.66585	3.66586	3.66584
14.87533	3.67955	3.67956	3.67954
14.88367	3.698	3.69801	3.69799
14.892	3.7127	3.71271	3.71269
14.90033	3.72395	3.72396	3.72394
14.90867	3.73705	3.73706	3.73704
14.917	3.74835	3.74836	3.74834
14.92533	3.7608	3.76081	3.76079
14.93367	3.7726	3.77261	3.77259
14.942	3.7836	3.78361	3.78359
14.95033	3.7998	3.79981	3.79979
14.95867	3.8145	3.81451	3.81449
14.967	3.8248	3.82481	3.82479
14.97533	3.83555	3.83556	3.83554
14.98367	3.8407	3.84071	3.84069
14.992	3.84835	3.84836	3.84834

15.00033	3.8481	3.84811	3.84809
15.00867	3.8461	3.84611	3.84609
15.017	3.84285	3.84286	3.84284
15.02533	3.82875	3.82876	3.82874
15.03367	3.8149	3.81491	3.81489
15.042	3.80435	3.80436	3.80434
15.05033	3.79865	3.79866	3.79864
15.05867	3.8132	3.81321	3.81319
15.067	3.8363	3.83631	3.83629
15.07533	3.8586	3.85861	3.85859
15.08367	3.87695	3.87696	3.87694
15.092	3.89455	3.89456	3.89454
15.10033	3.9119	3.91191	3.91189
15.10867	3.9263	3.92631	3.92629
15.117	3.9396	3.93961	3.93959
15.12533	3.95865	3.95866	3.95864
15.13367	3.9783	3.97831	3.97829
15.142	3.99645	3.99646	3.99644
15.15033	4.0168	4.01681	4.01679
15.15867	4.03465	4.03466	4.03464
15.167	4.04425	4.04426	4.04424
15.17533	4.0572	4.05721	4.05719
15.18367	4.0618	4.06181	4.06179
15.192	4.0637	4.06371	4.06369
15.20033	4.0637	4.06371	4.06369
15.20867	4.05545	4.05546	4.05544
15.217	4.0431	4.04311	4.04309
15.22533	4.0333	4.03331	4.03329
15.23367	4.02635	4.02636	4.02634
15.242	4.0312	4.03121	4.03119
15.25033	4.05265	4.05266	4.05264
15.25867	4.072	4.07201	4.07199
15.267	4.08805	4.08806	4.08804
15.27533	4.09985	4.09986	4.09984
15.28367	4.11145	4.11146	4.11144
15.292	4.12175	4.12176	4.12174
15.30033	4.1319	4.13191	4.13189
15.30867	4.1413	4.14131	4.14129
15.317	4.1501	4.15011	4.15009
15.32533	4.16025	4.16026	4.16024
15.33367	4.16855	4.16856	4.16854
15.342	4.1744	4.17441	4.17439
15.35033	4.174	4.17401	4.17399
15.35867	4.17285	4.17286	4.17284

15.367	4.16915	4.16916	4.16914
15.37533	4.1595	4.15951	4.15949
15.38367	4.14585	4.14586	4.14584
15.392	4.12715	4.12716	4.12714
15.40033	4.0984	4.09841	4.09839
15.40867	4.07215	4.07216	4.07214
15.417	4.04655	4.04656	4.04654
15.42533	4.0279	4.02791	4.02789
15.43367	4.0263	4.02631	4.02629
15.442	4.03175	4.03176	4.03174
15.45033	4.03175	4.03176	4.03174
15.45867	4.02995	4.02996	4.02994
15.467	4.02575	4.02576	4.02574
15.47533	4.0244	4.02441	4.02439
15.48367	4.0145	4.01451	4.01449
15.492	4.00735	4.00736	4.00734
15.50033	4.0062	4.00621	4.00619
15.50867	4.0039	4.00391	4.00389
15.517	4.0038	4.00381	4.00379
15.52533	4.0014	4.00141	4.00139
15.53367	3.9945	3.99451	3.99449
15.542	3.98545	3.98546	3.98544
15.55033	3.9766	3.97661	3.97659
15.55867	3.96105	3.96106	3.96104
15.567	3.94185	3.94186	3.94184
15.57533	3.9217	3.92171	3.92169
15.58367	3.8903	3.89031	3.89029
15.592	3.8578	3.85781	3.85779
15.60033	3.8286	3.82861	3.82859
15.60867	3.8045	3.80451	3.80449
15.617	3.79595	3.79596	3.79594
15.62533	3.80215	3.80216	3.80214
15.63367	3.80675	3.80676	3.80674
15.642	3.80725	3.80726	3.80724
15.65033	3.8055	3.80551	3.80549
15.65867	3.8058	3.80581	3.80579
15.667	3.80665	3.80666	3.80664
15.67533	3.80605	3.80606	3.80604
15.68367	3.8102	3.81021	3.81019
15.692	3.81505	3.81506	3.81504
15.70033	3.8163	3.81631	3.81629
15.70867	3.8178	3.81781	3.81779
15.717	3.82305	3.82306	3.82304
15.72533	3.8186	3.81861	3.81859

15.73367	3.8172	3.81721	3.81719
15.742	3.8091	3.80911	3.80909
15.75033	3.7996	3.79961	3.79959
15.75867	3.7854	3.78541	3.78539
15.767	3.76705	3.76706	3.76704
15.77533	3.73905	3.73906	3.73904
15.78367	3.71615	3.71616	3.71614
15.792	3.6943	3.69431	3.69429
15.80033	3.68615	3.68616	3.68614
15.80867	3.6942	3.69421	3.69419
15.817	3.70695	3.70696	3.70694
15.82533	3.71285	3.71286	3.71284
15.83367	3.71955	3.71956	3.71954
15.842	3.7247	3.72471	3.72469
15.85033	3.7302	3.73021	3.73019
15.85867	3.7301	3.73011	3.73009
15.867	3.73555	3.73556	3.73554
15.87533	3.7393	3.73931	3.73929
15.88367	3.7475	3.74751	3.74749
15.892	3.75885	3.75886	3.75884
15.90033	3.76395	3.76396	3.76394
15.90867	3.76215	3.76216	3.76214
15.917	3.7639	3.76391	3.76389
15.92533	3.7602	3.76021	3.76019
15.93367	3.75075	3.75076	3.75074
15.942	3.7393	3.73931	3.73929
15.95033	3.7265	3.72651	3.72649
15.95867	3.6997	3.69971	3.69969
15.967	3.67575	3.67576	3.67574
15.97533	3.6546	3.65461	3.65459
15.98367	3.63945	3.63946	3.63944
15.992	3.64515	3.64516	3.64514
16.00033	3.66025	3.66026	3.66024
16.00867	3.671	3.67101	3.67099
16.017	3.6752	3.67521	3.67519
16.02533	3.6812	3.68121	3.68119
16.03367	3.6862	3.68621	3.68619
16.042	3.68675	3.68676	3.68674
16.05033	3.6854	3.68541	3.68539
16.05867	3.6944	3.69441	3.69439
16.067	3.70375	3.70376	3.70374
16.07533	3.70905	3.70906	3.70904
16.08367	3.7144	3.71441	3.71439
16.092	3.72235	3.72236	3.72234

16.10033	3.7178	3.71781	3.71779
16.10867	3.7207	3.72071	3.72069
16.117	3.7111	3.71111	3.71109
16.12533	3.70135	3.70136	3.70134
16.13367	3.6886	3.68861	3.68859
16.142	3.66545	3.66546	3.66544
16.15033	3.6397	3.63971	3.63969
16.15867	3.6178	3.61781	3.61779
16.167	3.5988	3.59881	3.59879
16.17533	3.5964	3.59641	3.59639
16.18367	3.60935	3.60936	3.60934
16.192	3.6204	3.62041	3.62039
16.20033	3.6255	3.62551	3.62549
16.20867	3.62825	3.62826	3.62824
16.217	3.6349	3.63491	3.63489
16.22533	3.6383	3.63831	3.63829
16.23367	3.6405	3.64051	3.64049
16.242	3.6466	3.64661	3.64659
16.25033	3.6509	3.65091	3.65089
16.25867	3.6583	3.65831	3.65829
16.267	3.66805	3.66806	3.66804
16.27533	3.6741	3.67411	3.67409
16.28367	3.67195	3.67196	3.67194
16.292	3.67405	3.67406	3.67404
16.30033	3.6679	3.66791	3.66789
16.30867	3.65805	3.65806	3.65804
16.317	3.64785	3.64786	3.64784
16.32533	3.63055	3.63056	3.63054
16.33367	3.6037	3.60371	3.60369
16.342	3.5804	3.58041	3.58039
16.35033	3.56075	3.56076	3.56074
16.35867	3.5491	3.54911	3.54909
16.367	3.55965	3.55966	3.55964
16.37533	3.572	3.57201	3.57199
16.38367	3.5818	3.58181	3.58179
16.392	3.58685	3.58686	3.58684
16.40033	3.59325	3.59326	3.59324
16.40867	3.60115	3.60116	3.60114
16.417	3.59875	3.59876	3.59874
16.42533	3.60165	3.60166	3.60164
16.43367	3.61165	3.61166	3.61164
16.442	3.62345	3.62346	3.62344
16.45033	3.63155	3.63156	3.63154
16.45867	3.6394	3.63941	3.63939

16.467	3.6426	3.64261	3.64259
16.47533	3.6413	3.64131	3.64129
16.48367	3.64235	3.64236	3.64234
16.492	3.6347	3.63471	3.63469
16.50033	3.62495	3.62496	3.62494
16.50867	3.61295	3.61296	3.61294
16.517	3.58685	3.58686	3.58684
16.52533	3.56495	3.56496	3.56494
16.53367	3.54345	3.54346	3.54344
16.542	3.5291	3.52911	3.52909
16.55033	3.53365	3.53366	3.53364
16.55867	3.5497	3.54971	3.54969
16.567	3.558	3.55801	3.55799
16.57533	3.5653	3.56531	3.56529
16.58367	3.5685	3.56851	3.56849
16.592	3.57495	3.57496	3.57494
16.60033	3.57835	3.57836	3.57834
16.60867	3.58295	3.58296	3.58294
16.617	3.59085	3.59086	3.59084
16.62533	3.59875	3.59876	3.59874
16.63367	3.6078	3.60781	3.60779
16.642	3.6153	3.61531	3.61529
16.65033	3.6218	3.62181	3.62179
16.65867	3.6202	3.62021	3.62019
16.667	3.62385	3.62386	3.62384
16.67533	3.6186	3.61861	3.61859
16.68367	3.60885	3.60886	3.60884
16.692	3.59775	3.59776	3.59774
16.70033	3.5761	3.57611	3.57609
16.70867	3.5514	3.55141	3.55139
16.717	3.53015	3.53016	3.53014
16.72533	3.5125	3.51251	3.51249
16.73367	3.5092	3.50921	3.50919
16.742	3.5223	3.52231	3.52229
16.75033	3.5346	3.53461	3.53459
16.75867	3.54005	3.54006	3.54004
16.767	3.5474	3.54741	3.54739
16.77533	3.553	3.55301	3.55299
16.78367	3.55885	3.55886	3.55884
16.792	3.557	3.55701	3.55699
16.80033	3.5637	3.56371	3.56369
16.80867	3.5715	3.57151	3.57149
16.817	3.5793	3.57931	3.57929
16.82533	3.5902	3.59021	3.59019

16.83367	3.599	3.59901	3.59899
16.842	3.5971	3.59711	3.59709
16.85033	3.5982	3.59821	3.59819
16.85867	3.59555	3.59556	3.59554
16.867	3.58825	3.58826	3.58824
16.87533	3.57765	3.57766	3.57764
16.88367	3.5624	3.56241	3.56239
16.892	3.53495	3.53496	3.53494
16.90033	3.51345	3.51346	3.51344
16.90867	3.49145	3.49146	3.49144
16.917	3.4819	3.48191	3.48189
16.92533	3.49165	3.49166	3.49164
16.93367	3.5068	3.50681	3.50679
16.942	3.5147	3.51471	3.51469
16.95033	3.52105	3.52106	3.52104
16.95867	3.52685	3.52686	3.52684
16.967	3.53385	3.53386	3.53384
16.97533	3.53525	3.53526	3.53524
16.98367	3.53885	3.53886	3.53884
16.992	3.5474	3.54741	3.54739
17.00033	3.55835	3.55836	3.55834
17.00867	3.56845	3.56846	3.56844
17.017	3.5727	3.57271	3.57269
17.02533	3.57795	3.57796	3.57794
17.03367	3.5792	3.57921	3.57919
17.042	3.58165	3.58166	3.58164
17.05033	3.5729	3.57291	3.57289
17.05867	3.56225	3.56226	3.56224
17.067	3.54985	3.54986	3.54984
17.07533	3.5251	3.52511	3.52509
17.08367	3.50265	3.50266	3.50264
17.092	3.48445	3.48446	3.48444
17.10033	3.4689	3.46891	3.46889
17.10867	3.47195	3.47196	3.47194
17.117	3.48435	3.48436	3.48434
17.12533	3.49545	3.49546	3.49544
17.13367	3.49925	3.49926	3.49924
17.142	3.50505	3.50506	3.50504
17.15033	3.5105	3.51051	3.51049
17.15867	3.51285	3.51286	3.51284
17.167	3.51335	3.51336	3.51334
17.17533	3.5239	3.52391	3.52389
17.18367	3.53165	3.53166	3.53164
17.192	3.53825	3.53826	3.53824

17.20033	3.54985	3.54986	3.54984
17.20867	3.5592	3.55921	3.55919
17.217	3.55515	3.55516	3.55514
17.22533	3.55845	3.55846	3.55844
17.23367	3.5521	3.55211	3.55209
17.242	3.5422	3.54221	3.54219
17.25033	3.5329	3.53291	3.53289
17.25867	3.5127	3.51271	3.51269
17.267	3.48805	3.48806	3.48804
17.27533	3.46565	3.46566	3.46564
17.28367	3.44585	3.44586	3.44584
17.292	3.4408	3.44081	3.44079
17.30033	3.4528	3.45281	3.45279
17.30867	3.4631	3.46311	3.46309
17.317	3.47055	3.47056	3.47054
17.32533	3.4746	3.47461	3.47459
17.33367	3.48195	3.48196	3.48194
17.342	3.48735	3.48736	3.48734
17.35033	3.4899	3.48991	3.48989
17.35867	3.49315	3.49316	3.49314
17.367	3.5005	3.50051	3.50049
17.37533	3.5121	3.51211	3.51209
17.38367	3.5208	3.52081	3.52079
17.392	3.52615	3.52616	3.52614
17.40033	3.52635	3.52636	3.52634
17.40867	3.5273	3.52731	3.52729
17.417	3.5258	3.52581	3.52579
17.42533	3.51505	3.51506	3.51504
17.43367	3.5055	3.50551	3.50549
17.442	3.4913	3.49131	3.49129
17.45033	3.46505	3.46506	3.46504
17.45867	3.4425	3.44251	3.44249
17.467	3.4215	3.42151	3.42149
17.47533	3.40855	3.40856	3.40854
17.48367	3.4171	3.41711	3.41709
17.492	3.43025	3.43026	3.43024
17.50033	3.4386	3.43861	3.43859
17.50867	3.4438	3.44381	3.44379
17.517	3.4475	3.44751	3.44749
17.52533	3.45325	3.45326	3.45324
17.53367	3.45085	3.45086	3.45084
17.542	3.454	3.45401	3.45399
17.55033	3.4649	3.46491	3.46489
17.55867	3.4722	3.47221	3.47219

17.567	3.48025	3.48026	3.48024
17.57533	3.4895	3.48951	3.48949
17.58367	3.4934	3.49341	3.49339
17.592	3.4905	3.49051	3.49049
17.60033	3.4923	3.49231	3.49229
17.60867	3.4844	3.48441	3.48439
17.617	3.47405	3.47406	3.47404
17.62533	3.4629	3.46291	3.46289
17.63367	3.4368	3.43681	3.43679
17.642	3.41495	3.41496	3.41494
17.65033	3.39195	3.39196	3.39194
17.65867	3.3756	3.37561	3.37559
17.667	3.37635	3.37636	3.37634
17.67533	3.3907	3.39071	3.39069
17.68367	3.39895	3.39896	3.39894
17.692	3.4055	3.40551	3.40549
17.70033	3.4108	3.41081	3.41079
17.70867	3.4174	3.41741	3.41739
17.717	3.42035	3.42036	3.42034
17.72533	3.42365	3.42366	3.42364
17.73367	3.4299	3.42991	3.42989
17.742	3.4369	3.43691	3.43689
17.75033	3.4472	3.44721	3.44719
17.75867	3.4553	3.45531	3.45529
17.767	3.4612	3.46121	3.46119
17.77533	3.4586	3.45861	3.45859
17.78367	3.46035	3.46036	3.46034
17.792	3.4562	3.45621	3.45619
17.80033	3.44645	3.44646	3.44644
17.80867	3.4364	3.43641	3.43639
17.817	3.41905	3.41906	3.41904
17.82533	3.3934	3.39341	3.39339
17.83367	3.37145	3.37146	3.37144
17.842	3.3507	3.35071	3.35069
17.85033	3.34525	3.34526	3.34524
17.85867	3.3571	3.35711	3.35709
17.867	3.37185	3.37186	3.37184
17.87533	3.37855	3.37856	3.37854
17.88367	3.38605	3.38606	3.38604
17.892	3.39215	3.39216	3.39214
17.90033	3.39895	3.39896	3.39894
17.90867	3.39655	3.39656	3.39654
17.917	3.4027	3.40271	3.40269
17.92533	3.4112	3.41121	3.41119

17.93367	3.42005	3.42006	3.42004
17.942	3.43105	3.43106	3.43104
17.95033	3.4386	3.43861	3.43859
17.95867	3.4397	3.43971	3.43969
17.967	3.44365	3.44366	3.44364
17.97533	3.44435	3.44436	3.44434
17.98367	3.43665	3.43666	3.43664
17.992	3.4263	3.42631	3.42629
18.00033	3.41445	3.41446	3.41444
18.00867	3.38605	3.38606	3.38604
18.017	3.36485	3.36486	3.36484
18.02533	3.34615	3.34616	3.34614
18.03367	3.33505	3.33506	3.33504
18.042	3.34375	3.34376	3.34374
18.05033	3.35755	3.35756	3.35754
18.05867	3.36555	3.36556	3.36554
18.067	3.36975	3.36976	3.36974
18.07533	3.3767	3.37671	3.37669
18.08367	3.3828	3.38281	3.38279
18.092	3.38615	3.38616	3.38614
18.10033	3.39015	3.39016	3.39014
18.10867	3.4006	3.40061	3.40059
18.117	3.40965	3.40966	3.40964
18.12533	3.4177	3.41771	3.41769
18.13367	3.42845	3.42846	3.42844
18.142	3.4373	3.43731	3.43729
18.15033	3.43395	3.43396	3.43394
18.15867	3.43525	3.43526	3.43524
18.167	3.428	3.42801	3.42799
18.17533	3.4191	3.41911	3.41909
18.18367	3.40895	3.40896	3.40894
18.192	3.386	3.38601	3.38599
18.20033	3.36175	3.36176	3.36174
18.20867	3.34225	3.34226	3.34224
18.217	3.3253	3.32531	3.32529
18.22533	3.3273	3.32731	3.32729
18.23367	3.3401	3.34011	3.34009
18.242	3.3514	3.35141	3.35139
18.25033	3.3575	3.35751	3.35749
18.25867	3.363	3.36301	3.36299
18.267	3.37115	3.37116	3.37114
18.27533	3.3771	3.37711	3.37709
18.28367	3.38305	3.38306	3.38304
18.292	3.3904	3.39041	3.39039

18.30033	3.3948	3.39481	3.39479
18.30867	3.4048	3.40481	3.40479
18.317	3.4158	3.41581	3.41579
18.32533	3.4203	3.42031	3.42029
18.33367	3.41985	3.41986	3.41984
18.342	3.42435	3.42436	3.42434
18.35033	3.4226	3.42261	3.42259
18.35867	3.41275	3.41276	3.41274
18.367	3.4036	3.40361	3.40359
18.37533	3.3865	3.38651	3.38649
18.38367	3.35995	3.35996	3.35994
18.392	3.33815	3.33816	3.33814
18.40033	3.3192	3.31921	3.31919
18.40867	3.3102	3.31021	3.31019
18.417	3.32345	3.32346	3.32344
18.42533	3.3356	3.33561	3.33559
18.43367	3.34245	3.34246	3.34244
18.442	3.34715	3.34716	3.34714
18.45033	3.3532	3.35321	3.35319
18.45867	3.35895	3.35896	3.35894
18.467	3.35905	3.35906	3.35904
18.47533	3.36355	3.36356	3.36354
18.48367	3.37605	3.37606	3.37604
18.492	3.38515	3.38516	3.38514
18.50033	3.39145	3.39146	3.39144
18.50867	3.39965	3.39966	3.39964
18.517	3.40495	3.40496	3.40494
18.52533	3.40255	3.40256	3.40254
18.53367	3.40405	3.40406	3.40404
18.542	3.39495	3.39496	3.39494
18.55033	3.3843	3.38431	3.38429
18.55867	3.37155	3.37156	3.37154
18.567	3.3443	3.34431	3.34429
18.57533	3.3239	3.32391	3.32389
18.58367	3.3025	3.30251	3.30249
18.592	3.28865	3.28866	3.28864
18.60033	3.2931	3.29311	3.29309
18.60867	3.3074	3.30741	3.30739
18.617	3.31515	3.31516	3.31514
18.62533	3.3205	3.32051	3.32049
18.63367	3.3254	3.32541	3.32539
18.642	3.33335	3.33336	3.33334
18.65033	3.33635	3.33636	3.33634
18.65867	3.3442	3.34421	3.34419

18.667	3.353	3.35301	3.35299
18.67533	3.3577	3.35771	3.35769
18.68367	3.3658	3.36581	3.36579
18.692	3.37745	3.37746	3.37744
18.70033	3.3814	3.38141	3.38139
18.70867	3.37735	3.37736	3.37734
18.717	3.37835	3.37836	3.37834
18.72533	3.3731	3.37311	3.37309
18.73367	3.3619	3.36191	3.36189
18.742	3.3515	3.35151	3.35149
18.75033	3.32865	3.32866	3.32864
18.75867	3.30435	3.30436	3.30434
18.767	3.28175	3.28176	3.28174
18.77533	3.26325	3.26326	3.26324
18.78367	3.2593	3.25931	3.25929
18.792	3.272	3.27201	3.27199
18.80033	3.2821	3.28211	3.28209
18.80867	3.2872	3.28721	3.28719
18.817	3.29255	3.29256	3.29254
18.82533	3.2978	3.29781	3.29779
18.83367	3.3036	3.30361	3.30359
18.842	3.3029	3.30291	3.30289
18.85033	3.30715	3.30716	3.30714
18.85867	3.31675	3.31676	3.31674
18.867	3.32575	3.32576	3.32574
18.87533	3.33315	3.33316	3.33314
18.88367	3.33955	3.33956	3.33954
18.892	3.33955	3.33956	3.33954
18.90033	3.34065	3.34066	3.34064
18.90867	3.3372	3.33721	3.33719
18.917	3.32845	3.32846	3.32844
18.92533	3.31765	3.31766	3.31764
18.93367	3.30205	3.30206	3.30204
18.942	3.2739	3.27391	3.27389
18.95033	3.2529	3.25291	3.25289
18.95867	3.23135	3.23136	3.23134
18.967	3.22245	3.22246	3.22244
18.97533	3.2312	3.23121	3.23119
18.98367	3.24525	3.24526	3.24524
18.992	3.25065	3.25066	3.25064
19.00033	3.25585	3.25586	3.25584
19.00867	3.2617	3.26171	3.26169
19.017	3.26695	3.26696	3.26694
19.02533	3.27155	3.27156	3.27154

19.03367	3.27965	3.27966	3.27964
19.042	3.28625	3.28626	3.28624
19.05033	3.2928	3.29281	3.29279
19.05867	3.3009	3.30091	3.30089
19.067	3.3057	3.30571	3.30569
19.07533	3.3101	3.31011	3.31009
19.08367	3.30985	3.30986	3.30984
19.092	3.3091	3.30911	3.30909
19.10033	3.3012	3.30121	3.30119
19.10867	3.2901	3.29011	3.29009
19.117	3.27945	3.27946	3.27944
19.12533	3.2539	3.25391	3.25389
19.13367	3.23115	3.23116	3.23114
19.142	3.21035	3.21036	3.21034
19.15033	3.19295	3.19296	3.19294
19.15867	3.19555	3.19556	3.19554
19.167	3.2075	3.20751	3.20749
19.17533	3.2179	3.21791	3.21789
19.18367	3.2223	3.22231	3.22229
19.192	3.2281	3.22811	3.22809
19.20033	3.234	3.23401	3.23399
19.20867	3.239	3.23901	3.23899
19.217	3.24095	3.24096	3.24094
19.22533	3.2483	3.24831	3.24829
19.23367	3.25455	3.25456	3.25454
19.242	3.261	3.26101	3.26099
19.25033	3.27205	3.27206	3.27204
19.25867	3.27955	3.27956	3.27954
19.267	3.2736	3.27361	3.27359
19.27533	3.2762	3.27621	3.27619
19.28367	3.2713	3.27131	3.27129
19.292	3.262	3.26201	3.26199
19.30033	3.2515	3.25151	3.25149
19.30867	3.2336	3.23361	3.23359
19.317	3.2062	3.20621	3.20619
19.32533	3.18385	3.18386	3.18384
19.33367	3.16415	3.16416	3.16414
19.342	3.1611	3.16111	3.16109
19.35033	3.1738	3.17381	3.17379
19.35867	3.18485	3.18486	3.18484
19.367	3.1928	3.19281	3.19279
19.37533	3.19735	3.19736	3.19734
19.38367	3.20405	3.20406	3.20404
19.392	3.20885	3.20886	3.20884

19.40033	3.2144	3.21441	3.21439
19.40867	3.22015	3.22016	3.22014
19.417	3.2268	3.22681	3.22679
19.42533	3.23825	3.23826	3.23824
19.43367	3.24925	3.24926	3.24924
19.442	3.25435	3.25436	3.25434
19.45033	3.2563	3.25631	3.25629
19.45867	3.2582	3.25821	3.25819
19.467	3.25835	3.25836	3.25834
19.47533	3.24845	3.24846	3.24844
19.48367	3.23895	3.23896	3.23894
19.492	3.22495	3.22496	3.22494
19.50033	3.1985	3.19851	3.19849
19.50867	3.17865	3.17866	3.17864
19.517	3.16115	3.16116	3.16114
19.52533	3.15035	3.15036	3.15034
19.53367	3.16165	3.16166	3.16164
19.542	3.17355	3.17356	3.17354
19.55033	3.1824	3.18241	3.18239
19.55867	3.1871	3.18711	3.18709
19.567	3.19305	3.19306	3.19304
19.57533	3.2011	3.20111	3.20109
19.58367	3.2023	3.20231	3.20229
19.592	3.2094	3.20941	3.20939
19.60033	3.223	3.22301	3.22299
19.60867	3.23175	3.23176	3.23174
19.617	3.23815	3.23816	3.23814
19.62533	3.2495	3.24951	3.24949
19.63367	3.25845	3.25846	3.25844
19.642	3.25635	3.25636	3.25634
19.65033	3.26065	3.26066	3.26064
19.65867	3.25495	3.25496	3.25494
19.667	3.24615	3.24616	3.24614
19.67533	3.2364	3.23641	3.23639
19.68367	3.2128	3.21281	3.21279
19.692	3.19255	3.19256	3.19254
19.70033	3.1715	3.17151	3.17149
19.70867	3.1563	3.15631	3.15629
19.717	3.15815	3.15816	3.15814
19.72533	3.1742	3.17421	3.17419
19.73367	3.1843	3.18431	3.18429
19.742	3.1938	3.19381	3.19379
19.75033	3.19725	3.19726	3.19724
19.75867	3.2064	3.20641	3.20639

19.767	3.212	3.21201	3.21199
19.77533	3.2216	3.22161	3.22159
19.78367	3.2271	3.22711	3.22709
19.792	3.23425	3.23426	3.23424
19.80033	3.24605	3.24606	3.24604
19.80867	3.2562	3.25621	3.25619
19.817	3.2607	3.26071	3.26069
19.82533	3.26135	3.26136	3.26134
19.83367	3.26415	3.26416	3.26414
19.842	3.26235	3.26236	3.26234
19.85033	3.2536	3.25361	3.25359
19.85867	3.244	3.24401	3.24399
19.867	3.2265	3.22651	3.22649
19.87533	3.20105	3.20106	3.20104
19.88367	3.1807	3.18071	3.18069
19.892	3.161	3.16101	3.16099
19.90033	3.1555	3.15551	3.15549
19.90867	3.1676	3.16761	3.16759
19.917	3.18155	3.18156	3.18154
19.92533	3.1887	3.18871	3.18869
19.93367	3.1935	3.19351	3.19349
19.942	3.19795	3.19796	3.19794
19.95033	3.20565	3.20566	3.20564
19.95867	3.2044	3.20441	3.20439
19.967	3.2118	3.21181	3.21179
19.97533	3.2233	3.22331	3.22329
19.98367	3.23125	3.23126	3.23124
19.992	3.23875	3.23876	3.23874
20.00033	3.24855	3.24856	3.24854
20.00867	3.25135	3.25136	3.25134
20.017	3.25145	3.25146	3.25144
20.02533	3.2512	3.25121	3.25119
20.03367	3.24385	3.24386	3.24384
20.042	3.23205	3.23206	3.23204
20.05033	3.22095	3.22096	3.22094
20.05867	3.19445	3.19446	3.19444
20.067	3.1746	3.17461	3.17459
20.07533	3.1532	3.15321	3.15319
20.08367	3.14115	3.14116	3.14114
20.092	3.14535	3.14536	3.14534
20.10033	3.15995	3.15996	3.15994
20.10867	3.1682	3.16821	3.16819
20.117	3.175	3.17501	3.17499
20.12533	3.18015	3.18016	3.18014

20.13367	3.18605	3.18606	3.18604
20.142	3.1909	3.19091	3.19089
20.15033	3.19935	3.19936	3.19934
20.15867	3.206	3.20601	3.20599
20.167	3.2129	3.21291	3.21289
20.17533	3.22225	3.22226	3.22224
20.18367	3.22975	3.22976	3.22974
20.192	3.2365	3.23651	3.23649
20.20033	3.23525	3.23526	3.23524
20.20867	3.23735	3.23736	3.23734
20.217	3.23225	3.23226	3.23224
20.22533	3.222	3.22201	3.22199
20.23367	3.21305	3.21306	3.21304
20.242	3.19165	3.19166	3.19164
20.25033	3.1675	3.16751	3.16749
20.25867	3.14605	3.14606	3.14604
20.267	3.1272	3.12721	3.12719
20.27533	3.12735	3.12736	3.12734
20.28367	3.14	3.14001	3.13999
20.292	3.15145	3.15146	3.15144
20.30033	3.15735	3.15736	3.15734
20.30867	3.16415	3.16416	3.16414
20.317	3.17115	3.17116	3.17114
20.32533	3.17705	3.17706	3.17704
20.33367	3.17775	3.17776	3.17774
20.342	3.18435	3.18436	3.18434
20.35033	3.1919	3.19191	3.19189
20.35867	3.2004	3.20041	3.20039
20.367	3.2113	3.21131	3.21129
20.37533	3.2184	3.21841	3.21839
20.38367	3.21775	3.21776	3.21774
20.392	3.22	3.22001	3.21999
20.40033	3.2182	3.21821	3.21819
20.40867	3.20935	3.20936	3.20934
20.417	3.19885	3.19886	3.19884
20.42533	3.1843	3.18431	3.18429
20.43367	3.1582	3.15821	3.15819
20.442	3.1384	3.13841	3.13839
20.45033	3.11815	3.11816	3.11814
20.45867	3.11075	3.11076	3.11074
20.467	3.1224	3.12241	3.12239
20.47533	3.13445	3.13446	3.13444
20.48367	3.14245	3.14246	3.14244
20.492	3.1477	3.14771	3.14769

20.50033	3.155	3.15501	3.15499
20.50867	3.1615	3.16151	3.16149
20.517	3.16645	3.16646	3.16644
20.52533	3.1722	3.17221	3.17219
20.53367	3.17925	3.17926	3.17924
20.542	3.19055	3.19056	3.19054
20.55033	3.1974	3.19741	3.19739
20.55867	3.2028	3.20281	3.20279
20.567	3.2099	3.20991	3.20989
20.57533	3.20725	3.20726	3.20724
20.58367	3.20915	3.20916	3.20914
20.592	3.2012	3.20121	3.20119
20.60033	3.19335	3.19336	3.19334
20.60867	3.18275	3.18276	3.18274
20.617	3.1581	3.15811	3.15809
20.62533	3.1367	3.13671	3.13669
20.63367	3.11795	3.11796	3.11794
20.642	3.1035	3.10351	3.10349
20.65033	3.1083	3.10831	3.10829
20.65867	3.12025	3.12026	3.12024
20.667	3.13115	3.13116	3.13114
20.67533	3.1375	3.13751	3.13749
20.68367	3.14415	3.14416	3.14414
20.692	3.15235	3.15236	3.15234
20.70033	3.15305	3.15306	3.15304
20.70867	3.15815	3.15816	3.15814
20.717	3.1696	3.16961	3.16959
20.72533	3.1757	3.17571	3.17569
20.73367	3.18365	3.18366	3.18364
20.742	3.1972	3.19721	3.19719
20.75033	3.2064	3.20641	3.20639
20.75867	3.2027	3.20271	3.20269
20.767	3.20825	3.20826	3.20824
20.77533	3.20355	3.20356	3.20354
20.78367	3.19525	3.19526	3.19524
20.792	3.1854	3.18541	3.18539
20.80033	3.1662	3.16621	3.16619
20.80867	3.1422	3.14221	3.14219
20.817	3.1206	3.12061	3.12059
20.82533	3.10425	3.10426	3.10424
20.83367	3.1018	3.10181	3.10179
20.842	3.11675	3.11676	3.11674
20.85033	3.12805	3.12806	3.12804
20.85867	3.1377	3.13771	3.13769

20.867	3.142	3.14201	3.14199
20.87533	3.14945	3.14946	3.14944
20.88367	3.156	3.15601	3.15599
20.892	3.16415	3.16416	3.16414
20.90033	3.16895	3.16896	3.16894
20.90867	3.17795	3.17796	3.17794
20.917	3.19065	3.19066	3.19064
20.92533	3.19695	3.19696	3.19694
20.93367	3.20295	3.20296	3.20294
20.942	3.20825	3.20826	3.20824
20.95033	3.2119	3.21191	3.21189
20.95867	3.21245	3.21246	3.21244
20.967	3.20385	3.20386	3.20384
20.97533	3.1952	3.19521	3.19519
20.98367	3.1819	3.18191	3.18189
20.992	3.15635	3.15636	3.15634
21.00033	3.1379	3.13791	3.13789
21.00867	3.11945	3.11946	3.11944
21.017	3.1111	3.11111	3.11109
21.02533	3.11965	3.11966	3.11964
21.03367	3.1321	3.13211	3.13209
21.042	3.1394	3.13941	3.13939
21.05033	3.14655	3.14656	3.14654
21.05867	3.1523	3.15231	3.15229
21.067	3.1614	3.16141	3.16139
21.07533	3.16275	3.16276	3.16274
21.08367	3.17	3.17001	3.16999
21.092	3.17945	3.17946	3.17944
21.10033	3.1861	3.18611	3.18609
21.10867	3.1953	3.19531	3.19529
21.117	3.20445	3.20446	3.20444
21.12533	3.20975	3.20976	3.20974
21.13367	3.2083	3.20831	3.20829
21.142	3.21115	3.21116	3.21114
21.15033	3.2064	3.20641	3.20639
21.15867	3.1965	3.19651	3.19649
21.167	3.18715	3.18716	3.18714
21.17533	3.1637	3.16371	3.16369
21.18367	3.142	3.14201	3.14199
21.192	3.1204	3.12041	3.12039
21.20033	3.10465	3.10466	3.10464
21.20867	3.10755	3.10756	3.10754
21.217	3.12185	3.12186	3.12184
21.22533	3.13	3.13001	3.12999

21.23367	3.1364	3.13641	3.13639
21.242	3.14365	3.14366	3.14364
21.25033	3.14975	3.14976	3.14974
21.25867	3.15575	3.15576	3.15574
21.267	3.16005	3.16006	3.16004
21.27533	3.1662	3.16621	3.16619
21.28367	3.173	3.17301	3.17299
21.292	3.1817	3.18171	3.18169
21.30033	3.18995	3.18996	3.18994
21.30867	3.1975	3.19751	3.19749
21.317	3.19495	3.19496	3.19494
21.32533	3.19765	3.19766	3.19764
21.33367	3.192	3.19201	3.19199
21.342	3.1832	3.18321	3.18319
21.35033	3.1721	3.17211	3.17209
21.35867	3.15355	3.15356	3.15354
21.367	3.1267	3.12671	3.12669
21.37533	3.1071	3.10711	3.10709
21.38367	3.08665	3.08666	3.08664
21.392	3.0817	3.08171	3.08169
21.40033	3.0929	3.09291	3.09289
21.40867	3.10545	3.10546	3.10544
21.417	3.1095	3.10951	3.10949
21.42533	3.11445	3.11446	3.11444
21.43367	3.1191	3.11911	3.11909
21.442	3.1247	3.12471	3.12469
21.45033	3.12565	3.12566	3.12564
21.45867	3.13405	3.13406	3.13404
21.467	3.142	3.14201	3.14199
21.47533	3.1517	3.15171	3.15169
21.48367	3.16105	3.16106	3.16104
21.492	3.1668	3.16681	3.16679
21.50033	3.1688	3.16881	3.16879
21.50867	3.1696	3.16961	3.16959
21.517	3.1678	3.16781	3.16779
21.52533	3.1605	3.16051	3.16049
21.53367	3.1496	3.14961	3.14959
21.542	3.13805	3.13806	3.13804
21.55033	3.1125	3.11251	3.11249
21.55867	3.0927	3.09271	3.09269
21.567	3.0729	3.07291	3.07289
21.57533	3.05975	3.05976	3.05974
21.58367	3.0661	3.06611	3.06609
21.592	3.0777	3.07771	3.07769

21.60033	3.08765	3.08766	3.08764
21.60867	3.09215	3.09216	3.09214
21.617	3.099	3.09901	3.09899
21.62533	3.1054	3.10541	3.10539
21.63367	3.1105	3.11051	3.11049
21.642	3.1159	3.11591	3.11589
21.65033	3.12475	3.12476	3.12474
21.65867	3.13395	3.13396	3.13394
21.667	3.1407	3.14071	3.14069
21.67533	3.1491	3.14911	3.14909
21.68367	3.1576	3.15761	3.15759
21.692	3.1536	3.15361	3.15359
21.70033	3.15845	3.15846	3.15844
21.70867	3.1521	3.15211	3.15209
21.717	3.14395	3.14396	3.14394
21.72533	3.1342	3.13421	3.13419
21.73367	3.1133	3.11331	3.11329
21.742	3.08915	3.08916	3.08914
21.75033	3.06825	3.06826	3.06824
21.75867	3.0513	3.05131	3.05129
21.767	3.0519	3.05191	3.05189
21.77533	3.06615	3.06616	3.06614
21.78367	3.0779	3.07791	3.07789
21.792	3.0855	3.08551	3.08549
21.80033	3.0913	3.09131	3.09129
21.80867	3.09795	3.09796	3.09794
21.817	3.1032	3.10321	3.10319
21.82533	3.1089	3.10891	3.10889
21.83367	3.1155	3.11551	3.11549
21.842	3.12145	3.12146	3.12144
21.85033	3.13455	3.13456	3.13454
21.85867	3.14615	3.14616	3.14614
21.867	3.1501	3.15011	3.15009
21.87533	3.15025	3.15026	3.15024
21.88367	3.15395	3.15396	3.15394
21.892	3.1523	3.15231	3.15229
21.90033	3.1426	3.14261	3.14259
21.90867	3.1352	3.13521	3.13519
21.917	3.11915	3.11916	3.11914
21.92533	3.0944	3.09441	3.09439
21.93367	3.07455	3.07456	3.07454
21.942	3.057	3.05701	3.05699
21.95033	3.0505	3.05051	3.05049
21.95867	3.06255	3.06256	3.06254

21.967	3.07395	3.07396	3.07394
21.97533	3.08275	3.08276	3.08274
21.98367	3.08935	3.08936	3.08934
21.992	3.09575	3.09576	3.09574
22.00033	3.10485	3.10486	3.10484
22.00867	3.1031	3.10311	3.10309
22.017	3.1088	3.10881	3.10879
22.02533	3.121	3.12101	3.12099
22.03367	3.13155	3.13156	3.13154
22.042	3.13985	3.13986	3.13984
22.05033	3.1502	3.15021	3.15019
22.05867	3.15625	3.15626	3.15624
22.067	3.1572	3.15721	3.15719
22.07533	3.1586	3.15861	3.15859
22.08367	3.1523	3.15231	3.15229
22.092	3.1438	3.14381	3.14379
22.10033	3.1353	3.13531	3.13529
22.10867	3.10975	3.10976	3.10974
22.117	3.0909	3.09091	3.09089
22.12533	3.0701	3.07011	3.07009
22.13367	3.058	3.05801	3.05799
22.142	3.06405	3.06406	3.06404
22.15033	3.07975	3.07976	3.07974
22.15867	3.08865	3.08866	3.08864
22.167	3.09585	3.09586	3.09584
22.17533	3.1019	3.10191	3.10189
22.18367	3.11025	3.11026	3.11024
22.192	3.1134	3.11341	3.11339
22.20033	3.12005	3.12006	3.12004
22.20867	3.1285	3.12851	3.12849
22.217	3.13715	3.13716	3.13714
22.22533	3.15035	3.15036	3.15034
22.23367	3.16085	3.16086	3.16084
22.242	3.16705	3.16706	3.16704
22.25033	3.1683	3.16831	3.16829
22.25867	3.17205	3.17206	3.17204
22.267	3.1695	3.16951	3.16949
22.27533	3.16025	3.16026	3.16024
22.28367	3.1513	3.15131	3.15129
22.292	3.1313	3.13131	3.13129
22.30033	3.1076	3.10761	3.10759
22.30867	3.08965	3.08966	3.08964
22.317	3.0739	3.07391	3.07389
22.32533	3.07465	3.07466	3.07464

22.33367	3.088	3.08801	3.08799
22.342	3.1012	3.10121	3.10119
22.35033	3.1063	3.10631	3.10629
22.35867	3.1128	3.11281	3.11279
22.367	3.11965	3.11966	3.11964
22.37533	3.1278	3.12781	3.12779
22.38367	3.1256	3.12561	3.12559
22.392	3.13375	3.13376	3.13374
22.40033	3.14505	3.14506	3.14504
22.40867	3.15615	3.15616	3.15614
22.417	3.16565	3.16566	3.16564
22.42533	3.1749	3.17491	3.17489
22.43367	3.17595	3.17596	3.17594
22.442	3.1795	3.17951	3.17949
22.45033	3.1769	3.17691	3.17689
22.45867	3.16925	3.16926	3.16924
22.467	3.15895	3.15896	3.15894
22.47533	3.1457	3.14571	3.14569
22.48367	3.11985	3.11986	3.11984
22.492	3.10285	3.10286	3.10284
22.50033	3.08385	3.08386	3.08384
22.50867	3.0761	3.07611	3.07609
22.517	3.08535	3.08536	3.08534
22.52533	3.09825	3.09826	3.09824
22.53367	3.10665	3.10666	3.10664
22.542	3.1116	3.11161	3.11159
22.55033	3.1181	3.11811	3.11809
22.55867	3.12455	3.12456	3.12454
22.567	3.12765	3.12766	3.12764
22.57533	3.1341	3.13411	3.13409
22.58367	3.1408	3.14081	3.14079
22.592	3.14835	3.14836	3.14834
22.60033	3.15615	3.15616	3.15614
22.60867	3.16415	3.16416	3.16414
22.617	3.16865	3.16866	3.16864
22.62533	3.16735	3.16736	3.16734
22.63367	3.16855	3.16856	3.16854
22.642	3.1609	3.16091	3.16089
22.65033	3.1505	3.15051	3.15049
22.65867	3.1387	3.13871	3.13869
22.667	3.1137	3.11371	3.11369
22.67533	3.09205	3.09206	3.09204
22.68367	3.07095	3.07096	3.07094
22.692	3.05495	3.05496	3.05494

22.70033	3.0589	3.05891	3.05889
22.70867	3.0709	3.07091	3.07089
22.717	3.0796	3.07961	3.07959
22.72533	3.084	3.08401	3.08399
22.73367	3.0887	3.08871	3.08869
22.742	3.09395	3.09396	3.09394
22.75033	3.0953	3.09531	3.09529
22.75867	3.09555	3.09556	3.09554
22.767	3.1056	3.10561	3.10559
22.77533	3.1117	3.11171	3.11169
22.78367	3.12035	3.12036	3.12034
22.792	3.1321	3.13211	3.13209
22.80033	3.1376	3.13761	3.13759
22.80867	3.13385	3.13386	3.13384
22.817	3.13835	3.13836	3.13834
22.82533	3.1331	3.13311	3.13309
22.83367	3.12235	3.12236	3.12234
22.842	3.1127	3.11271	3.11269
22.85033	3.09345	3.09346	3.09344
22.85867	3.0681	3.06811	3.06809
22.867	3.04695	3.04696	3.04694
22.87533	3.0297	3.02971	3.02969
22.88367	3.02615	3.02616	3.02614
22.892	3.03885	3.03886	3.03884
22.90033	3.04865	3.04866	3.04864
22.90867	3.0545	3.05451	3.05449
22.917	3.05885	3.05886	3.05884
22.92533	3.0687	3.06871	3.06869
22.93367	3.07335	3.07336	3.07334
22.942	3.07555	3.07556	3.07554
22.95033	3.0788	3.07881	3.07879
22.95867	3.08945	3.08946	3.08944
22.967	3.1013	3.10131	3.10129
22.97533	3.1087	3.10871	3.10869
22.98367	3.11365	3.11366	3.11364
22.992	3.11805	3.11806	3.11804
23.00033	3.11895	3.11896	3.11894
23.00867	3.12	3.12001	3.11999
23.017	3.11045	3.11046	3.11044
23.02533	3.1003	3.10031	3.10029
23.03367	3.08575	3.08576	3.08574
23.042	3.06065	3.06066	3.06064
23.05033	3.0425	3.04251	3.04249
23.05867	3.02315	3.02316	3.02314

23.067	3.013	3.01301	3.01299
23.07533	3.0217	3.02171	3.02169
23.08367	3.0354	3.03541	3.03539
23.092	3.04285	3.04286	3.04284
23.10033	3.05005	3.05006	3.05004
23.10867	3.05595	3.05596	3.05594
23.117	3.06465	3.06466	3.06464
23.12533	3.06305	3.06306	3.06304
23.13367	3.0688	3.06881	3.06879
23.142	3.07915	3.07916	3.07914
23.15033	3.08505	3.08506	3.08504
23.15867	3.0958	3.09581	3.09579
23.167	3.1085	3.10851	3.10849
23.17533	3.1122	3.11221	3.11219
23.18367	3.11325	3.11326	3.11324
23.192	3.1148	3.11481	3.11479
23.20033	3.11075	3.11076	3.11074
23.20867	3.10165	3.10166	3.10164
23.217	3.09325	3.09326	3.09324
23.22533	3.0693	3.06931	3.06929
23.23367	3.0472	3.04721	3.04719
23.242	3.0266	3.02661	3.02659
23.25033	3.0129	3.01291	3.01289
23.25867	3.0152	3.01521	3.01519
23.267	3.0307	3.03071	3.03069
23.27533	3.0406	3.04061	3.04059
23.28367	3.04695	3.04696	3.04694
23.292	3.0523	3.05231	3.05229
23.30033	3.0584	3.05841	3.05839
23.30867	3.0648	3.06481	3.06479
23.317	3.06765	3.06766	3.06764
23.32533	3.07325	3.07326	3.07324
23.33367	3.0851	3.08511	3.08509
23.342	3.0976	3.09761	3.09759
23.35033	3.10715	3.10716	3.10714
23.35867	3.11375	3.11376	3.11374
23.367	3.1134	3.11341	3.11339
23.37533	3.11465	3.11466	3.11464
23.38367	3.11195	3.11196	3.11194
23.392	3.1036	3.10361	3.10359
23.40033	3.0948	3.09481	3.09479
23.40867	3.078	3.07801	3.07799
23.417	3.05315	3.05316	3.05314
23.42533	3.0347	3.03471	3.03469

23.43367	3.01525	3.01526	3.01524
23.442	3.013	3.01301	3.01299
23.45033	3.0238	3.02381	3.02379
23.45867	3.03645	3.03646	3.03644
23.467	3.04265	3.04266	3.04264
23.47533	3.0484	3.04841	3.04839
23.48367	3.05495	3.05496	3.05494
23.492	3.06195	3.06196	3.06194
23.50033	3.0599	3.05991	3.05989
23.50867	3.06805	3.06806	3.06804
23.517	3.0781	3.07811	3.07809
23.52533	3.0857	3.08571	3.08569
23.53367	3.0942	3.09421	3.09419
23.542	3.1029	3.10291	3.10289
23.55033	3.1056	3.10561	3.10559
23.55867	3.1096	3.10961	3.10959
23.567	3.1094	3.10941	3.10939
23.57533	3.1029	3.10291	3.10289
23.58367	3.0921	3.09211	3.09209
23.592	3.08075	3.08076	3.08074
23.60033	3.05325	3.05326	3.05324
23.60867	3.03375	3.03376	3.03374
23.617	3.01485	3.01486	3.01484
23.62533	3.0038	3.00381	3.00379
23.63367	3.0121	3.01211	3.01209
23.642	3.02425	3.02426	3.02424
23.65033	3.03305	3.03306	3.03304
23.65867	3.038	3.03801	3.03799
23.667	3.04575	3.04576	3.04574
23.67533	3.0498	3.04981	3.04979
23.68367	3.05185	3.05186	3.05184
23.692	3.0583	3.05831	3.05829
23.70033	3.06635	3.06636	3.06634
23.70867	3.07515	3.07516	3.07514
23.717	3.08345	3.08346	3.08344
23.72533	3.0902	3.09021	3.09019
23.73367	3.09535	3.09536	3.09534
23.742	3.09355	3.09356	3.09354
23.75033	3.0958	3.09581	3.09579
23.75867	3.0902	3.09021	3.09019
23.767	3.0785	3.07851	3.07849
23.77533	3.06705	3.06706	3.06704
23.78367	3.04505	3.04506	3.04504
23.792	3.02135	3.02136	3.02134

23.80033	2.9999	2.99991	2.99989
23.80867	2.9834	2.98341	2.98339
23.817	2.9868	2.98681	2.98679
23.82533	2.99905	2.99906	2.99904
23.83367	3.00765	3.00766	3.00764
23.842	3.0124	3.01241	3.01239
23.85033	3.0171	3.01711	3.01709
23.85867	3.0248	3.02481	3.02479
23.867	3.02975	3.02976	3.02974
23.87533	3.03665	3.03666	3.03664
23.88367	3.04225	3.04226	3.04224
23.892	3.04915	3.04916	3.04914
23.90033	3.05925	3.05926	3.05924
23.90867	3.0671	3.06711	3.06709
23.917	3.0723	3.07231	3.07229
23.92533	3.071	3.07101	3.07099
23.93367	3.0734	3.07341	3.07339
23.942	3.07055	3.07056	3.07054
23.95033	3.06005	3.06006	3.06004
23.95867	3.05215	3.05216	3.05214
23.967	3.03455	3.03456	3.03454
23.97533	3.00925	3.00926	3.00924
23.98367	2.98785	2.98786	2.98784
23.992	2.9696	2.96961	2.96959
24.00033	2.96205	2.96206	2.96204
24.00867	2.9745	2.97451	2.97449
24.017	2.9846	2.98461	2.98459
24.02533	2.99265	2.99266	2.99264
24.03367	2.998	2.99801	2.99799
24.042	3.0053	3.00531	3.00529
24.05033	3.0117	3.01171	3.01169
24.05867	3.01225	3.01226	3.01224
24.067	3.01775	3.01776	3.01774
24.07533	3.02995	3.02996	3.02994
24.08367	3.03785	3.03786	3.03784
24.092	3.0451	3.04511	3.04509
24.10033	3.0544	3.05441	3.05439
24.10867	3.05805	3.05806	3.05804
24.117	3.0578	3.05781	3.05779
24.12533	3.05915	3.05916	3.05914
24.13367	3.0516	3.05161	3.05159
24.142	3.04095	3.04096	3.04094
24.15033	3.0285	3.02851	3.02849
24.15867	3.0019	3.00191	3.00189

24.167	2.9821	2.98211	2.98209
24.17533	2.96165	2.96166	2.96164
24.18367	2.94955	2.94956	2.94954
24.192	2.9558	2.95581	2.95579
24.20033	2.96985	2.96986	2.96984
24.20867	2.97795	2.97796	2.97794
24.217	2.98435	2.98436	2.98434
24.22533	2.9886	2.98861	2.98859
24.23367	2.9978	2.99781	2.99779
24.242	3.00145	3.00146	3.00144
24.25033	3.0105	3.01051	3.01049
24.25867	3.01595	3.01596	3.01594
24.267	3.0228	3.02281	3.02279
24.27533	3.036	3.03601	3.03599
24.28367	3.0462	3.04621	3.04619
24.292	3.04985	3.04986	3.04984
24.30033	3.0501	3.05011	3.05009
24.30867	3.0512	3.05121	3.05119
24.317	3.04505	3.04506	3.04504
24.32533	3.0337	3.03371	3.03369
24.33367	3.0249	3.02491	3.02489
24.342	3.0036	3.00361	3.00359
24.35033	2.9807	2.98071	2.98069
24.35867	2.9607	2.96071	2.96069
24.367	2.945	2.94501	2.94499
24.37533	2.94375	2.94376	2.94374
24.38367	2.9569	2.95691	2.95689
24.392	2.9679	2.96791	2.96789
24.40033	2.9737	2.97371	2.97369
24.40867	2.979	2.97901	2.97899
24.417	2.9848	2.98481	2.98479
24.42533	2.99225	2.99226	2.99224
24.43367	2.9928	2.99281	2.99279
24.442	3.0001	3.00011	3.00009
24.45033	3.01045	3.01046	3.01044
24.45867	3.0192	3.01921	3.01919
24.467	3.0266	3.02661	3.02659
24.47533	3.03495	3.03496	3.03494
24.48367	3.03555	3.03556	3.03554
24.492	3.03905	3.03906	3.03904
24.50033	3.03835	3.03836	3.03834
24.50867	3.0299	3.02991	3.02989
24.517	3.02125	3.02126	3.02124
24.52533	3.00625	3.00626	3.00624

24.53367	2.97805	2.97806	2.97804
24.542	2.95925	2.95926	2.95924
24.55033	2.9395	2.93951	2.93949
24.55867	2.93205	2.93206	2.93204
24.567	2.94155	2.94156	2.94154
24.57533	2.9543	2.95431	2.95429
24.58367	2.95975	2.95976	2.95974
24.592	2.9646	2.96461	2.96459
24.60033	2.9717	2.97171	2.97169
24.60867	2.9771	2.97711	2.97709
24.617	2.97715	2.97716	2.97714
24.62533	2.98765	2.98766	2.98764
24.63367	2.99525	2.99526	2.99524
24.642	3.00345	3.00346	3.00344
24.65033	3.01415	3.01416	3.01414
24.65867	3.0237	3.02371	3.02369
24.667	3.02675	3.02676	3.02674
24.67533	3.0274	3.02741	3.02739
24.68367	3.0283	3.02831	3.02829
24.692	3.0207	3.02071	3.02069
24.70033	3.01085	3.01086	3.01084
24.70867	2.9997	2.99971	2.99969
24.717	2.97515	2.97516	2.97514
24.72533	2.95325	2.95326	2.95324
24.73367	2.93405	2.93406	2.93404
24.742	2.91985	2.91986	2.91984
24.75033	2.9259	2.92591	2.92589
24.75867	2.93845	2.93846	2.93844
24.767	2.9479	2.94791	2.94789
24.77533	2.9527	2.95271	2.95269
24.78367	2.96035	2.96036	2.96034
24.792	2.9666	2.96661	2.96659
24.80033	2.9713	2.97131	2.97129
24.80867	2.9735	2.97351	2.97349
24.817	2.98275	2.98276	2.98274
24.82533	2.99335	2.99336	2.99334
24.83367	3.00195	3.00196	3.00194
24.842	3.01285	3.01286	3.01284
24.85033	3.0192	3.01921	3.01919
24.85867	3.01845	3.01846	3.01844
24.867	3.02265	3.02266	3.02264
24.87533	3.0188	3.01881	3.01879
24.88367	3.01055	3.01056	3.01054
24.892	3.00005	3.00006	3.00004

24.90033	2.9816	2.98161	2.98159
24.90867	2.9577	2.95771	2.95769
24.917	2.93895	2.93896	2.93894
24.92533	2.92185	2.92186	2.92184
24.93367	2.9207	2.92071	2.92069
24.942	2.93445	2.93446	2.93444
24.95033	2.94565	2.94566	2.94564
24.95867	2.9523	2.95231	2.95229
24.967	2.95765	2.95766	2.95764
24.97533	2.96685	2.96686	2.96684
24.98367	2.97475	2.97476	2.97474
24.992	2.9818	2.98181	2.98179
25.00033	2.98985	2.98986	2.98984
25.00867	2.9981	2.99811	2.99809
25.017	3.00805	3.00806	3.00804
25.02533	3.01605	3.01606	3.01604
25.03367	3.02195	3.02196	3.02194
25.042	3.02605	3.02606	3.02604
25.05033	3.02695	3.02696	3.02694
25.05867	3.0277	3.02771	3.02769
25.067	3.0185	3.01851	3.01849
25.07533	3.01035	3.01036	3.01034
25.08367	2.9963	2.99631	2.99629
25.092	2.96985	2.96986	2.96984
25.10033	2.94915	2.94916	2.94914
25.10867	2.9311	2.93111	2.93109
25.117	2.9197	2.91971	2.91969
25.12533	2.9306	2.93061	2.93059
25.13367	2.94445	2.94446	2.94444
25.142	2.9526	2.95261	2.95259
25.15033	2.95585	2.95586	2.95584
25.15867	2.9614	2.96141	2.96139
25.167	2.96865	2.96866	2.96864
25.17533	2.96965	2.96966	2.96964
25.18367	2.9775	2.97751	2.97749
25.192	2.98685	2.98686	2.98684
25.20033	2.99255	2.99256	2.99254
25.20867	2.99965	2.99966	2.99964
25.217	3.01235	3.01236	3.01234
25.22533	3.0176	3.01761	3.01759
25.23367	3.01645	3.01646	3.01644
25.242	3.01815	3.01816	3.01814
25.25033	3.0109	3.01091	3.01089
25.25867	2.9998	2.99981	2.99979

25.267	2.98965	2.98966	2.98964
25.27533	2.96535	2.96536	2.96534
25.28367	2.94345	2.94346	2.94344
25.292	2.92195	2.92196	2.92194
25.30033	2.9075	2.90751	2.90749
25.30867	2.90875	2.90876	2.90874
25.317	2.92325	2.92326	2.92324
25.32533	2.931	2.93101	2.93099
25.33367	2.93695	2.93696	2.93694
25.342	2.9415	2.94151	2.94149
25.35033	2.9497	2.94971	2.94969
25.35867	2.9548	2.95481	2.95479
25.367	2.9613	2.96131	2.96129
25.37533	2.9667	2.96671	2.96669
25.38367	2.975	2.97501	2.97499
25.392	2.9874	2.98741	2.98739
25.40033	2.9952	2.99521	2.99519
25.40867	2.99885	2.99886	2.99884
25.417	3.0008	3.00081	3.00079
25.42533	3.00235	3.00236	3.00234
25.43367	3.0006	3.00061	3.00059
25.442	2.9913	2.99131	2.99129
25.45033	2.98175	2.98176	2.98174
25.45867	2.96345	2.96346	2.96344
25.467	2.93775	2.93776	2.93774
25.47533	2.91765	2.91766	2.91764
25.48367	2.89855	2.89856	2.89854
25.492	2.89315	2.89316	2.89314
25.50033	2.90445	2.90446	2.90444
25.50867	2.91615	2.91616	2.91614
25.517	2.921	2.92101	2.92099
25.52533	2.9282	2.92821	2.92819
25.53367	2.9337	2.93371	2.93369
25.542	2.94015	2.94016	2.94014
25.55033	2.9366	2.93661	2.93659
25.55867	2.94385	2.94386	2.94384
25.567	2.95485	2.95486	2.95484
25.57533	2.96245	2.96246	2.96244
25.58367	2.96805	2.96806	2.96804
25.592	2.97755	2.97756	2.97754
25.60033	2.98135	2.98136	2.98134
25.60867	2.9819	2.98191	2.98189
25.617	2.98125	2.98126	2.98124
25.62533	2.9738	2.97381	2.97379

25.63367	2.9614	2.96141	2.96139
25.642	2.9494	2.94941	2.94939
25.65033	2.9231	2.92311	2.92309
25.65867	2.90265	2.90266	2.90264
25.667	2.88155	2.88156	2.88154
25.67533	2.8703	2.87031	2.87029
25.68367	2.87665	2.87666	2.87664
25.692	2.8912	2.89121	2.89119
25.70033	2.8973	2.89731	2.89729
25.70867	2.90125	2.90126	2.90124
25.717	2.9067	2.90671	2.90669
25.72533	2.9123	2.91231	2.91229
25.73367	2.9169	2.91691	2.91689
25.742	2.92295	2.92296	2.92294
25.75033	2.92725	2.92726	2.92724
25.75867	2.93535	2.93536	2.93534
25.767	2.945	2.94501	2.94499
25.77533	2.9523	2.95231	2.95229
25.78367	2.9574	2.95741	2.95739
25.792	2.9557	2.95571	2.95569
25.80033	2.95695	2.95696	2.95694
25.80867	2.951	2.95101	2.95099
25.817	2.94065	2.94066	2.94064
25.82533	2.93055	2.93056	2.93054
25.83367	2.90775	2.90776	2.90774
25.842	2.8837	2.88371	2.88369
25.85033	2.8654	2.86541	2.86539
25.85867	2.8478	2.84781	2.84779
25.867	2.84815	2.84816	2.84814
25.87533	2.8597	2.85971	2.85969
25.88367	2.87025	2.87026	2.87024
25.892	2.8749	2.87491	2.87489
25.90033	2.8812	2.88121	2.88119
25.90867	2.8897	2.88971	2.88969
25.917	2.89545	2.89546	2.89544
25.92533	2.89515	2.89516	2.89514
25.93367	2.9035	2.90351	2.90349
25.942	2.91255	2.91256	2.91254
25.95033	2.922	2.92201	2.92199
25.95867	2.93455	2.93456	2.93454
25.967	2.9422	2.94221	2.94219
25.97533	2.94225	2.94226	2.94224
25.98367	2.9456	2.94561	2.94559
25.992	2.94125	2.94126	2.94124

26.00033	2.9336	2.93361	2.93359
26.00867	2.9238	2.92381	2.92379
26.017	2.9095	2.90951	2.90949
26.02533	2.88335	2.88336	2.88334
26.03367	2.86365	2.86366	2.86364
26.042	2.84545	2.84546	2.84544
26.05033	2.8402	2.84021	2.84019
26.05867	2.8516	2.85161	2.85159
26.067	2.86345	2.86346	2.86344
26.07533	2.87245	2.87246	2.87244
26.08367	2.8752	2.87521	2.87519
26.092	2.88305	2.88306	2.88304
26.10033	2.88855	2.88856	2.88854
26.10867	2.8925	2.89251	2.89249
26.117	2.89885	2.89886	2.89884
26.12533	2.9082	2.90821	2.90819
26.13367	2.91785	2.91786	2.91784
26.142	2.9249	2.92491	2.92489
26.15033	2.93235	2.93236	2.93234
26.15867	2.9387	2.93871	2.93869
26.167	2.9385	2.93851	2.93849
26.17533	2.9387	2.93871	2.93869
26.18367	2.9284	2.92841	2.92839
26.192	2.91745	2.91746	2.91744
26.20033	2.90505	2.90506	2.90504
26.20867	2.8816	2.88161	2.88159
26.217	2.8604	2.86041	2.86039
26.22533	2.84235	2.84236	2.84234
26.23367	2.82925	2.82926	2.82924
26.242	2.83535	2.83536	2.83534
26.25033	2.84835	2.84836	2.84834
26.25867	2.85535	2.85536	2.85534
26.267	2.8604	2.86041	2.86039
26.27533	2.86685	2.86686	2.86684
26.28367	2.8758	2.87581	2.87579
26.292	2.87685	2.87686	2.87684
26.30033	2.8813	2.88131	2.88129
26.30867	2.89015	2.89016	2.89014
26.317	2.8949	2.89491	2.89489
26.32533	2.90425	2.90426	2.90424
26.33367	2.91485	2.91486	2.91484
26.342	2.91855	2.91856	2.91854
26.35033	2.9177	2.91771	2.91769
26.35867	2.9194	2.91941	2.91939

26.367	2.91375	2.91376	2.91374
26.37533	2.90395	2.90396	2.90394
26.38367	2.89515	2.89516	2.89514
26.392	2.8744	2.87441	2.87439
26.40033	2.85025	2.85026	2.85024
26.40867	2.8295	2.82951	2.82949
26.417	2.8145	2.81451	2.81449
26.42533	2.81335	2.81336	2.81334
26.43367	2.83	2.83001	2.82999
26.442	2.83975	2.83976	2.83974
26.45033	2.84615	2.84616	2.84614
26.45867	2.8518	2.85181	2.85179
26.467	2.8607	2.86071	2.86069
26.47533	2.8668	2.86681	2.86679
26.48367	2.8669	2.86691	2.86689
26.492	2.873	2.87301	2.87299
26.50033	2.88535	2.88536	2.88534
26.50867	2.8956	2.89561	2.89559
26.517	2.90175	2.90176	2.90174
26.52533	2.9112	2.91121	2.91119
26.53367	2.91295	2.91296	2.91294
26.542	2.9129	2.91291	2.91289
26.55033	2.9112	2.91121	2.91119
26.55867	2.9031	2.90311	2.90309
26.567	2.89275	2.89276	2.89274
26.57533	2.8781	2.87811	2.87809
26.58367	2.85205	2.85206	2.85204
26.592	2.8348	2.83481	2.83479
26.60033	2.8152	2.81521	2.81519
26.60867	2.80895	2.80896	2.80894
26.617	2.81855	2.81856	2.81854
26.62533	2.8337	2.83371	2.83369
26.63367	2.8396	2.83961	2.83959
26.642	2.8455	2.84551	2.84549
26.65033	2.8515	2.85151	2.85149
26.65867	2.85845	2.85846	2.85844
26.667	2.851	2.85101	2.85099
26.67533	2.85635	2.85636	2.85634
26.68367	2.86665	2.86666	2.86664
26.692	2.87535	2.87536	2.87534
26.70033	2.8868	2.88681	2.88679
26.70867	2.89615	2.89616	2.89614
26.717	2.8991	2.89911	2.89909
26.72533	2.90015	2.90016	2.90014

26.73367	2.9016	2.90161	2.90159
26.742	2.8953	2.89531	2.89529
26.75033	2.88275	2.88276	2.88274
26.75867	2.8719	2.87191	2.87189
26.767	2.8475	2.84751	2.84749
26.77533	2.82595	2.82596	2.82594
26.78367	2.8065	2.80651	2.80649
26.792	2.792	2.79201	2.79199
26.80033	2.7964	2.79641	2.79639
26.80867	2.81	2.81001	2.80999
26.817	2.8199	2.81991	2.81989
26.82533	2.82515	2.82516	2.82514
26.83367	2.83265	2.83266	2.83264
26.842	2.83865	2.83866	2.83864
26.85033	2.84065	2.84066	2.84064
26.85867	2.8391	2.83911	2.83909
26.867	2.84575	2.84576	2.84574
26.87533	2.8558	2.85581	2.85579
26.88367	2.86675	2.86676	2.86674
26.892	2.8753	2.87531	2.87529
26.90033	2.8825	2.88251	2.88249
26.90867	2.88095	2.88096	2.88094
26.917	2.8845	2.88451	2.88449
26.92533	2.8812	2.88121	2.88119
26.93367	2.872	2.87201	2.87199
26.942	2.8614	2.86141	2.86139
26.95033	2.84405	2.84406	2.84404
26.95867	2.81795	2.81796	2.81794
26.967	2.8011	2.80111	2.80109
26.97533	2.7838	2.78381	2.78379
26.98367	2.78275	2.78276	2.78274
26.992	2.79595	2.79596	2.79594
27.00033	2.8078	2.80781	2.80779
27.00867	2.81525	2.81526	2.81524
27.017	2.82085	2.82086	2.82084
27.02533	2.82835	2.82836	2.82834
27.03367	2.8339	2.83391	2.83389
27.042	2.83045	2.83046	2.83044
27.05033	2.8396	2.83961	2.83959
27.05867	2.84905	2.84906	2.84904
27.067	2.85845	2.85846	2.85844
27.07533	2.86845	2.86846	2.86844
27.08367	2.8766	2.87661	2.87659
27.092	2.8788	2.87881	2.87879

27.10033	2.88265	2.88266	2.88264
27.10867	2.88285	2.88286	2.88284
27.117	2.8739	2.87391	2.87389
27.12533	2.8642	2.86421	2.86419
27.13367	2.8512	2.85121	2.85119
27.142	2.8255	2.82551	2.82549
27.15033	2.80705	2.80706	2.80704
27.15867	2.79005	2.79006	2.79004
27.167	2.7812	2.78121	2.78119
27.17533	2.79325	2.79326	2.79324
27.18367	2.80525	2.80526	2.80524
27.192	2.81465	2.81466	2.81464
27.20033	2.81975	2.81976	2.81974
27.20867	2.8288	2.82881	2.82879
27.217	2.8352	2.83521	2.83519
27.22533	2.8345	2.83451	2.83449
27.23367	2.83805	2.83806	2.83804
27.242	2.8489	2.84891	2.84889
27.25033	2.8581	2.85811	2.85809
27.25867	2.86805	2.86806	2.86804
27.267	2.88125	2.88126	2.88124
27.27533	2.8884	2.88841	2.88839
27.28367	2.88485	2.88486	2.88484
27.292	2.88705	2.88706	2.88704
27.30033	2.8808	2.88081	2.88079
27.30867	2.8723	2.87231	2.87229
27.317	2.86085	2.86086	2.86084
27.32533	2.8393	2.83931	2.83929
27.33367	2.81525	2.81526	2.81524
27.342	2.7922	2.79221	2.79219
27.35033	2.77315	2.77316	2.77314
27.35867	2.77175	2.77176	2.77174
27.367	2.7813	2.78131	2.78129
27.37533	2.7822	2.78221	2.78219
27.38367	2.7775	2.77751	2.77749
27.392	2.76735	2.76736	2.76734
27.40033	2.75695	2.75696	2.75694
27.40867	2.7353	2.73531	2.73529
27.417	2.70545	2.70546	2.70544
27.42533	2.67695	2.67696	2.67694
27.43367	2.6437	2.64371	2.64369
27.442	2.60875	2.60876	2.60874
27.45033	2.5647	2.56471	2.56469
27.45867	2.5074	2.50741	2.50739

27.467	2.44015	2.44016	2.44014
27.47533	2.36515	2.36516	2.36514
27.48367	2.27925	2.27926	2.27924
27.492	2.1788	2.17881	2.17879
27.50033	2.0737	2.07371	2.07369
27.50867	1.95365	1.95366	1.95364
27.517	1.8164	1.81641	1.81639
27.52533	1.68055	1.68056	1.68054
27.53367	1.53965	1.53966	1.53964
27.542	1.40155	1.40156	1.40154
27.55033	1.2799	1.27991	1.27989
27.55867	1.15225	1.15226	1.15224
27.567	1.0134	1.01341	1.01339
27.57533	0.86715	0.86716	0.86714
27.58367	0.71575	0.71576	0.71574
27.592	0.55885	0.55886	0.55884
27.60033	0.38335	0.38336	0.38334
27.60867	0.21025	0.21026	0.21024
27.617	0.03775	0.03776	0.03774
27.62533	-0.1447	-0.14469	-0.14471
27.63367	-0.3306	-0.33059	-0.33061
27.642	-0.5108	-0.51079	-0.51081
27.65033	-0.69025	-0.69024	-0.69026
27.65867	-0.8733	-0.87329	-0.87331
27.667	-1.03715	-1.03714	-1.03716
27.67533	-1.1924	-1.19239	-1.19241
27.68367	-1.3313	-1.33129	-1.33131
27.692	-1.4495	-1.44949	-1.44951
27.70033	-1.55515	-1.55514	-1.55516
27.70867	-1.62945	-1.62944	-1.62946
27.717	-1.67585	-1.67584	-1.67586
27.72533	-1.6853	-1.68529	-1.68531
27.73367	-1.6432	-1.64319	-1.64321
27.742	-1.5581	-1.55809	-1.55811
27.75033	-1.45385	-1.45384	-1.45386
27.75867	-1.3297	-1.32969	-1.32971
27.767	-1.1864	-1.18639	-1.18641
27.77533	-1.0248	-1.02479	-1.02481
27.78367	-0.85155	-0.85154	-0.85156
27.792	-0.6612	-0.66119	-0.66121
27.80033	-0.4621	-0.46209	-0.46211
27.80867	-0.25065	-0.25064	-0.25066
27.817	-0.02245	-0.02244	-0.02246
27.82533	0.2116	0.21161	0.21159

27.83367	0.44625	0.44626	0.44624
27.842	0.67425	0.67426	0.67424
27.85033	0.9045	0.90451	0.90449
27.85867	1.12695	1.12696	1.12694
27.867	1.33875	1.33876	1.33874
27.87533	1.5524	1.55241	1.55239
27.88367	1.7518	1.75181	1.75179
27.892	1.9428	1.94281	1.94279
27.90033	2.14485	2.14486	2.14484
27.90867	2.34595	2.34596	2.34594
27.917	2.56445	2.56446	2.56444
27.92533	2.7966	2.79661	2.79659
27.93367	3.01905	3.01906	3.01904
27.942	3.22525	3.22526	3.22524
27.95033	3.4299	3.42991	3.42989
27.95867	3.6246	3.62461	3.62459
27.967	3.81945	3.81946	3.81944
27.97533	3.99365	3.99366	3.99364
27.98367	4.18215	4.18216	4.18214
27.992	4.3692	4.36921	4.36919
28.00033	4.54425	4.54426	4.54424
28.00867	4.714	4.71401	4.71399
28.017	4.88165	4.88166	4.88164
28.02533	5.0382	5.03821	5.03819
28.03367	5.1929	5.19291	5.19289
28.042	5.3439	5.34391	5.34389
28.05033	5.48005	5.48006	5.48004
28.05867	5.6159	5.61591	5.61589
28.067	5.75295	5.75296	5.75294
28.07533	5.86575	5.86576	5.86574
28.08367	6.00275	6.00276	6.00274
28.092	6.13145	6.13146	6.13144
28.10033	6.27255	6.27256	6.27254
28.10867	6.42465	6.42466	6.42464
28.117	6.5708	6.57081	6.57079
28.12533	6.69625	6.69626	6.69624
28.13367	6.8179	6.81791	6.81789
28.142	6.93715	6.93716	6.93714
28.15033	7.0532	7.05321	7.05319
28.15867	7.1601	7.16011	7.16009
28.167	7.2714	7.27141	7.27139
28.17533	7.38015	7.38016	7.38014
28.18367	7.48535	7.48536	7.48534
28.192	7.59155	7.59156	7.59154

28.20033	7.6888	7.68881	7.68879
28.20867	7.7851	7.78511	7.78509
28.217	7.869	7.86901	7.86899
28.22533	7.96485	7.96486	7.96484
28.23367	8.05005	8.05006	8.05004
28.242	8.1334	8.13341	8.13339
28.25033	8.2193	8.21931	8.21929
28.25867	8.2905	8.29051	8.29049
28.267	8.3762	8.37621	8.37619
28.27533	8.45875	8.45876	8.45874
28.28367	8.5474	8.54741	8.54739
28.292	8.65365	8.65366	8.65364
28.30033	8.75505	8.75506	8.75504
28.30867	8.8447	8.84471	8.84469
28.317	8.91765	8.91766	8.91764
28.32533	8.99255	8.99256	8.99254
28.33367	9.0653	9.06531	9.06529
28.342	9.1373	9.13731	9.13729
28.35033	9.19775	9.19776	9.19774
28.35867	9.27785	9.27786	9.27784
28.367	9.3442	9.34421	9.34419
28.37533	9.41435	9.41436	9.41434
28.38367	9.47945	9.47946	9.47944
28.392	9.5384	9.53841	9.53839
28.40033	9.595	9.59501	9.59499
28.40867	9.6588	9.65881	9.65879
28.417	9.70685	9.70686	9.70684
28.42533	9.7587	9.75871	9.75869
28.43367	9.8193	9.81931	9.81929
28.442	9.8735	9.87351	9.87349
28.45033	9.92355	9.92356	9.92354
28.45867	9.98215	9.98216	9.98214
28.467	10.0493	10.04931	10.04929
28.47533	10.12045	10.12046	10.12044
28.48367	10.2046	10.20461	10.20459
28.492	10.27385	10.27386	10.27384
28.50033	10.3402	10.34021	10.34019
28.50867	10.39585	10.39586	10.39584
28.517	10.44015	10.44016	10.44014
28.52533	10.48685	10.48686	10.48684
28.53367	10.535	10.53501	10.53499
28.542	10.5815	10.58151	10.58149
28.55033	10.6443	10.64431	10.64429
28.55867	10.6962	10.69621	10.69619

28.567	10.7516	10.75161	10.75159
28.57533	10.7871	10.78711	10.78709
28.58367	10.842	10.84201	10.84199
28.592	10.88225	10.88226	10.88224
28.60033	10.93015	10.93016	10.93014
28.60867	10.97385	10.97386	10.97384
28.617	11.02075	11.02076	11.02074
28.62533	11.0698	11.06981	11.06979
28.63367	11.1217	11.12171	11.12169
28.642	11.1765	11.17651	11.17649
28.65033	11.23005	11.23006	11.23004
28.65867	11.30155	11.30156	11.30154
28.667	11.36425	11.36426	11.36424
28.67533	11.42075	11.42076	11.42074
28.68367	11.47015	11.47016	11.47014
28.692	11.52485	11.52486	11.52484
28.70033	11.5597	11.55971	11.55969
28.70867	11.60425	11.60426	11.60424
28.717	11.6573	11.65731	11.65729
28.72533	11.69285	11.69286	11.69284
28.73367	11.74755	11.74756	11.74754
28.742	11.7809	11.78091	11.78089
28.75033	11.8202	11.82021	11.82019
28.75867	11.8614	11.86141	11.86139
28.767	11.90455	11.90456	11.90454
28.77533	11.9332	11.93321	11.93319
28.78367	11.98135	11.98136	11.98134
28.792	12.01445	12.01446	12.01444
28.80033	12.0562	12.05621	12.05619
28.80867	12.0988	12.09881	12.09879
28.817	12.14295	12.14296	12.14294
28.82533	12.19515	12.19516	12.19514
28.83367	12.25225	12.25226	12.25224
28.842	12.3046	12.30461	12.30459
28.85033	12.3524	12.35241	12.35239
28.85867	12.38825	12.38826	12.38824
28.867	12.42655	12.42656	12.42654
28.87533	12.4578	12.45781	12.45779
28.88367	12.4939	12.49391	12.49389
28.892	12.5177	12.51771	12.51769
28.90033	12.55295	12.55296	12.55294
28.90867	12.5777	12.57771	12.57769
28.917	12.6046	12.60461	12.60459
28.92533	12.6428	12.64281	12.64279

28.93367	12.6615	12.66151	12.66149
28.942	12.688	12.68801	12.68799
28.95033	12.7169	12.71691	12.71689
28.95867	12.74715	12.74716	12.74714
28.967	12.77555	12.77556	12.77554
28.97533	12.81555	12.81556	12.81554
28.98367	12.839	12.83901	12.83899
28.992	12.8843	12.88431	12.88429
29.00033	12.91845	12.91846	12.91844
29.00867	12.96565	12.96566	12.96564
29.017	13.01455	13.01456	13.01454
29.02533	13.0649	13.06491	13.06489
29.03367	13.10365	13.10366	13.10364
29.042	13.143	13.14301	13.14299
29.05033	13.17685	13.17686	13.17684
29.05867	13.21	13.21001	13.20999
29.067	13.24565	13.24566	13.24564
29.07533	13.272	13.27201	13.27199
29.08367	13.2959	13.29591	13.29589
29.092	13.323	13.32301	13.32299
29.10033	13.3566	13.35661	13.35659
29.10867	13.3784	13.37841	13.37839
29.117	13.41365	13.41366	13.41364
29.12533	13.4541	13.45411	13.45409
29.13367	13.47145	13.47146	13.47144
29.142	13.49175	13.49176	13.49174
29.15033	13.52635	13.52636	13.52634
29.15867	13.57935	13.57936	13.57934
29.167	13.60315	13.60316	13.60314
29.17533	13.6489	13.64891	13.64889
29.18367	13.6921	13.69211	13.69209
29.192	13.75085	13.75086	13.75084
29.20033	13.80995	13.80996	13.80994
29.20867	13.8685	13.86851	13.86849
29.217	13.9232	13.92321	13.92319
29.22533	13.961	13.96101	13.96099
29.23367	13.9917	13.99171	13.99169
29.242	13.9983	13.99831	13.99829
29.25033	14.0254	14.02541	14.02539
29.25867	14.05355	14.05356	14.05354
29.267	14.0846	14.08461	14.08459
29.27533	14.10755	14.10756	14.10754
29.28367	14.1364	14.13641	14.13639
29.292	14.16655	14.16656	14.16654

29.30033	14.2023	14.20231	14.20229
29.30867	14.23395	14.23396	14.23394
29.317	14.2685	14.26851	14.26849
29.32533	14.29205	14.29206	14.29204
29.33367	14.3358	14.33581	14.33579
29.342	14.3673	14.36731	14.36729
29.35033	14.4209	14.42091	14.42089
29.35867	14.45975	14.45976	14.45974
29.367	14.4949	14.49491	14.49489
29.37533	14.55515	14.55516	14.55514
29.38367	14.5949	14.59491	14.59489
29.392	14.6544	14.65441	14.65439
29.40033	14.716	14.71601	14.71599
29.40867	14.76215	14.76216	14.76214
29.417	14.80825	14.80826	14.80824
29.42533	14.8502	14.85021	14.85019
29.43367	14.88195	14.88196	14.88194
29.442	15.11875	15.11876	15.11874
29.45033	15.16115	15.16116	15.16114
29.45867	15.2134	15.21341	15.21339
29.467	15.24545	15.24546	15.24544
29.47533	15.28405	15.28406	15.28404
29.48367	15.3164	15.31641	15.31639
29.492	15.3486	15.34861	15.34859
29.50033	15.38895	15.38896	15.38894
29.50867	15.4229	15.42291	15.42289
29.517	15.4625	15.46251	15.46249
29.52533	15.5062	15.50621	15.50619
29.53367	15.54355	15.54356	15.54354
29.542	15.5886	15.58861	15.58859
29.55033	15.6512	15.65121	15.65119
29.55867	15.69785	15.69786	15.69784
29.567	15.76805	15.76806	15.76804
29.57533	15.83825	15.83826	15.83824
29.58367	15.91925	15.91926	15.91924
29.592	15.9939	15.99391	15.99389
29.60033	16.0578	16.05781	16.05779
29.60867	16.0975	16.09751	16.09749
29.617	16.1582	16.15821	16.15819
29.62533	16.20755	16.20756	16.20754
29.63367	16.25705	16.25706	16.25704
29.642	16.3142	16.31421	16.31419
29.65033	16.36835	16.36836	16.36834
29.65867	16.4254	16.42541	16.42539

29.667	16.4922	16.49221	16.49219
29.67533	16.55555	16.55556	16.55554
29.68367	16.6121	16.61211	16.61209
29.692	16.69455	16.69456	16.69454
29.70033	16.7458	16.74581	16.74579
29.70867	16.82455	16.82456	16.82454
29.717	16.91085	16.91086	16.91084
29.72533	16.9905	16.99051	16.99049
29.73367	17.0842	17.08421	17.08419
29.742	17.177	17.17701	17.17699
29.75033	17.25875	17.25876	17.25874
29.75867	17.355	17.35501	17.35499
29.767	17.4544	17.45441	17.45439
29.77533	17.55535	17.55536	17.55534
29.78367	17.6331	17.63311	17.63309
29.792	17.697	17.69701	17.69699
29.80033	17.73465	17.73466	17.73464
29.80867	17.78165	17.78166	17.78164
29.817	17.82925	17.82926	17.82924
29.82533	17.88295	17.88296	17.88294
29.83367	17.92825	17.92826	17.92824
29.842	17.96975	17.96976	17.96974
29.85033	18.03175	18.03176	18.03174
29.85867	18.0777	18.07771	18.07769
29.867	18.12655	18.12656	18.12654
29.87533	18.1849	18.18491	18.18489
29.88367	18.23355	18.23356	18.23354
29.892	18.27875	18.27876	18.27874
29.90033	18.3358	18.33581	18.33579
29.90867	18.4068	18.40681	18.40679
29.917	18.4589	18.45891	18.45889
29.92533	18.53185	18.53186	18.53184
29.93367	18.60665	18.60666	18.60664
29.942	18.68705	18.68706	18.68704
29.95033	18.78065	18.78066	18.78064
29.95867	18.85615	18.85616	18.85614
29.967	18.9393	18.93931	18.93929
29.97533	19.0088	19.00881	19.00879
29.98367	19.0482	19.04821	19.04819
29.992	19.0915	19.09151	19.09149
30.00033	19.1401	19.14011	19.14009
30.00867	19.1792	19.17921	19.17919
30.017	19.21735	19.21736	19.21734
30.02533	19.26345	19.26346	19.26344

30.03367	19.31195	19.31196	19.31194
30.042	19.36355	19.36356	19.36354
30.05033	19.4128	19.41281	19.41279
30.05867	19.4522	19.45221	19.45219
30.067	19.48425	19.48426	19.48424
30.07533	19.53255	19.53256	19.53254
30.08367	19.59055	19.59056	19.59054
30.092	19.8957	19.89571	19.89569
30.10033	26.48565	26.48566	26.48564
30.10867	58.97415	58.97416	58.97414
30.117	148.6287	148.62871	148.62869
30.12533	196.4911	196.49111	196.49109
30.13367	186.98375	186.98376	186.98374
30.142	174.6287	174.62871	174.62869
30.15033	157.3748	157.37481	157.37479
30.15867	116.34305	116.34306	116.34304
30.167	74.0435	74.04351	74.04349
30.17533	48.2681	48.26811	48.26809
30.18367	35.8839	35.88391	35.88389
30.192	30.82685	30.82686	30.82684
30.20033	28.47965	28.47966	28.47964
30.20867	26.9843	26.98431	26.98429
30.217	25.5686	25.56861	25.56859
30.22533	24.04465	24.04466	24.04464
30.23367	22.529	22.52901	22.52899
30.242	21.15135	21.15136	21.15134
30.25033	20.0142	20.01421	20.01419
30.25867	19.14215	19.14216	19.14214
30.267	18.48865	18.48866	18.48864
30.27533	18.0155	18.01551	18.01549
30.28367	17.65945	17.65946	17.65944
30.292	17.4039	17.40391	17.40389
30.30033	17.2299	17.22991	17.22989
30.30867	17.1015	17.10151	17.10149
30.317	17.04795	17.04796	17.04794
30.32533	17.08625	17.08626	17.08624
30.33367	17.2042	17.20421	17.20419
30.342	17.4482	17.44821	17.44819
30.35033	17.81835	17.81836	17.81834
30.35867	18.291	18.29101	18.29099
30.367	18.79925	18.79926	18.79924
30.37533	19.2393	19.23931	19.23929
30.38367	19.4994	19.49941	19.49939
30.392	19.5007	19.50071	19.50069

30.40033	19.2678	19.26781	19.26779
30.40867	18.8639	18.86391	18.86389
30.417	18.3699	18.36991	18.36989
30.42533	17.88455	17.88456	17.88454
30.43367	17.43925	17.43926	17.43924
30.442	17.09135	17.09136	17.09134
30.45033	16.82875	16.82876	16.82874
30.45867	16.64355	16.64356	16.64354
30.467	16.5013	16.50131	16.50129
30.47533	16.38505	16.38506	16.38504
30.48367	16.2815	16.28151	16.28149
30.492	16.17425	16.17426	16.17424
30.50033	16.0526	16.05261	16.05259
30.50867	15.9259	15.92591	15.92589
30.517	15.7632	15.76321	15.76319
30.52533	15.59505	15.59506	15.59504
30.53367	15.41405	15.41406	15.41404
30.542	15.2326	15.23261	15.23259
30.55033	15.0559	15.05591	15.05589
30.55867	14.9013	14.90131	14.90129
30.567	14.7638	14.76381	14.76379
30.57533	14.6386	14.63861	14.63859
30.58367	14.53255	14.53256	14.53254
30.592	14.44205	14.44206	14.44204
30.60033	14.3587	14.35871	14.35869
30.60867	14.28875	14.28876	14.28874
30.617	14.2129	14.21291	14.21289
30.62533	14.14195	14.14196	14.14194
30.63367	14.07795	14.07796	14.07794
30.642	14.02345	14.02346	14.02344
30.65033	13.9737	13.97371	13.97369
30.65867	13.93405	13.93406	13.93404
30.667	13.8957	13.89571	13.89569
30.67533	13.8683	13.86831	13.86829
30.68367	13.8606	13.86061	13.86059
30.692	13.85865	13.85866	13.85864
30.70033	13.85795	13.85796	13.85794
30.70867	13.8543	13.85431	13.85429
30.717	13.8533	13.85331	13.85329
30.72533	13.8376	13.83761	13.83759
30.73367	13.8198	13.81981	13.81979
30.742	13.8055	13.80551	13.80549
30.75033	13.80585	13.80586	13.80584
30.75867	13.81135	13.81136	13.81134

30.767	13.835	13.83501	13.83499
30.77533	13.85765	13.85766	13.85764
30.78367	13.89375	13.89376	13.89374
30.792	13.90745	13.90746	13.90744
30.80033	13.9206	13.92061	13.92059
30.80867	13.91085	13.91086	13.91084
30.817	13.8985	13.89851	13.89849
30.82533	13.88775	13.88776	13.88774
30.83367	13.88315	13.88316	13.88314
30.842	13.8964	13.89641	13.89639
30.85033	13.93385	13.93386	13.93384
30.85867	14.00135	14.00136	14.00134
30.867	14.1119	14.11191	14.11189
30.87533	14.2438	14.24381	14.24379
30.88367	14.3952	14.39521	14.39519
30.892	14.5363	14.53631	14.53629
30.90033	14.6496	14.64961	14.64959
30.90867	14.7328	14.73281	14.73279
30.917	14.73245	14.73246	14.73244
30.92533	14.6797	14.67971	14.67969
30.93367	14.57355	14.57356	14.57354
30.942	14.418	14.41801	14.41799
30.95033	14.2433	14.24331	14.24329
30.95867	14.0413	14.04131	14.04129
30.967	13.85425	13.85426	13.85424
30.97533	13.6732	13.67321	13.67319
30.98367	13.5154	13.51541	13.51539
30.992	13.378	13.37801	13.37799
31.00033	13.2763	13.27631	13.27629
31.00867	13.19425	13.19426	13.19424
31.017	13.12135	13.12136	13.12134
31.02533	13.07945	13.07946	13.07944
31.03367	13.04645	13.04646	13.04644
31.042	13.0208	13.02081	13.02079
31.05033	13.00135	13.00136	13.00134
31.05867	12.9939	12.99391	12.99389
31.067	12.99295	12.99296	12.99294
31.07533	12.99865	12.99866	12.99864
31.08367	12.98665	12.98666	12.98664
31.092	12.98535	12.98536	12.98534
31.10033	12.9591	12.95911	12.95909
31.10867	12.9352	12.93521	12.93519
31.117	12.9163	12.91631	12.91629
31.12533	12.9088	12.90881	12.90879

31.13367	12.8871	12.88711	12.88709
31.142	12.87185	12.87186	12.87184
31.15033	12.8726	12.87261	12.87259
31.15867	12.87045	12.87046	12.87044
31.167	12.86775	12.86776	12.86774
31.17533	12.87075	12.87076	12.87074
31.18367	12.8712	12.87121	12.87119
31.192	12.87695	12.87696	12.87694
31.20033	12.8864	12.88641	12.88639
31.20867	12.90365	12.90366	12.90364
31.217	12.9173	12.91731	12.91729
31.22533	12.93645	12.93646	12.93644
31.23367	12.9534	12.95341	12.95339
31.242	12.9812	12.98121	12.98119
31.25033	13.00095	13.00096	13.00094
31.25867	13.0287	13.02871	13.02869
31.267	13.0545	13.05451	13.05449
31.27533	13.0729	13.07291	13.07289
31.28367	13.09095	13.09096	13.09094
31.292	13.088	13.08801	13.08799
31.30033	13.09775	13.09776	13.09774
31.30867	13.114	13.11401	13.11399
31.317	13.12935	13.12936	13.12934
31.32533	13.1495	13.14951	13.14949
31.33367	13.1754	13.17541	13.17539
31.342	13.19755	13.19756	13.19754
31.35033	13.24995	13.24996	13.24994
31.35867	13.2838	13.28381	13.28379
31.367	13.3177	13.31771	13.31769
31.37533	13.3545	13.35451	13.35449
31.38367	13.3943	13.39431	13.39429
31.392	13.4304	13.43041	13.43039
31.40033	13.4541	13.45411	13.45409
31.40867	13.4799	13.47991	13.47989
31.417	13.50685	13.50686	13.50684
31.42533	13.5142	13.51421	13.51419
31.43367	13.51585	13.51586	13.51584
31.442	13.51155	13.51156	13.51154
31.45033	13.4827	13.48271	13.48269
31.45867	13.4443	13.44431	13.44429
31.467	13.38605	13.38606	13.38604
31.47533	13.30645	13.30646	13.30644
31.48367	13.21865	13.21866	13.21864
31.492	13.1328	13.13281	13.13279

31.50033	13.04965	13.04966	13.04964
31.50867	12.9786	12.97861	12.97859
31.517	12.91265	12.91266	12.91264
31.52533	12.86715	12.86716	12.86714
31.53367	12.8238	12.82381	12.82379
31.542	12.79395	12.79396	12.79394
31.55033	12.76935	12.76936	12.76934
31.55867	12.7454	12.74541	12.74539
31.567	12.72595	12.72596	12.72594
31.57533	12.7048	12.70481	12.70479
31.58367	12.70045	12.70046	12.70044
31.592	12.6966	12.69661	12.69659
31.60033	12.69435	12.69436	12.69434
31.60867	12.6957	12.69571	12.69569
31.617	12.69735	12.69736	12.69734
31.62533	12.70945	12.70946	12.70944
31.63367	12.7244	12.72441	12.72439
31.642	12.7352	12.73521	12.73519
31.65033	12.7453	12.74531	12.74529
31.65867	12.7496	12.74961	12.74959
31.667	12.7415	12.74151	12.74149
31.67533	12.7425	12.74251	12.74249
31.68367	12.74535	12.74536	12.74534
31.692	12.7648	12.76481	12.76479
31.70033	12.78385	12.78386	12.78384
31.70867	12.8341	12.83411	12.83409
31.717	12.8894	12.88941	12.88939
31.72533	12.94595	12.94596	12.94594
31.73367	13.0255	13.02551	13.02549
31.742	13.1082	13.10821	13.10819
31.75033	13.17185	13.17186	13.17184
31.75867	13.23175	13.23176	13.23174
31.767	13.2717	13.27171	13.27169
31.77533	13.2778	13.27781	13.27779
31.78367	13.2594	13.25941	13.25939
31.792	13.2178	13.21781	13.21779
31.80033	13.16045	13.16046	13.16044
31.80867	13.0865	13.08651	13.08649
31.817	13.01225	13.01226	13.01224
31.82533	12.9421	12.94211	12.94209
31.83367	12.8763	12.87631	12.87629
31.842	12.81465	12.81466	12.81464
31.85033	12.75575	12.75576	12.75574
31.85867	12.7071	12.70711	12.70709

31.867	12.67255	12.67256	12.67254
31.87533	12.64515	12.64516	12.64514
31.88367	12.6206	12.62061	12.62059
31.892	12.5937	12.59371	12.59369
31.90033	12.57545	12.57546	12.57544
31.90867	12.5519	12.55191	12.55189
31.917	12.5329	12.53291	12.53289
31.92533	12.50795	12.50796	12.50794
31.93367	12.48715	12.48716	12.48714
31.942	12.46495	12.46496	12.46494
31.95033	12.45175	12.45176	12.45174
31.95867	12.44225	12.44226	12.44224
31.967	12.43025	12.43026	12.43024
31.97533	12.4278	12.42781	12.42779
31.98367	12.4216	12.42161	12.42159
31.992	12.41835	12.41836	12.41834
32.00033	12.4246	12.42461	12.42459
32.00867	12.44015	12.44016	12.44014
32.017	12.449	12.44901	12.44899
32.02533	12.45895	12.45896	12.45894
32.03367	12.47195	12.47196	12.47194
32.042	12.47295	12.47296	12.47294
32.05033	12.4836	12.48361	12.48359
32.05867	12.4887	12.48871	12.48869
32.067	12.50255	12.50256	12.50254
32.07533	12.5153	12.51531	12.51529
32.08367	12.5331	12.53311	12.53309
32.092	12.5507	12.55071	12.55069
32.10033	12.56985	12.56986	12.56984
32.10867	12.591	12.59101	12.59099
32.117	12.6289	12.62891	12.62889
32.12533	12.65845	12.65846	12.65844
32.13367	12.7015	12.70151	12.70149
32.142	12.74045	12.74046	12.74044
32.15033	12.7867	12.78671	12.78669
32.15867	12.8329	12.83291	12.83289
32.167	12.8812	12.88121	12.88119
32.17533	12.9188	12.91881	12.91879
32.18367	12.9473	12.94731	12.94729
32.192	12.97345	12.97346	12.97344
32.20033	12.9814	12.98141	12.98139
32.20867	12.98365	12.98366	12.98364
32.217	12.97265	12.97266	12.97264
32.22533	12.93775	12.93776	12.93774

32.23367	12.89895	12.89896	12.89894
32.242	12.8541	12.85411	12.85409
32.25033	12.81925	12.81926	12.81924
32.25867	12.78805	12.78806	12.78804
32.267	12.7552	12.75521	12.75519
32.27533	12.72335	12.72336	12.72334
32.28367	12.70285	12.70286	12.70284
32.292	12.6839	12.68391	12.68389
32.30033	12.67165	12.67166	12.67164
32.30867	12.65825	12.65826	12.65824
32.317	12.64725	12.64726	12.64724
32.32533	12.64025	12.64026	12.64024
32.33367	12.6348	12.63481	12.63479
32.342	12.627	12.62701	12.62699
32.35033	12.62085	12.62086	12.62084
32.35867	12.61895	12.61896	12.61894
32.367	12.62185	12.62186	12.62184
32.37533	12.6217	12.62171	12.62169
32.38367	12.63795	12.63796	12.63794
32.392	12.6547	12.65471	12.65469
32.40033	12.6697	12.66971	12.66969
32.40867	12.6918	12.69181	12.69179
32.417	12.71105	12.71106	12.71104
32.42533	12.7433	12.74331	12.74329
32.43367	12.7831	12.78311	12.78309
32.442	12.8317	12.83171	12.83169
32.45033	12.88375	12.88376	12.88374
32.45867	12.93715	12.93716	12.93714
32.467	12.9855	12.98551	12.98549
32.47533	13.0223	13.02231	13.02229
32.48367	13.0472	13.04721	13.04719
32.492	13.053	13.05301	13.05299
32.50033	13.0474	13.04741	13.04739
32.50867	13.02675	13.02676	13.02674
32.517	12.9912	12.99121	12.99119
32.52533	12.9524	12.95241	12.95239
32.53367	12.90305	12.90306	12.90304
32.542	12.85255	12.85256	12.85254
32.55033	12.79655	12.79656	12.79654
32.55867	12.74605	12.74606	12.74604
32.567	12.7002	12.70021	12.70019
32.57533	12.66565	12.66566	12.66564
32.58367	12.6264	12.62641	12.62639
32.592	12.59615	12.59616	12.59614

32.60033	12.56315	12.56316	12.56314
32.60867	12.53025	12.53026	12.53024
32.617	12.501	12.50101	12.50099
32.62533	12.48	12.48001	12.47999
32.63367	12.45815	12.45816	12.45814
32.642	12.43615	12.43616	12.43614
32.65033	12.42795	12.42796	12.42794
32.65867	12.4117	12.41171	12.41169
32.667	12.40565	12.40566	12.40564
32.67533	12.39375	12.39376	12.39374
32.68367	12.3911	12.39111	12.39109
32.692	12.38635	12.38636	12.38634
32.70033	12.3864	12.38641	12.38639
32.70867	12.39375	12.39376	12.39374
32.717	12.4006	12.40061	12.40059
32.72533	12.4107	12.41071	12.41069
32.73367	12.4282	12.42821	12.42819
32.742	12.44005	12.44006	12.44004
32.75033	12.4613	12.46131	12.46129
32.75867	12.48495	12.48496	12.48494
32.767	12.50775	12.50776	12.50774
32.77533	12.5386	12.53861	12.53859
32.78367	12.5607	12.56071	12.56069
32.792	12.57945	12.57946	12.57944
32.80033	12.59865	12.59866	12.59864
32.80867	12.62535	12.62536	12.62534
32.817	12.649	12.64901	12.64899
32.82533	12.67235	12.67236	12.67234
32.83367	12.70225	12.70226	12.70224
32.842	12.72735	12.72736	12.72734
32.85033	12.74905	12.74906	12.74904
32.85867	12.7671	12.76711	12.76709
32.867	12.7761	12.77611	12.77609
32.87533	12.7843	12.78431	12.78429
32.88367	12.7957	12.79571	12.79569
32.892	12.79	12.79001	12.78999
32.90033	12.79175	12.79176	12.79174
32.90867	12.7928	12.79281	12.79279
32.917	12.7869	12.78691	12.78689
32.92533	12.78345	12.78346	12.78344
32.93367	12.7804	12.78041	12.78039
32.942	12.7821	12.78211	12.78209
32.95033	12.77925	12.77926	12.77924
32.95867	12.76505	12.76506	12.76504

32.967	12.75685	12.75686	12.75684
32.97533	12.7259	12.72591	12.72589
32.98367	12.70945	12.70946	12.70944
32.992	12.68805	12.68806	12.68804
33.00033	12.6631	12.66311	12.66309
33.00867	12.6435	12.64351	12.64349
33.017	12.6285	12.62851	12.62849
33.02533	12.61715	12.61716	12.61714
33.03367	12.60135	12.60136	12.60134
33.042	12.5923	12.59231	12.59229
33.05033	12.58155	12.58156	12.58154
33.05867	12.5774	12.57741	12.57739
33.067	12.5806	12.58061	12.58059
33.07533	12.58455	12.58456	12.58454
33.08367	12.5904	12.59041	12.59039
33.092	12.5942	12.59421	12.59419
33.10033	12.6005	12.60051	12.60049
33.10867	12.60715	12.60716	12.60714
33.117	12.61595	12.61596	12.61594
33.12533	12.62875	12.62876	12.62874
33.13367	12.6351	12.63511	12.63509
33.142	12.6389	12.63891	12.63889
33.15033	12.631	12.63101	12.63099
33.15867	12.6117	12.61171	12.61169
33.167	12.5837	12.58371	12.58369
33.17533	12.55255	12.55256	12.55254
33.18367	12.524	12.52401	12.52399
33.192	12.48555	12.48556	12.48554
33.20033	12.46305	12.46306	12.46304
33.20867	12.43545	12.43546	12.43544
33.217	12.41125	12.41126	12.41124
33.22533	12.38845	12.38846	12.38844
33.23367	12.3679	12.36791	12.36789
33.242	12.3489	12.34891	12.34889
33.25033	12.33555	12.33556	12.33554
33.25867	12.32135	12.32136	12.32134
33.267	12.31435	12.31436	12.31434
33.27533	12.3019	12.30191	12.30189
33.28367	12.3011	12.30111	12.30109
33.292	12.30015	12.30016	12.30014
33.30033	12.30295	12.30296	12.30294
33.30867	12.30155	12.30156	12.30154
33.317	12.3106	12.31061	12.31059
33.32533	12.31265	12.31266	12.31264

33.33367	12.31735	12.31736	12.31734
33.342	12.31575	12.31576	12.31574
33.35033	12.31215	12.31216	12.31214
33.35867	12.3098	12.30981	12.30979
33.367	12.307	12.30701	12.30699
33.37533	12.3026	12.30261	12.30259
33.38367	12.3027	12.30271	12.30269
33.392	12.30235	12.30236	12.30234
33.40033	12.3	12.30001	12.29999
33.40867	12.30015	12.30016	12.30014
33.417	12.3043	12.30431	12.30429
33.42533	12.3013	12.30131	12.30129
33.43367	12.2995	12.29951	12.29949
33.442	12.30355	12.30356	12.30354
33.45033	12.3118	12.31181	12.31179
33.45867	12.31585	12.31586	12.31584
33.467	12.3268	12.32681	12.32679
33.47533	12.33695	12.33696	12.33694
33.48367	12.3486	12.34861	12.34859
33.492	12.3671	12.36711	12.36709
33.50033	12.38805	12.38806	12.38804
33.50867	12.4062	12.40621	12.40619
33.517	12.4285	12.42851	12.42849
33.52533	12.44275	12.44276	12.44274
33.53367	12.45255	12.45256	12.45254
33.542	12.45325	12.45326	12.45324
33.55033	12.4545	12.45451	12.45449
33.55867	12.4575	12.45751	12.45749
33.567	12.4555	12.45551	12.45549
33.57533	12.44715	12.44716	12.44714
33.58367	12.44075	12.44076	12.44074
33.592	12.42955	12.42956	12.42954
33.60033	12.4211	12.42111	12.42109
33.60867	12.41515	12.41516	12.41514
33.617	12.4109	12.41091	12.41089
33.62533	12.40585	12.40586	12.40584
33.63367	12.41185	12.41186	12.41184
33.642	12.42055	12.42056	12.42054
33.65033	12.44145	12.44146	12.44144
33.65867	12.47105	12.47106	12.47104
33.667	12.52045	12.52046	12.52044
33.67533	12.58115	12.58116	12.58114
33.68367	12.67105	12.67106	12.67104
33.692	12.79625	12.79626	12.79624

33.70033	12.9539	12.95391	12.95389
33.70867	13.15155	13.15156	13.15154
33.717	13.4062	13.40621	13.40619
33.72533	13.7191	13.71911	13.71909
33.73367	14.11435	14.11436	14.11434
33.742	14.61595	14.61596	14.61594
33.75033	15.238	15.23801	15.23799
33.75867	16.00165	16.00166	16.00164
33.767	16.9295	16.92951	16.92949
33.77533	18.04875	18.04876	18.04874
33.78367	19.38565	19.38566	19.38564
33.792	20.941	20.94101	20.94099
33.80033	22.74295	22.74296	22.74294
33.80867	24.7745	24.77451	24.77449
33.817	27.0443	27.04431	27.04429
33.82533	29.5014	29.50141	29.50139
33.83367	32.13135	32.13136	32.13134
33.842	34.8622	34.86221	34.86219
33.85033	37.6461	37.64611	37.64609
33.85867	40.3949	40.39491	40.39489
33.867	43.0197	43.01971	43.01969
33.87533	45.44785	45.44786	45.44784
33.88367	47.56655	47.56656	47.56654
33.892	49.2635	49.26351	49.26349
33.90033	50.43615	50.43616	50.43614
33.90867	50.98265	50.98266	50.98264
33.917	50.877	50.87701	50.87699
33.92533	50.16895	50.16898	50.16894
33.93367	48.9201	48.92011	48.92009
33.942	47.20765	47.20766	47.20764
33.95033	45.0979	45.09791	45.09789
33.95867	42.712	42.71201	42.71199
33.967	40.1412	40.14121	40.14119
33.97533	37.465	37.46501	37.46499
33.98367	34.79115	34.79116	34.79114
33.992	32.1909	32.19091	32.19089
34.00033	29.7271	29.72711	29.72709
34.00867	27.44715	27.44716	27.44714
34.017	25.3878	25.38781	25.38779
34.02533	23.54495	23.54496	23.54494
34.03367	21.9348	21.93481	21.93479
34.042	20.5387	20.53871	20.53869
34.05033	19.3402	19.34021	19.34019
34.05867	18.328	18.32801	18.32799

34.067	17.48865	17.48866	17.48864
34.07533	16.7744	16.77441	16.77439
34.08367	16.176	16.17601	16.17599
34.092	15.6838	15.68381	15.68379
34.10033	15.27265	15.27266	15.27264
34.10867	14.93335	14.93336	14.93334
34.117	14.6579	14.65791	14.65789
34.12533	14.4314	14.43141	14.43139
34.13367	14.24125	14.24126	14.24124
34.142	14.0773	14.07731	14.07729
34.15033	13.94505	13.94506	13.94504
34.15867	13.82725	13.82726	13.82724
34.167	13.71995	13.71996	13.71994
34.17533	13.62755	13.62756	13.62754
34.18367	13.54575	13.54576	13.54574
34.192	13.4662	13.46621	13.46619
34.20033	13.4002	13.40021	13.40019
34.20867	13.34195	13.34196	13.34194
34.217	13.28355	13.28356	13.28354
34.22533	13.233	13.23301	13.23299
34.23367	13.18785	13.18786	13.18784
34.242	13.1479	13.14791	13.14789
34.25033	13.11605	13.11606	13.11604
34.25867	13.0873	13.08731	13.08729
34.267	13.05585	13.05586	13.05584
34.27533	13.0262	13.02621	13.02619
34.28367	12.9951	12.99511	12.99509
34.292	12.95805	12.95806	12.95804
34.30033	12.92385	12.92386	12.92384
34.30867	12.90175	12.90176	12.90174
34.317	12.87285	12.87286	12.87284
34.32533	12.84475	12.84476	12.84474
34.33367	12.8223	12.82231	12.82229
34.342	12.80115	12.80116	12.80114
34.35033	12.78275	12.78276	12.78274
34.35867	12.7566	12.75661	12.75659
34.367	12.7414	12.74141	12.74139
34.37533	12.72355	12.72356	12.72354
34.38367	12.71085	12.71086	12.71084
34.392	12.7018	12.70181	12.70179
34.40033	12.6927	12.69271	12.69269
34.40867	12.6875	12.68751	12.68749
34.417	12.6843	12.68431	12.68429
34.42533	12.67885	12.67886	12.67884

34.43367	12.6814	12.68141	12.68139
34.442	12.69	12.69001	12.68999
34.45033	12.69515	12.69516	12.69514
34.45867	12.70495	12.70496	12.70494
34.467	12.7102	12.71021	12.71019
34.47533	12.7048	12.70481	12.70479
34.48367	12.697	12.69701	12.69699
34.492	12.6974	12.69741	12.69739
34.50033	12.69325	12.69326	12.69324
34.50867	12.6898	12.68981	12.68979
34.517	12.68705	12.68706	12.68704
34.52533	12.6793	12.67931	12.67929
34.53367	12.67175	12.67176	12.67174
34.542	12.65805	12.65806	12.65804
34.55033	12.6494	12.64941	12.64939
34.55867	12.6324	12.63241	12.63239
34.567	12.6165	12.61651	12.61649
34.57533	12.6038	12.60381	12.60379
34.58367	12.5941	12.59411	12.59409
34.592	12.57655	12.57656	12.57654
34.60033	12.56495	12.56496	12.56494
34.60867	12.55575	12.55576	12.55574
34.617	12.5475	12.54751	12.54749
34.62533	12.54235	12.54236	12.54234
34.63367	12.5376	12.53761	12.53759
34.642	12.53595	12.53596	12.53594
34.65033	12.5305	12.53051	12.53049
34.65867	12.51585	12.51586	12.51584
34.667	12.5023	12.50231	12.50229
34.67533	12.49255	12.49256	12.49254
34.68367	12.48225	12.48226	12.48224
34.692	12.47465	12.47466	12.47464
34.70033	12.46165	12.46166	12.46164
34.70867	12.45905	12.45906	12.45904
34.717	12.4497	12.44971	12.44969
34.72533	12.44185	12.44186	12.44184
34.73367	12.43415	12.43416	12.43414
34.742	12.4253	12.42531	12.42529
34.75033	12.41695	12.41696	12.41694
34.75867	12.41085	12.41086	12.41084
34.767	12.4118	12.41181	12.41179
34.77533	12.401	12.40101	12.40099
34.78367	12.3993	12.39931	12.39929
34.792	12.39665	12.39666	12.39664

34.80033	12.3957	12.39571	12.39569
34.80867	12.39685	12.39686	12.39684
34.817	12.406	12.40601	12.40599
34.82533	12.406	12.40601	12.40599
34.83367	12.41425	12.41426	12.41424
34.842	12.4066	12.40661	12.40659
34.85033	12.4033	12.40331	12.40329
34.85867	12.3977	12.39771	12.39769
34.867	12.39425	12.39426	12.39424
34.87533	12.3928	12.39281	12.39279
34.88367	12.3872	12.38721	12.38719
34.892	12.38765	12.38766	12.38764
34.90033	12.38225	12.38226	12.38224
34.90867	12.37935	12.37936	12.37934
34.917	12.37545	12.37546	12.37544
34.92533	12.3713	12.37131	12.37129
34.93367	12.3625	12.36251	12.36249
34.942	12.35935	12.35936	12.35934
34.95033	12.3564	12.35641	12.35639
34.95867	12.35375	12.35376	12.35374
34.967	12.3548	12.35481	12.35479
34.97533	12.35585	12.35586	12.35584
34.98367	12.3606	12.36061	12.36059
34.992	12.3542	12.35421	12.35419
35.00033	12.3682	12.36821	12.36819
35.00867	12.36805	12.36806	12.36804
35.017	12.37225	12.37226	12.37224
35.02533	12.38005	12.38006	12.38004
35.03367	12.3736	12.37361	12.37359
35.042	12.3694	12.36941	12.36939
35.05033	12.3615	12.36151	12.36149
35.05867	12.362	12.36201	12.36199
35.067	12.35515	12.35516	12.35514
35.07533	12.3515	12.35151	12.35149
35.08367	12.351	12.35101	12.35099
35.092	12.34775	12.34776	12.34774
35.10033	12.34085	12.34086	12.34084
35.10867	12.3383	12.33831	12.33829
35.117	12.33405	12.33406	12.33404
35.12533	12.3334	12.33341	12.33339
35.13367	12.33135	12.33136	12.33134
35.142	12.32705	12.32706	12.32704
35.15033	12.32665	12.32666	12.32664
35.15867	12.32905	12.32906	12.32904

35.167	12.33	12.33001	12.32999
35.17533	12.3347	12.33471	12.33469
35.18367	12.3404	12.34041	12.34039
35.192	12.3577	12.35771	12.35769
35.20033	12.3595	12.35951	12.35949
35.20867	12.37	12.37001	12.36999
35.217	12.36605	12.36606	12.36604
35.22533	12.3605	12.36051	12.36049
35.23367	12.35935	12.35936	12.35934
35.242	12.3622	12.36221	12.36219
35.25033	12.35605	12.35606	12.35604
35.25867	12.35085	12.35086	12.35084
35.267	12.3498	12.34981	12.34979
35.27533	12.35305	12.35306	12.35304
35.28367	12.3487	12.34871	12.34869
35.292	12.34765	12.34766	12.34764
35.30033	12.34775	12.34776	12.34774
35.30867	12.3372	12.33721	12.33719
35.317	12.33345	12.33346	12.33344
35.32533	12.33025	12.33026	12.33024
35.33367	12.33135	12.33136	12.33134
35.342	12.3347	12.33471	12.33469
35.35033	12.3375	12.33751	12.33749
35.35867	12.3409	12.34091	12.34089
35.367	12.34345	12.34346	12.34344
35.37533	12.35675	12.35676	12.35674
35.38367	12.35955	12.35956	12.35954
35.392	12.3665	12.36651	12.36649
35.40033	12.3726	12.37261	12.37259
35.40867	12.36995	12.36996	12.36994
35.417	12.367	12.36701	12.36699
35.42533	12.36585	12.36586	12.36584
35.43367	12.3635	12.36351	12.36349
35.442	12.3657	12.36571	12.36569
35.45033	12.35895	12.35896	12.35894
35.45867	12.36095	12.36096	12.36094
35.467	12.36375	12.36376	12.36374
35.47533	12.3608	12.36081	12.36079
35.48367	12.3582	12.35821	12.35819
35.492	12.35915	12.35916	12.35914
35.50033	12.35655	12.35656	12.35654
35.50867	12.3574	12.35741	12.35739
35.517	12.3588	12.35881	12.35879
35.52533	12.3582	12.35821	12.35819

35.53367	12.36485	12.36486	12.36484
35.542	12.36845	12.36846	12.36844
35.55033	12.38175	12.38176	12.38174
35.55867	12.3901	12.39011	12.39009
35.567	12.40295	12.40296	12.40294
35.57533	12.41355	12.41356	12.41354
35.58367	12.42105	12.42106	12.42104
35.592	12.42645	12.42646	12.42644
35.60033	12.4247	12.42471	12.42469
35.60867	12.4228	12.42281	12.42279
35.617	12.42865	12.42866	12.42864
35.62533	12.4321	12.43211	12.43209
35.63367	12.434	12.43401	12.43399
35.642	12.4405	12.44051	12.44049
35.65033	12.4372	12.43721	12.43719
35.65867	12.44495	12.44496	12.44494
35.667	12.4427	12.44271	12.44269
35.67533	12.44415	12.44416	12.44414
35.68367	12.44685	12.44686	12.44684
35.692	12.45205	12.45206	12.45204
35.70033	12.45855	12.45856	12.45854
35.70867	12.46715	12.46716	12.46714
35.717	12.4687	12.46871	12.46869
35.72533	12.48465	12.48466	12.48464
35.73367	12.49255	12.49256	12.49254
35.742	12.50975	12.50976	12.50974
35.75033	12.53535	12.53536	12.53534
35.75867	12.55125	12.55126	12.55124
35.767	12.57335	12.57336	12.57334
35.77533	12.5958	12.59581	12.59579
35.78367	12.6174	12.61741	12.61739
35.792	12.6382	12.63821	12.63819
35.80033	12.66065	12.66066	12.66064
35.80867	12.6939	12.69391	12.69389
35.817	12.72055	12.72056	12.72054
35.82533	12.7598	12.75981	12.75979
35.83367	12.7991	12.79911	12.79909
35.842	12.83235	12.83236	12.83234
35.85033	12.87655	12.87656	12.87654
35.85867	12.91735	12.91736	12.91734
35.867	12.95875	12.95876	12.95874
35.87533	12.9962	12.99621	12.99619
35.88367	13.0366	13.03661	13.03659
35.892	13.0791	13.07911	13.07909

35.90033	13.1111	13.11111	13.11109
35.90867	13.1477	13.14771	13.14769
35.917	13.17935	13.17936	13.17934
35.92533	13.20775	13.20776	13.20774
35.93367	13.23045	13.23046	13.23044
35.942	13.24665	13.24666	13.24664
35.95033	13.2621	13.26211	13.26209
35.95867	13.2597	13.25971	13.25969
35.967	13.2494	13.24941	13.24939
35.97533	13.22705	13.22706	13.22704
35.98367	13.1946	13.19461	13.19459
35.992	13.1649	13.16491	13.16489
36.00033	13.1289	13.12891	13.12889
36.00867	13.0851	13.08511	13.08509
36.017	13.05215	13.05216	13.05214
36.02533	13.01305	13.01306	13.01304
36.03367	12.96965	12.96966	12.96964
36.042	12.9358	12.93581	12.93579
36.05033	12.89655	12.89656	12.89654
36.05867	12.86725	12.86726	12.86724
36.067	12.83595	12.83596	12.83594
36.07533	12.81155	12.81156	12.81154
36.08367	12.7861	12.78611	12.78609
36.092	12.77075	12.77076	12.77074
36.10033	12.758	12.75801	12.75799
36.10867	12.7404	12.74041	12.74039
36.117	12.72935	12.72936	12.72934
36.12533	12.72455	12.72456	12.72454
36.13367	12.7212	12.72121	12.72119
36.142	12.71885	12.71886	12.71884
36.15033	12.706	12.70601	12.70599
36.15867	12.69195	12.69196	12.69194
36.167	12.6665	12.66651	12.66649
36.17533	12.6505	12.65051	12.65049
36.18367	12.6342	12.63421	12.63419
36.192	12.61835	12.61836	12.61834
36.20033	12.5937	12.59371	12.59369
36.20867	12.57195	12.57196	12.57194
36.217	12.55365	12.55366	12.55364
36.22533	12.52675	12.52676	12.52674
36.23367	12.5029	12.50291	12.50289
36.242	12.4862	12.48621	12.48619
36.25033	12.455	12.45501	12.45499
36.25867	12.44135	12.44136	12.44134

36.267	12.42125	12.42126	12.42124
36.27533	12.4042	12.40421	12.40419
36.28367	12.38235	12.38236	12.38234
36.292	12.3802	12.38021	12.38019
36.30033	12.36855	12.36856	12.36854
36.30867	12.36505	12.36506	12.36504
36.317	12.35925	12.35926	12.35924
36.32533	12.3589	12.35891	12.35889
36.33367	12.35625	12.35626	12.35624
36.342	12.34145	12.34146	12.34144
36.35033	12.3302	12.33021	12.33019
36.35867	12.3227	12.32271	12.32269
36.367	12.3183	12.31831	12.31829
36.37533	12.31055	12.31056	12.31054
36.38367	12.30145	12.30146	12.30144
36.392	12.29695	12.29696	12.29694
36.40033	12.294	12.29401	12.29399
36.40867	12.28395	12.28396	12.28394
36.417	12.28185	12.28186	12.28184
36.42533	12.2792	12.27921	12.27919
36.43367	12.27105	12.27106	12.27104
36.442	12.26735	12.26736	12.26734
36.45033	12.26545	12.26546	12.26544
36.45867	12.26355	12.26356	12.26354
36.467	12.2603	12.26031	12.26029
36.47533	12.2627	12.26271	12.26269
36.48367	12.2692	12.26921	12.26919
36.492	12.2708	12.27081	12.27079
36.50033	12.2849	12.28491	12.28489
36.50867	12.29025	12.29026	12.29024
36.517	12.2943	12.29431	12.29429
36.52533	12.29545	12.29546	12.29544
36.53367	12.2903	12.29031	12.29029
36.542	12.2896	12.28961	12.28959
36.55033	12.2828	12.28281	12.28279
36.55867	12.2888	12.28881	12.28879
36.567	12.2869	12.28691	12.28689
36.57533	12.27895	12.27896	12.27894
36.58367	12.27555	12.27556	12.27554
36.592	12.2738	12.27381	12.27379
36.60033	12.26465	12.26466	12.26464
36.60867	12.2665	12.26651	12.26649
36.617	12.25535	12.25536	12.25534
36.62533	12.2524	12.25241	12.25239

36.63367	12.2512	12.25121	12.25119
36.642	12.24925	12.24926	12.24924
36.65033	12.2488	12.24881	12.24879
36.65867	12.252	12.25201	12.25199
36.667	12.25875	12.25876	12.25874
36.67533	12.25705	12.25706	12.25704
36.68367	12.27115	12.27116	12.27114
36.692	12.2807	12.28071	12.28069
36.70033	12.27875	12.27876	12.27874
36.70867	12.278	12.27801	12.27799
36.717	12.28365	12.28366	12.28364
36.72533	12.275	12.27501	12.27499
36.73367	12.27615	12.27616	12.27614
36.742	12.2698	12.26981	12.26979
36.75033	12.2696	12.26961	12.26959
36.75867	12.2712	12.27121	12.27119
36.767	12.2671	12.26711	12.26709
36.77533	12.26565	12.26566	12.26564
36.78367	12.2627	12.26271	12.26269
36.792	12.2642	12.26421	12.26419
36.80033	12.2536	12.25361	12.25359
36.80867	12.25255	12.25256	12.25254
36.817	12.2529	12.25291	12.25289
36.82533	12.2512	12.25121	12.25119
36.83367	12.2509	12.25091	12.25089
36.842	12.25645	12.25646	12.25644
36.85033	12.26735	12.26736	12.26734
36.85867	12.27085	12.27086	12.27084
36.867	12.27575	12.27576	12.27574
36.87533	12.28285	12.28286	12.28284
36.88367	12.2877	12.28771	12.28769
36.892	12.29225	12.29226	12.29224
36.90033	12.2979	12.29791	12.29789
36.90867	12.29675	12.29676	12.29674
36.917	12.2935	12.29351	12.29349
36.92533	12.29385	12.29386	12.29384
36.93367	12.2905	12.29051	12.29049
36.942	12.29155	12.29156	12.29154
36.95033	12.29335	12.29336	12.29334
36.95867	12.2919	12.29191	12.29189
36.967	12.29425	12.29426	12.29424
36.97533	12.2914	12.29141	12.29139
36.98367	12.2938	12.29381	12.29379
36.992	12.29315	12.29316	12.29314

37.00033	12.2944	12.29441	12.29439
37.00867	12.28645	12.28646	12.28644
37.017	12.2968	12.29681	12.29679
37.02533	12.293	12.29301	12.29299
37.03367	12.29575	12.29576	12.29574
37.042	12.30145	12.30146	12.30144
37.05033	12.30175	12.30176	12.30174
37.05867	12.31765	12.31766	12.31764
37.067	12.3237	12.32371	12.32369
37.07533	12.32305	12.32306	12.32304
37.08367	12.3307	12.33071	12.33069
37.092	12.32685	12.32686	12.32684
37.10033	12.325	12.32501	12.32499
37.10867	12.32035	12.32036	12.32034
37.117	12.31505	12.31506	12.31504
37.12533	12.30825	12.30826	12.30824
37.13367	12.3053	12.30531	12.30529
37.142	12.29855	12.29856	12.29854
37.15033	12.3016	12.30161	12.30159
37.15867	12.29445	12.29446	12.29444
37.167	12.2878	12.28781	12.28779
37.17533	12.27865	12.27866	12.27864
37.18367	12.27635	12.27636	12.27634
37.192	12.27205	12.27206	12.27204
37.20033	12.26905	12.26906	12.26904
37.20867	12.26525	12.26526	12.26524
37.217	12.26335	12.26336	12.26334
37.22533	12.26305	12.26306	12.26304
37.23367	12.2647	12.26471	12.26469
37.242	12.2757	12.27571	12.27569
37.25033	12.2758	12.27581	12.27579
37.25867	12.287	12.28701	12.28699
37.267	12.2885	12.28851	12.28849
37.27533	12.2911	12.29111	12.29109
37.28367	12.28805	12.28806	12.28804
37.292	12.28435	12.28436	12.28434
37.30033	12.27945	12.27946	12.27944
37.30867	12.27935	12.27936	12.27934
37.317	12.2818	12.28181	12.28179
37.32533	12.28355	12.28356	12.28354
37.33367	12.28065	12.28066	12.28064
37.342	12.28415	12.28416	12.28414
37.35033	12.27755	12.27756	12.27754
37.35867	12.27465	12.27466	12.27464

37.367	12.27775	12.27776	12.27774
37.37533	12.278	12.27801	12.27799
37.38367	12.27705	12.27706	12.27704
37.392	12.2753	12.27531	12.27529
37.40033	12.27615	12.27616	12.27614
37.40867	12.2812	12.28121	12.28119
37.417	12.285	12.28501	12.28499
37.42533	12.27965	12.27966	12.27964
37.43367	12.29025	12.29026	12.29024
37.442	12.3033	12.30331	12.30329
37.45033	12.30965	12.30966	12.30964
37.45867	12.31405	12.31406	12.31404
37.467	12.309	12.30901	12.30899
37.47533	12.3034	12.30341	12.30339
37.48367	12.30355	12.30356	12.30354
37.492	12.2997	12.29971	12.29969
37.50033	12.29815	12.29816	12.29814
37.50867	12.29395	12.29396	12.29394
37.517	12.29675	12.29676	12.29674
37.52533	12.2928	12.29281	12.29279
37.53367	12.2887	12.28871	12.28869
37.542	12.28495	12.28496	12.28494
37.55033	12.28715	12.28716	12.28714
37.55867	12.2844	12.28441	12.28439
37.567	12.2839	12.28391	12.28389
37.57533	12.28705	12.28706	12.28704
37.58367	12.28345	12.28346	12.28344
37.592	12.2891	12.28911	12.28909
37.60033	12.2954	12.29541	12.29539
37.60867	12.30215	12.30216	12.30214
37.617	12.31445	12.31446	12.31444
37.62533	12.3249	12.32491	12.32489
37.63367	12.3352	12.33521	12.33519
37.642	12.338	12.33801	12.33799
37.65033	12.34675	12.34676	12.34674
37.65867	12.34365	12.34366	12.34364
37.667	12.34375	12.34376	12.34374
37.67533	12.34845	12.34846	12.34844
37.68367	12.34745	12.34746	12.34744
37.692	12.34655	12.34656	12.34654
37.70033	12.355	12.35501	12.35499
37.70867	12.3516	12.35161	12.35159
37.717	12.35145	12.35146	12.35144
37.72533	12.3547	12.35471	12.35469

37.73367	12.35155	12.35156	12.35154
37.742	12.35105	12.35106	12.35104
37.75033	12.35455	12.35456	12.35454
37.75867	12.34875	12.34876	12.34874
37.767	12.34285	12.34286	12.34284
37.77533	12.3515	12.35151	12.35149
37.78367	12.3431	12.34311	12.34309
37.792	12.35845	12.35846	12.35844
37.80033	12.36355	12.36356	12.36354
37.80867	12.3737	12.37371	12.37369
37.817	12.3792	12.37921	12.37919
37.82533	12.39245	12.39246	12.39244
37.83367	12.39665	12.39666	12.39664
37.842	12.39365	12.39366	12.39364
37.85033	12.39375	12.39376	12.39374
37.85867	12.3921	12.39211	12.39209
37.867	12.4023	12.40231	12.40229
37.87533	12.4032	12.40321	12.40319
37.88367	12.40135	12.40136	12.40134
37.892	12.41715	12.41716	12.41714
37.90033	12.42415	12.42416	12.42414
37.90867	12.42395	12.42396	12.42394
37.917	12.435	12.43501	12.43499
37.92533	12.4367	12.43671	12.43669
37.93367	12.44805	12.44806	12.44804
37.942	12.4518	12.45181	12.45179
37.95033	12.45595	12.45596	12.45594
37.95867	12.4594	12.45941	12.45939
37.967	12.47735	12.47736	12.47734
37.97533	12.4808	12.48081	12.48079
37.98367	12.4934	12.49341	12.49339
37.992	12.50695	12.50696	12.50694
38.00033	12.52365	12.52366	12.52364
38.00867	12.52915	12.52916	12.52914
38.017	12.53615	12.53616	12.53614
38.02533	12.53105	12.53106	12.53104
38.03367	12.53345	12.53346	12.53344
38.042	12.52415	12.52416	12.52414
38.05033	12.5191	12.51911	12.51909
38.05867	12.5147	12.51471	12.51469
38.067	12.50475	12.50476	12.50474
38.07533	12.4949	12.49491	12.49489
38.08367	12.4934	12.49341	12.49339
38.092	12.4741	12.47411	12.47409

38.10033	12.46325	12.46326	12.46324
38.10867	12.4512	12.45121	12.45119
38.117	12.43625	12.43626	12.43624
38.12533	12.42785	12.42786	12.42784
38.13367	12.42245	12.42246	12.42244
38.142	12.41505	12.41506	12.41504
38.15033	12.40285	12.40286	12.40284
38.15867	12.3976	12.39761	12.39759
38.167	12.39435	12.39436	12.39434
38.17533	12.39355	12.39356	12.39354
38.18367	12.39865	12.39866	12.39864
38.192	12.40105	12.40106	12.40104
38.20033	12.4045	12.40451	12.40449
38.20867	12.4066	12.40661	12.40659
38.217	12.3978	12.39781	12.39779
38.22533	12.39235	12.39236	12.39234
38.23367	12.3926	12.39261	12.39259
38.242	12.3858	12.38581	12.38579
38.25033	12.3864	12.38641	12.38639
38.25867	12.378	12.37801	12.37799
38.267	12.3814	12.38141	12.38139
38.27533	12.3739	12.37391	12.37389
38.28367	12.3663	12.36631	12.36629
38.292	12.3653	12.36531	12.36529
38.30033	12.3501	12.35011	12.35009
38.30867	12.34435	12.34436	12.34434
38.317	12.3428	12.34281	12.34279
38.32533	12.3386	12.33861	12.33859
38.33367	12.3366	12.33661	12.33659
38.342	12.3378	12.33781	12.33779
38.35033	12.3355	12.33551	12.33549
38.35867	12.3364	12.33641	12.33639
38.367	12.34155	12.34156	12.34154
38.37533	12.3503	12.35031	12.35029
38.38367	12.34955	12.34956	12.34954
38.392	12.3525	12.35251	12.35249
38.40033	12.35325	12.35326	12.35324
38.40867	12.3426	12.34261	12.34259
38.417	12.3441	12.34411	12.34409
38.42533	12.3437	12.34371	12.34369
38.43367	12.33135	12.33136	12.33134
38.442	12.3259	12.32591	12.32589
38.45033	12.32005	12.32006	12.32004
38.45867	12.31825	12.31826	12.31824

38.467	12.31465	12.31466	12.31464
38.47533	12.3067	12.30671	12.30669
38.48367	12.30625	12.30626	12.30624
38.492	12.30275	12.30276	12.30274
38.50033	12.29935	12.29936	12.29934
38.50867	12.30105	12.30106	12.30104
38.517	12.2997	12.29971	12.29969
38.52533	12.3045	12.30451	12.30449
38.53367	12.30845	12.30846	12.30844
38.542	12.3124	12.31241	12.31239
38.55033	12.3145	12.31451	12.31449
38.55867	12.32525	12.32526	12.32524
38.567	12.32975	12.32976	12.32974
38.57533	12.34075	12.34076	12.34074
38.58367	12.3434	12.34341	12.34339
38.592	12.3443	12.34431	12.34429
38.60033	12.3422	12.34221	12.34219
38.60867	12.3388	12.33881	12.33879
38.617	12.34315	12.34316	12.34314
38.62533	12.34205	12.34206	12.34204
38.63367	12.33345	12.33346	12.33344
38.642	12.34095	12.34096	12.34094
38.65033	12.3428	12.34281	12.34279
38.65867	12.3392	12.33921	12.33919
38.667	12.341	12.34101	12.34099
38.67533	12.34025	12.34026	12.34024
38.68367	12.3342	12.33421	12.33419
38.692	12.33565	12.33566	12.33564
38.70033	12.3325	12.33251	12.33249
38.70867	12.33785	12.33786	12.33784
38.717	12.33675	12.33676	12.33674
38.72533	12.34185	12.34186	12.34184
38.73367	12.3472	12.34721	12.34719
38.742	12.35475	12.35476	12.35474
38.75033	12.3669	12.36691	12.36689
38.75867	12.3702	12.37021	12.37019
38.767	12.38105	12.38106	12.38104
38.77533	12.38005	12.38006	12.38004
38.78367	12.36495	12.36496	12.36494
38.792	12.36575	12.36576	12.36574
38.80033	12.37015	12.37016	12.37014
38.80867	12.3715	12.37151	12.37149
38.817	12.3747	12.37471	12.37469
38.82533	12.3724	12.37241	12.37239

38.83367	12.36495	12.36496	12.36494
38.842	12.36155	12.36156	12.36154
38.85033	12.36125	12.36126	12.36124
38.85867	12.35385	12.35386	12.35384
38.867	12.3541	12.35411	12.35409
38.87533	12.34955	12.34956	12.34954
38.88367	12.35265	12.35266	12.35264
38.892	12.34555	12.34556	12.34554
38.90033	12.3457	12.34571	12.34569
38.90867	12.3583	12.35831	12.35829
38.917	12.35615	12.35616	12.35614
38.92533	12.36525	12.36526	12.36524
38.93367	12.37065	12.37066	12.37064
38.942	12.37145	12.37146	12.37144
38.95033	12.3788	12.37881	12.37879
38.95867	12.3829	12.38291	12.38289
38.967	12.38235	12.38236	12.38234
38.97533	12.37035	12.37036	12.37034
38.98367	12.3737	12.37371	12.37369
38.992	12.37185	12.37186	12.37184
39.00033	12.3678	12.36781	12.36779
39.00867	12.36745	12.36746	12.36744
39.017	12.36805	12.36806	12.36804
39.02533	12.3591	12.35911	12.35909
39.03367	12.3584	12.35841	12.35839
39.042	12.35535	12.35536	12.35534
39.05033	12.34805	12.34806	12.34804
39.05867	12.3496	12.34961	12.34959
39.067	12.3357	12.33571	12.33569
39.07533	12.34575	12.34576	12.34574
39.08367	12.33825	12.33826	12.33824
39.092	12.34095	12.34096	12.34094
39.10033	12.337	12.33701	12.33699
39.10867	12.34075	12.34076	12.34074
39.117	12.3562	12.35621	12.35619
39.12533	12.35955	12.35956	12.35954
39.13367	12.36195	12.36196	12.36194
39.142	12.3639	12.36391	12.36389
39.15033	12.35795	12.35796	12.35794
39.15867	12.35245	12.35246	12.35244
39.167	12.34935	12.34936	12.34934
39.17533	12.33915	12.33916	12.33914
39.18367	12.337	12.33701	12.33699
39.192	12.33955	12.33956	12.33954

39.20033	12.3311	12.33111	12.33109
39.20867	12.33085	12.33086	12.33084
39.217	12.32875	12.32876	12.32874
39.22533	12.3245	12.32451	12.32449
39.23367	12.3146	12.31461	12.31459
39.242	12.31515	12.31516	12.31514
39.25033	12.30255	12.30256	12.30254
39.25867	12.29935	12.29936	12.29934
39.267	12.29825	12.29826	12.29824
39.27533	12.29365	12.29366	12.29364
39.28367	12.29455	12.29456	12.29454
39.292	12.30035	12.30036	12.30034
39.30033	12.3043	12.30431	12.30429
39.30867	12.3099	12.30991	12.30989
39.317	12.3066	12.30661	12.30659
39.32533	12.3091	12.30911	12.30909
39.33367	12.30795	12.30796	12.30794
39.342	12.3065	12.30651	12.30649
39.35033	12.2983	12.29831	12.29829
39.35867	12.2972	12.29721	12.29719
39.367	12.29205	12.29206	12.29204
39.37533	12.28435	12.28436	12.28434
39.38367	12.294	12.29401	12.29399
39.392	12.29055	12.29056	12.29054
39.40033	12.27715	12.27716	12.27714
39.40867	12.28675	12.28676	12.28674
39.417	12.28035	12.28036	12.28034
39.42533	12.2822	12.28221	12.28219
39.43367	12.2795	12.27951	12.27949
39.442	12.2729	12.27291	12.27289
39.45033	12.2761	12.27611	12.27609
39.45867	12.27295	12.27296	12.27294
39.467	12.27705	12.27706	12.27704
39.47533	12.2854	12.28541	12.28539
39.48367	12.28715	12.28716	12.28714
39.492	12.29455	12.29456	12.29454
39.50033	12.30605	12.30606	12.30604
39.50867	12.3124	12.31241	12.31239
39.517	12.3209	12.32091	12.32089
39.52533	12.3188	12.31881	12.31879
39.53367	12.3158	12.31581	12.31579
39.542	12.321	12.32101	12.32099
39.55033	12.3042	12.30421	12.30419
39.55867	12.30765	12.30766	12.30764

39.567	12.3141	12.31411	12.31409
39.57533	12.3195	12.31951	12.31949
39.58367	12.3257	12.32571	12.32569
39.592	12.32745	12.32746	12.32744
39.60033	12.3154	12.31541	12.31539
39.60867	12.30845	12.30846	12.30844
39.617	12.30165	12.30166	12.30164
39.62533	12.30925	12.30926	12.30924
39.63367	12.31425	12.31426	12.31424
39.642	12.3142	12.31421	12.31419
39.65033	12.3196	12.31961	12.31959
39.65867	12.32585	12.32586	12.32584
39.667	12.3361	12.33611	12.33609
39.67533	12.33995	12.33996	12.33994
39.68367	12.3525	12.35251	12.35249
39.692	12.3594	12.35941	12.35939
39.70033	12.3692	12.36921	12.36919
39.70867	12.3742	12.37421	12.37419
39.717	12.37725	12.37726	12.37724
39.72533	12.3781	12.37811	12.37809
39.73367	12.38295	12.38296	12.38294
39.742	12.38415	12.38416	12.38414
39.75033	12.38595	12.38596	12.38594
39.75867	12.40095	12.40096	12.40094
39.767	12.42335	12.42336	12.42334
39.77533	12.44505	12.44506	12.44504
39.78367	12.473	12.47301	12.47299
39.792	12.50735	12.50736	12.50734
39.80033	12.53275	12.53276	12.53274
39.80867	12.56425	12.56426	12.56424
39.817	12.57745	12.57746	12.57744
39.82533	12.56955	12.56956	12.56954
39.83367	12.56085	12.56086	12.56084
39.842	12.5418	12.54181	12.54179
39.85033	12.53635	12.53636	12.53634
39.85867	12.50765	12.50766	12.50764
39.867	12.5012	12.50121	12.50119
39.87533	12.47965	12.47966	12.47964
39.88367	12.4743	12.47431	12.47429
39.892	12.455	12.45501	12.45499
39.90033	12.4385	12.43851	12.43849
39.90867	12.42035	12.42036	12.42034
39.917	12.40205	12.40206	12.40204
39.92533	12.40145	12.40146	12.40144

39.93367	12.3846	12.38461	12.38459
39.942	12.37665	12.37666	12.37664
39.95033	12.36805	12.36806	12.36804
39.95867	12.3659	12.36591	12.36589
39.967	12.3535	12.35351	12.35349
39.97533	12.3423	12.34231	12.34229
39.98367	12.33925	12.33926	12.33924
39.992	12.3339	12.33391	12.33389
40.00033	12.3264	12.32641	12.32639
40.00867	12.32225	12.32226	12.32224
40.017	12.32395	12.32396	12.32394
40.02533	12.31635	12.31636	12.31634
40.03367	12.32135	12.32136	12.32134
40.042	12.3167	12.31671	12.31669
40.05033	12.3238	12.32381	12.32379
40.05867	12.3306	12.33061	12.33059
40.067	12.33715	12.33716	12.33714
40.07533	12.3389	12.33891	12.33889
40.08367	12.33815	12.33816	12.33814
40.092	12.3309	12.33091	12.33089
40.10033	12.32065	12.32066	12.32064
40.10867	12.31655	12.31656	12.31654
40.117	12.31465	12.31466	12.31464
40.12533	12.3091	12.30911	12.30909
40.13367	12.3079	12.30791	12.30789
40.142	12.29225	12.29226	12.29224
40.15033	12.301	12.30101	12.30099
40.15867	12.2873	12.28731	12.28729
40.167	12.27805	12.27806	12.27804
40.17533	12.28365	12.28366	12.28364
40.18367	12.27415	12.27416	12.27414
40.192	12.26835	12.26836	12.26834
40.20033	12.2716	12.27161	12.27159
40.20867	12.26735	12.26736	12.26734
40.217	12.2707	12.27071	12.27069
40.22533	12.27105	12.27106	12.27104
40.23367	12.27245	12.27246	12.27244
40.242	12.28215	12.28216	12.28214
40.25033	12.2847	12.28471	12.28469
40.25867	12.29495	12.29496	12.29494
40.267	12.2972	12.29721	12.29719
40.27533	12.2845	12.28451	12.28449
40.28367	12.27215	12.27216	12.27214
40.292	12.2788	12.27881	12.27879

40.30033	12.2739	12.27391	12.27389
40.30867	12.26095	12.26096	12.26094
40.317	12.2573	12.25731	12.25729
40.32533	12.263	12.26301	12.26299
40.33367	12.26265	12.26266	12.26264
40.342	12.25575	12.25576	12.25574
40.35033	12.2507	12.25071	12.25069
40.35867	12.2516	12.25161	12.25159
40.367	12.244	12.24401	12.24399
40.37533	12.2408	12.24081	12.24079
40.38367	12.23785	12.23786	12.23784
40.392	12.23805	12.23806	12.23804
40.40033	12.23995	12.23996	12.23994
40.40867	12.239	12.23901	12.23899
40.417	12.2383	12.23831	12.23829
40.42533	12.2462	12.24621	12.24619
40.43367	12.2462	12.24621	12.24619
40.442	12.2584	12.25841	12.25839
40.45033	12.25255	12.25256	12.25254
40.45867	12.2506	12.25061	12.25059
40.467	12.2495	12.24951	12.24949
40.47533	12.2438	12.24381	12.24379
40.48367	12.24735	12.24736	12.24734
40.492	12.24595	12.24596	12.24594
40.50033	12.24365	12.24366	12.24364
40.50867	12.24135	12.24136	12.24134
40.517	12.23405	12.23406	12.23404
40.52533	12.23315	12.23316	12.23314
40.53367	12.23055	12.23056	12.23054
40.542	12.2254	12.22541	12.22539
40.55033	12.2296	12.22961	12.22959
40.55867	12.229	12.22901	12.22899
40.567	12.2188	12.21881	12.21879
40.57533	12.22115	12.22116	12.22114
40.58367	12.21965	12.21966	12.21964
40.592	12.2172	12.21721	12.21719
40.60033	12.2199	12.21991	12.21989
40.60867	12.2262	12.22621	12.22619
40.617	12.2375	12.23751	12.23749
40.62533	12.24185	12.24186	12.24184
40.63367	12.2466	12.24661	12.24659
40.642	12.24915	12.24916	12.24914
40.65033	12.24425	12.24426	12.24424
40.65867	12.23195	12.23196	12.23194

40.667	12.22675	12.22676	12.22674
40.67533	12.2276	12.22761	12.22759
40.68367	12.22445	12.22446	12.22444
40.692	12.221	12.22101	12.22099
40.70033	12.223	12.22301	12.22299
40.70867	12.2206	12.22061	12.22059
40.717	12.21355	12.21356	12.21354
40.72533	12.21225	12.21226	12.21224
40.73367	12.20795	12.20796	12.20794
40.742	12.20525	12.20526	12.20524
40.75033	12.2002	12.20021	12.20019
40.75867	12.19725	12.19726	12.19724
40.767	12.19795	12.19796	12.19794
40.77533	12.20475	12.20476	12.20474
40.78367	12.2046	12.20461	12.20459
40.792	12.2043	12.20431	12.20429
40.80033	12.21335	12.21336	12.21334
40.80867	12.21675	12.21676	12.21674
40.817	12.2275	12.22751	12.22749
40.82533	12.22825	12.22826	12.22824
40.83367	12.23235	12.23236	12.23234
40.842	12.2217	12.22171	12.22169
40.85033	12.2153	12.21531	12.21529
40.85867	12.21765	12.21766	12.21764
40.867	12.2126	12.21261	12.21259
40.87533	12.21205	12.21206	12.21204
40.88367	12.21715	12.21716	12.21714
40.892	12.20885	12.20886	12.20884
40.90033	12.20605	12.20606	12.20604
40.90867	12.1982	12.19821	12.19819
40.917	12.19645	12.19646	12.19644
40.92533	12.19335	12.19336	12.19334
40.93367	12.187	12.18701	12.18699
40.942	12.1868	12.18681	12.18679
40.95033	12.18505	12.18506	12.18504
40.95867	12.1881	12.18811	12.18809
40.967	12.18235	12.18236	12.18234
40.97533	12.19145	12.19146	12.19144
40.98367	12.19435	12.19436	12.19434
40.992	12.19565	12.19566	12.19564
41.00033	12.20595	12.20596	12.20594
41.00867	12.21305	12.21306	12.21304
41.017	12.2094	12.20941	12.20939
41.02533	12.20765	12.20766	12.20764

41.03367	12.2038	12.20381	12.20379
41.042	12.1961	12.19611	12.19609
41.05033	12.1966	12.19661	12.19659
41.05867	12.19305	12.19306	12.19304
41.067	12.18805	12.18806	12.18804
41.07533	12.18975	12.18976	12.18974
41.08367	12.183	12.18301	12.18299
41.092	12.1797	12.17971	12.17969
41.10033	12.1805	12.18051	12.18049
41.10867	12.17755	12.17756	12.17754
41.117	12.17435	12.17436	12.17434
41.12533	12.174	12.17401	12.17399
41.13367	12.17625	12.17626	12.17624
41.142	12.1694	12.16941	12.16939
41.15033	12.1772	12.17721	12.17719
41.15867	12.17715	12.17716	12.17714
41.167	12.1774	12.17741	12.17739
41.17533	12.18945	12.18946	12.18944
41.18367	12.19805	12.19806	12.19804
41.192	12.2024	12.20241	12.20239
41.20033	12.2011	12.20111	12.20109
41.20867	12.20695	12.20696	12.20694
41.217	12.1972	12.19721	12.19719
41.22533	12.19865	12.19866	12.19864
41.23367	12.19835	12.19836	12.19834
41.242	12.1869	12.18691	12.18689
41.25033	12.1887	12.18871	12.18869
41.25867	12.18015	12.18016	12.18014
41.267	12.1802	12.18021	12.18019
41.27533	12.17725	12.17726	12.17724
41.28367	12.17005	12.17006	12.17004
41.292	12.17075	12.17076	12.17074
41.30033	12.17785	12.17786	12.17784
41.30867	12.167	12.16701	12.16699
41.317	12.1648	12.16481	12.16479
41.32533	12.161	12.16101	12.16099
41.33367	12.1611	12.16111	12.16109
41.342	12.1681	12.16811	12.16809
41.35033	12.1724	12.17241	12.17239
41.35867	12.1728	12.17281	12.17279
41.367	12.1885	12.18851	12.18849
41.37533	12.18935	12.18936	12.18934
41.38367	12.19535	12.19536	12.19534
41.392	12.192	12.19201	12.19199

41.40033	12.18645	12.18646	12.18644
41.40867	12.18555	12.18556	12.18554
41.417	12.19285	12.19286	12.19284
41.42533	12.18825	12.18826	12.18824
41.43367	12.18565	12.18566	12.18564
41.442	12.1745	12.17451	12.17449
41.45033	12.17785	12.17786	12.17784
41.45867	12.176	12.17601	12.17599
41.467	12.16905	12.16906	12.16904
41.47533	12.1679	12.16791	12.16789
41.48367	12.1641	12.16411	12.16409
41.492	12.15885	12.15886	12.15884
41.50033	12.15625	12.15626	12.15624
41.50867	12.1575	12.15751	12.15749
41.517	12.1595	12.15951	12.15949
41.52533	12.1612	12.16121	12.16119
41.53367	12.16805	12.16806	12.16804
41.542	12.16215	12.16216	12.16214
41.55033	12.1768	12.17681	12.17679
41.55867	12.1773	12.17731	12.17729
41.567	12.1822	12.18221	12.18219
41.57533	12.1909	12.19091	12.19089
41.58367	12.18625	12.18626	12.18624
41.592	12.1813	12.18131	12.18129
41.60033	12.17505	12.17506	12.17504
41.60867	12.17835	12.17836	12.17834
41.617	12.17475	12.17476	12.17474
41.62533	12.1732	12.17321	12.17319
41.63367	12.17595	12.17596	12.17594
41.642	12.1711	12.17111	12.17109
41.65033	12.172	12.17201	12.17199
41.65867	12.1603	12.16031	12.16029
41.667	12.1587	12.15871	12.15869
41.67533	12.16295	12.16296	12.16294
41.68367	12.1548	12.15481	12.15479
41.692	12.15495	12.15496	12.15494
41.70033	12.15815	12.15816	12.15814
41.70867	12.14765	12.14766	12.14764
41.717	12.16195	12.16196	12.16194
41.72533	12.16255	12.16256	12.16254
41.73367	12.17565	12.17566	12.17564
41.742	12.18735	12.18736	12.18734
41.75033	12.1827	12.18271	12.18269
41.75867	12.19265	12.19266	12.19264

41.767	12.1948	12.19481	12.19479
41.77533	12.18875	12.18876	12.18874
41.78367	12.1847	12.18471	12.18469
41.792	12.1855	12.18551	12.18549
41.80033	12.1859	12.18591	12.18589
41.80867	12.1878	12.18781	12.18779
41.817	12.1853	12.18531	12.18529
41.82533	12.18975	12.18976	12.18974
41.83367	12.1939	12.19391	12.19389
41.842	12.1922	12.19221	12.19219
41.85033	12.188	12.18801	12.18799
41.85867	12.182	12.18201	12.18199
41.867	12.18485	12.18486	12.18484
41.87533	12.1833	12.18331	12.18329
41.88367	12.1896	12.18961	12.18959
41.892	12.1841	12.18411	12.18409
41.90033	12.19055	12.19056	12.19054
41.90867	12.1914	12.19141	12.19139
41.917	12.20745	12.20746	12.20744
41.92533	12.21675	12.21676	12.21674
41.93367	12.2236	12.22361	12.22359
41.942	12.2395	12.23951	12.23949
41.95033	12.24485	12.24486	12.24484
41.95867	12.2468	12.24681	12.24679
41.967	12.2466	12.24661	12.24659
41.97533	12.2429	12.24291	12.24289
41.98367	12.2503	12.25031	12.25029
41.992	12.25645	12.25646	12.25644
42.00033	12.24925	12.24926	12.24924
42.00867	12.2522	12.25221	12.25219
42.017	12.2571	12.25711	12.25709
42.02533	12.2575	12.25751	12.25749
42.03367	12.261	12.26101	12.26099
42.042	12.258	12.25801	12.25799
42.05033	12.2599	12.25991	12.25989
42.05867	12.2627	12.26271	12.26269
42.067	12.26495	12.26496	12.26494
42.07533	12.27155	12.27156	12.27154
42.08367	12.27405	12.27406	12.27404
42.092	12.28025	12.28026	12.28024
42.10033	12.2921	12.29211	12.29209
42.10867	12.30605	12.30606	12.30604
42.117	12.32315	12.32316	12.32314
42.12533	12.3305	12.33051	12.33049

42.13367	12.3488	12.34881	12.34879
42.142	12.34295	12.34296	12.34294
42.15033	12.34635	12.34636	12.34634
42.15867	12.34965	12.34966	12.34964
42.167	12.35125	12.35126	12.35124
42.17533	12.3512	12.35121	12.35119
42.18367	12.3536	12.35361	12.35359
42.192	12.3589	12.35891	12.35889
42.20033	12.36305	12.36306	12.36304
42.20867	12.36535	12.36536	12.36534
42.217	12.36635	12.36636	12.36634
42.22533	12.36955	12.36956	12.36954
42.23367	12.3611	12.36111	12.36109
42.242	12.36765	12.36766	12.36764
42.25033	12.37415	12.37416	12.37414
42.25867	12.379	12.37901	12.37899
42.267	12.37415	12.37416	12.37414
42.27533	12.3767	12.37671	12.37669
42.28367	12.38665	12.38666	12.38664
42.292	12.3905	12.39051	12.39049
42.30033	12.40355	12.40356	12.40354
42.30867	12.41035	12.41036	12.41034
42.317	12.41625	12.41626	12.41624
42.32533	12.41965	12.41966	12.41964
42.33367	12.42065	12.42066	12.42064
42.342	12.4255	12.42551	12.42549
42.35033	12.41755	12.41756	12.41754
42.35867	12.41955	12.41956	12.41954
42.367	12.4158	12.41581	12.41579
42.37533	12.41345	12.41346	12.41344
42.38367	12.4176	12.41761	12.41759
42.392	12.41925	12.41926	12.41924
42.40033	12.4108	12.41081	12.41079
42.40867	12.41005	12.41006	12.41004
42.417	12.41105	12.41106	12.41104
42.42533	12.40595	12.40596	12.40594
42.43367	12.40475	12.40476	12.40474
42.442	12.4126	12.41261	12.41259
42.45033	12.4103	12.41031	12.41029
42.45867	12.4168	12.41681	12.41679
42.467	12.41985	12.41986	12.41984
42.47533	12.4289	12.42891	12.42889
42.48367	12.4479	12.44791	12.44789
42.492	12.45515	12.45516	12.45514

42.50033	12.47355	12.47356	12.47354
42.50867	12.48685	12.48686	12.48684
42.517	12.4971	12.49711	12.49709
42.52533	12.4972	12.49721	12.49719
42.53367	12.49755	12.49756	12.49754
42.542	12.50375	12.50376	12.50374
42.55033	12.5088	12.50881	12.50879
42.55867	12.5198	12.51981	12.51979
42.567	12.529	12.52901	12.52899
42.57533	12.53385	12.53386	12.53384
42.58367	12.5422	12.54221	12.54219
42.592	12.5524	12.55241	12.55239
42.60033	12.5528	12.55281	12.55279
42.60867	12.5626	12.56261	12.56259
42.617	12.57705	12.57706	12.57704
42.62533	12.58525	12.58526	12.58524
42.63367	12.60425	12.60426	12.60424
42.642	12.6044	12.60441	12.60439
42.65033	12.6255	12.62551	12.62549
42.65867	12.63615	12.63616	12.63614
42.667	12.6547	12.65471	12.65469
42.67533	12.68305	12.68306	12.68304
42.68367	12.7086	12.70861	12.70859
42.692	12.72465	12.72466	12.72464
42.70033	12.7444	12.74441	12.74439
42.70867	12.7523	12.75231	12.75229
42.717	12.7623	12.76231	12.76229
42.72533	12.77355	12.77356	12.77354
42.73367	12.78615	12.78616	12.78614
42.742	12.79805	12.79806	12.79804
42.75033	12.8121	12.81211	12.81209
42.75867	12.82645	12.82646	12.82644
42.767	12.8349	12.83491	12.83489
42.77533	12.83675	12.83676	12.83674
42.78367	12.84625	12.84626	12.84624
42.792	12.8543	12.85431	12.85429
42.80033	12.8614	12.86141	12.86139
42.80867	12.87625	12.87626	12.87624
42.817	12.88645	12.88646	12.88644
42.82533	12.89215	12.89216	12.89214
42.83367	12.90175	12.90176	12.90174
42.842	12.92155	12.92156	12.92154
42.85033	12.93505	12.93506	12.93504
42.85867	12.95105	12.95106	12.95104

42.867	12.96005	12.96006	12.96004
42.87533	12.97835	12.97836	12.97834
42.88367	13.002	13.00201	13.00199
42.892	13.0011	13.00111	13.00109
42.90033	13.00325	13.00326	13.00324
42.90867	13.0034	13.00341	13.00339
42.917	13.00745	13.00746	13.00744
42.92533	13.0066	13.00661	13.00659
42.93367	13.0063	13.00631	13.00629
42.942	13.0087	13.00871	13.00869
42.95033	13.002	13.00201	13.00199
42.95867	12.9953	12.99531	12.99529
42.967	12.9972	12.99721	12.99719
42.97533	12.98055	12.98056	12.98054
42.98367	12.98025	12.98026	12.98024
42.992	12.96565	12.96566	12.96564
43.00033	12.9656	12.96561	12.96559
43.00867	12.95995	12.95996	12.95994
43.017	12.96145	12.96146	12.96144
43.02533	12.9571	12.95711	12.95709
43.03367	12.95415	12.95416	12.95414
43.042	12.94925	12.94926	12.94924
43.05033	12.9538	12.95381	12.95379
43.05867	12.9498	12.94981	12.94979
43.067	12.9563	12.95631	12.95629
43.07533	12.9382	12.93821	12.93819
43.08367	12.92555	12.92556	12.92554
43.092	12.9155	12.91551	12.91549
43.10033	12.89905	12.89906	12.89904
43.10867	12.8821	12.88211	12.88209
43.117	12.8663	12.86631	12.86629
43.12533	12.8547	12.85471	12.85469
43.13367	12.8396	12.83961	12.83959
43.142	12.8235	12.82351	12.82349
43.15033	12.8146	12.81461	12.81459
43.15867	12.80025	12.80026	12.80024
43.167	12.79235	12.79236	12.79234
43.17533	12.78015	12.78016	12.78014
43.18367	12.76705	12.76706	12.76704
43.192	12.7533	12.75331	12.75329
43.20033	12.7545	12.75451	12.75449
43.20867	12.7427	12.74271	12.74269
43.217	12.74495	12.74496	12.74494
43.22533	12.7461	12.74611	12.74609

43.23367	12.74835	12.74836	12.74834
43.242	12.752	12.75201	12.75199
43.25033	12.7612	12.76121	12.76119
43.25867	12.76025	12.76026	12.76024
43.267	12.76455	12.76456	12.76454
43.27533	12.75435	12.75436	12.75434
43.28367	12.75595	12.75596	12.75594
43.292	12.75	12.75001	12.74999
43.30033	12.75515	12.75516	12.75514
43.30867	12.7552	12.75521	12.75519
43.317	12.7544	12.75441	12.75439
43.32533	12.7524	12.75241	12.75239
43.33367	12.74865	12.74866	12.74864
43.342	12.7507	12.75071	12.75069
43.35033	12.75335	12.75336	12.75334
43.35867	12.75635	12.75636	12.75634
43.367	12.75565	12.75566	12.75564
43.37533	12.76405	12.76406	12.76404
43.38367	12.77185	12.77186	12.77184
43.392	12.77785	12.77786	12.77784
43.40033	12.794	12.79401	12.79399
43.40867	12.80595	12.80596	12.80594
43.417	12.821	12.82101	12.82099
43.42533	12.8415	12.84151	12.84149
43.43367	12.8535	12.85351	12.85349
43.442	12.8732	12.87321	12.87319
43.45033	12.88055	12.88056	12.88054
43.45867	12.8914	12.89141	12.89139
43.467	12.8899	12.88991	12.88989
43.47533	12.89845	12.89846	12.89844
43.48367	12.8999	12.89991	12.89989
43.492	12.90185	12.90186	12.90184
43.50033	12.9118	12.91181	12.91179
43.50867	12.91775	12.91776	12.91774
43.517	12.9239	12.92391	12.92389
43.52533	12.93455	12.93456	12.93454
43.53367	12.93735	12.93736	12.93734
43.542	12.93215	12.93216	12.93214
43.55033	12.9414	12.94141	12.94139
43.55867	12.9506	12.95061	12.95059
43.567	12.9591	12.95911	12.95909
43.57533	12.9642	12.96421	12.96419
43.58367	12.9722	12.97221	12.97219
43.592	12.9984	12.99841	12.99839

43.60033	13.00505	13.00506	13.00504
43.60867	13.01955	13.01956	13.01954
43.617	13.04125	13.04126	13.04124
43.62533	13.04865	13.04866	13.04864
43.63367	13.0675	13.06751	13.06749
43.642	13.08035	13.08036	13.08034
43.65033	13.08015	13.08016	13.08014
43.65867	13.0948	13.09481	13.09479
43.667	13.09985	13.09986	13.09984
43.67533	13.10355	13.10356	13.10354
43.68367	13.105	13.10501	13.10499
43.692	13.1168	13.11681	13.11679
43.70033	13.11785	13.11786	13.11784
43.70867	13.126	13.12601	13.12599
43.717	13.1261	13.12611	13.12609
43.72533	13.1341	13.13411	13.13409
43.73367	13.13875	13.13876	13.13874
43.742	13.14095	13.14096	13.14094
43.75033	13.14855	13.14856	13.14854
43.75867	13.1529	13.15291	13.15289
43.767	13.1658	13.16581	13.16579
43.77533	13.16855	13.16856	13.16854
43.78367	13.18305	13.18306	13.18304
43.792	13.2043	13.20431	13.20429
43.80033	13.2229	13.22291	13.22289
43.80867	13.22635	13.22636	13.22634
43.817	13.24575	13.24576	13.24574
43.82533	13.2587	13.25871	13.25869
43.83367	13.26275	13.26276	13.26274
43.842	13.26865	13.26866	13.26864
43.85033	13.26805	13.26806	13.26804
43.85867	13.2742	13.27421	13.27419
43.867	13.2782	13.27821	13.27819
43.87533	13.28175	13.28176	13.28174
43.88367	13.2852	13.28521	13.28519
43.892	13.29875	13.29876	13.29874
43.90033	13.30065	13.30066	13.30064
43.90867	13.2978	13.29781	13.29779
43.917	13.3074	13.30741	13.30739
43.92533	13.3106	13.31061	13.31059
43.93367	13.3145	13.31451	13.31449
43.942	13.3126	13.31261	13.31259
43.95033	13.31545	13.31546	13.31544
43.95867	13.33175	13.33176	13.33174

43.967	13.3393	13.33931	13.33929
43.97533	13.33875	13.33876	13.33874
43.98367	13.3643	13.36431	13.36429
43.992	13.3781	13.37811	13.37809
44.00033	13.399	13.39901	13.39899
44.00867	13.4148	13.41481	13.41479
44.017	13.41925	13.41926	13.41924
44.02533	13.42415	13.42416	13.42414
44.03367	13.42925	13.42926	13.42924
44.042	13.43105	13.43106	13.43104
44.05033	13.43235	13.43236	13.43234
44.05867	13.4263	13.42631	13.42629
44.067	13.43695	13.43696	13.43694
44.07533	13.44035	13.44036	13.44034
44.08367	13.4505	13.45051	13.45049
44.092	13.45095	13.45096	13.45094
44.10033	13.44295	13.44296	13.44294
44.10867	13.44665	13.44666	13.44664
44.117	13.45425	13.45426	13.45424
44.12533	13.46215	13.46216	13.46214
44.13367	13.46455	13.46456	13.46454
44.142	13.47075	13.47076	13.47074
44.15033	13.4839	13.48391	13.48389
44.15867	13.4977	13.49771	13.49769
44.167	13.5242	13.52421	13.52419
44.17533	13.5308	13.53081	13.53079
44.18367	13.54485	13.54486	13.54484
44.192	13.5639	13.56391	13.56389
44.20033	13.5675	13.56751	13.56749
44.20867	13.56945	13.56946	13.56944
44.217	13.5741	13.57411	13.57409
44.22533	13.5816	13.58161	13.58159
44.23367	13.58085	13.58086	13.58084
44.242	13.57895	13.57896	13.57894
44.25033	13.5948	13.59481	13.59479
44.25867	13.59325	13.59326	13.59324
44.267	13.5906	13.59061	13.59059
44.27533	13.59785	13.59786	13.59784
44.28367	13.6005	13.60051	13.60049
44.292	13.60075	13.60076	13.60074
44.30033	13.6013	13.60131	13.60129
44.30867	13.6062	13.60621	13.60619
44.317	13.611	13.61101	13.61099
44.32533	13.61195	13.61196	13.61194

44.33367	13.629	13.62901	13.62899
44.342	13.64385	13.64386	13.64384
44.35033	13.64825	13.64826	13.64824
44.35867	13.6753	13.67531	13.67529
44.367	13.6811	13.68111	13.68109
44.37533	13.70685	13.70686	13.70684
44.38367	13.72345	13.72346	13.72344
44.392	13.7199	13.71991	13.71989
44.40033	13.7283	13.72831	13.72829
44.40867	13.73195	13.73196	13.73194
44.417	13.7313	13.73131	13.73129
44.42533	13.7391	13.73911	13.73909
44.43367	13.7309	13.73091	13.73089
44.442	13.73865	13.73866	13.73864
44.45033	13.7571	13.75711	13.75709
44.45867	13.7519	13.75191	13.75189
44.467	13.74595	13.74596	13.74594
44.47533	13.7564	13.75641	13.75639
44.48367	13.7658	13.76581	13.76579
44.492	13.75655	13.75656	13.75654
44.50033	13.76625	13.76626	13.76624
44.50867	13.77385	13.77386	13.77384
44.517	13.78775	13.78776	13.78774
44.52533	13.7921	13.79211	13.79209
44.53367	13.8036	13.80361	13.80359
44.542	13.81245	13.81246	13.81244
44.55033	13.8375	13.83751	13.83749
44.55867	13.84635	13.84636	13.84634
44.567	13.86975	13.86976	13.86974
44.57533	13.8775	13.87751	13.87749
44.58367	13.87795	13.87796	13.87794
44.592	13.88135	13.88136	13.88134
44.60033	13.88335	13.88336	13.88334
44.60867	13.88715	13.88716	13.88714
44.617	13.89585	13.89586	13.89584
44.62533	13.894	13.89401	13.89399
44.63367	13.90205	13.90206	13.90204
44.642	13.90275	13.90276	13.90274
44.65033	13.9048	13.90481	13.90479
44.65867	13.906	13.90601	13.90599
44.667	13.90525	13.90526	13.90524
44.67533	13.9062	13.90621	13.90619
44.68367	13.9098	13.90981	13.90979
44.692	13.9186	13.91861	13.91859

44.70033	13.92505	13.92506	13.92504
44.70867	13.93925	13.93926	13.93924
44.717	13.9434	13.94341	13.94339
44.72533	13.96455	13.96456	13.96454
44.73367	13.98515	13.98516	13.98514
44.742	13.9996	13.99961	13.99959
44.75033	14.0199	14.01991	14.01989
44.75867	14.0271	14.02711	14.02709
44.767	14.0336	14.03361	14.03359
44.77533	14.032	14.03201	14.03199
44.78367	14.03665	14.03666	14.03664
44.792	14.04235	14.04236	14.04234
44.80033	14.0449	14.04491	14.04489
44.80867	14.04055	14.04056	14.04054
44.817	14.0413	14.04131	14.04129
44.82533	14.0517	14.05171	14.05169
44.83367	14.05805	14.05806	14.05804
44.842	14.0568	14.05681	14.05679
44.85033	14.0494	14.04941	14.04939
44.85867	14.0606	14.06061	14.06059
44.867	14.05175	14.05176	14.05174
44.87533	14.0601	14.06011	14.06009
44.88367	14.05725	14.05726	14.05724
44.892	14.07665	14.07666	14.07664
44.90033	14.08645	14.08646	14.08644
44.90867	14.11165	14.11166	14.11164
44.917	14.12005	14.12006	14.12004
44.92533	14.14265	14.14266	14.14264
44.93367	14.1651	14.16511	14.16509
44.942	14.1842	14.18421	14.18419
44.95033	14.1865	14.18651	14.18649
44.95867	14.2015	14.20151	14.20149
44.967	14.1997	14.19971	14.19969
44.97533	14.20525	14.20526	14.20524
44.98367	14.21665	14.21666	14.21664
44.992	14.20865	14.20866	14.20864
45.00033	14.2129	14.21291	14.21289
45.00867	14.2236	14.22361	14.22359
45.017	14.21945	14.21946	14.21944
45.02533	14.2261	14.22611	14.22609
45.03367	14.23295	14.23296	14.23294
45.042	14.2231	14.22311	14.22309
45.05033	14.23795	14.23796	14.23794
45.05867	14.24215	14.24216	14.24214

45.067	14.2393	14.23931	14.23929
45.07533	14.26275	14.26276	14.26274
45.08367	14.25775	14.25776	14.25774
45.092	14.27415	14.27416	14.27414
45.10033	14.29245	14.29246	14.29244
45.10867	14.3109	14.31091	14.31089
45.117	14.3278	14.32781	14.32779
45.12533	14.3464	14.34641	14.34639
45.13367	14.364	14.36401	14.36399
45.142	14.3642	14.36421	14.36419
45.15033	14.37	14.37001	14.36999
45.15867	14.37955	14.37956	14.37954
45.167	14.38015	14.38016	14.38014
45.17533	14.3732	14.37321	14.37319
45.18367	14.3784	14.37841	14.37839
45.192	14.3917	14.39171	14.39169
45.20033	14.39545	14.39546	14.39544
45.20867	14.4032	14.40321	14.40319
45.217	14.39935	14.39936	14.39934
45.22533	14.41005	14.41006	14.41004
45.23367	14.39925	14.39926	14.39924
45.242	14.4159	14.41591	14.41589
45.25033	14.41525	14.41526	14.41524
45.25867	14.4214	14.42141	14.42139
45.267	14.43545	14.43546	14.43544
45.27533	14.448	14.44801	14.44799
45.28367	14.463	14.46301	14.46299
45.292	14.48725	14.48726	14.48724
45.30033	14.5035	14.50351	14.50349
45.30867	14.52825	14.52826	14.52824
45.317	14.5438	14.54381	14.54379
45.32533	14.5582	14.55821	14.55819
45.33367	14.5543	14.55431	14.55429
45.342	14.55685	14.55686	14.55684
45.35033	14.56095	14.56096	14.56094
45.35867	14.5691	14.56911	14.56909
45.367	14.57445	14.57446	14.57444
45.37533	14.5798	14.57981	14.57979
45.38367	14.58505	14.58506	14.58504
45.392	14.5881	14.58811	14.58809
45.40033	14.5902	14.59021	14.59019
45.40867	14.59465	14.59466	14.59464
45.417	14.58955	14.58956	14.58954
45.42533	14.59255	14.59256	14.59254

45.43367	14.59205	14.59206	14.59204
45.442	14.5998	14.59981	14.59979
45.45033	14.60875	14.60876	14.60874
45.45867	14.6227	14.62271	14.62269
45.467	14.6354	14.63541	14.63539
45.47533	14.6532	14.65321	14.65319
45.48367	14.67575	14.67576	14.67574
45.492	14.69915	14.69916	14.69914
45.50033	14.72355	14.72356	14.72354
45.50867	14.7428	14.74281	14.74279
45.517	14.73835	14.73836	14.73834
45.52533	14.75355	14.75356	14.75354
45.53367	14.7492	14.74921	14.74919
45.542	14.7549	14.75491	14.75489
45.55033	14.7601	14.76011	14.76009
45.55867	14.7623	14.76231	14.76229
45.567	14.776	14.77601	14.77599
45.57533	14.77275	14.77276	14.77274
45.58367	14.7822	14.78221	14.78219
45.592	14.7855	14.78551	14.78549
45.60033	14.78775	14.78776	14.78774
45.60867	14.7851	14.78511	14.78509
45.617	14.78275	14.78276	14.78274
45.62533	14.802	14.80201	14.80199
45.63367	14.80045	14.80046	14.80044
45.642	14.80585	14.80586	14.80584
45.65033	14.82295	14.82296	14.82294
45.65867	14.8323	14.83231	14.83229
45.667	14.8602	14.86021	14.86019
45.67533	14.88655	14.88656	14.88654
45.68367	14.91035	14.91036	14.91034
45.692	14.93135	14.93136	14.93134
45.70033	14.92585	14.92586	14.92584
45.70867	14.93305	14.93306	14.93304
45.717	14.93515	14.93516	14.93514
45.72533	14.94025	14.94026	14.94024
45.73367	14.94615	14.94616	14.94614
45.742	14.94425	14.94426	14.94424
45.75033	14.9502	14.95021	14.95019
45.75867	14.94645	14.94646	14.94644
45.767	14.9565	14.95651	14.95649
45.77533	14.96005	14.96006	14.96004
45.78367	14.962	14.96201	14.96199
45.792	14.9612	14.96121	14.96119

45.80033	14.96805	14.96806	14.96804
45.80867	14.97665	14.97666	14.97664
45.817	14.97785	14.97786	14.97784
45.82533	14.9845	14.98451	14.98449
45.83367	14.99695	14.99696	14.99694
45.842	15.02405	15.02406	15.02404
45.85033	15.0331	15.03311	15.03309
45.85867	15.0704	15.07041	15.07039
45.867	15.0852	15.08521	15.08519
45.87533	15.1134	15.11341	15.11339
45.88367	15.12225	15.12226	15.12224
45.892	15.12485	15.12486	15.12484
45.90033	15.1263	15.12631	15.12629
45.90867	15.12635	15.12636	15.12634
45.917	15.1417	15.14171	15.14169
45.92533	15.1465	15.14651	15.14649
45.93367	15.14655	15.14656	15.14654
45.942	15.15505	15.15506	15.15504
45.95033	15.15285	15.15286	15.15284
45.95867	15.15155	15.15156	15.15154
45.967	15.17415	15.17416	15.17414
45.97533	15.1624	15.16241	15.16239
45.98367	15.16815	15.16816	15.16814
45.992	15.1745	15.17451	15.17449
46.00033	15.17145	15.17146	15.17144
46.00867	15.1807	15.18071	15.18069
46.017	15.1921	15.19211	15.19209
46.02533	15.212	15.21201	15.21199
46.03367	15.22815	15.22816	15.22814
46.042	15.24945	15.24946	15.24944
46.05033	15.2689	15.26891	15.26889
46.05867	15.2941	15.29411	15.29409
46.067	15.3158	15.31581	15.31579
46.07533	15.3268	15.32681	15.32679
46.08367	15.32425	15.32426	15.32424
46.092	15.32655	15.32656	15.32654
46.10033	15.33395	15.33396	15.33394
46.10867	15.33775	15.33776	15.33774
46.117	15.3372	15.33721	15.33719
46.12533	15.35155	15.35156	15.35154
46.13367	15.3456	15.34561	15.34559
46.142	15.3513	15.35131	15.35129
46.15033	15.34755	15.34756	15.34754
46.15867	15.3486	15.34861	15.34859

46.167	15.36325	15.36326	15.36324
46.17533	15.36185	15.36186	15.36184
46.18367	15.36685	15.36686	15.36684
46.192	15.3726	15.37261	15.37259
46.20033	15.37605	15.37606	15.37604
46.20867	15.39885	15.39886	15.39884
46.217	15.4137	15.41371	15.41369
46.22533	15.43365	15.43366	15.43364
46.23367	15.4504	15.45041	15.45039
46.242	15.4796	15.47961	15.47959
46.25033	15.5117	15.51171	15.51169
46.25867	15.5182	15.51821	15.51819
46.267	15.5177	15.51771	15.51769
46.27533	15.52275	15.52276	15.52274
46.28367	15.52535	15.52536	15.52534
46.292	15.53315	15.53316	15.53314
46.30033	15.53745	15.53746	15.53744
46.30867	15.53025	15.53026	15.53024
46.317	15.54535	15.54536	15.54534
46.32533	15.55055	15.55056	15.55054
46.33367	15.5517	15.55171	15.55169
46.342	15.54735	15.54736	15.54734
46.35033	15.5524	15.55241	15.55239
46.35867	15.55535	15.55536	15.55534
46.367	15.55795	15.55796	15.55794
46.37533	15.57305	15.57306	15.57304
46.38367	15.5885	15.58851	15.58849
46.392	15.5925	15.59251	15.59249
46.40033	15.60385	15.60386	15.60384
46.40867	15.6216	15.62161	15.62159
46.417	15.64275	15.64276	15.64274
46.42533	15.6782	15.67821	15.67819
46.43367	15.69215	15.69216	15.69214
46.442	15.71475	15.71476	15.71474
46.45033	15.71455	15.71456	15.71454
46.45867	15.7301	15.73011	15.73009
46.467	15.7318	15.73181	15.73179
46.47533	15.73545	15.73546	15.73544
46.48367	15.73995	15.73996	15.73994
46.492	15.73995	15.73996	15.73994
46.50033	15.75195	15.75196	15.75194
46.50867	15.753	15.75301	15.75299
46.517	15.7553	15.75531	15.75529
46.52533	15.76535	15.76536	15.76534

46.53367	15.7554	15.75541	15.75539
46.542	15.7704	15.77041	15.77039
46.55033	15.76805	15.76806	15.76804
46.55867	15.78185	15.78186	15.78184
46.567	15.7771	15.77711	15.77709
46.57533	15.7976	15.79761	15.79759
46.58367	15.80035	15.80036	15.80034
46.592	15.8125	15.81251	15.81249
46.60033	15.83615	15.83616	15.83614
46.60867	15.86725	15.86726	15.86724
46.617	15.8903	15.89031	15.89029
46.62533	15.9152	15.91521	15.91519
46.63367	15.92975	15.92976	15.92974
46.642	15.9228	15.92281	15.92279
46.65033	15.9303	15.93031	15.93029
46.65867	15.9389	15.93891	15.93889
46.667	15.9422	15.94221	15.94219
46.67533	15.9425	15.94251	15.94249
46.68367	15.95015	15.95016	15.95014
46.692	15.95845	15.95846	15.95844
46.70033	15.95585	15.95586	15.95584
46.70867	15.9649	15.96491	15.96489
46.717	15.9697	15.96971	15.96969
46.72533	15.96705	15.96706	15.96704
46.73367	15.9716	15.97161	15.97159
46.742	15.9739	15.97391	15.97389
46.75033	15.9834	15.98341	15.98339
46.75867	15.9878	15.98781	15.98779
46.767	15.99985	15.99986	15.99984
46.77533	16.01765	16.01766	16.01764
46.78367	16.03285	16.03286	16.03284
46.792	16.061	16.06101	16.06099
46.80033	16.0962	16.09621	16.09619
46.80867	16.10275	16.10276	16.10274
46.817	16.13155	16.13156	16.13154
46.82533	16.13115	16.13116	16.13114
46.83367	16.13905	16.13906	16.13904
46.842	16.14075	16.14076	16.14074
46.85033	16.1523	16.15231	16.15229
46.85867	16.15435	16.15436	16.15434
46.867	16.1565	16.15651	16.15649
46.87533	16.16175	16.16176	16.16174
46.88367	16.16555	16.16556	16.16554
46.892	16.1727	16.17271	16.17269

46.90033	16.16725	16.16726	16.16724
46.90867	16.1698	16.16981	16.16979
46.917	16.17245	16.17246	16.17244
46.92533	16.1815	16.18151	16.18149
46.93367	16.17595	16.17596	16.17594
46.942	16.18325	16.18326	16.18324
46.95033	16.20185	16.20186	16.20184
46.95867	16.21595	16.21596	16.21594
46.967	16.2303	16.23031	16.23029
46.97533	16.25605	16.25606	16.25604
46.98367	16.27835	16.27836	16.27834
46.992	16.3046	16.30461	16.30459
47.00033	16.33495	16.33496	16.33494
47.00867	16.3398	16.33981	16.33979
47.017	16.3393	16.33931	16.33929
47.02533	16.3471	16.34711	16.34709
47.03367	16.34385	16.34386	16.34384
47.042	16.3452	16.34521	16.34519
47.05033	16.35475	16.35476	16.35474
47.05867	16.35725	16.35726	16.35724
47.067	16.35675	16.35676	16.35674
47.07533	16.3691	16.36911	16.36909
47.08367	16.37145	16.37146	16.37144
47.092	16.37385	16.37386	16.37384
47.10033	16.3703	16.37031	16.37029
47.10867	16.379	16.37901	16.37899
47.117	16.38375	16.38376	16.38374
47.12533	16.39235	16.39236	16.39234
47.13367	16.39855	16.39856	16.39854
47.142	16.41085	16.41086	16.41084
47.15033	16.4297	16.42971	16.42969
47.15867	16.4457	16.44571	16.44569
47.167	16.4729	16.47291	16.47289
47.17533	16.50165	16.50166	16.50164
47.18367	16.52275	16.52276	16.52274
47.192	16.5378	16.53781	16.53779
47.20033	16.53785	16.53786	16.53784
47.20867	16.55255	16.55256	16.55254
47.217	16.5538	16.55381	16.55379
47.22533	16.5507	16.55071	16.55069
47.23367	16.5565	16.55651	16.55649
47.242	16.5622	16.56221	16.56219
47.25033	16.56825	16.56826	16.56824
47.25867	16.57315	16.57316	16.57314

47.267	16.56215	16.56216	16.56214
47.27533	16.57505	16.57506	16.57504
47.28367	16.57915	16.57916	16.57914
47.292	16.5749	16.57491	16.57489
47.30033	16.57945	16.57946	16.57944
47.30867	16.58565	16.58566	16.58564
47.317	16.59265	16.59266	16.59264
47.32533	16.61435	16.61436	16.61434
47.33367	16.6224	16.62241	16.62239
47.342	16.6364	16.63641	16.63639
47.35033	16.6554	16.65541	16.65539
47.35867	16.68875	16.68876	16.68874
47.367	16.7193	16.71931	16.71929
47.37533	16.7449	16.74491	16.74489
47.38367	16.7492	16.74921	16.74919
47.392	16.75335	16.75336	16.75334
47.40033	16.7541	16.75411	16.75409
47.40867	16.75375	16.75376	16.75374
47.417	16.7596	16.75961	16.75959
47.42533	16.7668	16.76681	16.76679
47.43367	16.7733	16.77331	16.77329
47.442	16.7757	16.77571	16.77569
47.45033	16.77085	16.77086	16.77084
47.45867	16.7708	16.77081	16.77079
47.467	16.7731	16.77311	16.77309
47.47533	16.773	16.77301	16.77299
47.48367	16.7734	16.77341	16.77339
47.492	16.78	16.78001	16.77999
47.50033	16.78305	16.78306	16.78304
47.50867	16.7976	16.79761	16.79759
47.517	16.81305	16.81306	16.81304
47.52533	16.82995	16.82996	16.82994
47.53367	16.84375	16.84376	16.84374
47.542	16.8777	16.87771	16.87769
47.55033	16.9062	16.90621	16.90619
47.55867	16.9336	16.93361	16.93359
47.567	16.9504	16.95041	16.95039
47.57533	16.94955	16.94956	16.94954
47.58367	16.94905	16.94906	16.94904
47.592	16.95415	16.95416	16.95414
47.60033	16.94765	16.94766	16.94764
47.60867	16.9553	16.95531	16.95529
47.617	16.9577	16.95771	16.95769
47.62533	16.95245	16.95246	16.95244

47.63367	16.9747	16.97471	16.97469
47.642	16.9698	16.96981	16.96979
47.65033	16.9719	16.97191	16.97189
47.65867	16.97705	16.97706	16.97704
47.667	16.9791	16.97911	16.97909
47.67533	16.98325	16.98326	16.98324
47.68367	16.9933	16.99331	16.99329
47.692	16.9913	16.99131	16.99129
47.70033	17.0103	17.01031	17.01029
47.70867	17.02095	17.02096	17.02094
47.717	17.02435	17.02436	17.02434
47.72533	17.0584	17.05841	17.05839
47.73367	17.0873	17.08731	17.08729
47.742	17.1141	17.11411	17.11409
47.75033	17.135	17.13501	17.13499
47.75867	17.14395	17.14396	17.14394
47.767	17.13875	17.13876	17.13874
47.77533	17.1491	17.14911	17.14909
47.78367	17.1503	17.15031	17.15029
47.792	17.1575	17.15751	17.15749
47.80033	17.15225	17.15226	17.15224
47.80867	17.16155	17.16156	17.16154
47.817	17.1663	17.16631	17.16629
47.82533	17.1674	17.16741	17.16739
47.83367	17.17365	17.17366	17.17364
47.842	17.1625	17.16251	17.16249
47.85033	17.16925	17.16926	17.16924
47.85867	17.1698	17.16981	17.16979
47.867	17.17625	17.17626	17.17624
47.87533	17.1755	17.17551	17.17549
47.88367	17.18175	17.18176	17.18174
47.892	17.19905	17.19906	17.19904
47.90033	17.21955	17.21956	17.21954
47.90867	17.2397	17.23971	17.23969
47.917	17.2654	17.26541	17.26539
47.92533	17.2888	17.28881	17.28879
47.93367	17.31905	17.31906	17.31904
47.942	17.3287	17.32871	17.32869
47.95033	17.3421	17.34211	17.34209
47.95867	17.33635	17.33636	17.33634
47.967	17.3397	17.33971	17.33969
47.97533	17.34045	17.34046	17.34044
47.98367	17.3412	17.34121	17.34119
47.992	17.3465	17.34651	17.34649

48.00033	17.34835	17.34836	17.34834
48.00867	17.35425	17.35426	17.35424
48.017	17.35615	17.35616	17.35614
48.02533	17.3586	17.35861	17.35859
48.03367	17.35665	17.35666	17.35664
48.042	17.3568	17.35681	17.35679
48.05033	17.35845	17.35846	17.35844
48.05867	17.36335	17.36336	17.36334
48.067	17.36665	17.36666	17.36664
48.07533	17.3841	17.38411	17.38409
48.08367	17.3919	17.39191	17.39189
48.092	17.39765	17.39766	17.39764
48.10033	17.435	17.43501	17.43499
48.10867	17.4573	17.45731	17.45729
48.117	17.4915	17.49151	17.49149
48.12533	17.50995	17.50996	17.50994
48.13367	17.524	17.52401	17.52399
48.142	17.5151	17.51511	17.51509
48.15033	17.5195	17.51951	17.51949
48.15867	17.52005	17.52006	17.52004
48.167	17.52685	17.52686	17.52684
48.17533	17.5232	17.52321	17.52319
48.18367	17.52515	17.52516	17.52514
48.192	17.53225	17.53226	17.53224
48.20033	17.5307	17.53071	17.53069
48.20867	17.53405	17.53406	17.53404
48.217	17.53015	17.53016	17.53014
48.22533	17.53405	17.53406	17.53404
48.23367	17.5321	17.53211	17.53209
48.242	17.5383	17.53831	17.53829
48.25033	17.541	17.54101	17.54099
48.25867	17.54925	17.54926	17.54924
48.267	17.5632	17.56321	17.56319
48.27533	17.57845	17.57846	17.57844
48.28367	17.5982	17.59821	17.59819
48.292	17.6224	17.62241	17.62239
48.30033	17.64875	17.64876	17.64874
48.30867	17.6831	17.68311	17.68309
48.317	17.701	17.70101	17.70099
48.32533	17.70255	17.70256	17.70254
48.33367	17.70355	17.70356	17.70354
48.342	17.697	17.69701	17.69699
48.35033	17.70385	17.70386	17.70384
48.35867	17.70105	17.70106	17.70104

48.367	17.71075	17.71076	17.71074
48.37533	17.70855	17.70856	17.70854
48.38367	17.7233	17.72331	17.72329
48.392	17.712	17.71201	17.71199
48.40033	17.70665	17.70666	17.70664
48.40867	17.70485	17.70486	17.70484
48.417	17.70295	17.70296	17.70294
48.42533	17.7048	17.70481	17.70479
48.43367	17.7116	17.71161	17.71159
48.442	17.71615	17.71616	17.71614
48.45033	17.7233	17.72331	17.72329
48.45867	17.7405	17.74051	17.74049
48.467	17.76455	17.76456	17.76454
48.47533	17.7859	17.78591	17.78589
48.48367	17.8163	17.81631	17.81629
48.492	17.83745	17.83746	17.83744
48.50033	17.85795	17.85796	17.85794
48.50867	17.86945	17.86946	17.86944
48.517	17.87165	17.87166	17.87164
48.52533	17.87015	17.87016	17.87014
48.53367	17.8644	17.86441	17.86439
48.542	17.87175	17.87176	17.87174
48.55033	17.86385	17.86386	17.86384
48.55867	17.8591	17.85911	17.85909
48.567	17.8666	17.86661	17.86659
48.57533	17.8723	17.87231	17.87229
48.58367	17.87015	17.87016	17.87014
48.592	17.8609	17.86091	17.86089
48.60033	17.8576	17.85761	17.85759
48.60867	17.85635	17.85636	17.85634
48.617	17.8604	17.86041	17.86039
48.62533	17.8688	17.86881	17.86879
48.63367	17.88265	17.88266	17.88264
48.642	17.89385	17.89386	17.89384
48.65033	17.9121	17.91211	17.91209
48.65867	17.9389	17.93891	17.93889
48.667	17.95565	17.95566	17.95564
48.67533	17.98945	17.98946	17.98944
48.68367	18.01265	18.01266	18.01264
48.692	18.0215	18.02151	18.02149
48.70033	18.02175	18.02176	18.02174
48.70867	18.02455	18.02456	18.02454
48.717	18.01905	18.01906	18.01904
48.72533	18.02295	18.02296	18.02294

48.73367	18.0211	18.02111	18.02109
48.742	18.0254	18.02541	18.02539
48.75033	18.02095	18.02096	18.02094
48.75867	18.02325	18.02326	18.02324
48.767	18.0226	18.02261	18.02259
48.77533	18.02125	18.02126	18.02124
48.78367	18.0131	18.01311	18.01309
48.792	18.0142	18.01421	18.01419
48.80033	18.01925	18.01926	18.01924
48.80867	18.01505	18.01506	18.01504
48.817	18.0235	18.02351	18.02349
48.82533	18.0364	18.03641	18.03639
48.83367	18.04485	18.04486	18.04484
48.842	18.05885	18.05886	18.05884
48.85033	18.09635	18.09636	18.09634
48.85867	18.12315	18.12316	18.12314
48.867	18.1474	18.14741	18.14739
48.87533	18.1546	18.15461	18.15459
48.88367	18.17815	18.17816	18.17814
48.892	18.17125	18.17126	18.17124
48.90033	18.1559	18.15591	18.15589
48.90867	18.15705	18.15706	18.15704
48.917	18.16615	18.16616	18.16614
48.92533	18.1642	18.16421	18.16419
48.93367	18.16485	18.16486	18.16484
48.942	18.166	18.16601	18.16599
48.95033	18.1635	18.16351	18.16349
48.95867	18.16285	18.16286	18.16284
48.967	18.17025	18.17026	18.17024
48.97533	18.14895	18.14896	18.14894
48.98367	18.1489	18.14891	18.14889
48.992	18.15395	18.15396	18.15394
49.00033	18.15575	18.15576	18.15574
49.00867	18.1641	18.16411	18.16409
49.017	18.1761	18.17611	18.17609
49.02533	18.18445	18.18446	18.18444
49.03367	18.21655	18.21656	18.21654
49.042	18.2383	18.23831	18.23829
49.05033	18.2707	18.27071	18.27069
49.05867	18.29365	18.29366	18.29364
49.067	18.3044	18.30441	18.30439
49.07533	18.3024	18.30241	18.30239
49.08367	18.30385	18.30386	18.30384
49.092	18.30505	18.30506	18.30504

49.10033	18.3016	18.30161	18.30159
49.10867	18.29725	18.29726	18.29724
49.117	18.2969	18.29691	18.29689
49.12533	18.2936	18.29361	18.29359
49.13367	18.2887	18.28871	18.28869
49.142	18.2847	18.28471	18.28469
49.15033	18.28665	18.28666	18.28664
49.15867	18.2764	18.27641	18.27639
49.167	18.2757	18.27571	18.27569
49.17533	18.28145	18.28146	18.28144
49.18367	18.28105	18.28106	18.28104
49.192	18.2956	18.29561	18.29559
49.20033	18.3076	18.30761	18.30759
49.20867	18.31195	18.31196	18.31194
49.217	18.33745	18.33746	18.33744
49.22533	18.3545	18.35451	18.35449
49.23367	18.38185	18.38186	18.38184
49.242	18.4073	18.40731	18.40729
49.25033	18.4348	18.43481	18.43479
49.25867	18.42905	18.42906	18.42904
49.267	18.4318	18.43181	18.43179
49.27533	18.4266	18.42661	18.42659
49.28367	18.4236	18.42361	18.42359
49.292	18.4261	18.42611	18.42609
49.30033	18.4168	18.41681	18.41679
49.30867	18.42125	18.42126	18.42124
49.317	18.4165	18.41651	18.41649
49.32533	18.41395	18.41396	18.41394
49.33367	18.4164	18.41641	18.41639
49.342	18.41435	18.41436	18.41434
49.35033	18.4023	18.40231	18.40229
49.35867	18.4074	18.40741	18.40739
49.367	18.41005	18.41006	18.41004
49.37533	18.4087	18.40871	18.40869
49.38367	18.41525	18.41526	18.41524
49.392	18.4288	18.42881	18.42879
49.40033	18.43955	18.43956	18.43954
49.40867	18.4588	18.45881	18.45879
49.417	18.4874	18.48741	18.48739
49.42533	18.5098	18.50981	18.50979
49.43367	18.5515	18.55151	18.55149
49.442	18.5457	18.54571	18.54569
49.45033	18.55505	18.55506	18.55504
49.45867	18.5551	18.55511	18.55509

49.467	18.54335	18.54336	18.54334
49.47533	18.53715	18.53716	18.53714
49.48367	18.54015	18.54016	18.54014
49.492	18.53395	18.53396	18.53394
49.50033	18.54115	18.54116	18.54114
49.50867	18.53275	18.53276	18.53274
49.517	18.5189	18.51891	18.51889
49.52533	18.52045	18.52046	18.52044
49.53367	18.5236	18.52361	18.52359
49.542	18.4973	18.49731	18.49729
49.55033	18.5094	18.50941	18.50939
49.55867	18.5115	18.51151	18.51149
49.567	18.5121	18.51211	18.51209
49.57533	18.5404	18.54041	18.54039
49.58367	18.5472	18.54721	18.54719
49.592	18.56515	18.56516	18.56514
49.60033	18.582	18.58201	18.58199
49.60867	18.60635	18.60636	18.60634
49.617	18.63475	18.63476	18.63474
49.62533	18.6459	18.64591	18.64589
49.63367	18.65465	18.65466	18.65464
49.642	18.64575	18.64576	18.64574
49.65033	18.65185	18.65186	18.65184
49.65867	18.65125	18.65126	18.65124
49.667	18.64405	18.64406	18.64404
49.67533	18.64145	18.64146	18.64144
49.68367	18.62595	18.62596	18.62594
49.692	18.63205	18.63206	18.63204
49.70033	18.6233	18.62331	18.62329
49.70867	18.62675	18.62676	18.62674
49.717	18.6106	18.61061	18.61059
49.72533	18.61905	18.61906	18.61904
49.73367	18.60015	18.60016	18.60014
49.742	18.6128	18.61281	18.61279
49.75033	18.61355	18.61356	18.61354
49.75867	18.62585	18.62586	18.62584
49.767	18.63415	18.63416	18.63414
49.77533	18.6501	18.65011	18.65009
49.78367	18.6755	18.67551	18.67549
49.792	18.70735	18.70736	18.70734
49.80033	18.73385	18.73386	18.73384
49.80867	18.7489	18.74891	18.74889
49.817	18.7493	18.74931	18.74929
49.82533	18.74455	18.74456	18.74454

49.83367	18.7429	18.74291	18.74289
49.842	18.74195	18.74196	18.74194
49.85033	18.7348	18.73481	18.73479
49.85867	18.73155	18.73156	18.73154
49.867	18.7279	18.72791	18.72789
49.87533	18.7285	18.72851	18.72849
49.88367	18.7311	18.73111	18.73109
49.892	18.71285	18.71286	18.71284
49.90033	18.71415	18.71416	18.71414
49.90867	18.7072	18.70721	18.70719
49.917	18.7028	18.70281	18.70279
49.92533	18.6967	18.69671	18.69669
49.93367	18.69985	18.69986	18.69984
49.942	18.7082	18.70821	18.70819
49.95033	18.72035	18.72036	18.72034
49.95867	18.7257	18.72571	18.72569
49.967	18.73595	18.73596	18.73594
49.97533	18.7773	18.77731	18.77729
49.98367	18.7817	18.78171	18.78169
49.992	18.81675	18.81676	18.81674
50.00033	18.8295	18.82951	18.82949
50.00867	18.8382	18.83821	18.83819
50.017	18.8366	18.83661	18.83659
50.02533	18.8271	18.82711	18.82709
50.03367	18.82	18.82001	18.81999
50.042	18.8243	18.82431	18.82429
50.05033	18.81045	18.81046	18.81044
50.05867	18.8188	18.81881	18.81879
50.067	18.81015	18.81016	18.81014
50.07533	18.8072	18.80721	18.80719
50.08367	18.8104	18.81041	18.81039
50.092	18.79615	18.79616	18.79614
50.10033	18.7827	18.78271	18.78269
50.10867	18.7876	18.78761	18.78759
50.117	18.7817	18.78171	18.78169
50.12533	18.79205	18.79206	18.79204
50.13367	18.7874	18.78741	18.78739
50.142	18.81385	18.81386	18.81384
50.15033	18.8167	18.81671	18.81669
50.15867	18.8378	18.83781	18.83779
50.167	18.86475	18.86476	18.86474
50.17533	18.89605	18.89606	18.89604
50.18367	18.91985	18.91986	18.91984
50.192	18.919	18.91901	18.91899

50.20033	18.9106	18.91061	18.91059
50.20867	18.911	18.91101	18.91099
50.217	18.9129	18.91291	18.91289
50.22533	18.91175	18.91176	18.91174
50.23367	18.9014	18.90141	18.90139
50.242	18.89425	18.89426	18.89424
50.25033	18.88585	18.88586	18.88584
50.25867	18.89575	18.89576	18.89574
50.267	18.87015	18.87016	18.87014
50.27533	18.8769	18.87691	18.87689
50.28367	18.8695	18.86951	18.86949
50.292	18.85675	18.85676	18.85674
50.30033	18.85695	18.85696	18.85694
50.30867	18.85855	18.85856	18.85854
50.317	18.86755	18.86756	18.86754
50.32533	18.8822	18.88221	18.88219
50.33367	18.8822	18.88221	18.88219
50.342	18.90455	18.90456	18.90454
50.35033	18.92875	18.92876	18.92874
50.35867	18.95145	18.95146	18.95144
50.367	18.9864	18.98641	18.98639
50.37533	18.9953	18.99531	18.99529
50.38367	18.9946	18.99461	18.99459
50.392	18.9907	18.99071	18.99069
50.40033	18.98495	18.98496	18.98494
50.40867	18.97045	18.97046	18.97044
50.417	18.96685	18.96686	18.96684
50.42533	18.9719	18.97191	18.97189
50.43367	18.9731	18.97311	18.97309
50.442	18.9665	18.96651	18.96649
50.45033	18.961	18.96101	18.96099
50.45867	18.9633	18.96331	18.96329
50.467	18.945	18.94501	18.94499
50.47533	18.93655	18.93656	18.93654
50.48367	18.93555	18.93556	18.93554
50.492	18.93055	18.93056	18.93054
50.50033	18.9401	18.94011	18.94009
50.50867	18.94665	18.94666	18.94664
50.517	18.94585	18.94586	18.94584
50.52533	18.96965	18.96966	18.96964
50.53367	18.9939	18.99391	18.99389
50.542	19.02095	19.02096	19.02094
50.55033	19.04475	19.04476	19.04474
50.55867	19.06705	19.06706	19.06704

50.567	19.07025	19.07026	19.07024
50.57533	19.0614	19.06141	19.06139
50.58367	19.05195	19.05196	19.05194
50.592	19.04245	19.04246	19.04244
50.60033	19.05155	19.05156	19.05154
50.60867	19.0489	19.04891	19.04889
50.617	19.0376	19.03761	19.03759
50.62533	19.0253	19.02531	19.02529
50.63367	19.02375	19.02376	19.02374
50.642	19.02345	19.02346	19.02344
50.65033	19.02275	19.02276	19.02274
50.65867	19.00725	19.00726	19.00724
50.667	19.0016	19.00161	19.00159
50.67533	19.00435	19.00436	19.00434
50.68367	18.99335	18.99336	18.99334
50.692	18.9965	18.99651	18.99649
50.70033	19.0154	19.01541	19.01539
50.70867	19.02275	19.02276	19.02274
50.717	19.0508	19.05081	19.05079
50.72533	19.0773	19.07731	19.07729
50.73367	19.09655	19.09656	19.09654
50.742	19.1212	19.12121	19.12119
50.75033	19.13075	19.13076	19.13074
50.75867	19.13065	19.13066	19.13064
50.767	19.1175	19.11751	19.11749
50.77533	19.1065	19.10651	19.10649
50.78367	19.1121	19.11211	19.11209
50.792	19.1121	19.11211	19.11209
50.80033	19.09305	19.09306	19.09304
50.80867	19.0919	19.09191	19.09189
50.817	19.0913	19.09131	19.09129
50.82533	19.08445	19.08446	19.08444
50.83367	19.0743	19.07431	19.07429
50.842	19.0629	19.06291	19.06289
50.85033	19.06175	19.06176	19.06174
50.85867	19.05105	19.05106	19.05104
50.867	19.0602	19.06021	19.06019
50.87533	19.05865	19.05866	19.05864
50.88367	19.06425	19.06426	19.06424
50.892	19.0778	19.07781	19.07779
50.90033	19.089	19.08901	19.08899
50.90867	19.1166	19.11661	19.11659
50.917	19.134	19.13401	19.13399
50.92533	19.1758	19.17581	19.17579

50.93367	19.1897	19.18971	19.18969
50.942	19.1945	19.19451	19.19449
50.95033	19.1805	19.18051	19.18049
50.95867	19.1736	19.17361	19.17359
50.967	19.17335	19.17336	19.17334
50.97533	19.16575	19.16576	19.16574
50.98367	19.1631	19.16311	19.16309
50.992	19.1488	19.14881	19.14879
51.00033	19.15995	19.15996	19.15994
51.00867	19.1447	19.14471	19.14469
51.017	19.14445	19.14446	19.14444
51.02533	19.1304	19.13041	19.13039
51.03367	19.1285	19.12851	19.12849
51.042	19.11895	19.11896	19.11894
51.05033	19.1171	19.11711	19.11709
51.05867	19.1115	19.11151	19.11149
51.067	19.11705	19.11706	19.11704
51.07533	19.1228	19.12281	19.12279
51.08367	19.14155	19.14156	19.14154
51.092	19.1541	19.15411	19.15409
51.10033	19.18395	19.18396	19.18394
51.10867	19.19065	19.19066	19.19064
51.117	19.22585	19.22586	19.22584
51.12533	19.23955	19.23956	19.23954
51.13367	19.2318	19.23181	19.23179
51.142	19.22455	19.22456	19.22454
51.15033	19.22085	19.22086	19.22084
51.15867	19.22175	19.22176	19.22174
51.167	19.2234	19.22341	19.22339
51.17533	19.194	19.19401	19.19399
51.18367	19.19425	19.19426	19.19424
51.192	19.1991	19.19911	19.19909
51.20033	19.1828	19.18281	19.18279
51.20867	19.18925	19.18926	19.18924
51.217	19.1819	19.18191	19.18189
51.22533	19.1497	19.14971	19.14969
51.23367	19.1525	19.15251	19.15249
51.242	19.1695	19.16951	19.16949
51.25033	19.1654	19.16541	19.16539
51.25867	19.17585	19.17586	19.17584
51.267	19.17995	19.17996	19.17994
51.27533	19.1906	19.19061	19.19059
51.28367	19.2203	19.22031	19.22029
51.292	19.24435	19.24436	19.24434

51.30033	19.2698	19.26981	19.26979
51.30867	19.2888	19.28881	19.28879
51.317	19.2941	19.29411	19.29409
51.32533	19.2748	19.27481	19.27479
51.33367	19.264	19.26401	19.26399
51.342	19.2691	19.26911	19.26909
51.35033	19.26085	19.26086	19.26084
51.35867	19.2486	19.24861	19.24859
51.367	19.247	19.24701	19.24699
51.37533	19.2501	19.25011	19.25009
51.38367	19.24325	19.24326	19.24324
51.392	19.2444	19.24441	19.24439
51.40033	19.2303	19.23031	19.23029
51.40867	19.2188	19.21881	19.21879
51.417	19.21175	19.21176	19.21174
51.42533	19.2168	19.21681	19.21679
51.43367	19.20305	19.20306	19.20304
51.442	19.2054	19.20541	19.20539
51.45033	19.22205	19.22206	19.22204
51.45867	19.23335	19.23336	19.23334
51.467	19.2543	19.25431	19.25429
51.47533	19.27955	19.27956	19.27954
51.48367	19.3047	19.30471	19.30469
51.492	19.3239	19.32391	19.32389
51.50033	19.3374	19.33741	19.33739
51.50867	19.3315	19.33151	19.33149
51.517	19.3263	19.32631	19.32629
51.52533	19.3165	19.31651	19.31649
51.53367	19.314	19.31401	19.31399
51.542	19.3068	19.30681	19.30679
51.55033	19.30065	19.30066	19.30064
51.55867	19.29725	19.29726	19.29724
51.567	19.28205	19.28206	19.28204
51.57533	19.2847	19.28471	19.28469
51.58367	19.27785	19.27786	19.27784
51.592	19.2687	19.26871	19.26869
51.60033	19.2655	19.26551	19.26549
51.60867	19.26575	19.26576	19.26574
51.617	19.25375	19.25376	19.25374
51.62533	19.2526	19.25261	19.25259
51.63367	19.2641	19.26411	19.26409
51.642	19.26975	19.26976	19.26974
51.65033	19.28625	19.28626	19.28624
51.65867	19.3131	19.31311	19.31309

51.667	19.34305	19.34306	19.34304
51.67533	19.36115	19.36116	19.36114
51.68367	19.3819	19.38191	19.38189
51.692	19.3822	19.38221	19.38219
51.70033	19.3687	19.36871	19.36869
51.70867	19.3667	19.36671	19.36669
51.717	19.3632	19.36321	19.36319
51.72533	19.3511	19.35111	19.35109
51.73367	19.35285	19.35286	19.35284
51.742	19.35115	19.35116	19.35114
51.75033	19.3396	19.33961	19.33959
51.75867	19.3358	19.33581	19.33579
51.767	19.3452	19.34521	19.34519
51.77533	19.3226	19.32261	19.32259
51.78367	19.3061	19.30611	19.30609
51.792	19.30135	19.30136	19.30134
51.80033	19.3031	19.30311	19.30309
51.80867	19.29575	19.29576	19.29574
51.817	19.30065	19.30066	19.30064
51.82533	19.3103	19.31031	19.31029
51.83367	19.3256	19.32561	19.32559
51.842	19.34635	19.34636	19.34634
51.85033	19.3684	19.36841	19.36839
51.85867	19.38615	19.38616	19.38614
51.867	19.4171	19.41711	19.41709
51.87533	19.4231	19.42311	19.42309
51.88367	19.4095	19.40951	19.40949
51.892	19.4018	19.40181	19.40179
51.90033	19.39745	19.39746	19.39744
51.90867	19.39465	19.39466	19.39464
51.917	19.3987	19.39871	19.39869
51.92533	19.3852	19.38521	19.38519
51.93367	19.37385	19.37386	19.37384
51.942	19.36675	19.36676	19.36674
51.95033	19.359	19.35901	19.35899
51.95867	19.3577	19.35771	19.35769
51.967	19.34255	19.34256	19.34254
51.97533	19.34165	19.34166	19.34164
51.98367	19.3398	19.33981	19.33979
51.992	19.33965	19.33966	19.33964
52.00033	19.34355	19.34356	19.34354
52.00867	19.3405	19.34051	19.34049
52.017	19.35285	19.35286	19.35284
52.02533	19.36025	19.36026	19.36024

52.03367	19.38255	19.38256	19.38254
52.042	19.4146	19.41461	19.41459
52.05033	19.43415	19.43416	19.43414
52.05867	19.44505	19.44506	19.44504
52.067	19.45535	19.45536	19.45534
52.07533	19.4424	19.44241	19.44239
52.08367	19.43945	19.43946	19.43944
52.092	19.4389	19.43891	19.43889
52.10033	19.4316	19.43161	19.43159
52.10867	19.43005	19.43006	19.43004
52.117	19.42085	19.42086	19.42084
52.12533	19.41605	19.41606	19.41604
52.13367	19.4065	19.40651	19.40649
52.142	19.40175	19.40176	19.40174
52.15033	19.39195	19.39196	19.39194
52.15867	19.37855	19.37856	19.37854
52.167	19.3726	19.37261	19.37259
52.17533	19.37495	19.37496	19.37494
52.18367	19.3679	19.36791	19.36789
52.192	19.37915	19.37916	19.37914
52.20033	19.3711	19.37111	19.37109
52.20867	19.38385	19.38386	19.38384
52.217	19.42155	19.42156	19.42154
52.22533	19.435	19.43501	19.43499
52.23367	19.46245	19.46246	19.46244
52.242	19.4815	19.48151	19.48149
52.25033	19.48265	19.48266	19.48264
52.25867	19.47945	19.47946	19.47944
52.267	19.46395	19.46396	19.46394
52.27533	19.4591	19.45911	19.45909
52.28367	19.44775	19.44776	19.44774
52.292	19.44345	19.44346	19.44344
52.30033	19.44105	19.44106	19.44104
52.30867	19.42035	19.42036	19.42034
52.317	19.428	19.42801	19.42799
52.32533	19.40595	19.40596	19.40594
52.33367	19.39015	19.39016	19.39014
52.342	19.37345	19.37346	19.37344
52.35033	19.36685	19.36686	19.36684
52.35867	19.35865	19.35866	19.35864
52.367	19.34285	19.34286	19.34284
52.37533	19.3354	19.33541	19.33539
52.38367	19.3275	19.32751	19.32749
52.392	19.33565	19.33566	19.33564

52.40033	19.34535	19.34536	19.34534
52.40867	19.37015	19.37016	19.37014
52.417	19.375	19.37501	19.37499
52.42533	19.3865	19.38651	19.38649
52.43367	19.37995	19.37996	19.37994
52.442	19.3668	19.36681	19.36679
52.45033	19.34925	19.34926	19.34924
52.45867	19.3288	19.32881	19.32879
52.467	19.30605	19.30606	19.30604
52.47533	19.28	19.28001	19.27999
52.48367	19.2446	19.24461	19.24459
52.492	19.2237	19.22371	19.22369
52.50033	19.195	19.19501	19.19499
52.50867	19.1577	19.15771	19.15769
52.517	19.13955	19.13956	19.13954
52.52533	19.1059	19.10591	19.10589
52.53367	19.0782	19.07821	19.07819
52.542	19.05585	19.05586	19.05584
52.55033	19.03145	19.03146	19.03144
52.55867	19.003	19.00301	19.00299
52.567	18.9813	18.98131	18.98129
52.57533	18.96135	18.96136	18.96134
52.58367	18.9457	18.94571	18.94569
52.592	18.9428	18.94281	18.94279
52.60033	18.93005	18.93006	18.93004
52.60867	18.9321	18.93211	18.93209
52.617	18.9186	18.91861	18.91859
52.62533	18.89455	18.89456	18.89454
52.63367	18.8526	18.85261	18.85259
52.642	18.81625	18.81626	18.81624
52.65033	18.76855	18.76856	18.76854
52.65867	18.7408	18.74081	18.74079
52.667	18.69815	18.69816	18.69814
52.67533	18.65325	18.65326	18.65324
52.68367	18.61015	18.61016	18.61014
52.692	18.56195	18.56196	18.56194
52.70033	18.529	18.52901	18.52899
52.70867	18.48385	18.48386	18.48384
52.717	18.43555	18.43556	18.43554
52.72533	18.39335	18.39336	18.39334
52.73367	18.35275	18.35276	18.35274
52.742	18.3093	18.30931	18.30929
52.75033	18.2801	18.28011	18.28009
52.75867	18.23905	18.23906	18.23904

52.767	18.20525	18.20526	18.20524
52.77533	18.1872	18.18721	18.18719
52.78367	18.17295	18.17296	18.17294
52.792	18.15905	18.15906	18.15904
52.80033	18.1395	18.13951	18.13949
52.80867	18.10475	18.10476	18.10474
52.817	18.05235	18.05236	18.05234
52.82533	18.0139	18.01391	18.01389
52.83367	17.96115	17.96116	17.96114
52.842	17.91095	17.91096	17.91094
52.85033	17.8706	17.87061	17.87059
52.85867	17.82475	17.82476	17.82474
52.867	17.7841	17.78411	17.78409
52.87533	17.7366	17.73661	17.73659
52.88367	17.68445	17.68446	17.68444
52.892	17.63855	17.63856	17.63854
52.90033	17.603	17.60301	17.60299
52.90867	17.55155	17.55156	17.55154
52.917	17.5071	17.50711	17.50709
52.92533	17.46345	17.46346	17.46344
52.93367	17.43385	17.43386	17.43384
52.942	17.38985	17.38986	17.38984
52.95033	17.3552	17.35521	17.35519
52.95867	17.32235	17.32236	17.32234
52.967	17.31405	17.31406	17.31404
52.97533	17.28345	17.28346	17.28344
52.98367	17.2618	17.26181	17.26179
52.992	17.2375	17.23751	17.23749
53.00033	17.1989	17.19891	17.19889
53.00867	17.1554	17.15541	17.15539
53.017	17.1117	17.11171	17.11169
53.02533	17.06475	17.06476	17.06474
53.03367	17.02875	17.02876	17.02874
53.042	16.9897	16.98971	16.98969
53.05033	16.94285	16.94286	16.94284
53.05867	16.90085	16.90086	16.90084
53.067	16.863	16.86301	16.86299
53.07533	16.8251	16.82511	16.82509
53.08367	16.78175	16.78176	16.78174
53.092	16.73845	16.73846	16.73844
53.10033	16.70445	16.70446	16.70444
53.10867	16.67135	16.67136	16.67134
53.117	16.62695	16.62696	16.62694
53.12533	16.5994	16.59941	16.59939

53.13367	16.5645	16.56451	16.56449
53.142	16.54185	16.54186	16.54184
53.15033	16.51405	16.51406	16.51404
53.15867	16.4993	16.49931	16.49929
53.167	16.47985	16.47986	16.47984
53.17533	16.46365	16.46366	16.46364
53.18367	16.43535	16.43536	16.43534
53.192	16.39235	16.39236	16.39234
53.20033	16.355	16.35501	16.35499
53.20867	16.3181	16.31811	16.31809
53.217	16.27935	16.27936	16.27934
53.22533	16.24935	16.24936	16.24934
53.23367	16.21065	16.21066	16.21064
53.242	16.17985	16.17986	16.17984
53.25033	16.14825	16.14826	16.14824
53.25867	16.10675	16.10676	16.10674
53.267	16.0752	16.07521	16.07519
53.27533	16.04155	16.04156	16.04154
53.28367	16.00735	16.00736	16.00734
53.292	15.98115	15.98116	15.98114
53.30033	15.94605	15.94606	15.94604
53.30867	15.9142	15.91421	15.91419
53.317	15.8848	15.88481	15.88479
53.32533	15.85845	15.85846	15.85844
53.33367	15.8342	15.83421	15.83419
53.342	15.8209	15.82091	15.82089
53.35033	15.80905	15.80906	15.80904
53.35867	15.7959	15.79591	15.79589
53.367	15.77175	15.77176	15.77174
53.37533	15.74115	15.74116	15.74114
53.38367	15.71235	15.71236	15.71234
53.392	15.67255	15.67256	15.67254
53.40033	15.644	15.64401	15.64399
53.40867	15.61345	15.61346	15.61344
53.417	15.5803	15.58031	15.58029
53.42533	15.5554	15.55541	15.55539
53.43367	15.52145	15.52146	15.52144
53.442	15.49635	15.49636	15.49634
53.45033	15.4596	15.45961	15.45959
53.45867	15.4249	15.42491	15.42489
53.467	15.39835	15.39836	15.39834
53.47533	15.37075	15.37076	15.37074
53.48367	15.3431	15.34311	15.34309
53.492	15.3194	15.31941	15.31939

53.50033	15.2927	15.29271	15.29269
53.50867	15.2691	15.26911	15.26909
53.517	15.2464	15.24641	15.24639
53.52533	15.2283	15.22831	15.22829
53.53367	15.2145	15.21451	15.21449
53.542	15.1968	15.19681	15.19679
53.55033	15.18515	15.18516	15.18514
53.55867	15.16085	15.16086	15.16084
53.567	15.13345	15.13346	15.13344
53.57533	15.0971	15.09711	15.09709
53.58367	15.07535	15.07536	15.07534
53.592	15.0403	15.04031	15.04029
53.60033	15.00685	15.00686	15.00684
53.60867	14.9841	14.98411	14.98409
53.617	14.9587	14.95871	14.95869
53.62533	14.93235	14.93236	14.93234
53.63367	14.8985	14.89851	14.89849
53.642	14.8721	14.87211	14.87209
53.65033	14.84075	14.84076	14.84074
53.65867	14.8089	14.80891	14.80889
53.667	14.792	14.79201	14.79199
53.67533	14.75955	14.75956	14.75954
53.68367	14.73235	14.73236	14.73234
53.692	14.70835	14.70836	14.70834
53.70033	14.68875	14.68876	14.68874
53.70867	14.66795	14.66796	14.66794
53.717	14.65455	14.65456	14.65454
53.72533	14.6378	14.63781	14.63779
53.73367	14.62265	14.62266	14.62264
53.742	14.61475	14.61476	14.61474
53.75033	14.5708	14.57081	14.57079
53.75867	14.5392	14.53921	14.53919
53.767	14.5116	14.51161	14.51159
53.77533	14.4908	14.49081	14.49079
53.78367	14.4541	14.45411	14.45409
53.792	14.4343	14.43431	14.43429
53.80033	14.40985	14.40986	14.40984
53.80867	14.38005	14.38006	14.38004
53.817	14.3556	14.35561	14.35559
53.82533	14.325	14.32501	14.32499
53.83367	14.30015	14.30016	14.30014
53.842	14.271	14.27101	14.27099
53.85033	14.2468	14.24681	14.24679
53.85867	14.22435	14.22436	14.22434

53.867	14.2034	14.20341	14.20339
53.87533	14.17295	14.17296	14.17294
53.88367	14.15175	14.15176	14.15174
53.892	14.1264	14.12641	14.12639
53.90033	14.11325	14.11326	14.11324
53.90867	14.09575	14.09576	14.09574
53.917	14.0795	14.07951	14.07949
53.92533	14.06105	14.06106	14.06104
53.93367	14.03995	14.03996	14.03994
53.942	14.00385	14.00386	14.00384
53.95033	13.98085	13.98086	13.98084
53.95867	13.94965	13.94966	13.94964
53.967	13.92425	13.92426	13.92424
53.97533	13.8963	13.89631	13.89629
53.98367	13.8712	13.87121	13.87119
53.992	13.84965	13.84966	13.84964
54.00033	13.82565	13.82566	13.82564
54.00867	13.80145	13.80146	13.80144
54.017	13.77835	13.77836	13.77834
54.02533	13.75335	13.75336	13.75334
54.03367	13.7212	13.72121	13.72119
54.042	13.6981	13.69811	13.69809
54.05033	13.67455	13.67456	13.67454
54.05867	13.65395	13.65396	13.65394
54.067	13.63155	13.63156	13.63154
54.07533	13.60645	13.60646	13.60644
54.08367	13.58765	13.58766	13.58764
54.092	13.57555	13.57556	13.57554
54.10033	13.5552	13.55521	13.55519
54.10867	13.5362	13.53621	13.53619
54.117	13.51185	13.51186	13.51184
54.12533	13.48685	13.48686	13.48684
54.13367	13.456	13.45601	13.45599
54.142	13.4295	13.42951	13.42949
54.15033	13.4046	13.40461	13.40459
54.15867	13.37995	13.37996	13.37994
54.167	13.3554	13.35541	13.35539
54.17533	13.33295	13.33296	13.33294
54.18367	13.30795	13.30796	13.30794
54.192	13.28405	13.28406	13.28404
54.20033	13.2612	13.26121	13.26119
54.20867	13.2366	13.23661	13.23659
54.217	13.20895	13.20896	13.20894
54.22533	13.18325	13.18326	13.18324

54.23367	13.1591	13.15911	13.15909
54.242	13.13085	13.13086	13.13084
54.25033	13.1099	13.10991	13.10989
54.25867	13.08405	13.08406	13.08404
54.267	13.06675	13.06676	13.06674
54.27533	13.0523	13.05231	13.05229
54.28367	13.0359	13.03591	13.03589
54.292	13.013	13.01301	13.01299
54.30033	12.99185	12.99186	12.99184
54.30867	12.965	12.96501	12.96499
54.317	12.93375	12.93376	12.93374
54.32533	12.9086	12.90861	12.90859
54.33367	12.88	12.88001	12.87999
54.342	12.85485	12.85486	12.85484
54.35033	12.831	12.83101	12.83099
54.35867	12.8086	12.80861	12.80859
54.367	12.78455	12.78456	12.78454
54.37533	12.76205	12.76206	12.76204
54.38367	12.73905	12.73906	12.73904
54.392	12.7143	12.71431	12.71429
54.40033	12.68945	12.68946	12.68944
54.40867	12.66465	12.66466	12.66464
54.417	12.64135	12.64136	12.64134
54.42533	12.6127	12.61271	12.61269
54.43367	12.58795	12.58796	12.58794
54.442	12.5665	12.56651	12.56649
54.45033	12.54255	12.54256	12.54254
54.45867	12.52295	12.52296	12.52294
54.467	12.5032	12.50321	12.50319
54.47533	12.482	12.48201	12.48199
54.48367	12.45995	12.45996	12.45994
54.492	12.4341	12.43411	12.43409
54.50033	12.4025	12.40251	12.40249
54.50867	12.376	12.37601	12.37599
54.517	12.34835	12.34836	12.34834
54.52533	12.32555	12.32556	12.32554
54.53367	12.29855	12.29856	12.29854
54.542	12.2747	12.27471	12.27469
54.55033	12.25325	12.25326	12.25324
54.55867	12.22885	12.22886	12.22884
54.567	12.2066	12.20661	12.20659
54.57533	12.18095	12.18096	12.18094
54.58367	12.1549	12.15491	12.15489
54.592	12.13335	12.13336	12.13334

54.60033	12.1058	12.10581	12.10579
54.60867	12.0795	12.07951	12.07949
54.617	12.05225	12.05226	12.05224
54.62533	12.02625	12.02626	12.02624
54.63367	12.00115	12.00116	12.00114
54.642	11.97665	11.97666	11.97664
54.65033	11.9573	11.95731	11.95729
54.65867	11.93235	11.93236	11.93234
54.667	11.90785	11.90786	11.90784
54.67533	11.88185	11.88186	11.88184
54.68367	11.853	11.85301	11.85299
54.692	11.81965	11.81966	11.81964
54.70033	11.7917	11.79171	11.79169
54.70867	11.7671	11.76711	11.76709
54.717	11.74335	11.74336	11.74334
54.72533	11.7188	11.71881	11.71879
54.73367	11.6946	11.69461	11.69459
54.742	11.6711	11.67111	11.67109
54.75033	11.6476	11.64761	11.64759
54.75867	11.62455	11.62456	11.62454
54.767	11.59895	11.59896	11.59894
54.77533	11.57305	11.57306	11.57304
54.78367	11.54965	11.54966	11.54964
54.792	11.5228	11.52281	11.52279
54.80033	11.49745	11.49746	11.49744
54.80867	11.4711	11.47111	11.47109
54.817	11.44465	11.44466	11.44464
54.82533	11.41935	11.41936	11.41934
54.83367	11.3965	11.39651	11.39649
54.842	11.3734	11.37341	11.37339
54.85033	11.3453	11.34531	11.34529
54.85867	11.31515	11.31516	11.31514
54.867	11.28365	11.28366	11.28364
54.87533	11.2503	11.25031	11.25029
54.88367	11.22315	11.22316	11.22314
54.892	11.19465	11.19466	11.19464
54.90033	11.1683	11.16831	11.16829
54.90867	11.14225	11.14226	11.14224
54.917	11.11665	11.11666	11.11664
54.92533	11.09265	11.09266	11.09264
54.93367	11.0675	11.06751	11.06749
54.942	11.0423	11.04231	11.04229
54.95033	11.0176	11.01761	11.01759
54.95867	10.98945	10.98946	10.98944

54.967	10.96395	10.96396	10.96394
54.97533	10.93785	10.93786	10.93784
54.98367	10.9112	10.91121	10.91119
54.992	10.88345	10.88346	10.88344
55.00033	10.85475	10.85476	10.85474
55.00867	10.8281	10.82811	10.82809
55.017	10.7977	10.79771	10.79769
55.02533	10.77455	10.77456	10.77454
55.03367	10.7433	10.74331	10.74329
55.042	10.714	10.71401	10.71399
55.05033	10.68085	10.68086	10.68084
55.05867	10.64635	10.64636	10.64634
55.067	10.6117	10.61171	10.61169
55.07533	10.58445	10.58446	10.58444
55.08367	10.55635	10.55636	10.55634
55.092	10.527	10.52701	10.52699
55.10033	10.50065	10.50066	10.50064
55.10867	10.4744	10.47441	10.47439
55.117	10.4493	10.44931	10.44929
55.12533	10.4245	10.42451	10.42449
55.13367	10.39755	10.39756	10.39754
55.142	10.3706	10.37061	10.37059
55.15033	10.34475	10.34476	10.34474
55.15867	10.3166	10.31661	10.31659
55.167	10.2901	10.29011	10.29009
55.17533	10.25975	10.25976	10.25974
55.18367	10.23265	10.23266	10.23264
55.192	10.2043	10.20431	10.20429
55.20033	10.17225	10.17226	10.17224
55.20867	10.14415	10.14416	10.14414
55.217	10.11335	10.11336	10.11334
55.22533	10.081	10.08101	10.08099
55.23367	10.04825	10.04826	10.04824
55.242	10.00755	10.00756	10.00754
55.25033	9.97245	9.97246	9.97244
55.25867	9.94225	9.94226	9.94224
55.267	9.9129	9.91291	9.91289
55.27533	9.88395	9.88396	9.88394
55.28367	9.8559	9.85591	9.85589
55.292	9.8304	9.83041	9.83039
55.30033	9.80185	9.80186	9.80184
55.30867	9.7761	9.77611	9.77609
55.317	9.75135	9.75136	9.75134
55.32533	9.7237	9.72371	9.72369

55.33367	9.69505	9.69506	9.69504
55.342	9.6686	9.66861	9.66859
55.35033	9.64125	9.64126	9.64124
55.35867	9.61175	9.61176	9.61174
55.367	9.5804	9.58041	9.58039
55.37533	9.54945	9.54946	9.54944
55.38367	9.51815	9.51816	9.51814
55.392	9.48685	9.48686	9.48684
55.40033	9.45465	9.45466	9.45464
55.40867	9.4166	9.41661	9.41659
55.417	9.38065	9.38066	9.38064
55.42533	9.3414	9.34141	9.34139
55.43367	9.3016	9.30161	9.30159
55.442	9.2672	9.26721	9.26719
55.45033	9.2372	9.23721	9.23719
55.45867	9.20795	9.20796	9.20794
55.467	9.17745	9.17746	9.17744
55.47533	9.15025	9.15026	9.15024
55.48367	9.12255	9.12256	9.12254
55.492	9.0949	9.09491	9.09489
55.50033	9.067	9.06701	9.06699
55.50867	9.0398	9.03981	9.03979
55.517	9.0109	9.01091	9.01089
55.52533	8.98395	8.98396	8.98394
55.53367	8.9535	8.95351	8.95349
55.542	8.92455	8.92456	8.92454
55.55033	8.89135	8.89136	8.89134
55.55867	8.86195	8.86196	8.86194
55.567	8.83015	8.83016	8.83014
55.57533	8.79585	8.79586	8.79584
55.58367	8.76285	8.76286	8.76284
55.592	8.7241	8.72411	8.72409
55.60033	8.68415	8.68416	8.68414
55.60867	8.6436	8.64361	8.64359
55.617	8.601	8.60101	8.60099
55.62533	8.56355	8.56356	8.56354
55.63367	8.5306	8.53061	8.53059
55.642	8.50015	8.50016	8.50014
55.65033	8.47095	8.47096	8.47094
55.65867	8.44145	8.44146	8.44144
55.667	8.41375	8.41376	8.41374
55.67533	8.3854	8.38541	8.38539
55.68367	8.3542	8.35421	8.35419
55.692	8.32755	8.32756	8.32754

55.70033	8.2982	8.29821	8.29819
55.70867	8.27105	8.27106	8.27104
55.717	8.24325	8.24326	8.24324
55.72533	8.2148	8.21481	8.21479
55.73367	8.18325	8.18326	8.18324
55.742	8.15425	8.15426	8.15424
55.75033	8.1215	8.12151	8.12149
55.75867	8.0879	8.08791	8.08789
55.767	8.05365	8.05366	8.05364
55.77533	8.01685	8.01686	8.01684
55.78367	7.97625	7.97626	7.97624
55.792	7.93285	7.93286	7.93284
55.80033	7.89055	7.89056	7.89054
55.80867	7.85015	7.85016	7.85014
55.817	7.81815	7.81816	7.81814
55.82533	7.7884	7.78841	7.78839
55.83367	7.75935	7.75936	7.75934
55.842	7.73035	7.73036	7.73034
55.85033	7.70375	7.70376	7.70374
55.85867	7.6766	7.67661	7.67659
55.867	7.64865	7.64866	7.64864
55.87533	7.62215	7.62216	7.62214
55.88367	7.59545	7.59546	7.59544
55.892	7.5701	7.57011	7.57009
55.90033	7.5435	7.54351	7.54349
55.90867	7.5162	7.51621	7.51619
55.917	7.4881	7.48811	7.48809
55.92533	7.4586	7.45861	7.45859
55.93367	7.43065	7.43066	7.43064
55.942	7.39755	7.39756	7.39754
55.95033	7.3642	7.36421	7.36419
55.95867	7.33045	7.33046	7.33044
55.967	7.2901	7.29011	7.29009
55.97533	7.2498	7.24981	7.24979
55.98367	7.20745	7.20746	7.20744
55.992	7.1668	7.16681	7.16679
56.00033	7.13475	7.13476	7.13474
56.00867	7.1066	7.10661	7.10659
56.017	7.0798	7.07981	7.07979
56.02533	7.05385	7.05386	7.05384
56.03367	7.0304	7.03041	7.03039
56.042	7.0054	7.00541	7.00539
56.05033	6.9788	6.97881	6.97879
56.05867	6.9533	6.95331	6.95329

56.067	6.92955	6.92956	6.92954
56.07533	6.9061	6.90611	6.90609
56.08367	6.8821	6.88211	6.88209
56.092	6.86035	6.86036	6.86034
56.10033	6.8379	6.83791	6.83789
56.10867	6.8102	6.81021	6.81019
56.117	6.7859	6.78591	6.78589
56.12533	6.7567	6.75671	6.75669
56.13367	6.7265	6.72651	6.72649
56.142	6.6976	6.69761	6.69759
56.15033	6.65915	6.65916	6.65914
56.15867	6.52465	6.52466	6.52464
56.167	6.4751	6.47511	6.47509
56.17533	6.43425	6.43426	6.43424
56.18367	6.4014	6.40141	6.40139
56.192	6.37675	6.37676	6.37674
56.20033	6.3535	6.35351	6.35349
56.20867	6.3299	6.32991	6.32989
56.217	6.3079	6.30791	6.30789
56.22533	6.2872	6.28721	6.28719
56.23367	6.26625	6.26626	6.26624
56.242	6.24605	6.24606	6.24604
56.25033	6.2265	6.22651	6.22649
56.25867	6.20665	6.20666	6.20664
56.267	6.18825	6.18826	6.18824
56.27533	6.1694	6.16941	6.16939
56.28367	6.15075	6.15076	6.15074
56.292	6.1292	6.12921	6.12919
56.30033	6.10745	6.10746	6.10744
56.30867	6.0857	6.08571	6.08569
56.317	6.05985	6.05986	6.05984
56.32533	6.03325	6.03326	6.03324
56.33367	6.00455	6.00456	6.00454
56.342	5.9681	5.96811	5.96809
56.35033	5.93395	5.93396	5.93394
56.35867	5.89755	5.89756	5.89754
56.367	5.86585	5.86586	5.86584
56.37533	5.8422	5.84221	5.84219
56.38367	5.8237	5.82371	5.82369
56.392	5.8061	5.80611	5.80609
56.40033	5.7898	5.78981	5.78979
56.40867	5.77125	5.77126	5.77124
56.417	5.75515	5.75516	5.75514
56.42533	5.7376	5.73761	5.73759

56.43367	5.7211	5.72111	5.72109
56.442	5.70545	5.70546	5.70544
56.45033	5.6913	5.69131	5.69129
56.45867	5.67585	5.67586	5.67584
56.467	5.6605	5.66051	5.66049
56.47533	5.6437	5.64371	5.64369
56.48367	5.62605	5.62606	5.62604
56.492	5.60835	5.60836	5.60834
56.50033	5.5874	5.58741	5.58739
56.50867	5.5632	5.56321	5.56319
56.517	5.54115	5.54116	5.54114
56.52533	5.50865	5.50866	5.50864
56.53367	5.4773	5.47731	5.47729
56.542	5.4441	5.44412	5.44409
56.55033	5.4121	5.41212	5.41209
56.55867	5.3908	5.39082	5.39079
56.567	5.3759	5.37592	5.37589
56.57533	5.3623	5.36232	5.36229
56.58367	5.34775	5.34777	5.34774
56.592	5.33455	5.33457	5.33454
56.60033	5.32145	5.32147	5.32144
56.60867	5.31005	5.31007	5.31004
56.617	5.29555	5.29557	5.29554
56.62533	5.2834	5.28342	5.28339
56.63367	5.2714	5.27142	5.27139
56.642	5.26005	5.26007	5.26004
56.65033	5.24895	5.24897	5.24894
56.65867	5.238	5.23802	5.23799
56.667	5.22285	5.22287	5.22284
56.67533	5.20855	5.20857	5.20854
56.68367	5.1893	5.18932	5.18929
56.692	5.17	5.17002	5.16999
56.70033	5.14865	5.14867	5.14864
56.70867	5.12455	5.12457	5.12454
56.717	5.0937	5.09372	5.09369
56.72533	5.0639	5.06392	5.06389
56.73367	5.03215	5.03217	5.03214
56.742	5.01045	5.01047	5.01044
56.75033	4.99725	4.99727	4.99724
56.75867	4.9869	4.98692	4.98689
56.767	4.9763	4.97632	4.97629
56.77533	4.96565	4.96567	4.96564
56.78367	4.956	4.95602	4.95599
56.792	4.94625	4.94627	4.94624

56.80033	4.93595	4.93597	4.93594
56.80867	4.92735	4.92737	4.92734
56.817	4.9178	4.91781	4.91779
56.82533	4.91175	4.91176	4.91174
56.83367	4.90415	4.90416	4.90414
56.842	4.89455	4.89456	4.89454
56.85033	4.88525	4.88526	4.88524
56.85867	4.87535	4.87536	4.87534
56.867	4.86155	4.86156	4.86154
56.87533	4.84895	4.84896	4.84894
56.88367	4.83175	4.83176	4.83174
56.892	4.81335	4.81336	4.81334
56.90033	4.788	4.78801	4.78799
56.90867	4.7624	4.76241	4.76239
56.917	4.73725	4.73726	4.73724
56.92533	4.7152	4.71521	4.71519
56.93367	4.7047	4.70471	4.70469
56.942	4.6997	4.69971	4.69969
56.95033	4.69385	4.69386	4.69384
56.95867	4.6891	4.68911	4.68909
56.967	4.68375	4.68376	4.68374
56.97533	4.6794	4.67941	4.67939
56.98367	4.67155	4.67156	4.67154
56.992	4.66415	4.66416	4.66414
57.00033	4.66235	4.66236	4.66234
57.00867	4.65905	4.65906	4.65904
57.017	4.65555	4.65556	4.65554
57.02533	4.65315	4.65316	4.65314
57.03367	4.6497	4.64971	4.64969
57.042	4.6433	4.64331	4.64329
57.05033	4.63695	4.63696	4.63694
57.05867	4.62795	4.62796	4.62794
57.067	4.61625	4.61626	4.61624
57.07533	4.60265	4.60266	4.60264
57.08367	4.58305	4.58306	4.58304
57.092	4.5622	4.56221	4.56219
57.10033	4.5401	4.54011	4.54009
57.10867	4.52125	4.52126	4.52124
57.117	4.51435	4.51436	4.51434
57.12533	4.5115	4.51151	4.51149
57.13367	4.5093	4.50931	4.50929
57.142	4.50735	4.50736	4.50734
57.15033	4.50575	4.50576	4.50574
57.15867	4.50335	4.50336	4.50334

57.167	4.50195	4.50196	4.50194
57.17533	4.5006	4.50061	4.50059
57.18367	4.49765	4.49766	4.49764
57.192	4.49765	4.49766	4.49764
57.20033	4.4985	4.49851	4.49849
57.20867	4.5001	4.50011	4.50009
57.217	4.4992	4.49921	4.49919
57.22533	4.496	4.49601	4.49599
57.23367	4.4923	4.49231	4.49229
57.242	4.4868	4.48681	4.48679
57.25033	4.47695	4.47696	4.47694
57.25867	4.4669	4.46691	4.46689
57.267	4.4542	4.45421	4.45419
57.27533	4.43345	4.43346	4.43344
57.28367	4.41455	4.41456	4.41454
57.292	4.39795	4.39796	4.39794
57.30033	4.38695	4.38696	4.38694
57.30867	4.3854	4.38541	4.38539
57.317	4.3862	4.38621	4.38619
57.32533	4.38615	4.38616	4.38614
57.33367	4.38505	4.38506	4.38504
57.342	4.38675	4.38676	4.38674
57.35033	4.3872	4.38721	4.38719
57.35867	4.3852	4.38521	4.38519
57.367	4.38505	4.38506	4.38504
57.37533	4.387	4.38702	4.38699
57.38367	4.38915	4.38917	4.38914
57.392	4.3906	4.39062	4.39059
57.40033	4.3926	4.39262	4.39259
57.40867	4.3915	4.39152	4.39149
57.417	4.38935	4.38937	4.38934
57.42533	4.38525	4.38527	4.38524
57.43367	4.37755	4.37757	4.37754
57.442	4.36905	4.36907	4.36904
57.45033	4.3589	4.35892	4.35889
57.45867	4.3395	4.33952	4.33949
57.467	4.3217	4.32172	4.32169
57.47533	4.30315	4.30317	4.30314
57.48367	4.2895	4.28952	4.28949
57.492	4.2867	4.28672	4.28669
57.50033	4.29015	4.29017	4.29014
57.50867	4.29085	4.29087	4.29084
57.517	4.2908	4.29082	4.29079
57.52533	4.2918	4.29182	4.29179

57.53367	4.29235	4.29237	4.29234
57.542	4.2915	4.29152	4.29149
57.55033	4.2918	4.29182	4.29179
57.55867	4.2924	4.29242	4.29239
57.567	4.29415	4.29417	4.29414
57.57533	4.2962	4.29622	4.29619
57.58367	4.2973	4.29732	4.29729
57.592	4.2981	4.29812	4.29809
57.60033	4.29575	4.29576	4.29574
57.60867	4.2931	4.29311	4.29309
57.617	4.286	4.28601	4.28599
57.62533	4.2776	4.27761	4.27759
57.63367	4.26745	4.26746	4.26744
57.642	4.25075	4.25076	4.25074
57.65033	4.2323	4.23231	4.23229
57.65867	4.2138	4.21381	4.21379
57.667	4.1975	4.19751	4.19749
57.67533	4.1912	4.19121	4.19119
57.68367	4.19265	4.19266	4.19264
57.692	4.1927	4.19271	4.19269
57.70033	4.193	4.19301	4.19299
57.70867	4.19345	4.19346	4.19344
57.717	4.19455	4.19456	4.19454
57.72533	4.19355	4.19356	4.19354
57.73367	4.1901	4.19011	4.19009
57.742	4.192	4.19201	4.19199
57.75033	4.19375	4.19376	4.19374
57.75867	4.195	4.19501	4.19499
57.767	4.1964	4.19641	4.19639
57.77533	4.1966	4.19661	4.19659
57.78367	4.19355	4.19356	4.19354
57.792	4.18995	4.18996	4.18994
57.80033	4.18405	4.18406	4.18404
57.80867	4.17555	4.17556	4.17554
57.817	4.16485	4.16486	4.16484
57.82533	4.15	4.15001	4.14999
57.83367	4.1301	4.13011	4.13009
57.842	4.111	4.11101	4.11099
57.85033	4.0909	4.09091	4.09089
57.85867	4.08035	4.08036	4.08034
57.867	4.0793	4.07931	4.07929
57.87533	4.08085	4.08086	4.08084
57.88367	4.0812	4.08121	4.08119
57.892	4.08105	4.08106	4.08104

57.90033	4.08125	4.08126	4.08124
57.90867	4.0811	4.08111	4.08109
57.917	4.08105	4.08106	4.08104
57.92533	4.07815	4.07816	4.07814
57.93367	4.0777	4.07771	4.07769
57.942	4.0788	4.07881	4.07879
57.95033	4.08	4.08001	4.07999
57.95867	4.0805	4.08051	4.08049
57.967	4.0786	4.07861	4.07859
57.97533	4.07395	4.07396	4.07394
57.98367	4.06985	4.06986	4.06984
57.992	4.0614	4.06141	4.06139
58.00033	4.0505	4.05051	4.05049
58.00867	4.03675	4.03676	4.03674
58.017	4.0163	4.01631	4.01629
58.02533	3.9948	3.99481	3.99479
58.03367	3.9742	3.97421	3.97419
58.042	3.95845	3.95846	3.95844
58.05033	3.9533	3.95331	3.95329
58.05867	3.9542	3.95421	3.95419
58.067	3.9555	3.95551	3.95549
58.07533	3.9554	3.95541	3.95539
58.08367	3.9528	3.95281	3.95279
58.092	3.95295	3.95296	3.95294
58.10033	3.9488	3.94881	3.94879
58.10867	3.94685	3.94686	3.94684
58.117	3.94595	3.94596	3.94594
58.12533	3.94475	3.94476	3.94474
58.13367	3.94325	3.94326	3.94324
58.142	3.94445	3.94446	3.94444
58.15033	3.94305	3.94306	3.94304
58.15867	3.93785	3.93786	3.93784
58.167	3.933	3.93301	3.93299
58.17533	3.92505	3.92506	3.92504
58.18367	3.91405	3.91406	3.91404
58.192	3.9011	3.90111	3.90109
58.20033	3.8823	3.88231	3.88229
58.20867	3.85955	3.85956	3.85954
58.217	3.83665	3.83666	3.83664
58.22533	3.8177	3.81771	3.81769
58.23367	3.8073	3.80731	3.80729
58.242	3.807	3.80701	3.80699
58.25033	3.8067	3.80671	3.80669
58.25867	3.80515	3.80516	3.80514

58.267	3.80435	3.80436	3.80434
58.27533	3.80255	3.80256	3.80254
58.28367	3.8022	3.80221	3.80219
58.292	3.79925	3.79926	3.79924
58.30033	3.7962	3.79621	3.79619
58.30867	3.7948	3.79481	3.79479
58.317	3.79575	3.79576	3.79574
58.32533	3.795	3.79501	3.79499
58.33367	3.79265	3.79266	3.79264
58.342	3.7884	3.78841	3.78839
58.35033	3.78385	3.78386	3.78384
58.35867	3.77655	3.77656	3.77654
58.367	3.76525	3.76526	3.76524
58.37533	3.75365	3.75366	3.75364
58.38367	3.7361	3.73611	3.73609
58.392	3.71115	3.71116	3.71114
58.40033	3.68965	3.68966	3.68964
58.40867	3.66765	3.66766	3.66764
58.417	3.6519	3.65191	3.65189
58.42533	3.6494	3.64941	3.64939
58.43367	3.65115	3.65116	3.65114
58.442	3.6493	3.64931	3.64929
58.45033	3.6465	3.64651	3.64649
58.45867	3.64595	3.64596	3.64594
58.467	3.6432	3.64321	3.64319
58.47533	3.63745	3.63746	3.63744
58.48367	3.63405	3.63406	3.63404
58.492	3.6335	3.63351	3.63349
58.50033	3.63275	3.63276	3.63274
58.50867	3.6317	3.63171	3.63169
58.517	3.6327	3.63271	3.63269
58.52533	3.6293	3.62931	3.62929
58.53367	3.6241	3.62411	3.62409
58.542	3.6178	3.61781	3.61779
58.55033	3.6071	3.60711	3.60709
58.55867	3.59405	3.59406	3.59404
58.567	3.57955	3.57956	3.57954
58.57533	3.557	3.55701	3.55699
58.58367	3.53355	3.53356	3.53354
58.592	3.51015	3.51016	3.51014
58.60033	3.4902	3.49021	3.49019
58.60867	3.48335	3.48336	3.48334
58.617	3.4838	3.48381	3.48379
58.62533	3.48245	3.48246	3.48244

58.63367	3.48025	3.48026	3.48024
58.642	3.4779	3.47791	3.47789
58.65033	3.47665	3.47666	3.47664
58.65867	3.4731	3.47311	3.47309
58.667	3.4702	3.47021	3.47019
58.67533	3.46845	3.46846	3.46844
58.68367	3.46635	3.46636	3.46634
58.692	3.4677	3.46771	3.46769
58.70033	3.46935	3.46936	3.46934
58.70867	3.46685	3.46686	3.46684
58.717	3.4603	3.46031	3.46029
58.72533	3.45565	3.45566	3.45564
58.73367	3.44585	3.44586	3.44584
58.742	3.4339	3.43391	3.43389
58.75033	3.41875	3.41876	3.41874
58.75867	3.3988	3.39881	3.39879
58.767	3.3731	3.37311	3.37309
58.77533	3.3494	3.34941	3.34939
58.78367	3.3267	3.32671	3.32669
58.792	3.3134	3.31341	3.31339
58.80033	3.31105	3.31106	3.31104
58.80867	3.3116	3.31161	3.31159
58.817	3.3103	3.31031	3.31029
58.82533	3.30775	3.30776	3.30774
58.83367	3.30635	3.30636	3.30634
58.842	3.303	3.30301	3.30299
58.85033	3.29605	3.29606	3.29604
58.85867	3.2939	3.29391	3.29389
58.867	3.29225	3.29226	3.29224
58.87533	3.2925	3.29251	3.29249
58.88367	3.2914	3.29141	3.29139
58.892	3.29025	3.29026	3.29024
58.90033	3.28535	3.28536	3.28534
58.90867	3.28105	3.28106	3.28104
58.917	3.2739	3.27391	3.27389
58.92533	3.2629	3.26291	3.26289
58.93367	3.249	3.24901	3.24899
58.942	3.23025	3.23026	3.23024
58.95033	3.20565	3.20566	3.20564
58.95867	3.1806	3.18061	3.18059
58.967	3.15535	3.15536	3.15534
58.97533	3.13775	3.13776	3.13774
58.98367	3.13235	3.13236	3.13234
58.992	3.13225	3.13226	3.13224

59.00033	3.1299	3.12991	3.12989
59.00867	3.1268	3.12681	3.12679
59.017	3.12465	3.12466	3.12464
59.02533	3.12095	3.12096	3.12094
59.03367	3.11645	3.11646	3.11644
59.042	3.1146	3.11461	3.11459
59.05033	3.1121	3.11211	3.11209
59.05867	3.1111	3.11111	3.11109
59.067	3.10915	3.10916	3.10914
59.07533	3.10845	3.10846	3.10844
59.08367	3.1049	3.10491	3.10489
59.092	3.09845	3.09846	3.09844
59.10033	3.0915	3.09151	3.09149
59.10867	3.07975	3.07976	3.07974
59.117	3.0648	3.06481	3.06479
59.12533	3.0493	3.04931	3.04929
59.13367	3.026	3.02601	3.02599
59.142	2.99865	2.99866	2.99864
59.15033	2.9728	2.97281	2.97279
59.15867	2.94845	2.94846	2.94844
59.167	2.9377	2.93771	2.93769
59.17533	2.9356	2.93561	2.93559
59.18367	2.9339	2.93391	2.93389
59.192	2.93115	2.93116	2.93114
59.20033	2.92775	2.92776	2.92774
59.20867	2.9262	2.92621	2.92619
59.217	2.92205	2.92206	2.92204
59.22533	2.9183	2.91831	2.91829
59.23367	2.91555	2.91556	2.91554
59.242	2.912	2.91201	2.91199
59.25033	2.91025	2.91026	2.91024
59.25867	2.91065	2.91066	2.91064
59.267	2.90835	2.90836	2.90834
59.27533	2.90155	2.90156	2.90154
59.28367	2.8954	2.89541	2.89539
59.292	2.88645	2.88646	2.88644
59.30033	2.8717	2.87171	2.87169
59.30867	2.8565	2.85651	2.85649
59.317	2.83595	2.83596	2.83594
59.32533	2.809	2.80901	2.80899
59.33367	2.782	2.78201	2.78199
59.342	2.7565	2.75651	2.75649
59.35033	2.7386	2.73861	2.73859
59.35867	2.7369	2.73691	2.73689

59.367	2.73695	2.73696	2.73694
59.37533	2.73405	2.73406	2.73404
59.38367	2.73005	2.73006	2.73004
59.392	2.7294	2.72941	2.72939
59.40033	2.72475	2.72476	2.72474
59.40867	2.71995	2.71996	2.71994
59.417	2.7148	2.71481	2.71479
59.42533	2.71535	2.71536	2.71534
59.43367	2.7152	2.71521	2.71519
59.442	2.71265	2.71266	2.71264
59.45033	2.71005	2.71006	2.71004
59.45867	2.70575	2.70576	2.70574
59.467	2.6996	2.69961	2.69959
59.47533	2.692	2.69201	2.69199
59.48367	2.6803	2.68031	2.68029
59.492	2.66625	2.66626	2.66624
59.50033	2.64905	2.64906	2.64904
59.50867	2.6218	2.62181	2.62179
59.517	2.59535	2.59536	2.59534
59.52533	2.57025	2.57026	2.57024
59.53367	2.5477	2.54771	2.54769
59.542	2.53925	2.53926	2.53924
59.55033	2.5394	2.53941	2.53939
59.55867	2.5378	2.53781	2.53779
59.567	2.53495	2.53496	2.53494
59.57533	2.53155	2.53156	2.53154
59.58367	2.53015	2.53016	2.53014
59.592	2.5231	2.52311	2.52309
59.60033	2.52015	2.52016	2.52014
59.60867	2.5176	2.51761	2.51759
59.617	2.51595	2.51596	2.51594
59.62533	2.51695	2.51696	2.51694
59.63367	2.5181	2.51811	2.51809
59.642	2.51475	2.51476	2.51474
59.65033	2.5087	2.50871	2.50869
59.65867	2.50285	2.50286	2.50284
59.667	2.4932	2.49321	2.49319
59.67533	2.47835	2.47836	2.47834
59.68367	2.46265	2.46266	2.46264
59.692	2.43985	2.43986	2.43984
59.70033	2.41255	2.41256	2.41254
59.70867	2.3859	2.38591	2.38589
59.717	2.3612	2.36121	2.36119
59.72533	2.3469	2.34691	2.34689

59.73367	2.3454	2.34541	2.34539
59.742	2.3469	2.34691	2.34689
59.75033	2.34475	2.34476	2.34474
59.75867	2.3428	2.34281	2.34279
59.767	2.34025	2.34026	2.34024
59.77533	2.33785	2.33786	2.33784
59.78367	2.33415	2.33416	2.33414
59.792	2.3315	2.33151	2.33149
59.80033	2.3317	2.33171	2.33168
59.80867	2.33115	2.33116	2.33113
59.817	2.3293	2.32931	2.32928
59.82533	2.3285	2.32851	2.32848
59.83367	2.32405	2.32406	2.32403
59.842	2.31895	2.31896	2.31893
59.85033	2.3107	2.31071	2.31068
59.85867	2.2985	2.29851	2.29848
59.867	2.2837	2.28371	2.28368
59.87533	2.26455	2.26456	2.26453
59.88367	2.23645	2.23646	2.23643
59.892	2.2103	2.21031	2.21028
59.90033	2.18405	2.18406	2.18403
59.90867	2.1639	2.16391	2.16388
59.917	2.15945	2.15946	2.15943
59.92533	2.1617	2.16171	2.16168
59.93367	2.16045	2.16046	2.16043
59.942	2.1582	2.15821	2.15818
59.95033	2.15635	2.15636	2.15633
59.95867	2.15455	2.15456	2.15453
59.967	2.1478	2.14781	2.14778
59.97533	2.1479	2.14791	2.14788
59.98367	2.1474	2.14741	2.14738
59.992	2.14805	2.14806	2.14803
60.00033	2.14825	2.14826	2.14823
